# Global burden of injuries attributable to alcohol consumption in 2004: a novel way of calculating the burden of injuries attributable to alcohol consumption

**DOI:** 10.1186/1478-7954-10-9

**Published:** 2012-05-18

**Authors:** Kevin D Shield, Gerrit Gmel, Jayadeep Patra, Jürgen Rehm

**Affiliations:** 1 Centre for Addiction and Mental Health (CAMH), Toronto, Canada; 2 Institute of Medical Science, University of Toronto, Toronto, Canada; 3 Ecole Polytechnique Fédérale de Lausanne, Lausanne, Switzerland; 4 Dalla Lana School of Public Health (DLSPH), University of Toronto, Toronto, Canada; 5 Institute for Clinical Psychology and Psychotherapy, TU Dresden, Dresden, Germany; 6 Department of Psychiatry, University of Toronto, Toronto, Canada

**Keywords:** Alcohol, Injury, Attributable fraction, Burden of disease, Mortality, Years of potential life lost

## Abstract

****Background**:**

Alcohol consumption is a major risk factor for injuries; however, international data on this burden are limited. This article presents new methods to quantify the burden of injuries attributable to alcohol consumption and quantifies the number of deaths, potential years of life lost (PYLL), and disability-adjusted life years (DALYs) lost from injuries attributable to alcohol consumption for 2004.

****Methods**:**

Data on drinking indicators were obtained from the Comparative Risk Assessment study. Data on mortality, PYLL, and DALYs for injuries were obtained from the World Health Organization. Alcohol-attributable fractions were calculated based on a new risk modeling methodology, which accounts for average and heavy drinking occasions. 95% confidence intervals (CIs) were calculated using a Monte Carlo simulation method.

****Results**:**

In 2004, 851,900 (95% CI: 419,400 to 1,282,500) deaths, 19,051,000 (95% CI: 9,767,000 to 28,243,000) PYLL, and 21,688,000 (95% CI: 11,097,000 to 32,385,000) DALYs for people 15 years and older were due to injuries attributable to alcohol consumption. With respect to the total number of deaths, harms to others were responsible for 15.1% of alcohol-attributable injury deaths, 14.5% of alcohol-attributable injury PYLL, and 11.35% of alcohol-attributable injury DALYs. The overall burden of injuries attributable to alcohol consumption corresponds to 17.3% of all injury deaths, 16.7% of all PYLL, and 13.6% of all DALYs caused by injuries, or 1.4% of all deaths, 2.0% of all PYLL, and 1.4% of all DALYs in 2004.

****Conclusions**:**

The novel methodology described in this article to calculate the burden of injuries attributable to alcohol consumption improves on previous methodology by more accurately calculating the burden of injuries attributable to one’s own drinking, and for the first time, calculates the burden of injuries attributable to the alcohol consumption of others. The burden of injuries attributable to alcohol consumption is large and is entirely avoidable, and policies and strategies to reduce it are recommended.

## **Introduction**

Alcohol consumption is the sixth leading cause of death and the third leading cause of disability-adjusted life years (DALYs) lost globally, with injuries accounting for a large part of the alcohol-attributable burden of disease [1,2]. Previous research, with varying study designs such as cross-sectional studies [3], case-crossover studies [4], case–control studies [5], systematic reviews, and meta-analyses [6,7] has shown a strong association between alcohol consumption and many types of intentional and unintentional injuries. Moreover, it has been shown that alcohol consumption fulfills the standard epidemiological criteria of causality for many injury outcomes [8]. However, reporting of the burden of injuries has not kept pace with new methodology to calculate alcohol-attributable risk. Previous global estimates of the alcohol-attributable burden of injuries were calculated using simplistic methods based on one country and then scaling these estimates according to average volume of consumption and patterns of drinking [9]. The most recently proposed method of calculating the alcohol-attributable burden of disease did not account for injuries caused by other people’s drinking (the burden of which has been estimated to be substantial [10,11]) or account for the overlap in binge consumption and average consumption, which leads to an overestimation of the alcohol-attributable injuries caused to the drinker [12].

In this article we present new methodology to calculate the alcohol-attributable burden of disease for injuries using formulas that take into consideration the main drivers of alcohol-attributable risk – namely average daily consumption and binge drinking. This calculation of the global burden of injuries attributable to alcohol is possibly due, in part, to the persistent relationship between alcohol consumption and injury risk which has remained strikingly similar throughout the last 50 years of observational research [13,14] and across cultural and geographical boundaries [15-18]. This means that, although drinking patterns may vary across countries, cultures, and age and sex groups, the same risk function can be used for all countries to determine injury burden estimates based on country-specific drinking behavior. This information can be aggregated to regional or global levels to gain a more accurate comparative picture.

It is the aim of this article to utilize the new methodology outlined herein to estimate the burden of alcohol-attributable injuries caused to the drinker and to others, and to estimate for 2004 the global burden of injuries attributable to alcohol consumption in terms of mortality, potential years of life lost (PYLL), and DALYs for each Global Burden of Disease (GBD) region.

## **Methods**

Our methodology has two steps: [1] calculation of the injury-, sex-, age-, consumption-, and region-specific alcohol-attributable fractions (AAFs) and [2] application of these AAFs to mortality, PYLL, and DALY data.

### **Definition of regions and population data**

The GBD regions (2005) are based on geography, child and adult mortality, and major causes of death [19]. Population estimates by country in 2004 were based on data obtained from the 2008 revisions of the United Nations Population Division [20].

#### *Step 1: Calculation of the AAFs by sex and alcohol consumption*

##### **Alcohol consumption measures**

Two dimensions of alcohol consumption play a role in affecting the probability of injury: binge drinking and average daily alcohol consumption.

A binge drinker was defined as a person who consumed at least five drinks (for men) or four drinks (for women) of alcohol on at least one occasion in the past month, assuming that the average drink size is 12 g of pure ethanol. Estimates for the prevalence of binge drinkers, current drinkers, and past year abstainers were obtained from the 2005 Comparative Risk Assessment (CRA) study [19].

Average daily alcohol consumption was calculated based on 80% of per capita consumption of alcohol (to account for alcohol not consumed) and the prevalence of current drinkers. Total adult (age 15 years and over) per capita alcohol consumption for 2004 for each region was calculated by adding the estimated recorded and unrecorded adult per capita consumption and then subtracting tourist (the amount of alcohol consumed by citizens of other countries) adult per capita consumption [21].

Estimates of recorded adult per capita alcohol consumption were obtained from the Global Information System on Alcohol and Health database [22]. These estimates were based on government records (taxation), industry publications for the production and sales of alcohol, and data from the Food and Agriculture Organization [22]. Unrecorded and tourist adult per capita consumption estimates were taken from the ongoing CRA study [22]. The main sources for unrecorded consumption were home production, alcohol intended for industrial, technical, and medical uses, and illegal production or importation of alcohol [22]. As no variance estimates for unrecorded and tourist alcohol consumption existed, we estimated the variance to be five times that of the variance of recorded alcohol consumption proportionate to the mean [23].

For this study we used two different types of drinking days: binge drinking days and average drinking days. Prevalence of binge drinkers and the frequency of binge drinking days and alcohol consumption on binge drinking days (for binge drinkers) were obtained from the 2005 CRA study. Average alcohol consumption on nonbinge drinking days (defined as a drinking day that was not a binge drinking day) was assumed to be the same for binge drinkers and nonbinge drinkers. Average alcohol consumption on nonbinge drinking days was calculated such that every day that a person was not binge drinking was considered to be an average drinking day. The volume of alcohol consumed on an average drinking day was then calculated using per capita consumption and binge drinking consumption, such that the amount of alcohol consumed on the nonbinge drinking days plus the amount of alcohol consumed on binge drinking days was equal to 80% of per capita alcohol consumption.

### **Risk relations**

Sources for relative risk (RR) functions by GBD code are outlined in Table 1. Alcohol-attributable harms were calculated if a meta-analysis existed. The RR functions for injuries, expressed as a function of alcohol consumption in grams per occasion (x), are as follows [6]:$RR_{\mathrm{MVA}}:1n(RR_{\mathrm{MVA}})=3.292589*{\left(\frac{x+0.004}{100}\right)}^{2}$$RR_{Non-MVA}:1n(RR_{Non-MVA})=2.189702*{\left(\frac{x+0.004}{100}\right)}^{0.5}$where RR_MVA_ represents the RR for motor vehicle accidents, and RR_Non-MVA_ represents the RR for nonmotor vehicle accidents. These RR functions are based on epidemiological studies that measure postinjury blood alcohol content and, thus, can be used to calculate a person’s risk on an average drinking day and on a binge drinking day.

**Table 1:** Injury categories and the source of the relative risk relationships with alcohol consumption

**Condition**	**GBD code**	**ICD-10 codes**	**Relative risk source**
Unintentional injuries	III A		
Motor vehicle accidents	III A 1	§	Taylor et al., 2010 [6] for relative risk
Poisonings	III A 2	X40-X49	Taylor et al., 2010 [6] for relative risk
Falls	III A 3	W00-W19	Taylor et al., 2010 [6] for relative risk
Fires	III A 4	X00-X09	Taylor et al., 2010 [6] for relative risk
Drowning	III A 5	W65-W74	Taylor et al., 2010 [6] for relative risk
Other Unintentional injuries	III A 6	†Rest of V-series and W20-W64, W 75-W99, X10-X39, X50-X59, Y40-Y86, Y88, and Y89	Taylor et al., 2010 [6] for relative risk
Intentional injuries	III B		
Self-inflicted injuries	III B 1	X60-X84 and Y87.0	Taylor et al., 2010 [6] for relative risk
Violence	III B 2	X85-Y09, Y87.1	Taylor et al., 2010 [6] for relative risk
Other intentional injuries	III B 4	†	Taylor et al., 2010 [6] for relative risk

§ V021–V029, V031–V039, V041–V049, V092, V093, V123–V129, V133–V139, V143–V149, V194–V196, V203–V209, V213–V219, V223–V229, V233–V239, V243–V249,V253–V259, V263–V269, V273– V279, V283–V289, V294–V299, V304–V309, V314–V319, V324–V329, V334–V339, V344–V349, V354–V359, V364–V369, V374–V379, V384–V389, V394–V399, V404–V409, V414–V419, V424–V429, V434–V439, V444–V449, V454–V459, V464– V469, V474–V479, V484–V489, V494–V499, V504–V509, V514–V519, V524–V529, V534–V539, V544–V549, V554–V559, V564–V569, V574–V579, V584–V589, V594–V599, V604–V609, V614–V619, V624–V629, V634–V639, V644–V649, V654– V659, V664–V669, V674–V679, V684–V689, V694–V699, V704–V709, V714–V719, V724–V729, V734–V739, V744–V749, V754–V759, V764–V769, V774–V779, V784–V789, V794–V799, V803–V805, V811, V821, V830–V833, V840–V843, V850– V853, V860–V863, V870–V878, V892. †Rest of V = V-series MINUS §.

### **Estimating the AAFs for harms caused to oneself**

The AAFs for injuries were modeled according to methodology that takes into account two dimensions of alcohol consumption:

1 binge drinking (both the number of occasions and the amount consumed per occasion)

2 average daily alcohol consumption (on nonbinge drinking days)

When calculating the AAFs, we also included alcohol metabolism rates for men and women to calculate a person’s time at risk of an injury outcome, according to methods outlined by Taylor and colleagues [12].

The AAFs for intentional and unintentional injuries attributable to alcohol consumption were calculated as follows: $AAF=\frac{P_{\mathrm{abs}}+P_{current(non-binge)}RR_{current(non-binge)}+P_{current(binge)}RR_{current(binge)}-1}{P_{\mathrm{abs}}+P_{current(non-binge)}RR_{current(non-binge)}+P_{current(binge)}RR_{current(binge)}}$ where P_abs_ represents the prevalence of current abstainers, and P_current(binge)_ and P_current(non-binge)_ are the prevalence of current drinkers who engage in binge drinking and the prevalence of current drinkers who do not engage in binge drinking, respectively. The RRs were calculated separately for current drinkers who engage in binge drinking and current drinkers who do not engage in binge drinking. RR_current(non-binge)_ was calculated as follows:$RR_{current(non-binge)}=(RR_{average}-1)*P_{\mathrm{nonbingedays}}+1$ and RR_current(binge)_ was calculated as follows:$RR_{current(binge)}=(RR_{\mathrm{average}}-1)*P_{\mathrm{nonbingedays}}+(RR_{\mathrm{binge}}-1)*P_{\mathrm{bingedays}}+1$ where risk on average drinking days (RR_average_) was calculated as follows:$RR_{\mathrm{average}}=P_{\mathrm{dayatrisk}}(x)*(RR_{\mathrm{injury}}(x)-1)+1$ and where risk on binge drinking days (RR_binge_) was calculated as follows:$RR_{\mathrm{binge}}=P_{\mathrm{dayatrisk}}(x)*(RR_{\mathrm{injury}}(x)-1)+1$ where P_dayatrisk_ represents the proportion of a day at risk given an alcohol consumption on that day (x), and RR_injury_ is the relative risk for injury given an amount of alcohol consumed (x), where x is the amount of alcohol consumed on binge drinking days for RR_binge_ and the amount of alcohol consumed on nonbinge drinking days for RR_average_*P_dayatrisk_ is calculated based on the average rate at which alcohol is metabolized.

Since these AAFs were calculated based on samples of emergency room patients, we estimated the AAF for mortality from motor vehicle accidents by multiplying the AAF for morbidity for motor vehicle accidents by 3/2. Similarly, to estimate the AAF for mortality due to nonmotor vehicle accidents, we multiplied the AAF for morbidity for nonmotor vehicle accidents by 9/4. These methods were based on two studies that compared blood alcohol levels of emergency room patients with blood alcohol levels obtained from coroners’ reports of patients who died from an injury [24,25].

For women, the AAF for motor vehicle accidents was calculated by multiplying the AAF for motor vehicle accidents for men by the product of the per capita consumption of alcohol for women divided by the per capita consumption of alcohol for men.

### **Estimating the AAFs for harms caused to others**

The AAFs for deaths and morbidity caused by drinkers to others due to motor vehicle accidents were calculated based on recent data reported by Laslett et al., 2011 [11]. The AAFs for the alcohol-attributable injuries to others were calculated as follows: $AAF_{\mathrm{Otherage}}=(1-AAF_{\mathrm{selfagecountryi}})*\left(1-\exp\left[1n\left(1-AAF_{\mathrm{otherageAustralia}}\right)*\frac{AAF_{\mathrm{selfcountryi}}}{AAF_{\mathrm{sefAustralia}}}\right]\right)$where AAF_Otherage_ represents the AAF for motor vehicle accident injuries caused by others, AAF_selfcountryi_ represents the AAF for motor vehicle accident injuries caused to oneself for an entire country i, and AAF_selfagecountryi_ represents the AAF for motor vehicle accident injuries caused to oneself for each specific age group. AAF_selfAustralia_ represents the AAF for motor vehicle accident injuries caused to oneself in Australia, and AAF_otherageAustralia_ represents the AAF for motor vehicle accident injuries caused by others for each specific age group in Australia.

The AAFs for deaths and injuries caused by an assault by someone who had been drinking were calculated based on recent data reported by Laslett et al., 2011. These AAFs were calculated as follows:$AAF_{Assaultage\backslash \_countryi}=AAF_{Assaultage\backslash \_Australia}*(AAF_{Assault\backslash \_countryi}/AAF_{Assault\backslash \_Australia})$ where AAF_Assaultage_countryi_ represents the age-specific AAF for deaths or injuries caused by assault, AAF_Assault_countryi_ represents the AAF for assaults for an entire country, AAF_Assault_Australia_ represents the AAF for deaths or injuries caused by assaults for Australia and AAF_Assaultage_Australiai_ represents the AAF for deaths or injuries caused by assaults for each specific age group in Australia.

### **Estimating the 95% confidence intervals for the AAFs**

The 95% confidence intervals (CIs) for the AAFs were calculated using a Monte Carlo approach, with 40,000 simulations that estimated the lowest-level parameters used in the AAF formulas [23]. For the AAFs of binge and average consumption for each simulation, we generated estimates for the prevalence of past year abstainers from which a prevalence of current drinkers was estimated. Additionally, we generated estimates of the RR function betas and the formula used to calculate the average rate at which alcohol is metabolized, which, in turn, was used to calculate P_dayatrisk_. For the calculation of the 95% CIs, we also generated estimates for the prevalence of binge drinkers among current drinkers and the average number of drinks consumed on binge drinking occasions and on nonbinge drinking occasions. The resulting 40,000 AAF estimates for binge and average consumption were used to calculate the variance of the AAFs and the 95% CIs for each disease category.

#### *Step 2: Application of the AAFs to region-specific mortality, PYLL, and DALY data*

This step required multiplying the sex-, age-, consumption- ,and injury-specific AAFs by mortality, PYLL, and DALY data, respectively.

##### **Estimates of mortality and morbidity**

To quantify the burden of injuries attributable to alcohol consumption we used an event-based measure (mortality) and time-based measures (PYLL and DALYs). DALYs combine years of life lost due to premature mortality and years lived with disabilities. Comprehensive revision estimates for 2004 of mortality, PYLL, and DALYs for the 160 GBD disease and injury categories were provided by the World Health Organization (WHO)[26]. Methods to estimate the mortality, PYLL, and DALYs in the GBD project are described elsewhere [27,28]. Estimates of mortality, PYLL, and DALYs were available for each country, which were used to calculate regional estimates. This meant that for each region, sex-, age-, injury-, and consumption-specific AAFs were applied to sex-, age-, and injury-specific outcome data.

All statistics and analyses were performed using R version 2.11.1.

## **Results**

Table 2 outlines the prevalence of current drinkers, people who engage in binge drinking, average number of binge drinking days in a year, the average number of drinks consumed during a binge drinking occasion, and the per capita consumption by sex for each CRA region. Men exhibited a higher per capita consumption and a higher prevalence of current drinkers and of binge drinkers than did women in every region. The prevalence of current drinkers and per capita consumption varied greatly, with Western Europe having the highest prevalence of current drinkers and North Africa/Middle East having the lowest prevalence of current drinkers. Eastern Europe had the highest per capita consumption for men and women, while Southern Asia had the lowest per capita consumption for women and North Africa/Middle East had the lowest for men.

**Table 2:** Drinking indicators by Global Burden of Disease region for 2005

	**Men**					**Women**				
**GBD Region**	**Current drinkers**	**Prevalence of binge drinkers [among current drinkers]**	**Binge drinking occasions per year**	**Drinks consumed during a binge drinking occasion**	**Per capita consumption (l/year per person)**	**Current drinkers**	**Prevalence of binge drinkers [among current drinkers]**	**Binge drinking occasions per year**	**Drinks consumed during a binge drinking occasion**	**Per capita consumption (l/year per person)**
Asia, Pacific [High Income]	87.43%	17.17%	26	7	15.23	75.62%	3.93%	26	6	4.63
Asia, Central	63.87%	52.76%	52	7	10.62	46.83%	8.78%	52	6	3.23
Asia, East	71.71%	13.48%	26	7	9.88	37.50%	0.54%	26	6	1.95
Asia, South	16.68%	45.09%	52	7	3.80	2.64%	9.22%	52	6	0.24
Asia, Southeast	27.21%	10.58%	52	7	5.21	5.63%	2.89%	52	6	0.47
Australasia	87.08%	10.00%	26	7	14.29	80.02%	2.84%	26	6	5.78
Caribbean	65.17%	20.17%	26	7	9.36	34.23%	5.11%	26	6	2.74
Europe, Central	77.41%	25.21%	52	7	21.81	59.05%	3.26%	52	6	6.70
Europe, Eastern	71.74%	59.39%	78	9	25.19	50.77%	13.25%	78	7	8.07
Europe, Western	87.80%	12.04%	26	7	17.64	77.56%	1.80%	26	6	7.06
Latin America, Andean	67.92%	18.47%	52	7	11.35	47.14%	3.84%	52	6	3.43
Latin America, Central	57.83%	22.54%	78	8	11.73	34.88%	1.56%	78	6	3.23
Latin America, Southern	86.48%	16.80%	26	7	13.91	66.75%	0.17%	26	6	5.28
Latin America, Tropical	58.67%	21.15%	52	7	14.11	41.48%	5.36%	52	6	4.39
Northern Africa / Middle East	8.90%	7.21%	26	7	2.04	2.40%	4.10%	26	6	0.26
North America [High Income]	72.70%	13.74%	26	7	14.38	60.98%	3.39%	26	6	5.05
Oceania	79.59%	25.20%	52	7	5.55	47.59%	10.74%	52	6	0.94
Sub-Saharan Africa, Central	49.95%	32.01%	52	7	5.83	29.88%	16.58%	52	6	2.18
Sub-Saharan Africa, East	29.83%	20.98%	52	7	7.37	19.34%	1.00%	52	6	2.19
Sub-Saharan Africa, Southern	37.53%	39.00%	78	8	14.28	13.60%	21.29%	78	6	3.07
Sub-Saharan Africa, Western	41.39%	32.40%	52	7	11.69	24.76%	20.88%	52	6	3.94
World	50.22%	24.24%	-	-	9.74	30.82%	5.48%	-	-	2.6

**Figure 1:**
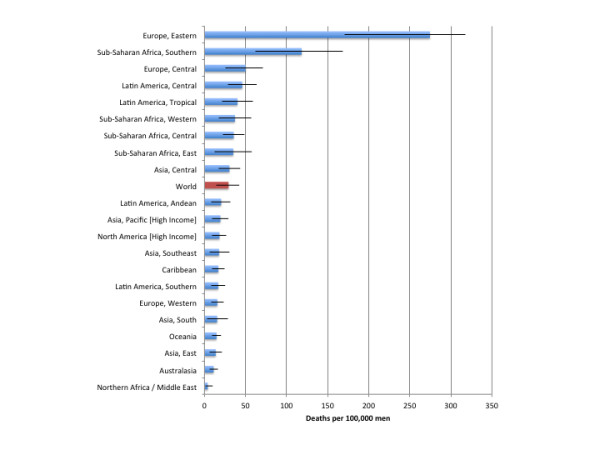
Population-standardized alcohol-attributable deaths per 100,000 people by GBD region for men.

**Figure 2:**
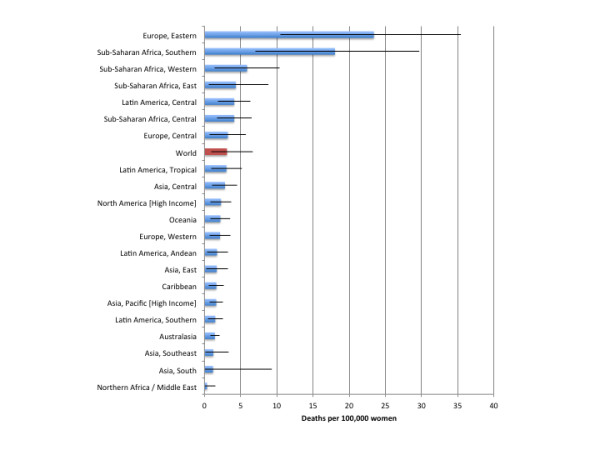
Population-standardized alcohol-attributable deaths per 100,000 people by GBD region for women.

Table 3 outlines the deaths attributable to alcohol consumption by region and sex. 851,900 (95% CI: 419,400 to 1,282,500) deaths were due to alcohol-attributable injuries, of which 221,100 (95% CI: 140,000 to 312,000) deaths were caused by alcohol-attributable harms to others (alcohol-attributable injuries caused by others are outlined in Additional file 1). Figures 1 and 2 outline the burden of injuries in deaths per 100,000 people for men and women, respectively. Alcohol-attributable injuries account for 1.43% (95% CI: 0.70% to 2.15%) of all deaths and 15.10% (95% CI: 7.43% to 22.73%) of all deaths from injuries. Of this total, 761,300 (95% CI: 390,800 to 1,088,900) deaths were among men, representing 2.42% (95% CI: 1.24% to 3.46%) of all deaths and 20.26% (95% CI: 10.40% to 28.98%) of all deaths from injuries for men. 90,600 (95% CI: 28,500 to 193,600) deaths were among women, representing 0.32% (95% CI: 0.10% to 0.69%) of all deaths and 4.81% (95% CI: 1.51% to 10.27%) of all deaths from injuries for women. Adjusting the figures in Table 3 for the population of each region, we observed that Eastern Europe had the largest population-standardized mortality rate, with 135.4 (95% CI: 82.0 to 161.4) deaths per 100,000 people caused by injuries attributable to alcohol consumption, and North Africa/Middle East had the lowest death rate, with 2.0 (95% CI: 0.5 to 5.5) deaths per 100,000 people caused by injuries attributable to alcohol consumption. All AAFs for injuries as well as alcohol-attributable injury deaths by age, sex, region, and cause are provided in Additional file 2, Additional file 3, Additional file 4 and Additional file 5.

**Table 3:** Alcohol-attributable deaths caused by injuries by Global Burden of Disease region for 2004

	**Men**			**Women**			**Total**		
	**Point estimate**	**Lower 95% confidence interval**	**Upper 95% confidence interval**	**Point estimate**	**Lower 95% confidence interval**	**Upper 95% confidence interval**	**Point estimate**	**Lower 95% confidence interval**	**Upper 95% confidence interval**
Asia, Pacific [High Income]	17,030	8,340	25,730	1,530	620	2,440	18,560	8,960	28,170
Asia, Central	8,640	4,940	12,340	1,010	410	1,620	9,650	5,340	13,960
Asia, East	80,300	37,750	122,860	10,310	1,760	19,810	90,620	39,510	142,670
Asia, South	77,810	21,330	144,490	7,070	290	49,630	84,890	21,620	194,120
Asia, Southeast	37,260	13,000	62,920	3,280	320	8,990	40,540	13,320	71,910
Australasia	1,210	630	1,800	160	90	230	1,380	720	2,030
Caribbean	2,410	1,350	3,460	300	120	490	2,710	1,470	3,950
Europe, Central	27,480	13,820	39,030	2,120	480	3,760	29,600	14,290	42,790
Europe, Eastern	258,530	161,260	298,650	29,310	12,980	44,570	287,840	174,240	343,220
Europe, Western	30,270	14,990	45,550	4,490	1,360	7,620	34,760	16,340	53,170
Latin America, Andean	3,790	1,660	5,930	370	100	690	4,160	1,760	6,620
Latin America, Central	38,050	23,870	52,240	4,010	1,950	6,130	42,060	25,820	58,370
Latin America, Southern	3,880	1,920	5,840	390	110	670	4,270	2,040	6,510
Latin America, Tropical	33,810	18,020	49,600	3,060	1,070	5,110	36,870	19,090	54,710
Northern Africa / Middle East	7,530	1,710	19,660	670	0	2,840	8,200	1,710	22,490
North America [High Income]	25,840	13,290	38,400	3,850	1,450	6,250	29,690	14,740	44,650
Oceania	430	270	580	70	30	120	500	300	700
Sub-Saharan Africa, Central	9,500	6,180	12,810	1,410	600	2,220	10,910	6,780	15,030
Sub-Saharan Africa, East	31,430	12,860	50,150	5,080	850	10,000	36,510	13,710	60,150
Sub-Saharan Africa, Southern	32,110	17,060	45,430	5,570	2,290	9,030	37,680	19,350	54,460
Sub-Saharan Africa, Western	33,990	16,560	51,420	6,510	1,680	11,380	40,500	18,240	62,800
World	761,300	390,800	1,088,900	90,600	28,500	193,600	851,900	419,400	1,282,500

**Figure 3:**
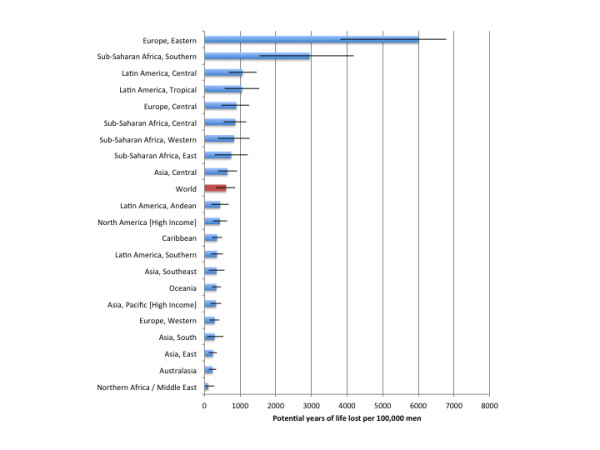
Population-standardized alcohol-attributable potential years of life lost per 100,000 people by GBD region for men.

**Figure 4:**
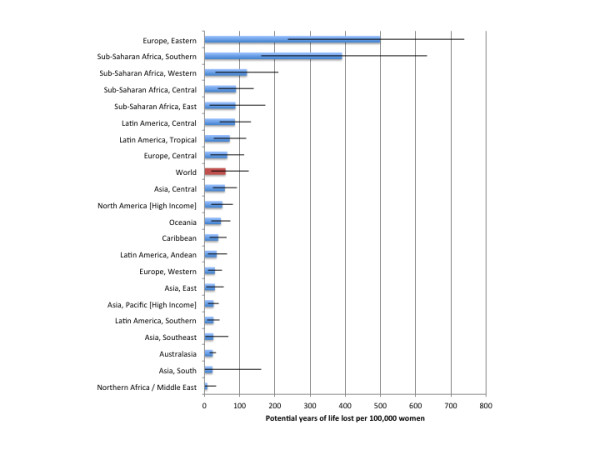
Population-standardized alcohol-attributable potential years of life lost per 100,000 people by GBD region for women.

Globally, in 2004, 19,051,000 (95% CI: 9,767,000 to 28,243,000) PYLL or 320.1 (95% CI: 161.1 to 477.9) PYLL per 100,000 people were caused by injuries attributable to alcohol consumption, of which 5,989,300 (95% CI: 3,788,700 to 8,423,600) were caused by harms to others. Alcohol-attributable injuries accounted for 2.02% (95% CI: 1.03% to 2.99%) PYLL and 14.54% (95% CI: 7.45% to 21.55%) of all PYLL caused by injuries worldwide. Table 4 outlines estimates of the PYLL attributable to alcohol consumption by region and sex for 2004. Figures 3 and 4 outline the burden of injuries in PYLL per 100,000 people for men and women, respectively. Alcohol-attributable injury PYLL among men were far in excess of the estimates calculated for women, with 605.8 (95% CI: 317.9 to 860.0) PYLL per 100,000 men (3.29% [95% CI: 1.76% to 4.65%] of all PYLL and 19.11% [95% CI: 10.22% to 26.95%] of all PYLL caused by injuries for men) compared to 59.9 (95% CI: 19.4 to 126.7) PYLL per 100,000 women (0.48% [95% CI: 0.16% to 1.00%] of all PYLL and 4.92% [95% CI: 1.62% to 10.20%] of all PYLL caused by injuries for women). Eastern Europe had the highest burden in terms of PYLL for men and women, with 6,003.8 (95% CI: 3813.4 to 6785.6) PYLL per 100,000 men and 499.1 (95% CI: 236.1 to738.2) PYLL per 100,000 women. North Africa/Middle East had the lowest burden of PYLL, with 107.3 (95% CI: 22.9 to 282.9) PYLL per 100,000 men and 7.8 (95% CI: 0.0 to 160) PYLL per 100,000 women.

**Table 4:** Alcohol-attributable PYLL caused by injuries by Global Burden of Disease region for 2004

	**Men**			**Women**			**Total**		
	**Point estimate**	**Lower 95% confidence interval**	**Upper 95% confidence interval**	**Point estimate**	**Lower 95% confidence interval**	**Upper 95% confidence interval**	**Point estimate**	**Lower 95% confidence interval**	**Upper 95% confidence interval**
Asia, Pacific [High Income]	274,300	138,300	410,300	24,800	10,100	39,500	299,100	148,400	449,700
Asia, Central	200,500	116,000	284,900	23,900	9,800	37,900	224,300	125,700	322,900
Asia, East	1,511,900	775,000	2,248,800	212,600	42,000	400,600	1,724,500	817,000	2,649,400
Asia, South	1,670,100	520,100	3,086,300	167,100	7,300	1,049,400	1,837,200	527,400	4,135,800
Asia, Southeast	799,800	310,200	1,316,400	78,100	7,900	208,300	878,000	318,100	1,524,600
Australasia	24,400	13,200	35,500	3,100	1,800	4,300	27,400	15,000	39,800
Caribbean	55,100	32,400	77,800	8,100	3,100	13,200	63,300	35,500	91,000
Europe, Central	494,400	258,900	693,600	43,800	11,000	76,600	538,300	269,900	770,200
Europe, Eastern	5,699,200	3,622,900	6,438,200	632,500	296,200	939,000	6,331,700	3,919,100	7,377,200
Europe, Western	520,300	274,000	766,500	64,400	19,400	109,500	584,700	293,400	875,900
Latin America, Andean	90,000	40,600	139,400	9,500	2,600	17,700	99,500	43,200	157,100
Latin America, Central	984,400	624,100	1,344,600	106,500	52,800	162,500	1,090,900	676,900	1,507,100
Latin America, Southern	87,300	45,000	129,700	8,000	2,700	13,500	95,300	47,700	143,200
Latin America, Tropical	926,900	504,500	1,349,400	82,000	30,700	134,600	1,008,900	535,200	1,484,000
Northern Africa / Middle East	219,500	47,000	587,600	18,800	0	76,900	238,300	47,000	664,500
North America [High Income]	610,100	324,400	895,800	89,600	34,500	144,700	699,700	358,900	1,040,500
Oceania	11,400	7,400	15,400	2,000	800	3,200	13,400	8,200	18,600
Sub-Saharan Africa, Central	259,500	171,000	348,100	40,400	17,300	63,600	299,900	188,200	411,600
Sub-Saharan Africa, East	785,900	344,300	1,230,700	138,700	24,800	268,400	924,700	369,100	1,499,100
Sub-Saharan Africa, Southern	882,400	473,800	1,245,100	146,600	61,600	236,100	1,029,100	535,400	1,481,200
Sub-Saharan Africa, Western	865,800	439,200	1,292,300	176,900	48,300	306,800	1,042,700	487,500	1,599,100
World	16,973,000	9,082,000	23,936,000	2,078,000	685,000	4,306,000	19,051,000	9,767,000	28,243,000

**Figure 5:**
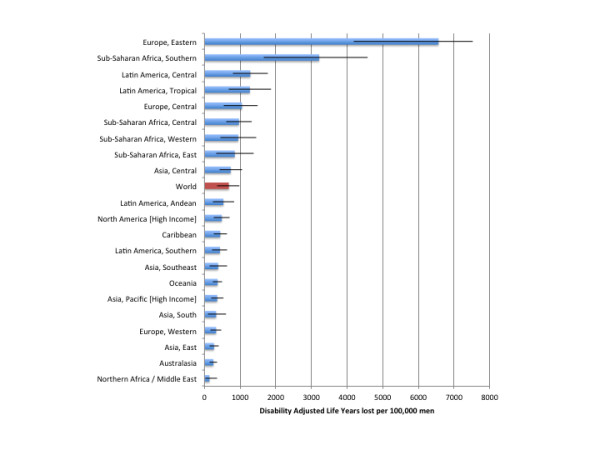
Population-standardized alcohol-attributable disability-adjusted years of life lost per 100,000 people by GBD region for men.

**Figure 6:**
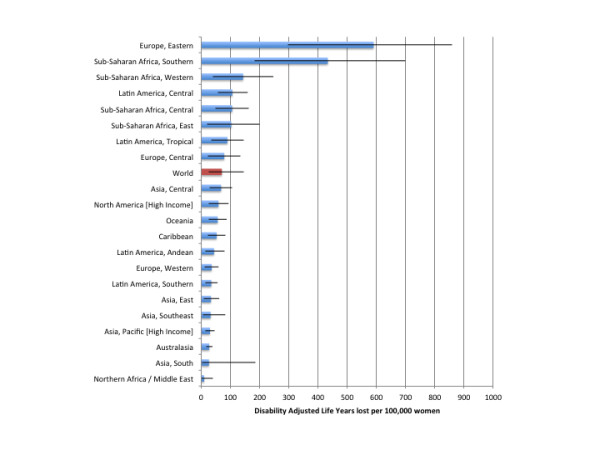
Population-standardized alcohol-attributable disability-adjusted years of life lost per 100,000 people by GBD region for women.

In 2004, alcohol-attributable injuries accounted for 21,668,000 (95% CI: 11,097,000 to 32,385,000) DALYs, of which 6,917,300 (95% CI: 4,396,000 to 9,731,100) were caused by alcohol-attributable injuries caused by others. Alcohol-attributable injuries in 2004 accounted for 1.40% (95% CI: 0.71% to 2.09%) of all DALYs and 11.35% (95% CI: 5.81% to 16.96%) of DALYs caused by injuries among men and women. Table 5 outlines estimates of the DALYs attributable to alcohol consumption by region and by sex for 2004. Figures 5 and 6 outline the burden of injuries in DALYs per 100,000 people for men and women, respectively. Alcohol accounted for 681.8 (95% CI: 354.8 to977.9) DALYs per 100,000 men and 70.4 (95% CI: 25.3 to 146.4) DALYs per 100,000 women. Eastern Europe had the highest DALYs per 100,000 people, with 6,561.7 (95% CI: 4191.3 to 7523.2) DALYs per 100,000 men and 590.2 (95% CI: 296.3 to 860.0) DALYs per 100,000 women. The North Africa/Middle East region was estimated to have the lowest alcohol-attributable injury DALYs worldwide, with 138.5 (95% CI: 22.2 to 364.1) DALYs per 100,000 men and 9.9 (95% CI: 1.1 to 40.6) DALYs per 100,000 women. An outline of the alcohol-attributable years of life lived with disability is provided in Additional file 6.

**Table 5:** Alcohol-attributable DALYs caused by injuries by Global Burden of Disease region for 2004

	**Men**			**Women**			**Total**		
	**Point estimate**	**Lower 95% confidence interval**	**Upper 95% confidence interval**	**Point estimate**	**Lower 95% confidence interval**	**Upper 95% confidence interval**	**Point estimate**	**Lower 95% confidence interval**	**Upper 95% confidence interval**
Asia, Pacific [High Income]	301,300	152,000	450,600	28,900	12,500	45,300	330,200	164,500	495,900
Asia, Central	230,300	131,200	329,300	28,400	12,600	44,100	258,600	143,900	373,400
Asia, East	1,690,700	858,200	2,523,100	240,200	56,100	443,700	1,930,900	914,300	2,966,800
Asia, South	1,918,200	586,800	3,558,600	198,700	26,800	1,215,700	2,117,000	613,600	4,774,200
Asia, Southeast	902,500	347,700	1,489,100	102,700	23,300	255,600	1,005,200	371,000	1,744,700
Australasia	26,900	14,500	39,200	3,600	2,100	5,100	30,500	16,600	44,300
Caribbean	69,300	40,300	98,300	11,200	4,800	17,600	80,500	45,100	115,900
Europe, Central	578,300	300,000	820,200	53,400	14,900	92,000	631,700	314,900	912,100
Europe, Eastern	6,227,900	3,981,100	7,137,100	745,100	369,700	1,090,500	6,972,900	4,350,800	8,227,600
Europe, Western	587,200	307,300	867,200	77,700	25,000	130,300	664,900	332,300	997,500
Latin America, Andean	109,000	48,300	169,600	12,200	4,000	22,200	121,200	52,400	191,800
Latin America, Central	1,193,900	743,100	1,644,600	133,300	71,500	198,300	1,327,200	814,600	1,842,900
Latin America, Southern	108,000	56,400	159,600	10,900	4,400	17,600	118,800	60,800	177,100
Latin America, Tropical	1,128,000	606,700	1,649,300	103,400	42,700	166,100	1,231,400	649,400	1,815,400
Northern Africa / Middle East	284,800	52,200	758,300	24,400	3,100	94,700	309,200	55,300	853,000
North America [High Income]	678,700	359,500	997,800	104,000	42,900	165,100	782,700	402,500	1,162,900
Oceania	12,500	8,000	17,000	2,500	1,200	3,800	15,000	9,200	20,800
Sub-Saharan Africa, Central	292,700	191,400	394,000	48,400	22,900	73,800	341,100	214,400	467,800
Sub-Saharan Africa, East	895,900	386,700	1,408,500	163,400	37,700	307,800	1,059,300	424,400	1,716,300
Sub-Saharan Africa, Southern	966,400	511,300	1,367,100	165,100	72,300	263,800	1,131,600	583,600	1,630,800
Sub-Saharan Africa, Western	997,300	499,500	1,495,200	210,600	64,500	358,000	1,207,900	563,900	1,853,100
World	19,200,000	10,182,000	27,374,000	2,468,000	915,000	5,011,000	21,668,000	11,097,000	32,385,000

## **Discussion**

Alcohol is a substantial risk factor for the global burden of injuries in terms of death, PYLL, and DALYs. We found that this burden of injuries for 2004 varied by region corresponding with drinking prevalence: those regions exhibiting a low prevalence of current drinkers, such as in North Africa/Middle East and Southern Asia, carried a relatively low burden of injuries attributable to alcohol. This can be contrasted with Eastern Europe, where drinking prevalence, binge drinking, and the burden due to alcohol-attributable injury were all high.

Before we discuss the implications of these findings, the potential weaknesses associated with this analysis should be discussed in detail. First, quantification of the global burden using insurance and police records, which would provide the most accurate data, appears not to be possible as no such reporting system exists [29]. Second, there are limitations regarding the quality of global mortality data (see [27]). For most of the world, there are no vital registries, i.e., there is scarce or no available information on causes of death. In these instances, data on the missing causes of death have to be statistically estimated [28,30,31]. Although we do not incorporate the variation of the mortality estimates in these countries into our analysis, estimations by the WHO/GBD of the number of deaths in countries where little or no data are available increase the uncertainty of our estimates of the number of deaths, PYLL, and DALYs attributable to alcohol consumption [27]. Third, there are weaknesses associated with the assumptions made in the calculation of the DALYs (see [32]), particularly the calculation of weights for DALYs, although these assumptions have been shown to exert only a minor effect on the variation of DALY estimates for injuries [27]. Fourth, alcohol consumption variables used in our analysis came from population surveys which have limitations with respect to coverage, and the survey instruments involved commonly have inherent biases due to self-reporting of data, leading to an underestimation of level of drinking and number of binge drinkers [33]. While the distribution of average volume of alcohol consumption can be adjusted for the amount of alcohol actually consumed [34], there is currently no methodology to correct the underestimation of irregular binge drinking [35], thereby causing an underestimation of the global burden of injuries attributable to alcohol consumption.

Our study is further limited by the use of the RR for all nonmotor vehicle accident injuries. We suspect that the risk relationship may change by injury type, but the body of research relating alcohol consumption to injury is relatively sparse (except with respect to motor vehicle accidents), meaning that meta-analytic techniques used to generate stable risk curves are not usable due to a scarcity of data points. This is especially important for alcohol consumption and resulting intentional and unintentional nonmotor vehicle accident injuries, due to alcohol playing a very different role in intentional and unintentional nonmotor vehicle accident injuries [6]. However, the risk estimates for intentional and unintentional injuries were not stable in the meta-analyses performed by Taylor and colleagues and, thus, were combined [6]. The resulting aggregate RR showed little heterogeneity among studies that examined intentional and unintentional nonmotor vehicle accident injuries [6]. Additionally, although not taken into consideration in our analysis, previous research has suggested that the RRs for injury may be dependent upon previous alcohol consumption patterns, with heavy consumers of alcohol at a lower risk than those people who do not frequently consume large amounts of alcohol [36-38].

Our study is limited also by the information on harms to others available in the literature; we used data from Australia to model harms to others. Because of limited data, we were unable to determine if a linear relationship exists between the AAFs for motor vehicle accident injuries to passengers, pedestrians, and drivers who did not cause the accident and the AAFs for motor vehicle accident deaths to drivers who caused the accident. We were also unable to quantify the relationship between age-specific AAFs for assaults and population AAFs for assaults. This lack of data leads to two different formulas being used for AAFs for motor vehicle accidents caused to others and for AAFs for assaults; the AAFs for assaults were calculated assuming a linear relationship, and the AAFs for motor vehicle accidents caused to others had to be log transformed. Log transformation of the AAFs for motor vehicle accidents caused to others was required in order to keep the resulting total AAFs within the boundaries of 0 and 1. In the case of the AAFs for assaults, log transformation was not necessary since the age-specific AAFs were very similar.

Regardless of these limitations, the method of calculating the burden of injury attributable to alcohol consumption presented in this article is an improvement on previous methods used to calculate the alcohol-attributable burden. For the first time at a global level the burden of alcohol-attributable injuries has been estimated using consumption data and RRs, and alcohol-related harms to others have been calculated.

## **Conclusion**

Given the severity of the alcohol-attributable burden of injuries and the expectation that it will increase in developing countries [39,40], it is imperative to accurately characterize this burden and develop strategies aimed at reducing it. This article presents a new method to calculate the burden of disease attributable to alcohol consumption and is an improvement over previous methods. Additionally, given the size of the estimated alcohol-attributable burden of injuries, strategies aimed at reducing this burden should target two key areas of concern: 1) the need to decrease the harmful consumption of alcohol, by methods such as regulating the availability of alcohol [41], and 2) the need to decrease the frequency of drunk driving by, for example, lowering the maximum allowable blood alcohol concentration level for driving, especially in the case of younger drivers [36].

## **Additional files**

## **Competing interests**

The authors have declared that no competing interests exist.

## **Authors’ contributions**

Kevin Shield and Jürgen Rehm conceptualized the overall article. Kevin Shield, Gerrit Gmel, Jayadeep Patra, and Jürgen Rehm contributed to the methodology, identified sources for risk relations and exposure, and contributed to the writing. Kevin Shield performed all statistical analyses. All authors have approved the final version.

## Supplementary Material

Additional file 1 Alcohol-attributable injury caused by harms to others by global burden of disease region. Click here for file

Additional file 2 Alcohol-Attributable Fractions for injuries. Click here for file

Additional file 3 Alcohol-Attributable Fractions for injuries. Click here for file

Additional file 4 Deaths from injuries attributable to alcohol consumption. Click here for file

Additional file 5 Deaths from injuries attributable to alcohol consumption. Click here for file

Additional file 6 Population-standardized alcohol-attributable years of life lived with disability per 100,000 people by GBD region for men and women. Click here for file
