# Supplementary material for: Global burden of injuries attributable to alcohol consumption in 2004: a novel way of calculating the burden of injuries attributable to alcohol consumption
Source: Popul Health Metr. 2012 May 18;10:9. doi: 10.1186/1478-7954-10-9 (PMC3463441; doi:10.1186/1478-7954-10-9)
Supplement: Additional file 1 — Alcohol-attributable injury caused by harms to others by global burden of disease region. [file 1478-7954-10-9-S1.docx]

Appendix 1: Alcohol-Attributable Fractions for injuries (without harms to others included)

Table 1: Morbidity Alcohol-Attributable Fractions for injuries (without harms to others included): Asia Pacific [High Income]

|  |  |  | 0 to 14 years of age | | |  | 15 to 34 years of age | | |  | 35 to 64 years of age | | |  | 65 years of age and older | | |
| --- | --- | --- | --- | --- | --- | --- | --- | --- | --- | --- | --- | --- | --- | --- | --- | --- | --- |
|  |  |  | Point estimate | Lower 95% CI | Upper 95% CI |  | Point estimate | Lower 95% CI | Upper 95% CI |  | Point estimate | Lower 95% CI | Upper 95% CI |  | Point estimate | Lower 95% CI | Upper 95% CI |
| Women | |  |  |  |  |  |  |  |  |  |  |  |  |  |  |  |  |
| Injuries | |  |  |  |  |  |  |  |  |  |  |  |  |  |  |  |  |
|  | Unintentional injuries | |  |  |  |  |  |  |  |  |  |  |  |  |  |  |  |
|  |  | Transport injuries | 0.00% | 0.00% | 0.00% |  | 1.26% | 0.77% | 1.76% |  | 3.17% | 1.65% | 4.69% |  | 0.83% | 0.49% | 1.18% |
|  |  | Poisonings | 0.00% | 0.00% | 0.00% |  | 1.82% | 0.79% | 2.85% |  | 2.83% | 1.09% | 4.57% |  | 0.83% | 0.36% | 1.30% |
|  |  | Falls | 0.00% | 0.00% | 0.00% |  | 1.82% | 0.79% | 2.85% |  | 2.83% | 1.09% | 4.57% |  | 0.83% | 0.36% | 1.30% |
|  |  | Fires, heat and hot substances | 0.00% | 0.00% | 0.00% |  | 1.82% | 0.79% | 2.85% |  | 2.83% | 1.09% | 4.57% |  | 0.83% | 0.36% | 1.30% |
|  |  | Drownings | 0.00% | 0.00% | 0.00% |  | 1.82% | 0.79% | 2.85% |  | 2.83% | 1.09% | 4.57% |  | 0.83% | 0.36% | 1.30% |
|  |  | Other unintentional injuries | 0.00% | 0.00% | 0.00% |  | 1.82% | 0.79% | 2.85% |  | 2.83% | 1.09% | 4.57% |  | 0.83% | 0.36% | 1.30% |
|  | Intentional injuries | |  |  |  |  |  |  |  |  |  |  |  |  |  |  |  |
|  |  | Self-inflicted injuries | 0.00% | 0.00% | 0.00% |  | 1.82% | 0.79% | 2.85% |  | 2.83% | 1.09% | 4.57% |  | 0.83% | 0.36% | 1.30% |
|  |  | Violence | 0.00% | 0.00% | 0.00% |  | 0.00% | 0.00% | 0.00% |  | 0.00% | 0.00% | 0.00% |  | 0.00% | 0.00% | 0.00% |
|  |  | Other intentional injuries | 0.00% | 0.00% | 0.00% |  | 1.82% | 0.79% | 2.85% |  | 2.83% | 1.09% | 4.57% |  | 0.83% | 0.36% | 1.30% |
| Men | |  |  |  |  |  |  |  |  |  |  |  |  |  |  |  |  |
| Injuries | |  |  |  |  |  |  |  |  |  |  |  |  |  |  |  |  |
|  | Unintentional injuries | |  |  |  |  |  |  |  |  |  |  |  |  |  |  |  |
|  |  | Transport injuries | 0.00% | 0.00% | 0.00% |  | 4.15% | 2.52% | 5.78% |  | 10.42% | 5.42% | 15.43% |  | 2.74% | 1.62% | 3.87% |
|  |  | Poisonings | 0.00% | 0.00% | 0.00% |  | 7.33% | 3.07% | 11.59% |  | 16.65% | 7.93% | 25.37% |  | 5.58% | 2.24% | 8.91% |
|  |  | Falls | 0.00% | 0.00% | 0.00% |  | 7.33% | 3.07% | 11.59% |  | 16.65% | 7.93% | 25.37% |  | 5.58% | 2.24% | 8.91% |
|  |  | Fires, heat and hot substances | 0.00% | 0.00% | 0.00% |  | 7.33% | 3.07% | 11.59% |  | 16.65% | 7.93% | 25.37% |  | 5.58% | 2.24% | 8.91% |
|  |  | Drownings | 0.00% | 0.00% | 0.00% |  | 7.33% | 3.07% | 11.59% |  | 16.65% | 7.93% | 25.37% |  | 5.58% | 2.24% | 8.91% |
|  |  | Other unintentional injuries | 0.00% | 0.00% | 0.00% |  | 7.33% | 3.07% | 11.59% |  | 16.65% | 7.93% | 25.37% |  | 5.58% | 2.24% | 8.91% |
|  | Intentional injuries | |  |  |  |  |  |  |  |  |  |  |  |  |  |  |  |
|  |  | Self-inflicted injuries | 0.00% | 0.00% | 0.00% |  | 7.33% | 3.07% | 11.59% |  | 16.65% | 7.93% | 25.37% |  | 5.58% | 2.24% | 8.91% |
|  |  | Violence | 0.00% | 0.00% | 0.00% |  | 0.00% | 0.00% | 0.00% |  | 0.00% | 0.00% | 0.00% |  | 0.00% | 0.00% | 0.00% |
|  |  | Other intentional injuries | 0.00% | 0.00% | 0.00% |  | 7.33% | 3.07% | 11.59% |  | 16.65% | 7.93% | 25.37% |  | 5.58% | 2.24% | 8.91% |

Table 2: Morbidity Alcohol-Attributable Fractions for injuries (without harms to others included): Asia Central

|  |  |  | 0 to 14 years of age | | |  | 15 to 34 years of age | | |  | 35 to 64 years of age | | |  | 65 years of age and older | | |
| --- | --- | --- | --- | --- | --- | --- | --- | --- | --- | --- | --- | --- | --- | --- | --- | --- | --- |
|  |  |  | Point estimate | Lower 95% CI | Upper 95% CI |  | Point estimate | Lower 95% CI | Upper 95% CI |  | Point estimate | Lower 95% CI | Upper 95% CI |  | Point estimate | Lower 95% CI | Upper 95% CI |
| Women | |  |  |  |  |  |  |  |  |  |  |  |  |  |  |  |  |
| Injuries | |  |  |  |  |  |  |  |  |  |  |  |  |  |  |  |  |
|  | Unintentional injuries | |  |  |  |  |  |  |  |  |  |  |  |  |  |  |  |
|  |  | Transport injuries | 0.00% | 0.00% | 0.00% |  | 4.05% | 2.66% | 5.44% |  | 6.01% | 4.13% | 7.90% |  | 3.07% | 1.96% | 4.17% |
|  |  | Poisonings | 0.00% | 0.00% | 0.00% |  | 1.40% | 0.16% | 2.63% |  | 3.37% | 0.48% | 6.26% |  | 0.39% | 0.00% | 0.80% |
|  |  | Falls | 0.00% | 0.00% | 0.00% |  | 1.40% | 0.16% | 2.63% |  | 3.37% | 0.48% | 6.26% |  | 0.39% | 0.00% | 0.80% |
|  |  | Fires, heat and hot substances | 0.00% | 0.00% | 0.00% |  | 1.40% | 0.16% | 2.63% |  | 3.37% | 0.48% | 6.26% |  | 0.39% | 0.00% | 0.80% |
|  |  | Drownings | 0.00% | 0.00% | 0.00% |  | 1.40% | 0.16% | 2.63% |  | 3.37% | 0.48% | 6.26% |  | 0.39% | 0.00% | 0.80% |
|  |  | Other unintentional injuries | 0.00% | 0.00% | 0.00% |  | 1.40% | 0.16% | 2.63% |  | 3.37% | 0.48% | 6.26% |  | 0.39% | 0.00% | 0.80% |
|  | Intentional injuries | |  |  |  |  |  |  |  |  |  |  |  |  |  |  |  |
|  |  | Self-inflicted injuries | 0.00% | 0.00% | 0.00% |  | 1.40% | 0.16% | 2.63% |  | 3.37% | 0.48% | 6.26% |  | 0.39% | 0.00% | 0.80% |
|  |  | Violence | 0.00% | 0.00% | 0.00% |  | 0.00% | 0.00% | 0.00% |  | 0.00% | 0.00% | 0.00% |  | 0.00% | 0.00% | 0.00% |
|  |  | Other intentional injuries | 0.00% | 0.00% | 0.00% |  | 1.40% | 0.16% | 2.63% |  | 3.37% | 0.48% | 6.26% |  | 0.39% | 0.00% | 0.80% |
| Men | |  |  |  |  |  |  |  |  |  |  |  |  |  |  |  |  |
| Injuries | |  |  |  |  |  |  |  |  |  |  |  |  |  |  |  |  |
|  | Unintentional injuries | |  |  |  |  |  |  |  |  |  |  |  |  |  |  |  |
|  |  | Transport injuries | 0.00% | 0.00% | 0.00% |  | 13.31% | 8.75% | 17.86% |  | 19.75% | 13.56% | 25.94% |  | 10.07% | 6.44% | 13.70% |
|  |  | Poisonings | 0.00% | 0.00% | 0.00% |  | 9.73% | 3.90% | 15.56% |  | 11.78% | 5.46% | 18.09% |  | 5.71% | 2.48% | 8.95% |
|  |  | Falls | 0.00% | 0.00% | 0.00% |  | 9.73% | 3.90% | 15.56% |  | 11.78% | 5.46% | 18.09% |  | 5.71% | 2.48% | 8.95% |
|  |  | Fires, heat and hot substances | 0.00% | 0.00% | 0.00% |  | 9.73% | 3.90% | 15.56% |  | 11.78% | 5.46% | 18.09% |  | 5.71% | 2.48% | 8.95% |
|  |  | Drownings | 0.00% | 0.00% | 0.00% |  | 9.73% | 3.90% | 15.56% |  | 11.78% | 5.46% | 18.09% |  | 5.71% | 2.48% | 8.95% |
|  |  | Other unintentional injuries | 0.00% | 0.00% | 0.00% |  | 9.73% | 3.90% | 15.56% |  | 11.78% | 5.46% | 18.09% |  | 5.71% | 2.48% | 8.95% |
|  | Intentional injuries | |  |  |  |  |  |  |  |  |  |  |  |  |  |  |  |
|  |  | Self-inflicted injuries | 0.00% | 0.00% | 0.00% |  | 9.73% | 3.90% | 15.56% |  | 11.78% | 5.46% | 18.09% |  | 5.71% | 2.48% | 8.95% |
|  |  | Violence | 0.00% | 0.00% | 0.00% |  | 0.00% | 0.00% | 0.00% |  | 0.00% | 0.00% | 0.00% |  | 0.00% | 0.00% | 0.00% |
|  |  | Other intentional injuries | 0.00% | 0.00% | 0.00% |  | 9.73% | 3.90% | 15.56% |  | 11.78% | 5.46% | 18.09% |  | 5.71% | 2.48% | 8.95% |

Table 3: Morbidity Alcohol-Attributable Fractions for injuries (without harms to others included): Asia East

|  |  |  | 0 to 14 years of age | | |  | 15 to 34 years of age | | |  | 35 to 64 years of age | | |  | 65 years of age and older | | |
| --- | --- | --- | --- | --- | --- | --- | --- | --- | --- | --- | --- | --- | --- | --- | --- | --- | --- |
|  |  |  | Point estimate | Lower 95% CI | Upper 95% CI |  | Point estimate | Lower 95% CI | Upper 95% CI |  | Point estimate | Lower 95% CI | Upper 95% CI |  | Point estimate | Lower 95% CI | Upper 95% CI |
| Women | |  |  |  |  |  |  |  |  |  |  |  |  |  |  |  |  |
| Injuries | |  |  |  |  |  |  |  |  |  |  |  |  |  |  |  |  |
|  | Unintentional injuries | |  |  |  |  |  |  |  |  |  |  |  |  |  |  |  |
|  |  | Transport injuries | 0.00% | 0.00% | 0.00% |  | 0.36% | 0.21% | 0.51% |  | 1.32% | 0.59% | 2.04% |  | 0.51% | 0.25% | 0.77% |
|  |  | Poisonings | 0.00% | 0.00% | 0.00% |  | 0.27% | 0.04% | 0.49% |  | 1.32% | 0.00% | 3.02% |  | 0.54% | 0.00% | 1.09% |
|  |  | Falls | 0.00% | 0.00% | 0.00% |  | 0.27% | 0.04% | 0.49% |  | 1.32% | 0.00% | 3.02% |  | 0.54% | 0.00% | 1.09% |
|  |  | Fires, heat and hot substances | 0.00% | 0.00% | 0.00% |  | 0.27% | 0.04% | 0.49% |  | 1.32% | 0.00% | 3.02% |  | 0.54% | 0.00% | 1.09% |
|  |  | Drownings | 0.00% | 0.00% | 0.00% |  | 0.27% | 0.04% | 0.49% |  | 1.32% | 0.00% | 3.02% |  | 0.54% | 0.00% | 1.09% |
|  |  | Other unintentional injuries | 0.00% | 0.00% | 0.00% |  | 0.27% | 0.04% | 0.49% |  | 1.32% | 0.00% | 3.02% |  | 0.54% | 0.00% | 1.09% |
|  | Intentional injuries | |  |  |  |  |  |  |  |  |  |  |  |  |  |  |  |
|  |  | Self-inflicted injuries | 0.00% | 0.00% | 0.00% |  | 0.27% | 0.04% | 0.49% |  | 1.32% | 0.00% | 3.02% |  | 0.54% | 0.00% | 1.09% |
|  |  | Violence | 0.00% | 0.00% | 0.00% |  | 0.00% | 0.00% | 0.00% |  | 0.00% | 0.00% | 0.00% |  | 0.00% | 0.00% | 0.00% |
|  |  | Other intentional injuries | 0.00% | 0.00% | 0.00% |  | 0.27% | 0.04% | 0.49% |  | 1.32% | 0.00% | 3.02% |  | 0.54% | 0.00% | 1.09% |
| Men | |  |  |  |  |  |  |  |  |  |  |  |  |  |  |  |  |
| Injuries | |  |  |  |  |  |  |  |  |  |  |  |  |  |  |  |  |
|  | Unintentional injuries | |  |  |  |  |  |  |  |  |  |  |  |  |  |  |  |
|  |  | Transport injuries | 0.00% | 0.00% | 0.00% |  | 1.83% | 1.05% | 2.60% |  | 6.66% | 3.00% | 10.32% |  | 2.57% | 1.25% | 3.90% |
|  |  | Poisonings | 0.00% | 0.00% | 0.00% |  | 2.28% | 1.04% | 3.52% |  | 11.66% | 4.42% | 18.90% |  | 5.92% | 1.91% | 9.92% |
|  |  | Falls | 0.00% | 0.00% | 0.00% |  | 2.28% | 1.04% | 3.52% |  | 11.66% | 4.42% | 18.90% |  | 5.92% | 1.91% | 9.92% |
|  |  | Fires, heat and hot substances | 0.00% | 0.00% | 0.00% |  | 2.28% | 1.04% | 3.52% |  | 11.66% | 4.42% | 18.90% |  | 5.92% | 1.91% | 9.92% |
|  |  | Drownings | 0.00% | 0.00% | 0.00% |  | 2.28% | 1.04% | 3.52% |  | 11.66% | 4.42% | 18.90% |  | 5.92% | 1.91% | 9.92% |
|  |  | Other unintentional injuries | 0.00% | 0.00% | 0.00% |  | 2.28% | 1.04% | 3.52% |  | 11.66% | 4.42% | 18.90% |  | 5.92% | 1.91% | 9.92% |
|  | Intentional injuries | |  |  |  |  |  |  |  |  |  |  |  |  |  |  |  |
|  |  | Self-inflicted injuries | 0.00% | 0.00% | 0.00% |  | 2.28% | 1.04% | 3.52% |  | 11.66% | 4.42% | 18.90% |  | 5.92% | 1.91% | 9.92% |
|  |  | Violence | 0.00% | 0.00% | 0.00% |  | 0.00% | 0.00% | 0.00% |  | 0.00% | 0.00% | 0.00% |  | 0.00% | 0.00% | 0.00% |
|  |  | Other intentional injuries | 0.00% | 0.00% | 0.00% |  | 2.28% | 1.04% | 3.52% |  | 11.66% | 4.42% | 18.90% |  | 5.92% | 1.91% | 9.92% |

Table 4: Morbidity Alcohol-Attributable Fractions for injuries (without harms to others included): Asia South

|  |  |  | 0 to 14 years of age | | |  | 15 to 34 years of age | | |  | 35 to 64 years of age | | |  | 65 years of age and older | | |
| --- | --- | --- | --- | --- | --- | --- | --- | --- | --- | --- | --- | --- | --- | --- | --- | --- | --- |
|  |  |  | Point estimate | Lower 95% CI | Upper 95% CI |  | Point estimate | Lower 95% CI | Upper 95% CI |  | Point estimate | Lower 95% CI | Upper 95% CI |  | Point estimate | Lower 95% CI | Upper 95% CI |
| Women | |  |  |  |  |  |  |  |  |  |  |  |  |  |  |  |  |
| Injuries | |  |  |  |  |  |  |  |  |  |  |  |  |  |  |  |  |
|  | Unintentional injuries | |  |  |  |  |  |  |  |  |  |  |  |  |  |  |  |
|  |  | Transport injuries | 0.00% | 0.00% | 0.00% |  | 0.19% | 0.09% | 0.29% |  | 0.50% | 0.13% | 0.88% |  | 0.17% | 0.00% | 0.35% |
|  |  | Poisonings | 0.00% | 0.00% | 0.00% |  | 0.05% | 0.00% | 1.16% |  | 0.47% | 0.00% | 8.75% |  | 0.01% | 0.00% | 1.24% |
|  |  | Falls | 0.00% | 0.00% | 0.00% |  | 0.05% | 0.00% | 1.16% |  | 0.47% | 0.00% | 8.75% |  | 0.01% | 0.00% | 1.24% |
|  |  | Fires, heat and hot substances | 0.00% | 0.00% | 0.00% |  | 0.05% | 0.00% | 1.16% |  | 0.47% | 0.00% | 8.75% |  | 0.01% | 0.00% | 1.24% |
|  |  | Drownings | 0.00% | 0.00% | 0.00% |  | 0.05% | 0.00% | 1.16% |  | 0.47% | 0.00% | 8.75% |  | 0.01% | 0.00% | 1.24% |
|  |  | Other unintentional injuries | 0.00% | 0.00% | 0.00% |  | 0.05% | 0.00% | 1.16% |  | 0.47% | 0.00% | 8.75% |  | 0.01% | 0.00% | 1.24% |
|  | Intentional injuries | |  |  |  |  |  |  |  |  |  |  |  |  |  |  |  |
|  |  | Self-inflicted injuries | 0.00% | 0.00% | 0.00% |  | 0.05% | 0.00% | 1.16% |  | 0.47% | 0.00% | 8.75% |  | 0.01% | 0.00% | 1.24% |
|  |  | Violence | 0.00% | 0.00% | 0.00% |  | 0.00% | 0.00% | 0.00% |  | 0.00% | 0.00% | 0.00% |  | 0.00% | 0.00% | 0.00% |
|  |  | Other intentional injuries | 0.00% | 0.00% | 0.00% |  | 0.05% | 0.00% | 1.16% |  | 0.47% | 0.00% | 8.75% |  | 0.01% | 0.00% | 1.24% |
| Men | |  |  |  |  |  |  |  |  |  |  |  |  |  |  |  |  |
| Injuries | |  |  |  |  |  |  |  |  |  |  |  |  |  |  |  |  |
|  | Unintentional injuries | |  |  |  |  |  |  |  |  |  |  |  |  |  |  |  |
|  |  | Transport injuries | 0.00% | 0.00% | 0.00% |  | 2.97% | 1.43% | 4.51% |  | 7.85% | 1.99% | 13.70% |  | 2.69% | 0.00% | 5.40% |
|  |  | Poisonings | 0.00% | 0.00% | 0.00% |  | 2.62% | 0.39% | 4.84% |  | 6.97% | 1.01% | 12.94% |  | 3.34% | 0.01% | 6.68% |
|  |  | Falls | 0.00% | 0.00% | 0.00% |  | 2.62% | 0.39% | 4.84% |  | 6.97% | 1.01% | 12.94% |  | 3.34% | 0.01% | 6.68% |
|  |  | Fires, heat and hot substances | 0.00% | 0.00% | 0.00% |  | 2.62% | 0.39% | 4.84% |  | 6.97% | 1.01% | 12.94% |  | 3.34% | 0.01% | 6.68% |
|  |  | Drownings | 0.00% | 0.00% | 0.00% |  | 2.62% | 0.39% | 4.84% |  | 6.97% | 1.01% | 12.94% |  | 3.34% | 0.01% | 6.68% |
|  |  | Other unintentional injuries | 0.00% | 0.00% | 0.00% |  | 2.62% | 0.39% | 4.84% |  | 6.97% | 1.01% | 12.94% |  | 3.34% | 0.01% | 6.68% |
|  | Intentional injuries | |  |  |  |  |  |  |  |  |  |  |  |  |  |  |  |
|  |  | Self-inflicted injuries | 0.00% | 0.00% | 0.00% |  | 2.62% | 0.39% | 4.84% |  | 6.97% | 1.01% | 12.94% |  | 3.34% | 0.01% | 6.68% |
|  |  | Violence | 0.00% | 0.00% | 0.00% |  | 0.00% | 0.00% | 0.00% |  | 0.00% | 0.00% | 0.00% |  | 0.00% | 0.00% | 0.00% |
|  |  | Other intentional injuries | 0.00% | 0.00% | 0.00% |  | 2.62% | 0.3% | 4.84% |  | 6.97% | 1.01% | 12.94% |  | 3.34% | 0.01% | 6.68% |

Table 5: Morbidity Alcohol-Attributable Fractions for injuries (without harms to others included): Asia Southeast

|  |  |  | 0 to 14 years of age | | |  | 15 to 34 years of age | | |  | 35 to 64 years of age | | |  | 65 years of age and older | | |
| --- | --- | --- | --- | --- | --- | --- | --- | --- | --- | --- | --- | --- | --- | --- | --- | --- | --- |
|  |  |  | Point estimate | Lower 95% CI | Upper 95% CI |  | Point estimate | Lower 95% CI | Upper 95% CI |  | Point estimate | Lower 95% CI | Upper 95% CI |  | Point estimate | Lower 95% CI | Upper 95% CI |
| Women | |  |  |  |  |  |  |  |  |  |  |  |  |  |  |  |  |
| Injuries | |  |  |  |  |  |  |  |  |  |  |  |  |  |  |  |  |
|  | Unintentional injuries | |  |  |  |  |  |  |  |  |  |  |  |  |  |  |  |
|  |  | Transport injuries | 0.00% | 0.00% | 0.00% |  | 0.22% | 0.13% | 0.31% |  | 0.62% | 0.00% | 1.48% |  | 0.09% | 0.00% | 0.19% |
|  |  | Poisonings | 0.00% | 0.00% | 0.00% |  | 0.20% | 0.00% | 0.79% |  | 0.49% | 0.00% | 2.22% |  | 0.02% | 0.00% | 0.08% |
|  |  | Falls | 0.00% | 0.00% | 0.00% |  | 0.20% | 0.00% | 0.79% |  | 0.49% | 0.00% | 2.22% |  | 0.02% | 0.00% | 0.08% |
|  |  | Fires, heat and hot substances | 0.00% | 0.00% | 0.00% |  | 0.20% | 0.00% | 0.79% |  | 0.49% | 0.00% | 2.22% |  | 0.02% | 0.00% | 0.08% |
|  |  | Drownings | 0.00% | 0.00% | 0.00% |  | 0.20% | 0.00% | 0.79% |  | 0.49% | 0.00% | 2.22% |  | 0.02% | 0.00% | 0.08% |
|  |  | Other unintentional injuries | 0.00% | 0.00% | 0.00% |  | 0.20% | 0.00% | 0.79% |  | 0.49% | 0.00% | 2.22% |  | 0.02% | 0.00% | 0.08% |
|  | Intentional injuries | |  |  |  |  |  |  |  |  |  |  |  |  |  |  |  |
|  |  | Self-inflicted injuries | 0.00% | 0.00% | 0.00% |  | 0.20% | 0.00% | 0.79% |  | 0.49% | 0.00% | 2.22% |  | 0.02% | 0.00% | 0.08% |
|  |  | Violence | 0.00% | 0.00% | 0.00% |  | 0.00% | 0.00% | 0.00% |  | 0.00% | 0.00% | 0.00% |  | 0.00% | 0.00% | 0.00% |
|  |  | Other intentional injuries | 0.00% | 0.00% | 0.00% |  | 0.20% | 0.00% | 0.79% |  | 0.49% | 0.00% | 2.22% |  | 0.02% | 0.00% | 0.08% |
| Men | |  |  |  |  |  |  |  |  |  |  |  |  |  |  |  |  |
| Injuries | |  |  |  |  |  |  |  |  |  |  |  |  |  |  |  |  |
|  | Unintentional injuries | |  |  |  |  |  |  |  |  |  |  |  |  |  |  |  |
|  |  | Transport injuries | 0.00% | 0.00% | 0.00% |  | 2.48% | 1.47% | 3.50% |  | 6.94% | 0.00% | 16.49% |  | 1.05% | 0.04% | 2.07% |
|  |  | Poisonings | 0.00% | 0.00% | 0.00% |  | 2.34% | 0.70% | 3.97% |  | 9.68% | 2.55% | 16.81% |  | 2.97% | 0.62% | 5.33% |
|  |  | Falls | 0.00% | 0.00% | 0.00% |  | 2.34% | 0.70% | 3.97% |  | 9.68% | 2.55% | 16.81% |  | 2.97% | 0.62% | 5.33% |
|  |  | Fires, heat and hot substances | 0.00% | 0.00% | 0.00% |  | 2.34% | 0.70% | 3.97% |  | 9.68% | 2.55% | 16.81% |  | 2.97% | 0.62% | 5.33% |
|  |  | Drownings | 0.00% | 0.00% | 0.00% |  | 2.34% | 0.70% | 3.97% |  | 9.68% | 2.55% | 16.81% |  | 2.97% | 0.62% | 5.33% |
|  |  | Other unintentional injuries | 0.00% | 0.00% | 0.00% |  | 2.34% | 0.70% | 3.97% |  | 9.68% | 2.55% | 16.81% |  | 2.97% | 0.62% | 5.33% |
|  | Intentional injuries | |  |  |  |  |  |  |  |  |  |  |  |  |  |  |  |
|  |  | Self-inflicted injuries | 0.00% | 0.00% | 0.00% |  | 2.34% | 0.70% | 3.97% |  | 9.68% | 2.55% | 16.81% |  | 2.97% | 0.62% | 5.33% |
|  |  | Violence | 0.00% | 0.00% | 0.00% |  | 0.00% | 0.00% | 0.00% |  | 0.00% | 0.00% | 0.00% |  | 0.00% | 0.00% | 0.00% |
|  |  | Other intentional injuries | 0.00% | 0.00% | 0.00% |  | 2.34% | 0.70% | 3.97% |  | 9.68% | 2.55% | 16.81% |  | 2.97% | 0.62% | 5.33% |

Table 6: Morbidity Alcohol-Attributable Fractions for injuries (without harms to others included): Australasia

|  |  |  | 0 to 14 years of age | | |  | 15 to 34 years of age | | |  | 35 to 64 years of age | | |  | 65 years of age and older | | |
| --- | --- | --- | --- | --- | --- | --- | --- | --- | --- | --- | --- | --- | --- | --- | --- | --- | --- |
|  |  |  | Point estimate | Lower 95% CI | Upper 95% CI |  | Point estimate | Lower 95% CI | Upper 95% CI |  | Point estimate | Lower 95% CI | Upper 95% CI |  | Point estimate | Lower 95% CI | Upper 95% CI |
| Women | |  |  |  |  |  |  |  |  |  |  |  |  |  |  |  |  |
| Injuries | |  |  |  |  |  |  |  |  |  |  |  |  |  |  |  |  |
|  | Unintentional injuries | |  |  |  |  |  |  |  |  |  |  |  |  |  |  |  |
|  |  | Transport injuries | 0.00% | 0.00% | 0.00% |  | 1.92% | 1.18% | 2.65% |  | 2.27% | 1.24% | 3.31% |  | 0.88% | 0.41% | 1.34% |
|  |  | Poisonings | 0.00% | 0.00% | 0.00% |  | 3.04% | 1.46% | 4.62% |  | 2.84% | 1.33% | 4.36% |  | 1.35% | 0.63% | 2.06% |
|  |  | Falls | 0.00% | 0.00% | 0.00% |  | 3.04% | 1.46% | 4.62% |  | 2.84% | 1.33% | 4.36% |  | 1.35% | 0.63% | 2.06% |
|  |  | Fires, heat and hot substances | 0.00% | 0.00% | 0.00% |  | 3.04% | 1.46% | 4.62% |  | 2.84% | 1.33% | 4.36% |  | 1.35% | 0.63% | 2.06% |
|  |  | Drownings | 0.00% | 0.00% | 0.00% |  | 3.04% | 1.46% | 4.62% |  | 2.84% | 1.33% | 4.36% |  | 1.35% | 0.63% | 2.06% |
|  |  | Other unintentional injuries | 0.00% | 0.00% | 0.00% |  | 3.04% | 1.46% | 4.62% |  | 2.84% | 1.33% | 4.36% |  | 1.35% | 0.63% | 2.06% |
|  | Intentional injuries | |  |  |  |  |  |  |  |  |  |  |  |  |  |  |  |
|  |  | Self-inflicted injuries | 0.00% | 0.00% | 0.00% |  | 3.04% | 1.46% | 4.62% |  | 2.84% | 1.33% | 4.36% |  | 1.35% | 0.63% | 2.06% |
|  |  | Violence | 0.00% | 0.00% | 0.00% |  | 0.00% | 0.00% | 0.00% |  | 0.00% | 0.00% | 0.00% |  | 0.00% | 0.00% | 0.00% |
|  |  | Other intentional injuries | 0.00% | 0.00% | 0.00% |  | 3.04% | 1.46% | 4.62% |  | 2.84% | 1.33% | 4.36% |  | 1.35% | 0.63% | 2.06% |
| Men | |  |  |  |  |  |  |  |  |  |  |  |  |  |  |  |  |
| Injuries | |  |  |  |  |  |  |  |  |  |  |  |  |  |  |  |  |
|  | Unintentional injuries | |  |  |  |  |  |  |  |  |  |  |  |  |  |  |  |
|  |  | Transport injuries | 0.00% | 0.00% | 0.00% |  | 4.74% | 2.92% | 6.56% |  | 5.63% | 3.08% | 8.17% |  | 2.17% | 1.01% | 3.32% |
|  |  | Poisonings | 0.00% | 0.00% | 0.00% |  | 8.63% | 4.02% | 13.23% |  | 12.03% | 5.79% | 18.28% |  | 7.29% | 3.22% | 11.35% |
|  |  | Falls | 0.00% | 0.00% | 0.00% |  | 8.63% | 4.02% | 13.23% |  | 12.03% | 5.79% | 18.28% |  | 7.29% | 3.22% | 11.35% |
|  |  | Fires, heat and hot substances | 0.00% | 0.00% | 0.00% |  | 8.63% | 4.02% | 13.23% |  | 12.03% | 5.79% | 18.28% |  | 7.29% | 3.22% | 11.35% |
|  |  | Drownings | 0.00% | 0.00% | 0.00% |  | 8.63% | 4.02% | 13.23% |  | 12.03% | 5.79% | 18.28% |  | 7.29% | 3.22% | 11.35% |
|  |  | Other unintentional injuries | 0.00% | 0.00% | 0.00% |  | 8.63% | 4.02% | 13.23% |  | 12.03% | 5.79% | 18.28% |  | 7.29% | 3.22% | 11.35% |
|  | Intentional injuries | |  |  |  |  |  |  |  |  |  |  |  |  |  |  |  |
|  |  | Self-inflicted injuries | 0.00% | 0.00% | 0.00% |  | 8.63% | 4.02% | 13.23% |  | 12.03% | 5.79% | 18.28% |  | 7.29% | 3.22% | 11.35% |
|  |  | Violence | 0.00% | 0.00% | 0.00% |  | 0.00% | 0.00% | 0.00% |  | 0.00% | 0.00% | 0.00% |  | 0.00% | 0.00% | 0.00% |
|  |  | Other intentional injuries | 0.00% | 0.00% | 0.00% |  | 8.63% | 4.02% | 13.23% |  | 12.03% | 5.79% | 18.28% |  | 7.29% | 3.22% | 11.35% |

Table 7: Morbidity Alcohol-Attributable Fractions for injuries (without harms to others included): Caribbean

|  |  |  | 0 to 14 years of age | | |  | 15 to 34 years of age | | |  | 35 to 64 years of age | | |  | 65 years of age and older | | |
| --- | --- | --- | --- | --- | --- | --- | --- | --- | --- | --- | --- | --- | --- | --- | --- | --- | --- |
|  |  |  | Point estimate | Lower 95% CI | Upper 95% CI |  | Point estimate | Lower 95% CI | Upper 95% CI |  | Point estimate | Lower 95% CI | Upper 95% CI |  | Point estimate | Lower 95% CI | Upper 95% CI |
| Women | |  |  |  |  |  |  |  |  |  |  |  |  |  |  |  |  |
| Injuries | |  |  |  |  |  |  |  |  |  |  |  |  |  |  |  |  |
|  | Unintentional injuries | |  |  |  |  |  |  |  |  |  |  |  |  |  |  |  |
|  |  | Transport injuries | 0.00% | 0.00% | 0.00% |  | 1.40% | 0.86% | 1.94% |  | 1.48% | 0.88% | 2.08% |  | 0.42% | 0.22% | 0.63% |
|  |  | Poisonings | 0.00% | 0.00% | 0.00% |  | 2.54% | 0.33% | 4.75% |  | 0.70% | 0.23% | 1.16% |  | 0.11% | 0.03% | 0.19% |
|  |  | Falls | 0.00% | 0.00% | 0.00% |  | 2.54% | 0.33% | 4.75% |  | 0.70% | 0.23% | 1.16% |  | 0.11% | 0.03% | 0.19% |
|  |  | Fires, heat and hot substances | 0.00% | 0.00% | 0.00% |  | 2.54% | 0.33% | 4.75% |  | 0.70% | 0.23% | 1.16% |  | 0.11% | 0.03% | 0.19% |
|  |  | Drownings | 0.00% | 0.00% | 0.00% |  | 2.54% | 0.33% | 4.75% |  | 0.70% | 0.23% | 1.16% |  | 0.11% | 0.03% | 0.19% |
|  |  | Other unintentional injuries | 0.00% | 0.00% | 0.00% |  | 2.54% | 0.33% | 4.75% |  | 0.70% | 0.23% | 1.16% |  | 0.11% | 0.03% | 0.19% |
|  | Intentional injuries | |  |  |  |  |  |  |  |  |  |  |  |  |  |  |  |
|  |  | Self-inflicted injuries | 0.00% | 0.00% | 0.00% |  | 2.54% | 0.33% | 4.75% |  | 0.70% | 0.23% | 1.16% |  | 0.11% | 0.03% | 0.19% |
|  |  | Violence | 0.00% | 0.00% | 0.00% |  | 0.00% | 0.00% | 0.00% |  | 0.00% | 0.00% | 0.00% |  | 0.00% | 0.00% | 0.00% |
|  |  | Other intentional injuries | 0.00% | 0.00% | 0.00% |  | 2.54% | 0.33% | 4.75% |  | 0.70% | 0.23% | 1.16% |  | 0.11% | 0.03% | 0.19% |
| Men | |  |  |  |  |  |  |  |  |  |  |  |  |  |  |  |  |
| Injuries | |  |  |  |  |  |  |  |  |  |  |  |  |  |  |  |  |
|  | Unintentional injuries | |  |  |  |  |  |  |  |  |  |  |  |  |  |  |  |
|  |  | Transport injuries | 0.00% | 0.00% | 0.00% |  | 4.77% | 2.93% | 6.62% |  | 5.05% | 2.99% | 7.10% |  | 1.44% | 0.74% | 2.14% |
|  |  | Poisonings | 0.00% | 0.00% | 0.00% |  | 5.74% | 2.33% | 9.15% |  | 7.76% | 3.19% | 12.34% |  | 3.39% | 1.20% | 5.58% |
|  |  | Falls | 0.00% | 0.00% | 0.00% |  | 5.74% | 2.33% | 9.15% |  | 7.76% | 3.19% | 12.34% |  | 3.39% | 1.20% | 5.58% |
|  |  | Fires, heat and hot substances | 0.00% | 0.00% | 0.00% |  | 5.74% | 2.33% | 9.15% |  | 7.76% | 3.19% | 12.34% |  | 3.39% | 1.20% | 5.58% |
|  |  | Drownings | 0.00% | 0.00% | 0.00% |  | 5.74% | 2.33% | 9.15% |  | 7.76% | 3.19% | 12.34% |  | 3.39% | 1.20% | 5.58% |
|  |  | Other unintentional injuries | 0.00% | 0.00% | 0.00% |  | 5.74% | 2.33% | 9.15% |  | 7.76% | 3.19% | 12.34% |  | 3.39% | 1.20% | 5.58% |
|  | Intentional injuries | |  |  |  |  |  |  |  |  |  |  |  |  |  |  |  |
|  |  | Self-inflicted injuries | 0.00% | 0.00% | 0.00% |  | 5.74% | 2.33% | 9.15% |  | 7.76% | 3.19% | 12.34% |  | 3.39% | 1.20% | 5.58% |
|  |  | Violence | 0.00% | 0.00% | 0.00% |  | 0.00% | 0.00% | 0.00% |  | 0.00% | 0.00% | 0.00% |  | 0.00% | 0.00% | 0.00% |
|  |  | Other intentional injuries | 0.00% | 0.00% | 0.00% |  | 5.74% | 2.33% | 9.15% |  | 7.76% | 3.19% | 12.34% |  | 3.39% | 1.20% | 5.58% |

Table 8: Morbidity Alcohol-Attributable Fractions for injuries (without harms to others included): Europe Central

|  |  |  | 0 to 14 years of age | | |  | 15 to 34 years of age | | |  | 35 to 64 years of age | | |  | 65 years of age and older | | |
| --- | --- | --- | --- | --- | --- | --- | --- | --- | --- | --- | --- | --- | --- | --- | --- | --- | --- |
|  |  |  | Point estimate | Lower 95% CI | Upper 95% CI |  | Point estimate | Lower 95% CI | Upper 95% CI |  | Point estimate | Lower 95% CI | Upper 95% CI |  | Point estimate | Lower 95% CI | Upper 95% CI |
| Women | |  |  |  |  |  |  |  |  |  |  |  |  |  |  |  |  |
| Injuries | |  |  |  |  |  |  |  |  |  |  |  |  |  |  |  |  |
|  | Unintentional injuries | |  |  |  |  |  |  |  |  |  |  |  |  |  |  |  |
|  |  | Transport injuries | 0.00% | 0.00% | 0.00% |  | 4.92% | 3.01% | 6.83% |  | 8.26% | 2.42% | 14.11% |  | 3.61% | 0.91% | 6.31% |
|  |  | Poisonings | 0.00% | 0.00% | 0.00% |  | 5.09% | 0.07% | 10.12% |  | 6.18% | 0.23% | 12.12% |  | 0.45% | 0.09% | 0.82% |
|  |  | Falls | 0.00% | 0.00% | 0.00% |  | 5.09% | 0.07% | 10.12% |  | 6.18% | 0.23% | 12.12% |  | 0.45% | 0.09% | 0.82% |
|  |  | Fires, heat and hot substances | 0.00% | 0.00% | 0.00% |  | 5.09% | 0.07% | 10.12% |  | 6.18% | 0.23% | 12.12% |  | 0.45% | 0.09% | 0.82% |
|  |  | Drownings | 0.00% | 0.00% | 0.00% |  | 5.09% | 0.07% | 10.12% |  | 6.18% | 0.23% | 12.12% |  | 0.45% | 0.09% | 0.82% |
|  |  | Other unintentional injuries | 0.00% | 0.00% | 0.00% |  | 5.09% | 0.07% | 10.12% |  | 6.18% | 0.23% | 12.12% |  | 0.45% | 0.09% | 0.82% |
|  | Intentional injuries | |  |  |  |  |  |  |  |  |  |  |  |  |  |  |  |
|  |  | Self-inflicted injuries | 0.00% | 0.00% | 0.00% |  | 5.09% | 0.07% | 10.12% |  | 6.18% | 0.23% | 12.12% |  | 0.45% | 0.09% | 0.82% |
|  |  | Violence | 0.00% | 0.00% | 0.00% |  | 0.00% | 0.00% | 0.00% |  | 0.00% | 0.00% | 0.00% |  | 0.00% | 0.00% | 0.00% |
|  |  | Other intentional injuries | 0.00% | 0.00% | 0.00% |  | 5.09% | 0.07% | 10.12% |  | 6.18% | 0.23% | 12.12% |  | 0.45% | 0.09% | 0.82% |
| Men | |  |  |  |  |  |  |  |  |  |  |  |  |  |  |  |  |
| Injuries | |  |  |  |  |  |  |  |  |  |  |  |  |  |  |  |  |
|  | Unintentional injuries | |  |  |  |  |  |  |  |  |  |  |  |  |  |  |  |
|  |  | Transport injuries | 0.00% | 0.00% | 0.00% |  | 16.01% | 9.79% | 22.24% |  | 26.89% | 7.87% | 45.91% |  | 11.75% | 2.97% | 20.53% |
|  |  | Poisonings | 0.00% | 0.00% | 0.00% |  | 16.99% | 7.81% | 26.18% |  | 29.66% | 14.84% | 44.49% |  | 18.48% | 8.03% | 28.92% |
|  |  | Falls | 0.00% | 0.00% | 0.00% |  | 16.99% | 7.81% | 26.18% |  | 29.66% | 14.84% | 44.49% |  | 18.48% | 8.03% | 28.92% |
|  |  | Fires, heat and hot substances | 0.00% | 0.00% | 0.00% |  | 16.99% | 7.81% | 26.18% |  | 29.66% | 14.84% | 44.49% |  | 18.48% | 8.03% | 28.92% |
|  |  | Drownings | 0.00% | 0.00% | 0.00% |  | 16.99% | 7.81% | 26.18% |  | 29.66% | 14.84% | 44.49% |  | 18.48% | 8.03% | 28.92% |
|  |  | Other unintentional injuries | 0.00% | 0.00% | 0.00% |  | 16.99% | 7.81% | 26.18% |  | 29.66% | 14.84% | 44.49% |  | 18.48% | 8.03% | 28.92% |
|  | Intentional injuries | |  |  |  |  |  |  |  |  |  |  |  |  |  |  |  |
|  |  | Self-inflicted injuries | 0.00% | 0.00% | 0.00% |  | 16.99% | 7.81% | 26.18% |  | 29.66% | 14.84% | 44.49% |  | 18.48% | 8.03% | 28.92% |
|  |  | Violence | 0.00% | 0.00% | 0.00% |  | 0.00% | 0.00% | 0.00% |  | 0.00% | 0.00% | 0.00% |  | 0.00% | 0.00% | 0.00% |
|  |  | Other intentional injuries | 0.00% | 0.00% | 0.00% |  | 16.99% | 7.81% | 26.18% |  | 29.66% | 14.84% | 44.49% |  | 18.48% | 8.03% | 28.92% |

Table 9: Morbidity Alcohol-Attributable Fractions for injuries (without harms to others included): Europe Eastern

|  |  |  | 0 to 14 years of age | | |  | 15 to 34 years of age | | |  | 35 to 64 years of age | | |  | 65 years of age and older | | |
| --- | --- | --- | --- | --- | --- | --- | --- | --- | --- | --- | --- | --- | --- | --- | --- | --- | --- |
|  |  |  | Point estimate | Lower 95% CI | Upper 95% CI |  | Point estimate | Lower 95% CI | Upper 95% CI |  | Point estimate | Lower 95% CI | Upper 95% CI |  | Point estimate | Lower 95% CI | Upper 95% CI |
| Women | |  |  |  |  |  |  |  |  |  |  |  |  |  |  |  |  |
| Injuries | |  |  |  |  |  |  |  |  |  |  |  |  |  |  |  |  |
|  | Unintentional injuries | |  |  |  |  |  |  |  |  |  |  |  |  |  |  |  |
|  |  | Transport injuries | 0.00% | 0.00% | 0.00% |  | 19.22% | 19.22% | 19.22% |  | 19.22% | 19.22% | 19.22% |  | 18.23% | 13.98% | 19.22% |
|  |  | Poisonings | 0.00% | 0.00% | 0.00% |  | 13.08% | 1.48% | 24.69% |  | 10.53% | 0.76% | 20.30% |  | 1.27% | 0.00% | 2.73% |
|  |  | Falls | 0.00% | 0.00% | 0.00% |  | 13.08% | 1.48% | 24.69% |  | 10.53% | 0.76% | 20.30% |  | 1.27% | 0.00% | 2.73% |
|  |  | Fires, heat and hot substances | 0.00% | 0.00% | 0.00% |  | 13.08% | 1.48% | 24.69% |  | 10.53% | 0.76% | 20.30% |  | 1.27% | 0.00% | 2.73% |
|  |  | Drownings | 0.00% | 0.00% | 0.00% |  | 13.08% | 1.48% | 24.69% |  | 10.53% | 0.76% | 20.30% |  | 1.27% | 0.00% | 2.73% |
|  |  | Other unintentional injuries | 0.00% | 0.00% | 0.00% |  | 13.08% | 1.48% | 24.69% |  | 10.53% | 0.76% | 20.30% |  | 1.27% | 0.00% | 2.73% |
|  | Intentional injuries | |  |  |  |  |  |  |  |  |  |  |  |  |  |  |  |
|  |  | Self-inflicted injuries | 0.00% | 0.00% | 0.00% |  | 13.08% | 1.48% | 24.69% |  | 10.53% | 0.76% | 20.30% |  | 1.27% | 0.00% | 2.73% |
|  |  | Violence | 0.00% | 0.00% | 0.00% |  | 0.00% | 0.00% | 0.00% |  | 0.00% | 0.00% | 0.00% |  | 0.00% | 0.00% | 0.00% |
|  |  | Other intentional injuries | 0.00% | 0.00% | 0.00% |  | 13.08% | 1.48% | 24.69% |  | 10.53% | 0.76% | 20.30% |  | 1.27% | 0.00% | 2.73% |
| Men | |  |  |  |  |  |  |  |  |  |  |  |  |  |  |  |  |
| Injuries | |  |  |  |  |  |  |  |  |  |  |  |  |  |  |  |  |
|  | Unintentional injuries | |  |  |  |  |  |  |  |  |  |  |  |  |  |  |  |
|  |  | Transport injuries | 0.00% | 0.00% | 0.00% |  | 60.00% | 60.00% | 60.00% |  | 60.00% | 60.00% | 60.00% |  | 56.91% | 43.66% | 60.00% |
|  |  | Poisonings | 0.00% | 0.00% | 0.00% |  | 37.05% | 20.67% | 53.43% |  | 33.29% | 18.39% | 48.18% |  | 19.13% | 8.87% | 29.39% |
|  |  | Falls | 0.00% | 0.00% | 0.00% |  | 37.05% | 20.67% | 53.43% |  | 33.29% | 18.39% | 48.18% |  | 19.13% | 8.87% | 29.39% |
|  |  | Fires, heat and hot substances | 0.00% | 0.00% | 0.00% |  | 37.05% | 20.67% | 53.43% |  | 33.29% | 18.39% | 48.18% |  | 19.13% | 8.87% | 29.39% |
|  |  | Drownings | 0.00% | 0.00% | 0.00% |  | 37.05% | 20.67% | 53.43% |  | 33.29% | 18.39% | 48.18% |  | 19.13% | 8.87% | 29.39% |
|  |  | Other unintentional injuries | 0.00% | 0.00% | 0.00% |  | 37.05% | 20.67% | 53.43% |  | 33.29% | 18.39% | 48.18% |  | 19.13% | 8.87% | 29.39% |
|  | Intentional injuries | |  |  |  |  |  |  |  |  |  |  |  |  |  |  |  |
|  |  | Self-inflicted injuries | 0.00% | 0.00% | 0.00% |  | 37.05% | 20.67% | 53.43% |  | 33.29% | 18.39% | 48.18% |  | 19.13% | 8.87% | 29.39% |
|  |  | Violence | 0.00% | 0.00% | 0.00% |  | 0.00% | 0.00% | 0.00% |  | 0.00% | 0.00% | 0.00% |  | 0.00% | 0.00% | 0.00% |
|  |  | Other intentional injuries | 0.00% | 0.00% | 0.00% |  | 37.05% | 20.67% | 53.43% |  | 33.29% | 18.39% | 48.18% |  | 19.13% | 8.87% | 29.39% |

Table 10: Morbidity Alcohol-Attributable Fractions for injuries (without harms to others included): Europe Western

|  |  |  | 0 to 14 years of age | | |  | 15 to 34 years of age | | |  | 35 to 64 years of age | | |  | 65 years of age and older | | |
| --- | --- | --- | --- | --- | --- | --- | --- | --- | --- | --- | --- | --- | --- | --- | --- | --- | --- |
|  |  |  | Point estimate | Lower 95% CI | Upper 95% CI |  | Point estimate | Lower 95% CI | Upper 95% CI |  | Point estimate | Lower 95% CI | Upper 95% CI |  | Point estimate | Lower 95% CI | Upper 95% CI |
| Women | |  |  |  |  |  |  |  |  |  |  |  |  |  |  |  |  |
| Injuries | |  |  |  |  |  |  |  |  |  |  |  |  |  |  |  |  |
|  | Unintentional injuries | |  |  |  |  |  |  |  |  |  |  |  |  |  |  |  |
|  |  | Transport injuries | 0.00% | 0.00% | 0.00% |  | 2.76% | 1.50% | 4.02% |  | 3.69% | 1.44% | 5.94% |  | 1.25% | 0.49% | 2.00% |
|  |  | Poisonings | 0.00% | 0.00% | 0.00% |  | 2.81% | 1.02% | 4.60% |  | 4.92% | 0.57% | 9.26% |  | 1.76% | 0.64% | 2.88% |
|  |  | Falls | 0.00% | 0.00% | 0.00% |  | 2.81% | 1.02% | 4.60% |  | 4.92% | 0.57% | 9.26% |  | 1.76% | 0.64% | 2.88% |
|  |  | Fires, heat and hot substances | 0.00% | 0.00% | 0.00% |  | 2.81% | 1.02% | 4.60% |  | 4.92% | 0.57% | 9.26% |  | 1.76% | 0.64% | 2.88% |
|  |  | Drownings | 0.00% | 0.00% | 0.00% |  | 2.81% | 1.02% | 4.60% |  | 4.92% | 0.57% | 9.26% |  | 1.76% | 0.64% | 2.88% |
|  |  | Other unintentional injuries | 0.00% | 0.00% | 0.00% |  | 2.81% | 1.02% | 4.60% |  | 4.92% | 0.57% | 9.26% |  | 1.76% | 0.64% | 2.88% |
|  | Intentional injuries | |  |  |  |  |  |  |  |  |  |  |  |  |  |  |  |
|  |  | Self-inflicted injuries | 0.00% | 0.00% | 0.00% |  | 2.81% | 1.02% | 4.60% |  | 4.92% | 0.57% | 9.26% |  | 1.76% | 0.64% | 2.88% |
|  |  | Violence | 0.00% | 0.00% | 0.00% |  | 0.00% | 0.00% | 0.00% |  | 0.00% | 0.00% | 0.00% |  | 0.00% | 0.00% | 0.00% |
|  |  | Other intentional injuries | 0.00% | 0.00% | 0.00% |  | 2.81% | 1.02% | 4.60% |  | 4.92% | 0.57% | 9.26% |  | 1.76% | 0.64% | 2.88% |
| Men | |  |  |  |  |  |  |  |  |  |  |  |  |  |  |  |  |
| Injuries | |  |  |  |  |  |  |  |  |  |  |  |  |  |  |  |  |
|  | Unintentional injuries | |  |  |  |  |  |  |  |  |  |  |  |  |  |  |  |
|  |  | Transport injuries | 0.00% | 0.00% | 0.00% |  | 6.91% | 3.76% | 10.06% |  | 9.23% | 3.61% | 14.85% |  | 3.12% | 1.23% | 5.00% |
|  |  | Poisonings | 0.00% | 0.00% | 0.00% |  | 12.35% | 5.54% | 19.16% |  | 17.51% | 8.21% | 26.81% |  | 9.12% | 3.82% | 14.42% |
|  |  | Falls | 0.00% | 0.00% | 0.00% |  | 12.35% | 5.54% | 19.16% |  | 17.51% | 8.21% | 26.81% |  | 9.12% | 3.82% | 14.42% |
|  |  | Fires, heat and hot substances | 0.00% | 0.00% | 0.00% |  | 12.35% | 5.54% | 19.16% |  | 17.51% | 8.21% | 26.81% |  | 9.12% | 3.82% | 14.42% |
|  |  | Drownings | 0.00% | 0.00% | 0.00% |  | 12.35% | 5.54% | 19.16% |  | 17.51% | 8.21% | 26.81% |  | 9.12% | 3.82% | 14.42% |
|  |  | Other unintentional injuries | 0.00% | 0.00% | 0.00% |  | 12.35% | 5.54% | 19.16% |  | 17.51% | 8.21% | 26.81% |  | 9.12% | 3.82% | 14.42% |
|  | Intentional injuries | |  |  |  |  |  |  |  |  |  |  |  |  |  |  |  |
|  |  | Self-inflicted injuries | 0.00% | 0.00% | 0.00% |  | 12.35% | 5.54% | 19.16% |  | 17.51% | 8.21% | 26.81% |  | 9.12% | 3.82% | 14.42% |
|  |  | Violence | 0.00% | 0.00% | 0.00% |  | 0.00% | 0.00% | 0.00% |  | 0.00% | 0.00% | 0.00% |  | 0.00% | 0.00% | 0.00% |
|  |  | Other intentional injuries | 0.00% | 0.00% | 0.00% |  | 12.35% | 5.54% | 19.16% |  | 17.51% | 8.21% | 26.81% |  | 9.12% | 3.82% | 14.42% |

Table 11: Morbidity Alcohol-Attributable Fractions for injuries (without harms to others included): Latin America Andean

|  |  |  | 0 to 14 years of age | | |  | 15 to 34 years of age | | |  | 35 to 64 years of age | | |  | 65 years of age and older | | |
| --- | --- | --- | --- | --- | --- | --- | --- | --- | --- | --- | --- | --- | --- | --- | --- | --- | --- |
|  |  |  | Point estimate | Lower 95% CI | Upper 95% CI |  | Point estimate | Lower 95% CI | Upper 95% CI |  | Point estimate | Lower 95% CI | Upper 95% CI |  | Point estimate | Lower 95% CI | Upper 95% CI |
| Women | |  |  |  |  |  |  |  |  |  |  |  |  |  |  |  |  |
| Injuries | |  |  |  |  |  |  |  |  |  |  |  |  |  |  |  |  |
|  | Unintentional injuries | |  |  |  |  |  |  |  |  |  |  |  |  |  |  |  |
|  |  | Transport injuries | 0.00% | 0.00% | 0.00% |  | 2.24% | 1.35% | 3.12% |  | 2.98% | 1.42% | 4.54% |  | 0.36% | 0.20% | 0.52% |
|  |  | Poisonings | 0.00% | 0.00% | 0.00% |  | 2.17% | 0.00% | 5.65% |  | 1.52% | 0.00% | 3.31% |  | 0.76% | 0.00% | 1.77% |
|  |  | Falls | 0.00% | 0.00% | 0.00% |  | 2.17% | 0.00% | 5.65% |  | 1.52% | 0.00% | 3.31% |  | 0.76% | 0.00% | 1.77% |
|  |  | Fires, heat and hot substances | 0.00% | 0.00% | 0.00% |  | 2.17% | 0.00% | 5.65% |  | 1.52% | 0.00% | 3.31% |  | 0.76% | 0.00% | 1.77% |
|  |  | Drownings | 0.00% | 0.00% | 0.00% |  | 2.17% | 0.00% | 5.65% |  | 1.52% | 0.00% | 3.31% |  | 0.76% | 0.00% | 1.77% |
|  |  | Other unintentional injuries | 0.00% | 0.00% | 0.00% |  | 2.17% | 0.00% | 5.65% |  | 1.52% | 0.00% | 3.31% |  | 0.76% | 0.00% | 1.77% |
|  | Intentional injuries | |  |  |  |  |  |  |  |  |  |  |  |  |  |  |  |
|  |  | Self-inflicted injuries | 0.00% | 0.00% | 0.00% |  | 2.17% | 0.00% | 5.65% |  | 1.52% | 0.00% | 3.31% |  | 0.76% | 0.00% | 1.77% |
|  |  | Violence | 0.00% | 0.00% | 0.00% |  | 0.00% | 0.00% | 0.00% |  | 0.00% | 0.00% | 0.00% |  | 0.00% | 0.00% | 0.00% |
|  |  | Other intentional injuries | 0.00% | 0.00% | 0.00% |  | 2.17% | 0.00% | 5.65% |  | 1.52% | 0.00% | 3.31% |  | 0.76% | 0.00% | 1.77% |
| Men | |  |  |  |  |  |  |  |  |  |  |  |  |  |  |  |  |
| Injuries | |  |  |  |  |  |  |  |  |  |  |  |  |  |  |  |  |
|  | Unintentional injuries | |  |  |  |  |  |  |  |  |  |  |  |  |  |  |  |
|  |  | Transport injuries | 0.00% | 0.00% | 0.00% |  | 7.40% | 4.48% | 10.33% |  | 9.86% | 4.70% | 15.01% |  | 1.19% | 0.65% | 1.73% |
|  |  | Poisonings | 0.00% | 0.00% | 0.00% |  | 7.46% | 2.14% | 12.78% |  | 12.65% | 3.64% | 21.66% |  | 1.29% | 0.33% | 2.25% |
|  |  | Falls | 0.00% | 0.00% | 0.00% |  | 7.46% | 2.14% | 12.78% |  | 12.65% | 3.64% | 21.66% |  | 1.29% | 0.33% | 2.25% |
|  |  | Fires, heat and hot substances | 0.00% | 0.00% | 0.00% |  | 7.46% | 2.14% | 12.78% |  | 12.65% | 3.64% | 21.66% |  | 1.29% | 0.33% | 2.25% |
|  |  | Drownings | 0.00% | 0.00% | 0.00% |  | 7.46% | 2.14% | 12.78% |  | 12.65% | 3.64% | 21.66% |  | 1.29% | 0.33% | 2.25% |
|  |  | Other unintentional injuries | 0.00% | 0.00% | 0.00% |  | 7.46% | 2.14% | 12.78% |  | 12.65% | 3.64% | 21.66% |  | 1.29% | 0.33% | 2.25% |
|  | Intentional injuries | |  |  |  |  |  |  |  |  |  |  |  |  |  |  |  |
|  |  | Self-inflicted injuries | 0.00% | 0.00% | 0.00% |  | 7.46% | 2.14% | 12.78% |  | 12.65% | 3.64% | 21.66% |  | 1.29% | 0.33% | 2.25% |
|  |  | Violence | 0.00% | 0.00% | 0.00% |  | 0.00% | 0.00% | 0.00% |  | 0.00% | 0.00% | 0.00% |  | 0.00% | 0.00% | 0.00% |
|  |  | Other intentional injuries | 0.00% | 0.00% | 0.00% |  | 7.46% | 2.14% | 12.78% |  | 12.65% | 3.64% | 21.66% |  | 1.29% | 0.33% | 2.25% |

Table 12: Morbidity Alcohol-Attributable Fractions for injuries (without harms to others included): Latin America Central

|  |  |  | 0 to 14 years of age | | |  | 15 to 34 years of age | | |  | 35 to 64 years of age | | |  | 65 years of age and older | | |
| --- | --- | --- | --- | --- | --- | --- | --- | --- | --- | --- | --- | --- | --- | --- | --- | --- | --- |
|  |  |  | Point estimate | Lower 95% CI | Upper 95% CI |  | Point estimate | Lower 95% CI | Upper 95% CI |  | Point estimate | Lower 95% CI | Upper 95% CI |  | Point estimate | Lower 95% CI | Upper 95% CI |
| Women | |  |  |  |  |  |  |  |  |  |  |  |  |  |  |  |  |
| Injuries | |  |  |  |  |  |  |  |  |  |  |  |  |  |  |  |  |
|  | Unintentional injuries | |  |  |  |  |  |  |  |  |  |  |  |  |  |  |  |
|  |  | Transport injuries | 0.00% | 0.00% | 0.00% |  | 5.99% | 3.83% | 8.16% |  | 7.32% | 4.83% | 9.80% |  | 3.87% | 2.28% | 5.46% |
|  |  | Poisonings | 0.00% | 0.00% | 0.00% |  | 2.25% | 0.00% | 5.35% |  | 1.45% | 0.00% | 3.37% |  | 0.40% | 0.02% | 0.78% |
|  |  | Falls | 0.00% | 0.00% | 0.00% |  | 2.25% | 0.00% | 5.35% |  | 1.45% | 0.00% | 3.37% |  | 0.40% | 0.02% | 0.78% |
|  |  | Fires, heat and hot substances | 0.00% | 0.00% | 0.00% |  | 2.25% | 0.00% | 5.35% |  | 1.45% | 0.00% | 3.37% |  | 0.40% | 0.02% | 0.78% |
|  |  | Drownings | 0.00% | 0.00% | 0.00% |  | 2.25% | 0.00% | 5.35% |  | 1.45% | 0.00% | 3.37% |  | 0.40% | 0.02% | 0.78% |
|  |  | Other unintentional injuries | 0.00% | 0.00% | 0.00% |  | 2.25% | 0.00% | 5.35% |  | 1.45% | 0.00% | 3.37% |  | 0.40% | 0.02% | 0.78% |
|  | Intentional injuries | |  |  |  |  |  |  |  |  |  |  |  |  |  |  |  |
|  |  | Self-inflicted injuries | 0.00% | 0.00% | 0.00% |  | 2.25% | 0.00% | 5.35% |  | 1.45% | 0.00% | 3.37% |  | 0.40% | 0.02% | 0.78% |
|  |  | Violence | 0.00% | 0.00% | 0.00% |  | 0.00% | 0.00% | 0.00% |  | 0.00% | 0.00% | 0.00% |  | 0.00% | 0.00% | 0.00% |
|  |  | Other intentional injuries | 0.00% | 0.00% | 0.00% |  | 2.25% | 0.00% | 5.35% |  | 1.45% | 0.00% | 3.37% |  | 0.40% | 0.02% | 0.78% |
| Men | |  |  |  |  |  |  |  |  |  |  |  |  |  |  |  |  |
| Injuries | |  |  |  |  |  |  |  |  |  |  |  |  |  |  |  |  |
|  | Unintentional injuries | |  |  |  |  |  |  |  |  |  |  |  |  |  |  |  |
|  |  | Transport injuries | 0.00% | 0.00% | 0.00% |  | 21.77% | 13.90% | 29.64% |  | 26.58% | 17.55% | 35.60% |  | 14.05% | 8.27% | 19.82% |
|  |  | Poisonings | 0.00% | 0.00% | 0.00% |  | 10.54% | 4.29% | 16.80% |  | 14.11% | 6.02% | 22.20% |  | 8.54% | 3.07% | 14.02% |
|  |  | Falls | 0.00% | 0.00% | 0.00% |  | 10.54% | 4.29% | 16.80% |  | 14.11% | 6.02% | 22.20% |  | 8.54% | 3.07% | 14.02% |
|  |  | Fires, heat and hot substances | 0.00% | 0.00% | 0.00% |  | 10.54% | 4.29% | 16.80% |  | 14.11% | 6.02% | 22.20% |  | 8.54% | 3.07% | 14.02% |
|  |  | Drownings | 0.00% | 0.00% | 0.00% |  | 10.54% | 4.29% | 16.80% |  | 14.11% | 6.02% | 22.20% |  | 8.54% | 3.07% | 14.02% |
|  |  | Other unintentional injuries | 0.00% | 0.00% | 0.00% |  | 10.54% | 4.29% | 16.80% |  | 14.11% | 6.02% | 22.20% |  | 8.54% | 3.07% | 14.02% |
|  | Intentional injuries | |  |  |  |  |  |  |  |  |  |  |  |  |  |  |  |
|  |  | Self-inflicted injuries | 0.00% | 0.00% | 0.00% |  | 10.54% | 4.29% | 16.80% |  | 14.11% | 6.02% | 22.20% |  | 8.54% | 3.07% | 14.02% |
|  |  | Violence | 0.00% | 0.00% | 0.00% |  | 0.00% | 0.00% | 0.00% |  | 0.00% | 0.00% | 0.00% |  | 0.00% | 0.00% | 0.00% |
|  |  | Other intentional injuries | 0.00% | 0.00% | 0.00% |  | 10.54% | 4.29% | 16.80% |  | 14.11% | 6.02% | 22.20% |  | 8.54% | 3.07% | 14.02% |

Table 13: Morbidity Alcohol-Attributable Fractions for injuries (without harms to others included): Latin America Southern

|  |  |  | 0 to 14 years of age | | |  | 15 to 34 years of age | | |  | 35 to 64 years of age | | |  | 65 years of age and older | | |
| --- | --- | --- | --- | --- | --- | --- | --- | --- | --- | --- | --- | --- | --- | --- | --- | --- | --- |
|  |  |  | Point estimate | Lower 95% CI | Upper 95% CI |  | Point estimate | Lower 95% CI | Upper 95% CI |  | Point estimate | Lower 95% CI | Upper 95% CI |  | Point estimate | Lower 95% CI | Upper 95% CI |
| Women | |  |  |  |  |  |  |  |  |  |  |  |  |  |  |  |  |
| Injuries | |  |  |  |  |  |  |  |  |  |  |  |  |  |  |  |  |
|  | Unintentional injuries | |  |  |  |  |  |  |  |  |  |  |  |  |  |  |  |
|  |  | Transport injuries | 0.00% | 0.00% | 0.00% |  | 2.73% | 1.65% | 3.82% |  | 2.00% | 1.01% | 2.99% |  | 1.00% | 0.48% | 1.52% |
|  |  | Poisonings | 0.00% | 0.00% | 0.00% |  | 1.90% | 0.50% | 3.31% |  | 3.44% | 0.00% | 7.30% |  | 1.28% | 0.32% | 2.23% |
|  |  | Falls | 0.00% | 0.00% | 0.00% |  | 1.90% | 0.50% | 3.31% |  | 3.44% | 0.00% | 7.30% |  | 1.28% | 0.32% | 2.23% |
|  |  | Fires, heat and hot substances | 0.00% | 0.00% | 0.00% |  | 1.90% | 0.50% | 3.31% |  | 3.44% | 0.00% | 7.30% |  | 1.28% | 0.32% | 2.23% |
|  |  | Drownings | 0.00% | 0.00% | 0.00% |  | 1.90% | 0.50% | 3.31% |  | 3.44% | 0.00% | 7.30% |  | 1.28% | 0.32% | 2.23% |
|  |  | Other unintentional injuries | 0.00% | 0.00% | 0.00% |  | 1.90% | 0.50% | 3.31% |  | 3.44% | 0.00% | 7.30% |  | 1.28% | 0.32% | 2.23% |
|  | Intentional injuries | |  |  |  |  |  |  |  |  |  |  |  |  |  |  |  |
|  |  | Self-inflicted injuries | 0.00% | 0.00% | 0.00% |  | 1.90% | 0.50% | 3.31% |  | 3.44% | 0.00% | 7.30% |  | 1.28% | 0.32% | 2.23% |
|  |  | Violence | 0.00% | 0.00% | 0.00% |  | 0.00% | 0.00% | 0.00% |  | 0.00% | 0.00% | 0.00% |  | 0.00% | 0.00% | 0.00% |
|  |  | Other intentional injuries | 0.00% | 0.00% | 0.00% |  | 1.90% | 0.50% | 3.31% |  | 3.44% | 0.00% | 7.30% |  | 1.28% | 0.32% | 2.23% |
| Men | |  |  |  |  |  |  |  |  |  |  |  |  |  |  |  |  |
| Injuries | |  |  |  |  |  |  |  |  |  |  |  |  |  |  |  |  |
|  | Unintentional injuries | |  |  |  |  |  |  |  |  |  |  |  |  |  |  |  |
|  |  | Transport injuries | 0.00% | 0.00% | 0.00% |  | 7.20% | 4.34% | 10.07% |  | 5.28% | 2.67% | 7.88% |  | 2.64% | 1.28% | 4.01% |
|  |  | Poisonings | 0.00% | 0.00% | 0.00% |  | 10.21% | 4.17% | 16.26% |  | 10.37% | 4.11% | 16.63% |  | 6.53% | 2.34% | 10.71% |
|  |  | Falls | 0.00% | 0.00% | 0.00% |  | 10.21% | 4.17% | 16.26% |  | 10.37% | 4.11% | 16.63% |  | 6.53% | 2.34% | 10.71% |
|  |  | Fires, heat and hot substances | 0.00% | 0.00% | 0.00% |  | 10.21% | 4.17% | 16.26% |  | 10.37% | 4.11% | 16.63% |  | 6.53% | 2.34% | 10.71% |
|  |  | Drownings | 0.00% | 0.00% | 0.00% |  | 10.21% | 4.17% | 16.26% |  | 10.37% | 4.11% | 16.63% |  | 6.53% | 2.34% | 10.71% |
|  |  | Other unintentional injuries | 0.00% | 0.00% | 0.00% |  | 10.21% | 4.17% | 16.26% |  | 10.37% | 4.11% | 16.63% |  | 6.53% | 2.34% | 10.71% |
|  | Intentional injuries | |  |  |  |  |  |  |  |  |  |  |  |  |  |  |  |
|  |  | Self-inflicted injuries | 0.00% | 0.00% | 0.00% |  | 10.21% | 4.17% | 16.26% |  | 10.37% | 4.11% | 16.63% |  | 6.53% | 2.34% | 10.71% |
|  |  | Violence | 0.00% | 0.00% | 0.00% |  | 0.00% | 0.00% | 0.00% |  | 0.00% | 0.00% | 0.00% |  | 0.00% | 0.00% | 0.00% |
|  |  | Other intentional injuries | 0.00% | 0.00% | 0.00% |  | 10.21% | 4.17% | 16.26% |  | 10.37% | 4.11% | 16.63% |  | 6.53% | 2.34% | 10.71% |

Table 14: Morbidity Alcohol-Attributable Fractions for injuries (without harms to others included): Latin America Tropical

|  |  |  | 0 to 14 years of age | | |  | 15 to 34 years of age | | |  | 35 to 64 years of age | | |  | 65 years of age and older | | |
| --- | --- | --- | --- | --- | --- | --- | --- | --- | --- | --- | --- | --- | --- | --- | --- | --- | --- |
|  |  |  | Point estimate | Lower 95% CI | Upper 95% CI |  | Point estimate | Lower 95% CI | Upper 95% CI |  | Point estimate | Lower 95% CI | Upper 95% CI |  | Point estimate | Lower 95% CI | Upper 95% CI |
| Women | |  |  |  |  |  |  |  |  |  |  |  |  |  |  |  |  |
| Injuries | |  |  |  |  |  |  |  |  |  |  |  |  |  |  |  |  |
|  | Unintentional injuries | |  |  |  |  |  |  |  |  |  |  |  |  |  |  |  |
|  |  | Transport injuries | 0.00% | 0.00% | 0.00% |  | 4.11% | 2.07% | 6.16% |  | 3.27% | 0.70% | 5.83% |  | 0.36% | 0.16% | 0.55% |
|  |  | Poisonings | 0.00% | 0.00% | 0.00% |  | 2.83% | 0.00% | 6.06% |  | 4.20% | 0.00% | 9.13% |  | 0.37% | 0.01% | 0.73% |
|  |  | Falls | 0.00% | 0.00% | 0.00% |  | 2.83% | 0.00% | 6.06% |  | 4.20% | 0.00% | 9.13% |  | 0.37% | 0.01% | 0.73% |
|  |  | Fires, heat and hot substances | 0.00% | 0.00% | 0.00% |  | 2.83% | 0.00% | 6.06% |  | 4.20% | 0.00% | 9.13% |  | 0.37% | 0.01% | 0.73% |
|  |  | Drownings | 0.00% | 0.00% | 0.00% |  | 2.83% | 0.00% | 6.06% |  | 4.20% | 0.00% | 9.13% |  | 0.37% | 0.01% | 0.73% |
|  |  | Other unintentional injuries | 0.00% | 0.00% | 0.00% |  | 2.83% | 0.00% | 6.06% |  | 4.20% | 0.00% | 9.13% |  | 0.37% | 0.01% | 0.73% |
|  | Intentional injuries | |  |  |  |  |  |  |  |  |  |  |  |  |  |  |  |
|  |  | Self-inflicted injuries | 0.00% | 0.00% | 0.00% |  | 2.83% | 0.00% | 6.06% |  | 4.20% | 0.00% | 9.13% |  | 0.37% | 0.01% | 0.73% |
|  |  | Violence | 0.00% | 0.00% | 0.00% |  | 0.00% | 0.00% | 0.00% |  | 0.00% | 0.00% | 0.00% |  | 0.00% | 0.00% | 0.00% |
|  |  | Other intentional injuries | 0.00% | 0.00% | 0.00% |  | 2.83% | 0.00% | 6.06% |  | 4.20% | 0.00% | 9.13% |  | 0.37% | 0.01% | 0.73% |
| Men | |  |  |  |  |  |  |  |  |  |  |  |  |  |  |  |  |
| Injuries | |  |  |  |  |  |  |  |  |  |  |  |  |  |  |  |  |
|  | Unintentional injuries | |  |  |  |  |  |  |  |  |  |  |  |  |  |  |  |
|  |  | Transport injuries | 0.00% | 0.00% | 0.00% |  | 13.24% | 6.66% | 19.82% |  | 10.51% | 2.25% | 18.76% |  | 1.14% | 0.53% | 1.76% |
|  |  | Poisonings | 0.00% | 0.00% | 0.00% |  | 15.75% | 6.20% | 25.31% |  | 14.78% | 5.25% | 24.31% |  | 2.47% | 0.64% | 4.29% |
|  |  | Falls | 0.00% | 0.00% | 0.00% |  | 15.75% | 6.20% | 25.31% |  | 14.78% | 5.25% | 24.31% |  | 2.47% | 0.64% | 4.29% |
|  |  | Fires, heat and hot substances | 0.00% | 0.00% | 0.00% |  | 15.75% | 6.20% | 25.31% |  | 14.78% | 5.25% | 24.31% |  | 2.47% | 0.64% | 4.29% |
|  |  | Drownings | 0.00% | 0.00% | 0.00% |  | 15.75% | 6.20% | 25.31% |  | 14.78% | 5.25% | 24.31% |  | 2.47% | 0.64% | 4.29% |
|  |  | Other unintentional injuries | 0.00% | 0.00% | 0.00% |  | 15.75% | 6.20% | 25.31% |  | 14.78% | 5.25% | 24.31% |  | 2.47% | 0.64% | 4.29% |
|  | Intentional injuries | |  |  |  |  |  |  |  |  |  |  |  |  |  |  |  |
|  |  | Self-inflicted injuries | 0.00% | 0.00% | 0.00% |  | 15.75% | 6.20% | 25.31% |  | 14.78% | 5.25% | 24.31% |  | 2.47% | 0.64% | 4.29% |
|  |  | Violence | 0.00% | 0.00% | 0.00% |  | 0.00% | 0.00% | 0.00% |  | 0.00% | 0.00% | 0.00% |  | 0.00% | 0.00% | 0.00% |
|  |  | Other intentional injuries | 0.00% | 0.00% | 0.00% |  | 15.75% | 6.20% | 25.31% |  | 14.78% | 5.25% | 24.31% |  | 2.47% | 0.64% | 4.29% |

Table 15: Morbidity Alcohol-Attributable Fractions for injuries (without harms to others included): North Africa Middle East

|  |  |  | 0 to 14 years of age | | |  | 15 to 34 years of age | | |  | 35 to 64 years of age | | |  | 65 years of age and older | | |
| --- | --- | --- | --- | --- | --- | --- | --- | --- | --- | --- | --- | --- | --- | --- | --- | --- | --- |
|  |  |  | Point estimate | Lower 95% CI | Upper 95% CI |  | Point estimate | Lower 95% CI | Upper 95% CI |  | Point estimate | Lower 95% CI | Upper 95% CI |  | Point estimate | Lower 95% CI | Upper 95% CI |
| Women | |  |  |  |  |  |  |  |  |  |  |  |  |  |  |  |  |
| Injuries | |  |  |  |  |  |  |  |  |  |  |  |  |  |  |  |  |
|  | Unintentional injuries | |  |  |  |  |  |  |  |  |  |  |  |  |  |  |  |
|  |  | Transport injuries | 0.00% | 0.00% | 0.00% |  | 0.35% | 0.00% | 1.91% |  | 0.07% | 0.00% | 0.21% |  | 0.01% | 0.00% | 0.01% |
|  |  | Poisonings | 0.00% | 0.00% | 0.00% |  | 0.22% | 0.00% | 1.45% |  | 0.13% | 0.00% | 1.89% |  | 0.02% | 0.00% | 0.57% |
|  |  | Falls | 0.00% | 0.00% | 0.00% |  | 0.22% | 0.00% | 1.45% |  | 0.13% | 0.00% | 1.89% |  | 0.02% | 0.00% | 0.57% |
|  |  | Fires, heat and hot substances | 0.00% | 0.00% | 0.00% |  | 0.22% | 0.00% | 1.45% |  | 0.13% | 0.00% | 1.89% |  | 0.02% | 0.00% | 0.57% |
|  |  | Drownings | 0.00% | 0.00% | 0.00% |  | 0.22% | 0.00% | 1.45% |  | 0.13% | 0.00% | 1.89% |  | 0.02% | 0.00% | 0.57% |
|  |  | Other unintentional injuries | 0.00% | 0.00% | 0.00% |  | 0.22% | 0.00% | 1.45% |  | 0.13% | 0.00% | 1.89% |  | 0.02% | 0.00% | 0.57% |
|  | Intentional injuries | |  |  |  |  |  |  |  |  |  |  |  |  |  |  |  |
|  |  | Self-inflicted injuries | 0.00% | 0.00% | 0.00% |  | 0.22% | 0.00% | 1.45% |  | 0.13% | 0.00% | 1.89% |  | 0.02% | 0.00% | 0.57% |
|  |  | Violence | 0.00% | 0.00% | 0.00% |  | 0.00% | 0.00% | 0.00% |  | 0.00% | 0.00% | 0.00% |  | 0.00% | 0.00% | 0.00% |
|  |  | Other intentional injuries | 0.00% | 0.00% | 0.00% |  | 0.22% | 0.00% | 1.45% |  | 0.13% | 0.00% | 1.89% |  | 0.02% | 0.00% | 0.57% |
| Men | |  |  |  |  |  |  |  |  |  |  |  |  |  |  |  |  |
| Injuries | |  |  |  |  |  |  |  |  |  |  |  |  |  |  |  |  |
|  | Unintentional injuries | |  |  |  |  |  |  |  |  |  |  |  |  |  |  |  |
|  |  | Transport injuries | 0.00% | 0.00% | 0.00% |  | 2.69% | 0.00% | 14.83% |  | 0.54% | 0.00% | 1.62% |  | 0.06% | 0.01% | 0.10% |
|  |  | Poisonings | 0.00% | 0.00% | 0.00% |  | 3.51% | 0.07% | 6.96% |  | 1.45% | 0.00% | 3.11% |  | 0.04% | 0.01% | 0.07% |
|  |  | Falls | 0.00% | 0.00% | 0.00% |  | 3.51% | 0.07% | 6.96% |  | 1.45% | 0.00% | 3.11% |  | 0.04% | 0.01% | 0.07% |
|  |  | Fires, heat and hot substances | 0.00% | 0.00% | 0.00% |  | 3.51% | 0.07% | 6.96% |  | 1.45% | 0.00% | 3.11% |  | 0.04% | 0.01% | 0.07% |
|  |  | Drownings | 0.00% | 0.00% | 0.00% |  | 3.51% | 0.07% | 6.96% |  | 1.45% | 0.00% | 3.11% |  | 0.04% | 0.01% | 0.07% |
|  |  | Other unintentional injuries | 0.00% | 0.00% | 0.00% |  | 3.51% | 0.07% | 6.96% |  | 1.45% | 0.00% | 3.11% |  | 0.04% | 0.01% | 0.07% |
|  | Intentional injuries | |  |  |  |  |  |  |  |  |  |  |  |  |  |  |  |
|  |  | Self-inflicted injuries | 0.00% | 0.00% | 0.00% |  | 3.51% | 0.07% | 6.96% |  | 1.45% | 0.00% | 3.11% |  | 0.04% | 0.01% | 0.07% |
|  |  | Violence | 0.00% | 0.00% | 0.00% |  | 0.00% | 0.00% | 0.00% |  | 0.00% | 0.00% | 0.00% |  | 0.00% | 0.00% | 0.00% |
|  |  | Other intentional injuries | 0.00% | 0.00% | 0.00% |  | 3.51% | 0.07% | 6.96% |  | 1.45% | 0.00% | 3.11% |  | 0.04% | 0.01% | 0.07% |

Table 16: Morbidity Alcohol-Attributable Fractions for injuries (without harms to others included): North America High Income

|  |  |  | 0 to 14 years of age | | |  | 15 to 34 years of age | | |  | 35 to 64 years of age | | |  | 65 years of age and older | | |
| --- | --- | --- | --- | --- | --- | --- | --- | --- | --- | --- | --- | --- | --- | --- | --- | --- | --- |
|  |  |  | Point estimate | Lower 95% CI | Upper 95% CI |  | Point estimate | Lower 95% CI | Upper 95% CI |  | Point estimate | Lower 95% CI | Upper 95% CI |  | Point estimate | Lower 95% CI | Upper 95% CI |
| Women | |  |  |  |  |  |  |  |  |  |  |  |  |  |  |  |  |
| Injuries | |  |  |  |  |  |  |  |  |  |  |  |  |  |  |  |  |
|  | Unintentional injuries | |  |  |  |  |  |  |  |  |  |  |  |  |  |  |  |
|  |  | Transport injuries | 0.00% | 0.00% | 0.00% |  | 3.81% | 1.74% | 5.89% |  | 1.61% | 0.73% | 2.48% |  | 0.55% | 0.27% | 0.83% |
|  |  | Poisonings | 0.00% | 0.00% | 0.00% |  | 3.56% | 1.03% | 6.08% |  | 2.48% | 0.85% | 4.12% |  | 0.67% | 0.24% | 1.11% |
|  |  | Falls | 0.00% | 0.00% | 0.00% |  | 3.56% | 1.03% | 6.08% |  | 2.48% | 0.85% | 4.12% |  | 0.67% | 0.24% | 1.11% |
|  |  | Fires, heat and hot substances | 0.00% | 0.00% | 0.00% |  | 3.56% | 1.03% | 6.08% |  | 2.48% | 0.85% | 4.12% |  | 0.67% | 0.24% | 1.11% |
|  |  | Drownings | 0.00% | 0.00% | 0.00% |  | 3.56% | 1.03% | 6.08% |  | 2.48% | 0.85% | 4.12% |  | 0.67% | 0.24% | 1.11% |
|  |  | Other unintentional injuries | 0.00% | 0.00% | 0.00% |  | 3.56% | 1.03% | 6.08% |  | 2.48% | 0.85% | 4.12% |  | 0.67% | 0.24% | 1.11% |
|  | Intentional injuries | |  |  |  |  |  |  |  |  |  |  |  |  |  |  |  |
|  |  | Self-inflicted injuries | 0.00% | 0.00% | 0.00% |  | 3.56% | 1.03% | 6.08% |  | 2.48% | 0.85% | 4.12% |  | 0.67% | 0.24% | 1.11% |
|  |  | Violence | 0.00% | 0.00% | 0.00% |  | 0.00% | 0.00% | 0.00% |  | 0.00% | 0.00% | 0.00% |  | 0.00% | 0.00% | 0.00% |
|  |  | Other intentional injuries | 0.00% | 0.00% | 0.00% |  | 3.56% | 1.03% | 6.08% |  | 2.48% | 0.85% | 4.12% |  | 0.67% | 0.24% | 1.11% |
| Men | |  |  |  |  |  |  |  |  |  |  |  |  |  |  |  |  |
| Injuries | |  |  |  |  |  |  |  |  |  |  |  |  |  |  |  |  |
|  | Unintentional injuries | |  |  |  |  |  |  |  |  |  |  |  |  |  |  |  |
|  |  | Transport injuries | 0.00% | 0.00% | 0.00% |  | 10.85% | 4.94% | 16.77% |  | 4.57% | 2.09% | 7.06% |  | 1.57% | 0.78% | 2.37% |
|  |  | Poisonings | 0.00% | 0.00% | 0.00% |  | 17.04% | 7.87% | 26.20% |  | 10.35% | 4.44% | 16.26% |  | 4.58% | 1.71% | 7.44% |
|  |  | Falls | 0.00% | 0.00% | 0.00% |  | 17.04% | 7.87% | 26.20% |  | 10.35% | 4.44% | 16.26% |  | 4.58% | 1.71% | 7.44% |
|  |  | Fires, heat and hot substances | 0.00% | 0.00% | 0.00% |  | 17.04% | 7.87% | 26.20% |  | 10.35% | 4.44% | 16.26% |  | 4.58% | 1.71% | 7.44% |
|  |  | Drownings | 0.00% | 0.00% | 0.00% |  | 17.04% | 7.87% | 26.20% |  | 10.35% | 4.44% | 16.26% |  | 4.58% | 1.71% | 7.44% |
|  |  | Other unintentional injuries | 0.00% | 0.00% | 0.00% |  | 17.04% | 7.87% | 26.20% |  | 10.35% | 4.44% | 16.26% |  | 4.58% | 1.71% | 7.44% |
|  | Intentional injuries | |  |  |  |  |  |  |  |  |  |  |  |  |  |  |  |
|  |  | Self-inflicted injuries | 0.00% | 0.00% | 0.00% |  | 17.04% | 7.87% | 26.20% |  | 10.35% | 4.44% | 16.26% |  | 4.58% | 1.71% | 7.44% |
|  |  | Violence | 0.00% | 0.00% | 0.00% |  | 0.00% | 0.00% | 0.00% |  | 0.00% | 0.00% | 0.00% |  | 0.00% | 0.00% | 0.00% |
|  |  | Other intentional injuries | 0.00% | 0.00% | 0.00% |  | 17.04% | 7.87% | 26.20% |  | 10.35% | 4.44% | 16.26% |  | 4.58% | 1.71% | 7.44% |

Table 17: Morbidity Alcohol-Attributable Fractions for injuries (without harms to others included): Oceania

|  |  |  | 0 to 14 years of age | | |  | 15 to 34 years of age | | |  | 35 to 64 years of age | | |  | 65 years of age and older | | |
| --- | --- | --- | --- | --- | --- | --- | --- | --- | --- | --- | --- | --- | --- | --- | --- | --- | --- |
|  |  |  | Point estimate | Lower 95% CI | Upper 95% CI |  | Point estimate | Lower 95% CI | Upper 95% CI |  | Point estimate | Lower 95% CI | Upper 95% CI |  | Point estimate | Lower 95% CI | Upper 95% CI |
| Women | |  |  |  |  |  |  |  |  |  |  |  |  |  |  |  |  |
| Injuries | |  |  |  |  |  |  |  |  |  |  |  |  |  |  |  |  |
|  | Unintentional injuries | |  |  |  |  |  |  |  |  |  |  |  |  |  |  |  |
|  |  | Transport injuries | 0.00% | 0.00% | 0.00% |  | 1.80% | 1.14% | 2.46% |  | 1.40% | 0.88% | 1.93% |  | 0.66% | 0.38% | 0.93% |
|  |  | Poisonings | 0.00% | 0.00% | 0.00% |  | 1.48% | 0.55% | 2.42% |  | 0.81% | 0.20% | 1.41% |  | 0.37% | 0.09% | 0.64% |
|  |  | Falls | 0.00% | 0.00% | 0.00% |  | 1.48% | 0.55% | 2.42% |  | 0.81% | 0.20% | 1.41% |  | 0.37% | 0.09% | 0.64% |
|  |  | Fires, heat and hot substances | 0.00% | 0.00% | 0.00% |  | 1.48% | 0.55% | 2.42% |  | 0.81% | 0.20% | 1.41% |  | 0.37% | 0.09% | 0.64% |
|  |  | Drownings | 0.00% | 0.00% | 0.00% |  | 1.48% | 0.55% | 2.42% |  | 0.81% | 0.20% | 1.41% |  | 0.37% | 0.09% | 0.64% |
|  |  | Other unintentional injuries | 0.00% | 0.00% | 0.00% |  | 1.48% | 0.55% | 2.42% |  | 0.81% | 0.20% | 1.41% |  | 0.37% | 0.09% | 0.64% |
|  | Intentional injuries | |  |  |  |  |  |  |  |  |  |  |  |  |  |  |  |
|  |  | Self-inflicted injuries | 0.00% | 0.00% | 0.00% |  | 1.48% | 0.55% | 2.42% |  | 0.81% | 0.20% | 1.41% |  | 0.37% | 0.09% | 0.64% |
|  |  | Violence | 0.00% | 0.00% | 0.00% |  | 0.00% | 0.00% | 0.00% |  | 0.00% | 0.00% | 0.00% |  | 0.00% | 0.00% | 0.00% |
|  |  | Other intentional injuries | 0.00% | 0.00% | 0.00% |  | 1.48% | 0.55% | 2.42% |  | 0.81% | 0.20% | 1.41% |  | 0.37% | 0.09% | 0.64% |
| Men | |  |  |  |  |  |  |  |  |  |  |  |  |  |  |  |  |
| Injuries | |  |  |  |  |  |  |  |  |  |  |  |  |  |  |  |  |
|  | Unintentional injuries | |  |  |  |  |  |  |  |  |  |  |  |  |  |  |  |
|  |  | Transport injuries | 0.00% | 0.00% | 0.00% |  | 10.61% | 6.74% | 14.48% |  | 8.27% | 5.16% | 11.38% |  | 3.87% | 2.26% | 5.47% |
|  |  | Poisonings | 0.00% | 0.00% | 0.00% |  | 5.03% | 2.39% | 7.67% |  | 4.58% | 2.14% | 7.02% |  | 2.26% | 1.03% | 3.48% |
|  |  | Falls | 0.00% | 0.00% | 0.00% |  | 5.03% | 2.39% | 7.67% |  | 4.58% | 2.14% | 7.02% |  | 2.26% | 1.03% | 3.48% |
|  |  | Fires, heat and hot substances | 0.00% | 0.00% | 0.00% |  | 5.03% | 2.39% | 7.67% |  | 4.58% | 2.14% | 7.02% |  | 2.26% | 1.03% | 3.48% |
|  |  | Drownings | 0.00% | 0.00% | 0.00% |  | 5.03% | 2.39% | 7.67% |  | 4.58% | 2.14% | 7.02% |  | 2.26% | 1.03% | 3.48% |
|  |  | Other unintentional injuries | 0.00% | 0.00% | 0.00% |  | 5.03% | 2.39% | 7.67% |  | 4.58% | 2.14% | 7.02% |  | 2.26% | 1.03% | 3.48% |
|  | Intentional injuries | |  |  |  |  |  |  |  |  |  |  |  |  |  |  |  |
|  |  | Self-inflicted injuries | 0.00% | 0.00% | 0.00% |  | 5.03% | 2.39% | 7.67% |  | 4.58% | 2.14% | 7.02% |  | 2.26% | 1.03% | 3.48% |
|  |  | Violence | 0.00% | 0.00% | 0.00% |  | 0.00% | 0.00% | 0.00% |  | 0.00% | 0.00% | 0.00% |  | 0.00% | 0.00% | 0.00% |
|  |  | Other intentional injuries | 0.00% | 0.00% | 0.00% |  | 5.03% | 2.39% | 7.67% |  | 4.58% | 2.14% | 7.02% |  | 2.26% | 1.03% | 3.48% |

Table 18: Morbidity Alcohol-Attributable Fractions for injuries (without harms to others included): Sub-Saharan Africa Central

|  |  |  | 0 to 14 years of age | | |  | 15 to 34 years of age | | |  | 35 to 64 years of age | | |  | 65 years of age and older | | |
| --- | --- | --- | --- | --- | --- | --- | --- | --- | --- | --- | --- | --- | --- | --- | --- | --- | --- |
|  |  |  | Point estimate | Lower 95% CI | Upper 95% CI |  | Point estimate | Lower 95% CI | Upper 95% CI |  | Point estimate | Lower 95% CI | Upper 95% CI |  | Point estimate | Lower 95% CI | Upper 95% CI |
| Women | |  |  |  |  |  |  |  |  |  |  |  |  |  |  |  |  |
| Injuries | |  |  |  |  |  |  |  |  |  |  |  |  |  |  |  |  |
|  | Unintentional injuries | |  |  |  |  |  |  |  |  |  |  |  |  |  |  |  |
|  |  | Transport injuries | 0.00% | 0.00% | 0.00% |  | 2.91% | 1.82% | 3.99% |  | 3.30% | 2.09% | 4.52% |  | 0.92% | 0.52% | 1.31% |
|  |  | Poisonings | 0.00% | 0.00% | 0.00% |  | 1.82% | 0.63% | 3.01% |  | 1.89% | 0.37% | 3.41% |  | 0.41% | 0.11% | 0.71% |
|  |  | Falls | 0.00% | 0.00% | 0.00% |  | 1.82% | 0.63% | 3.01% |  | 1.89% | 0.37% | 3.41% |  | 0.41% | 0.11% | 0.71% |
|  |  | Fires, heat and hot substances | 0.00% | 0.00% | 0.00% |  | 1.82% | 0.63% | 3.01% |  | 1.89% | 0.37% | 3.41% |  | 0.41% | 0.11% | 0.71% |
|  |  | Drownings | 0.00% | 0.00% | 0.00% |  | 1.82% | 0.63% | 3.01% |  | 1.89% | 0.37% | 3.41% |  | 0.41% | 0.11% | 0.71% |
|  |  | Other unintentional injuries | 0.00% | 0.00% | 0.00% |  | 1.82% | 0.63% | 3.01% |  | 1.89% | 0.37% | 3.41% |  | 0.41% | 0.11% | 0.71% |
|  | Intentional injuries | |  |  |  |  |  |  |  |  |  |  |  |  |  |  |  |
|  |  | Self-inflicted injuries | 0.00% | 0.00% | 0.00% |  | 1.82% | 0.63% | 3.01% |  | 1.89% | 0.37% | 3.41% |  | 0.41% | 0.11% | 0.71% |
|  |  | Violence | 0.00% | 0.00% | 0.00% |  | 0.00% | 0.00% | 0.00% |  | 0.00% | 0.00% | 0.00% |  | 0.00% | 0.00% | 0.00% |
|  |  | Other intentional injuries | 0.00% | 0.00% | 0.00% |  | 1.82% | 0.63% | 3.01% |  | 1.89% | 0.37% | 3.41% |  | 0.41% | 0.11% | 0.71% |
| Men | |  |  |  |  |  |  |  |  |  |  |  |  |  |  |  |  |
| Injuries | |  |  |  |  |  |  |  |  |  |  |  |  |  |  |  |  |
|  | Unintentional injuries | |  |  |  |  |  |  |  |  |  |  |  |  |  |  |  |
|  |  | Transport injuries | 0.00% | 0.00% | 0.00% |  | 7.78% | 4.88% | 10.68% |  | 8.84% | 5.59% | 12.10% |  | 2.45% | 1.40% | 3.50% |
|  |  | Poisonings | 0.00% | 0.00% | 0.00% |  | 4.62% | 2.03% | 7.20% |  | 5.33% | 2.24% | 8.43% |  | 1.39% | 0.59% | 2.19% |
|  |  | Falls | 0.00% | 0.00% | 0.00% |  | 4.62% | 2.03% | 7.20% |  | 5.33% | 2.24% | 8.43% |  | 1.39% | 0.59% | 2.19% |
|  |  | Fires, heat and hot substances | 0.00% | 0.00% | 0.00% |  | 4.62% | 2.03% | 7.20% |  | 5.33% | 2.24% | 8.43% |  | 1.39% | 0.59% | 2.19% |
|  |  | Drownings | 0.00% | 0.00% | 0.00% |  | 4.62% | 2.03% | 7.20% |  | 5.33% | 2.24% | 8.43% |  | 1.39% | 0.59% | 2.19% |
|  |  | Other unintentional injuries | 0.00% | 0.00% | 0.00% |  | 4.62% | 2.03% | 7.20% |  | 5.33% | 2.24% | 8.43% |  | 1.39% | 0.59% | 2.19% |
|  | Intentional injuries | |  |  |  |  |  |  |  |  |  |  |  |  |  |  |  |
|  |  | Self-inflicted injuries | 0.00% | 0.00% | 0.00% |  | 4.62% | 2.03% | 7.20% |  | 5.33% | 2.24% | 8.43% |  | 1.39% | 0.59% | 2.19% |
|  |  | Violence | 0.00% | 0.00% | 0.00% |  | 0.00% | 0.00% | 0.00% |  | 0.00% | 0.00% | 0.00% |  | 0.00% | 0.00% | 0.00% |
|  |  | Other intentional injuries | 0.00% | 0.00% | 0.00% |  | 4.62% | 2.03% | 7.20% |  | 5.33% | 2.24% | 8.43% |  | 1.39% | 0.59% | 2.19% |

Table 19: Morbidity Alcohol-Attributable Fractions for injuries (without harms to others included): Sub-Saharan Africa East

|  |  |  | 0 to 14 years of age | | |  | 15 to 34 years of age | | |  | 35 to 64 years of age | | |  | 65 years of age and older | | |
| --- | --- | --- | --- | --- | --- | --- | --- | --- | --- | --- | --- | --- | --- | --- | --- | --- | --- |
|  |  |  | Point estimate | Lower 95% CI | Upper 95% CI |  | Point estimate | Lower 95% CI | Upper 95% CI |  | Point estimate | Lower 95% CI | Upper 95% CI |  | Point estimate | Lower 95% CI | Upper 95% CI |
| Women | |  |  |  |  |  |  |  |  |  |  |  |  |  |  |  |  |
| Injuries | |  |  |  |  |  |  |  |  |  |  |  |  |  |  |  |  |
|  | Unintentional injuries | |  |  |  |  |  |  |  |  |  |  |  |  |  |  |  |
|  |  | Transport injuries | 0.00% | 0.00% | 0.00% |  | 1.12% | 0.47% | 1.78% |  | 3.76% | 0.00% | 7.70% |  | 0.96% | 0.39% | 1.52% |
|  |  | Poisonings | 0.00% | 0.00% | 0.00% |  | 0.89% | 0.00% | 2.37% |  | 2.84% | 0.00% | 7.12% |  | 0.81% | 0.00% | 2.05% |
|  |  | Falls | 0.00% | 0.00% | 0.00% |  | 0.89% | 0.00% | 2.37% |  | 2.84% | 0.00% | 7.12% |  | 0.81% | 0.00% | 2.05% |
|  |  | Fires, heat and hot substances | 0.00% | 0.00% | 0.00% |  | 0.89% | 0.00% | 2.37% |  | 2.84% | 0.00% | 7.12% |  | 0.81% | 0.00% | 2.05% |
|  |  | Drownings | 0.00% | 0.00% | 0.00% |  | 0.89% | 0.00% | 2.37% |  | 2.84% | 0.00% | 7.12% |  | 0.81% | 0.00% | 2.05% |
|  |  | Other unintentional injuries | 0.00% | 0.00% | 0.00% |  | 0.89% | 0.00% | 2.37% |  | 2.84% | 0.00% | 7.12% |  | 0.81% | 0.00% | 2.05% |
|  | Intentional injuries | |  |  |  |  |  |  |  |  |  |  |  |  |  |  |  |
|  |  | Self-inflicted injuries | 0.00% | 0.00% | 0.00% |  | 0.89% | 0.00% | 2.37% |  | 2.84% | 0.00% | 7.12% |  | 0.81% | 0.00% | 2.05% |
|  |  | Violence | 0.00% | 0.00% | 0.00% |  | 0.00% | 0.00% | 0.00% |  | 0.00% | 0.00% | 0.00% |  | 0.00% | 0.00% | 0.00% |
|  |  | Other intentional injuries | 0.00% | 0.00% | 0.00% |  | 0.89% | 0.00% | 2.37% |  | 2.84% | 0.00% | 7.12% |  | 0.81% | 0.00% | 2.05% |
| Men | |  |  |  |  |  |  |  |  |  |  |  |  |  |  |  |  |
| Injuries | |  |  |  |  |  |  |  |  |  |  |  |  |  |  |  |  |
|  | Unintentional injuries | |  |  |  |  |  |  |  |  |  |  |  |  |  |  |  |
|  |  | Transport injuries | 0.00% | 0.00% | 0.00% |  | 3.77% | 1.58% | 5.97% |  | 12.65% | 0.00% | 25.87% |  | 3.22% | 1.32% | 5.12% |
|  |  | Poisonings | 0.00% | 0.00% | 0.00% |  | 5.08% | 1.30% | 8.86% |  | 14.88% | 4.41% | 25.36% |  | 5.42% | 1.32% | 9.53% |
|  |  | Falls | 0.00% | 0.00% | 0.00% |  | 5.08% | 1.30% | 8.86% |  | 14.88% | 4.41% | 25.36% |  | 5.42% | 1.32% | 9.53% |
|  |  | Fires, heat and hot substances | 0.00% | 0.00% | 0.00% |  | 5.08% | 1.30% | 8.86% |  | 14.88% | 4.41% | 25.36% |  | 5.42% | 1.32% | 9.53% |
|  |  | Drownings | 0.00% | 0.00% | 0.00% |  | 5.08% | 1.30% | 8.86% |  | 14.88% | 4.41% | 25.36% |  | 5.42% | 1.32% | 9.53% |
|  |  | Other unintentional injuries | 0.00% | 0.00% | 0.00% |  | 5.08% | 1.30% | 8.86% |  | 14.88% | 4.41% | 25.36% |  | 5.42% | 1.32% | 9.53% |
|  | Intentional injuries | |  |  |  |  |  |  |  |  |  |  |  |  |  |  |  |
|  |  | Self-inflicted injuries | 0.00% | 0.00% | 0.00% |  | 5.08% | 1.30% | 8.86% |  | 14.88% | 4.41% | 25.36% |  | 5.42% | 1.32% | 9.53% |
|  |  | Violence | 0.00% | 0.00% | 0.00% |  | 0.00% | 0.00% | 0.00% |  | 0.00% | 0.00% | 0.00% |  | 0.00% | 0.00% | 0.00% |
|  |  | Other intentional injuries | 0.00% | 0.00% | 0.00% |  | 5.08% | 1.30% | 8.86% |  | 14.88% | 4.41% | 25.36% |  | 5.42% | 1.32% | 9.53% |

Table 20: Morbidity Alcohol-Attributable Fractions for injuries (without harms to others included): Sub-Saharan Africa Southern

|  |  |  | 0 to 14 years of age | | |  | 15 to 34 years of age | | |  | 35 to 64 years of age | | |  | 65 years of age and older | | |
| --- | --- | --- | --- | --- | --- | --- | --- | --- | --- | --- | --- | --- | --- | --- | --- | --- | --- |
|  |  |  | Point estimate | Lower 95% CI | Upper 95% CI |  | Point estimate | Lower 95% CI | Upper 95% CI |  | Point estimate | Lower 95% CI | Upper 95% CI |  | Point estimate | Lower 95% CI | Upper 95% CI |
| Women | |  |  |  |  |  |  |  |  |  |  |  |  |  |  |  |  |
| Injuries | |  |  |  |  |  |  |  |  |  |  |  |  |  |  |  |  |
|  | Unintentional injuries | |  |  |  |  |  |  |  |  |  |  |  |  |  |  |  |
|  |  | Transport injuries | 0.00% | 0.00% | 0.00% |  | 8.03% | 3.84% | 12.23% |  | 7.68% | 3.26% | 12.10% |  | 0.95% | 0.45% | 1.45% |
|  |  | Poisonings | 0.00% | 0.00% | 0.00% |  | 5.78% | 0.00% | 13.28% |  | 4.57% | 0.00% | 10.57% |  | 0.83% | 0.00% | 1.81% |
|  |  | Falls | 0.00% | 0.00% | 0.00% |  | 5.78% | 0.00% | 13.28% |  | 4.57% | 0.00% | 10.57% |  | 0.83% | 0.00% | 1.81% |
|  |  | Fires, heat and hot substances | 0.00% | 0.00% | 0.00% |  | 5.78% | 0.00% | 13.28% |  | 4.57% | 0.00% | 10.57% |  | 0.83% | 0.00% | 1.81% |
|  |  | Drownings | 0.00% | 0.00% | 0.00% |  | 5.78% | 0.00% | 13.28% |  | 4.57% | 0.00% | 10.57% |  | 0.83% | 0.00% | 1.81% |
|  |  | Other unintentional injuries | 0.00% | 0.00% | 0.00% |  | 5.78% | 0.00% | 13.28% |  | 4.57% | 0.00% | 10.57% |  | 0.83% | 0.00% | 1.81% |
|  | Intentional injuries | |  |  |  |  |  |  |  |  |  |  |  |  |  |  |  |
|  |  | Self-inflicted injuries | 0.00% | 0.00% | 0.00% |  | 5.78% | 0.00% | 13.28% |  | 4.57% | 0.00% | 10.57% |  | 0.83% | 0.00% | 1.81% |
|  |  | Violence | 0.00% | 0.00% | 0.00% |  | 0.00% | 0.00% | 0.00% |  | 0.00% | 0.00% | 0.00% |  | 0.00% | 0.00% | 0.00% |
|  |  | Other intentional injuries | 0.00% | 0.00% | 0.00% |  | 5.78% | 0.00% | 13.28% |  | 4.57% | 0.00% | 10.57% |  | 0.83% | 0.00% | 1.81% |
| Men | |  |  |  |  |  |  |  |  |  |  |  |  |  |  |  |  |
| Injuries | |  |  |  |  |  |  |  |  |  |  |  |  |  |  |  |  |
|  | Unintentional injuries | |  |  |  |  |  |  |  |  |  |  |  |  |  |  |  |
|  |  | Transport injuries | 0.00% | 0.00% | 0.00% |  | 37.33% | 17.85% | 56.82% |  | 35.70% | 15.17% | 56.24% |  | 4.41% | 2.09% | 6.73% |
|  |  | Poisonings | 0.00% | 0.00% | 0.00% |  | 22.82% | 9.68% | 35.96% |  | 22.73% | 9.46% | 36.01% |  | 5.13% | 1.57% | 8.70% |
|  |  | Falls | 0.00% | 0.00% | 0.00% |  | 22.82% | 9.68% | 35.96% |  | 22.73% | 9.46% | 36.01% |  | 5.13% | 1.57% | 8.70% |
|  |  | Fires, heat and hot substances | 0.00% | 0.00% | 0.00% |  | 22.82% | 9.68% | 35.96% |  | 22.73% | 9.46% | 36.01% |  | 5.13% | 1.57% | 8.70% |
|  |  | Drownings | 0.00% | 0.00% | 0.00% |  | 22.82% | 9.68% | 35.96% |  | 22.73% | 9.46% | 36.01% |  | 5.13% | 1.57% | 8.70% |
|  |  | Other unintentional injuries | 0.00% | 0.00% | 0.00% |  | 22.82% | 9.68% | 35.96% |  | 22.73% | 9.46% | 36.01% |  | 5.13% | 1.57% | 8.70% |
|  | Intentional injuries | |  |  |  |  |  |  |  |  |  |  |  |  |  |  |  |
|  |  | Self-inflicted injuries | 0.00% | 0.00% | 0.00% |  | 22.82% | 9.68% | 35.96% |  | 22.73% | 9.46% | 36.01% |  | 5.13% | 1.57% | 8.70% |
|  |  | Violence | 0.00% | 0.00% | 0.00% |  | 0.00% | 0.00% | 0.00% |  | 0.00% | 0.00% | 0.00% |  | 0.00% | 0.00% | 0.00% |
|  |  | Other intentional injuries | 0.00% | 0.00% | 0.00% |  | 22.82% | 9.68% | 35.96% |  | 22.73% | 9.46% | 36.01% |  | 5.13% | 1.57% | 8.70% |

Table 21: Morbidity Alcohol-Attributable Fractions for injuries (without harms to others included): Sub-Saharan Africa West

|  |  |  | 0 to 14 years of age | | |  | 15 to 34 years of age | | |  | 35 to 64 years of age | | |  | 65 years of age and older | | |
| --- | --- | --- | --- | --- | --- | --- | --- | --- | --- | --- | --- | --- | --- | --- | --- | --- | --- |
|  |  |  | Point estimate | Lower 95% CI | Upper 95% CI |  | Point estimate | Lower 95% CI | Upper 95% CI |  | Point estimate | Lower 95% CI | Upper 95% CI |  | Point estimate | Lower 95% CI | Upper 95% CI |
| Women | |  |  |  |  |  |  |  |  |  |  |  |  |  |  |  |  |
| Injuries | |  |  |  |  |  |  |  |  |  |  |  |  |  |  |  |  |
|  | Unintentional injuries | |  |  |  |  |  |  |  |  |  |  |  |  |  |  |  |
|  |  | Transport injuries | 0.00% | 0.00% | 0.00% |  | 1.44% | 0.52% | 2.35% |  | 3.80% | 0.34% | 7.26% |  | 0.92% | 0.59% | 1.26% |
|  |  | Poisonings | 0.00% | 0.00% | 0.00% |  | 1.96% | 0.04% | 3.87% |  | 6.10% | 0.62% | 11.57% |  | 1.57% | 0.38% | 2.77% |
|  |  | Falls | 0.00% | 0.00% | 0.00% |  | 1.96% | 0.04% | 3.87% |  | 6.10% | 0.62% | 11.57% |  | 1.57% | 0.38% | 2.77% |
|  |  | Fires, heat and hot substances | 0.00% | 0.00% | 0.00% |  | 1.96% | 0.04% | 3.87% |  | 6.10% | 0.62% | 11.57% |  | 1.57% | 0.38% | 2.77% |
|  |  | Drownings | 0.00% | 0.00% | 0.00% |  | 1.96% | 0.04% | 3.87% |  | 6.10% | 0.62% | 11.57% |  | 1.57% | 0.38% | 2.77% |
|  |  | Other unintentional injuries | 0.00% | 0.00% | 0.00% |  | 1.96% | 0.04% | 3.87% |  | 6.10% | 0.62% | 11.57% |  | 1.57% | 0.38% | 2.77% |
|  | Intentional injuries | |  |  |  |  |  |  |  |  |  |  |  |  |  |  |  |
|  |  | Self-inflicted injuries | 0.00% | 0.00% | 0.00% |  | 1.96% | 0.04% | 3.87% |  | 6.10% | 0.62% | 11.57% |  | 1.57% | 0.38% | 2.77% |
|  |  | Violence | 0.00% | 0.00% | 0.00% |  | 0.00% | 0.00% | 0.00% |  | 0.00% | 0.00% | 0.00% |  | 0.00% | 0.00% | 0.00% |
|  |  | Other intentional injuries | 0.00% | 0.00% | 0.00% |  | 1.96% | 0.04% | 3.87% |  | 6.10% | 0.62% | 11.57% |  | 1.57% | 0.38% | 2.77% |
| Men | |  |  |  |  |  |  |  |  |  |  |  |  |  |  |  |  |
| Injuries | |  |  |  |  |  |  |  |  |  |  |  |  |  |  |  |  |
|  | Unintentional injuries | |  |  |  |  |  |  |  |  |  |  |  |  |  |  |  |
|  |  | Transport injuries | 0.00% | 0.00% | 0.00% |  | 4.26% | 1.54% | 6.98% |  | 11.26% | 1.01% | 21.52% |  | 2.74% | 1.74% | 3.74% |
|  |  | Poisonings | 0.00% | 0.00% | 0.00% |  | 7.70% | 2.67% | 12.73% |  | 16.43% | 6.28% | 26.59% |  | 4.17% | 1.51% | 6.83% |
|  |  | Falls | 0.00% | 0.00% | 0.00% |  | 7.70% | 2.67% | 12.73% |  | 16.43% | 6.28% | 26.59% |  | 4.17% | 1.51% | 6.83% |
|  |  | Fires, heat and hot substances | 0.00% | 0.00% | 0.00% |  | 7.70% | 2.67% | 12.73% |  | 16.43% | 6.28% | 26.59% |  | 4.17% | 1.51% | 6.83% |
|  |  | Drownings | 0.00% | 0.00% | 0.00% |  | 7.70% | 2.67% | 12.73% |  | 16.43% | 6.28% | 26.59% |  | 4.17% | 1.51% | 6.83% |
|  |  | Other unintentional injuries | 0.00% | 0.00% | 0.00% |  | 7.70% | 2.67% | 12.73% |  | 16.43% | 6.28% | 26.59% |  | 4.17% | 1.51% | 6.83% |
|  | Intentional injuries | |  |  |  |  |  |  |  |  |  |  |  |  |  |  |  |
|  |  | Self-inflicted injuries | 0.00% | 0.00% | 0.00% |  | 7.70% | 2.67% | 12.73% |  | 16.43% | 6.28% | 26.59% |  | 4.17% | 1.51% | 6.83% |
|  |  | Violence | 0.00% | 0.00% | 0.00% |  | 0.00% | 0.00% | 0.00% |  | 0.00% | 0.00% | 0.00% |  | 0.00% | 0.00% | 0.00% |
|  |  | Other intentional injuries | 0.00% | 0.00% | 0.00% |  | 7.70% | 2.67% | 12.73% |  | 16.43% | 6.28% | 26.59% |  | 4.17% | 1.51% | 6.83% |

Table 22: Mortality Alcohol-Attributable Fractions for injuries (without harms to others included): Asia Pacific [High Income]

|  |  |  | 0 to 14 years of age | | |  | 15 to 34 years of age | | |  | 35 to 64 years of age | | |  | 65 years of age and older | | |
| --- | --- | --- | --- | --- | --- | --- | --- | --- | --- | --- | --- | --- | --- | --- | --- | --- | --- |
|  |  |  | Point estimate | Lower 95% CI | Upper 95% CI |  | Point estimate | Lower 95% CI | Upper 95% CI |  | Point estimate | Lower 95% CI | Upper 95% CI |  | Point estimate | Lower 95% CI | Upper 95% CI |
| Women | |  |  |  |  |  |  |  |  |  |  |  |  |  |  |  |  |
| Injuries | |  |  |  |  |  |  |  |  |  |  |  |  |  |  |  |  |
|  | Unintentional injuries | |  |  |  |  |  |  |  |  |  |  |  |  |  |  |  |
|  |  | Transport injuries | 0.00% | 0.00% | 0.00% |  | 1.89% | 1.15% | 2.63% |  | 4.75% | 2.47% | 7.03% |  | 1.25% | 0.74% | 1.76% |
|  |  | Poisonings | 0.00% | 0.00% | 0.00% |  | 4.09% | 1.78% | 6.41% |  | 6.38% | 2.46% | 10.29% |  | 1.86% | 0.80% | 2.92% |
|  |  | Falls | 0.00% | 0.00% | 0.00% |  | 4.09% | 1.78% | 6.41% |  | 6.38% | 2.46% | 10.29% |  | 1.86% | 0.80% | 2.92% |
|  |  | Fires, heat and hot substances | 0.00% | 0.00% | 0.00% |  | 4.09% | 1.78% | 6.41% |  | 6.38% | 2.46% | 10.29% |  | 1.86% | 0.80% | 2.92% |
|  |  | Drownings | 0.00% | 0.00% | 0.00% |  | 4.09% | 1.78% | 6.41% |  | 6.38% | 2.46% | 10.29% |  | 1.86% | 0.80% | 2.92% |
|  |  | Other unintentional injuries | 0.00% | 0.00% | 0.00% |  | 4.09% | 1.78% | 6.41% |  | 6.38% | 2.46% | 10.29% |  | 1.86% | 0.80% | 2.92% |
|  | Intentional injuries | |  |  |  |  |  |  |  |  |  |  |  |  |  |  |  |
|  |  | Self-inflicted injuries | 0.00% | 0.00% | 0.00% |  | 4.09% | 1.78% | 6.41% |  | 6.38% | 2.46% | 10.29% |  | 1.86% | 0.80% | 2.92% |
|  |  | Violence | 0.00% | 0.00% | 0.00% |  | 0.00% | 0.00% | 0.00% |  | 0.00% | 0.00% | 0.00% |  | 0.00% | 0.00% | 0.00% |
|  |  | Other intentional injuries | 0.00% | 0.00% | 0.00% |  | 4.09% | 1.78% | 6.41% |  | 6.38% | 2.46% | 10.29% |  | 1.86% | 0.80% | 2.92% |
| Men | |  |  |  |  |  |  |  |  |  |  |  |  |  |  |  |  |
| Injuries | |  |  |  |  |  |  |  |  |  |  |  |  |  |  |  |  |
|  | Unintentional injuries | |  |  |  |  |  |  |  |  |  |  |  |  |  |  |  |
|  |  | Transport injuries | 0.00% | 0.00% | 0.00% |  | 6.22% | 3.78% | 8.66% |  | 15.63% | 8.13% | 23.14% |  | 4.12% | 2.43% | 5.80% |
|  |  | Poisonings | 0.00% | 0.00% | 0.00% |  | 16.49% | 6.90% | 26.08% |  | 37.47% | 17.84% | 57.09% |  | 12.55% | 5.04% | 20.06% |
|  |  | Falls | 0.00% | 0.00% | 0.00% |  | 16.49% | 6.90% | 26.08% |  | 37.47% | 17.84% | 57.09% |  | 12.55% | 5.04% | 20.06% |
|  |  | Fires, heat and hot substances | 0.00% | 0.00% | 0.00% |  | 16.49% | 6.90% | 26.08% |  | 37.47% | 17.84% | 57.09% |  | 12.55% | 5.04% | 20.06% |
|  |  | Drownings | 0.00% | 0.00% | 0.00% |  | 16.49% | 6.90% | 26.08% |  | 37.47% | 17.84% | 57.09% |  | 12.55% | 5.04% | 20.06% |
|  |  | Other unintentional injuries | 0.00% | 0.00% | 0.00% |  | 16.49% | 6.90% | 26.08% |  | 37.47% | 17.84% | 57.09% |  | 12.55% | 5.04% | 20.06% |
|  | Intentional injuries | |  |  |  |  |  |  |  |  |  |  |  |  |  |  |  |
|  |  | Self-inflicted injuries | 0.00% | 0.00% | 0.00% |  | 16.49% | 6.90% | 26.08% |  | 37.47% | 17.84% | 57.09% |  | 12.55% | 5.04% | 20.06% |
|  |  | Violence | 0.00% | 0.00% | 0.00% |  | 0.00% | 0.00% | 0.00% |  | 0.00% | 0.00% | 0.00% |  | 0.00% | 0.00% | 0.00% |
|  |  | Other intentional injuries | 0.00% | 0.00% | 0.00% |  | 16.49% | 6.90% | 26.08% |  | 37.47% | 17.84% | 57.09% |  | 12.55% | 5.04% | 20.06% |

Table 23: Mortality Alcohol-Attributable Fractions for injuries (without harms to others included): Asia Central

|  |  |  | 0 to 14 years of age | | |  | 15 to 34 years of age | | |  | 35 to 64 years of age | | |  | 65 years of age and older | | |
| --- | --- | --- | --- | --- | --- | --- | --- | --- | --- | --- | --- | --- | --- | --- | --- | --- | --- |
|  |  |  | Point estimate | Lower 95% CI | Upper 95% CI |  | Point estimate | Lower 95% CI | Upper 95% CI |  | Point estimate | Lower 95% CI | Upper 95% CI |  | Point estimate | Lower 95% CI | Upper 95% CI |
| Women | |  |  |  |  |  |  |  |  |  |  |  |  |  |  |  |  |
| Injuries | |  |  |  |  |  |  |  |  |  |  |  |  |  |  |  |  |
|  | Unintentional injuries | |  |  |  |  |  |  |  |  |  |  |  |  |  |  |  |
|  |  | Transport injuries | 0.00% | 0.00% | 0.00% |  | 6.08% | 4.00% | 8.16% |  | 9.02% | 6.19% | 11.85% |  | 4.60% | 2.94% | 6.26% |
|  |  | Poisonings | 0.00% | 0.00% | 0.00% |  | 3.15% | 0.37% | 5.92% |  | 7.58% | 1.07% | 14.09% |  | 0.88% | 0.00% | 1.81% |
|  |  | Falls | 0.00% | 0.00% | 0.00% |  | 3.15% | 0.37% | 5.92% |  | 7.58% | 1.07% | 14.09% |  | 0.88% | 0.00% | 1.81% |
|  |  | Fires, heat and hot substances | 0.00% | 0.00% | 0.00% |  | 3.15% | 0.37% | 5.92% |  | 7.58% | 1.07% | 14.09% |  | 0.88% | 0.00% | 1.81% |
|  |  | Drownings | 0.00% | 0.00% | 0.00% |  | 3.15% | 0.37% | 5.92% |  | 7.58% | 1.07% | 14.09% |  | 0.88% | 0.00% | 1.81% |
|  |  | Other unintentional injuries | 0.00% | 0.00% | 0.00% |  | 3.15% | 0.37% | 5.92% |  | 7.58% | 1.07% | 14.09% |  | 0.88% | 0.00% | 1.81% |
|  | Intentional injuries | |  |  |  |  |  |  |  |  |  |  |  |  |  |  |  |
|  |  | Self-inflicted injuries | 0.00% | 0.00% | 0.00% |  | 3.15% | 0.37% | 5.92% |  | 7.58% | 1.07% | 14.09% |  | 0.88% | 0.00% | 1.81% |
|  |  | Violence | 0.00% | 0.00% | 0.00% |  | 0.00% | 0.00% | 0.00% |  | 0.00% | 0.00% | 0.00% |  | 0.00% | 0.00% | 0.00% |
|  |  | Other intentional injuries | 0.00% | 0.00% | 0.00% |  | 3.15% | 0.37% | 5.92% |  | 7.58% | 1.07% | 14.09% |  | 0.88% | 0.00% | 1.81% |
| Men | |  |  |  |  |  |  |  |  |  |  |  |  |  |  |  |  |
| Injuries | |  |  |  |  |  |  |  |  |  |  |  |  |  |  |  |  |
|  | Unintentional injuries | |  |  |  |  |  |  |  |  |  |  |  |  |  |  |  |
|  |  | Transport injuries | 0.00% | 0.00% | 0.00% |  | 19.96% | 13.13% | 26.79% |  | 29.63% | 20.34% | 38.92% |  | 15.11% | 9.67% | 20.55% |
|  |  | Poisonings | 0.00% | 0.00% | 0.00% |  | 21.89% | 8.77% | 35.01% |  | 26.50% | 12.29% | 40.70% |  | 12.86% | 5.59% | 20.13% |
|  |  | Falls | 0.00% | 0.00% | 0.00% |  | 21.89% | 8.77% | 35.01% |  | 26.50% | 12.29% | 40.70% |  | 12.86% | 5.59% | 20.13% |
|  |  | Fires, heat and hot substances | 0.00% | 0.00% | 0.00% |  | 21.89% | 8.77% | 35.01% |  | 26.50% | 12.29% | 40.70% |  | 12.86% | 5.59% | 20.13% |
|  |  | Drownings | 0.00% | 0.00% | 0.00% |  | 21.89% | 8.77% | 35.01% |  | 26.50% | 12.29% | 40.70% |  | 12.86% | 5.59% | 20.13% |
|  |  | Other unintentional injuries | 0.00% | 0.00% | 0.00% |  | 21.89% | 8.77% | 35.01% |  | 26.50% | 12.29% | 40.70% |  | 12.86% | 5.59% | 20.13% |
|  | Intentional injuries | |  |  |  |  |  |  |  |  |  |  |  |  |  |  |  |
|  |  | Self-inflicted injuries | 0.00% | 0.00% | 0.00% |  | 21.89% | 8.77% | 35.01% |  | 26.50% | 12.29% | 40.70% |  | 12.86% | 5.59% | 20.13% |
|  |  | Violence | 0.00% | 0.00% | 0.00% |  | 0.00% | 0.00% | 0.00% |  | 0.00% | 0.00% | 0.00% |  | 0.00% | 0.00% | 0.00% |
|  |  | Other intentional injuries | 0.00% | 0.00% | 0.00% |  | 21.89% | 8.77% | 35.01% |  | 26.50% | 12.29% | 40.70% |  | 12.86% | 5.59% | 20.13% |

Table 24: Mortality Alcohol-Attributable Fractions for injuries (without harms to others included): Asia East

|  |  |  | 0 to 14 years of age | | |  | 15 to 34 years of age | | |  | 35 to 64 years of age | | |  | 65 years of age and older | | |
| --- | --- | --- | --- | --- | --- | --- | --- | --- | --- | --- | --- | --- | --- | --- | --- | --- | --- |
|  |  |  | Point estimate | Lower 95% CI | Upper 95% CI |  | Point estimate | Lower 95% CI | Upper 95% CI |  | Point estimate | Lower 95% CI | Upper 95% CI |  | Point estimate | Lower 95% CI | Upper 95% CI |
| Women | |  |  |  |  |  |  |  |  |  |  |  |  |  |  |  |  |
| Injuries | |  |  |  |  |  |  |  |  |  |  |  |  |  |  |  |  |
|  | Unintentional injuries | |  |  |  |  |  |  |  |  |  |  |  |  |  |  |  |
|  |  | Transport injuries | 0.00% | 0.00% | 0.00% |  | 0.54% | 0.31% | 0.77% |  | 1.98% | 0.89% | 3.06% |  | 0.76% | 0.37% | 1.16% |
|  |  | Poisonings | 0.00% | 0.00% | 0.00% |  | 0.60% | 0.09% | 1.11% |  | 2.98% | 0.00% | 6.80% |  | 1.21% | 0.00% | 2.46% |
|  |  | Falls | 0.00% | 0.00% | 0.00% |  | 0.60% | 0.09% | 1.11% |  | 2.98% | 0.00% | 6.80% |  | 1.21% | 0.00% | 2.46% |
|  |  | Fires, heat and hot substances | 0.00% | 0.00% | 0.00% |  | 0.60% | 0.09% | 1.11% |  | 2.98% | 0.00% | 6.80% |  | 1.21% | 0.00% | 2.46% |
|  |  | Drownings | 0.00% | 0.00% | 0.00% |  | 0.60% | 0.09% | 1.11% |  | 2.98% | 0.00% | 6.80% |  | 1.21% | 0.00% | 2.46% |
|  |  | Other unintentional injuries | 0.00% | 0.00% | 0.00% |  | 0.60% | 0.09% | 1.11% |  | 2.98% | 0.00% | 6.80% |  | 1.21% | 0.00% | 2.46% |
|  | Intentional injuries | |  |  |  |  |  |  |  |  |  |  |  |  |  |  |  |
|  |  | Self-inflicted injuries | 0.00% | 0.00% | 0.00% |  | 0.60% | 0.09% | 1.11% |  | 2.98% | 0.00% | 6.80% |  | 1.21% | 0.00% | 2.46% |
|  |  | Violence | 0.00% | 0.00% | 0.00% |  | 0.00% | 0.00% | 0.00% |  | 0.00% | 0.00% | 0.00% |  | 0.00% | 0.00% | 0.00% |
|  |  | Other intentional injuries | 0.00% | 0.00% | 0.00% |  | 0.60% | 0.09% | 1.11% |  | 2.98% | 0.00% | 6.80% |  | 1.21% | 0.00% | 2.46% |
| Men | |  |  |  |  |  |  |  |  |  |  |  |  |  |  |  |  |
| Injuries | |  |  |  |  |  |  |  |  |  |  |  |  |  |  |  |  |
|  | Unintentional injuries | |  |  |  |  |  |  |  |  |  |  |  |  |  |  |  |
|  |  | Transport injuries | 0.00% | 0.00% | 0.00% |  | 2.74% | 1.58% | 3.90% |  | 9.99% | 4.51% | 15.48% |  | 3.86% | 1.87% | 5.85% |
|  |  | Poisonings | 0.00% | 0.00% | 0.00% |  | 5.13% | 2.33% | 7.92% |  | 26.24% | 9.95% | 42.53% |  | 13.32% | 4.31% | 22.33% |
|  |  | Falls | 0.00% | 0.00% | 0.00% |  | 5.13% | 2.33% | 7.92% |  | 26.24% | 9.95% | 42.53% |  | 13.32% | 4.31% | 22.33% |
|  |  | Fires, heat and hot substances | 0.00% | 0.00% | 0.00% |  | 5.13% | 2.33% | 7.92% |  | 26.24% | 9.95% | 42.53% |  | 13.32% | 4.31% | 22.33% |
|  |  | Drownings | 0.00% | 0.00% | 0.00% |  | 5.13% | 2.33% | 7.92% |  | 26.24% | 9.95% | 42.53% |  | 13.32% | 4.31% | 22.33% |
|  |  | Other unintentional injuries | 0.00% | 0.00% | 0.00% |  | 5.13% | 2.33% | 7.92% |  | 26.24% | 9.95% | 42.53% |  | 13.32% | 4.31% | 22.33% |
|  | Intentional injuries | |  |  |  |  |  |  |  |  |  |  |  |  |  |  |  |
|  |  | Self-inflicted injuries | 0.00% | 0.00% | 0.00% |  | 5.13% | 2.33% | 7.92% |  | 26.24% | 9.95% | 42.53% |  | 13.32% | 4.31% | 22.33% |
|  |  | Violence | 0.00% | 0.00% | 0.00% |  | 0.00% | 0.00% | 0.00% |  | 0.00% | 0.00% | 0.00% |  | 0.00% | 0.00% | 0.00% |
|  |  | Other intentional injuries | 0.00% | 0.00% | 0.00% |  | 5.13% | 2.33% | 7.92% |  | 26.24% | 9.95% | 42.53% |  | 13.32% | 4.31% | 22.33% |

Table 25: Mortality Alcohol-Attributable Fractions for injuries (without harms to others included): Asia South

|  |  |  | 0 to 14 years of age | | |  | 15 to 34 years of age | | |  | 35 to 64 years of age | | |  | 65 years of age and older | | |
| --- | --- | --- | --- | --- | --- | --- | --- | --- | --- | --- | --- | --- | --- | --- | --- | --- | --- |
|  |  |  | Point estimate | Lower 95% CI | Upper 95% CI |  | Point estimate | Lower 95% CI | Upper 95% CI |  | Point estimate | Lower 95% CI | Upper 95% CI |  | Point estimate | Lower 95% CI | Upper 95% CI |
| Women | |  |  |  |  |  |  |  |  |  |  |  |  |  |  |  |  |
| Injuries | |  |  |  |  |  |  |  |  |  |  |  |  |  |  |  |  |
|  | Unintentional injuries | |  |  |  |  |  |  |  |  |  |  |  |  |  |  |  |
|  |  | Transport injuries | 0.00% | 0.00% | 0.00% |  | 0.29% | 0.14% | 0.44% |  | 0.76% | 0.19% | 1.32% |  | 0.26% | 0.00% | 0.52% |
|  |  | Poisonings | 0.00% | 0.00% | 0.00% |  | 0.10% | 0.00% | 2.62% |  | 1.07% | 0.00% | 19.70% |  | 0.03% | 0.00% | 2.79% |
|  |  | Falls | 0.00% | 0.00% | 0.00% |  | 0.10% | 0.00% | 2.62% |  | 1.07% | 0.00% | 19.70% |  | 0.03% | 0.00% | 2.79% |
|  |  | Fires, heat and hot substances | 0.00% | 0.00% | 0.00% |  | 0.10% | 0.00% | 2.62% |  | 1.07% | 0.00% | 19.70% |  | 0.03% | 0.00% | 2.79% |
|  |  | Drownings | 0.00% | 0.00% | 0.00% |  | 0.10% | 0.00% | 2.62% |  | 1.07% | 0.00% | 19.70% |  | 0.03% | 0.00% | 2.79% |
|  |  | Other unintentional injuries | 0.00% | 0.00% | 0.00% |  | 0.10% | 0.00% | 2.62% |  | 1.07% | 0.00% | 19.70% |  | 0.03% | 0.00% | 2.79% |
|  | Intentional injuries | |  |  |  |  |  |  |  |  |  |  |  |  |  |  |  |
|  |  | Self-inflicted injuries | 0.00% | 0.00% | 0.00% |  | 0.10% | 0.00% | 2.62% |  | 1.07% | 0.00% | 19.70% |  | 0.03% | 0.00% | 2.79% |
|  |  | Violence | 0.00% | 0.00% | 0.00% |  | 0.00% | 0.00% | 0.00% |  | 0.00% | 0.00% | 0.00% |  | 0.00% | 0.00% | 0.00% |
|  |  | Other intentional injuries | 0.00% | 0.00% | 0.00% |  | 0.10% | 0.00% | 2.62% |  | 1.07% | 0.00% | 19.70% |  | 0.03% | 0.00% | 2.79% |
| Men | |  |  |  |  |  |  |  |  |  |  |  |  |  |  |  |  |
| Injuries | |  |  |  |  |  |  |  |  |  |  |  |  |  |  |  |  |
|  | Unintentional injuries | |  |  |  |  |  |  |  |  |  |  |  |  |  |  |  |
|  |  | Transport injuries | 0.00% | 0.00% | 0.00% |  | 4.46% | 2.15% | 6.77% |  | 11.77% | 2.98% | 20.55% |  | 4.04% | 0.00% | 8.10% |
|  |  | Poisonings | 0.00% | 0.00% | 0.00% |  | 5.89% | 0.87% | 10.90% |  | 15.69% | 2.27% | 29.11% |  | 7.52% | 0.02% | 15.03% |
|  |  | Falls | 0.00% | 0.00% | 0.00% |  | 5.89% | 0.87% | 10.90% |  | 15.69% | 2.27% | 29.11% |  | 7.52% | 0.02% | 15.03% |
|  |  | Fires, heat and hot substances | 0.00% | 0.00% | 0.00% |  | 5.89% | 0.87% | 10.90% |  | 15.69% | 2.27% | 29.11% |  | 7.52% | 0.02% | 15.03% |
|  |  | Drownings | 0.00% | 0.00% | 0.00% |  | 5.89% | 0.87% | 10.90% |  | 15.69% | 2.27% | 29.11% |  | 7.52% | 0.02% | 15.03% |
|  |  | Other unintentional injuries | 0.00% | 0.00% | 0.00% |  | 5.89% | 0.87% | 10.90% |  | 15.69% | 2.27% | 29.11% |  | 7.52% | 0.02% | 15.03% |
|  | Intentional injuries | |  |  |  |  |  |  |  |  |  |  |  |  |  |  |  |
|  |  | Self-inflicted injuries | 0.00% | 0.00% | 0.00% |  | 5.89% | 0.87% | 10.90% |  | 15.69% | 2.27% | 29.11% |  | 7.52% | 0.02% | 15.03% |
|  |  | Violence | 0.00% | 0.00% | 0.00% |  | 0.00% | 0.00% | 0.00% |  | 0.00% | 0.00% | 0.00% |  | 0.00% | 0.00% | 0.00% |
|  |  | Other intentional injuries | 0.00% | 0.00% | 0.00% |  | 5.89% | 0.87% | 10.90% |  | 15.69% | 2.27% | 29.11% |  | 7.52% | 0.02% | 15.03% |

Table 26: Mortality Alcohol-Attributable Fractions for injuries (without harms to others included): Asia Southeast

|  |  |  | 0 to 14 years of age | | |  | 15 to 34 years of age | | |  | 35 to 64 years of age | | |  | 65 years of age and older | | |
| --- | --- | --- | --- | --- | --- | --- | --- | --- | --- | --- | --- | --- | --- | --- | --- | --- | --- |
|  |  |  | Point estimate | Lower 95% CI | Upper 95% CI |  | Point estimate | Lower 95% CI | Upper 95% CI |  | Point estimate | Lower 95% CI | Upper 95% CI |  | Point estimate | Lower 95% CI | Upper 95% CI |
| Women | |  |  |  |  |  |  |  |  |  |  |  |  |  |  |  |  |
| Injuries | |  |  |  |  |  |  |  |  |  |  |  |  |  |  |  |  |
|  | Unintentional injuries | |  |  |  |  |  |  |  |  |  |  |  |  |  |  |  |
|  |  | Transport injuries | 0.00% | 0.00% | 0.00% |  | 0.34% | 0.20% | 0.47% |  | 0.94% | 0.00% | 2.23% |  | 0.14% | 0.01% | 0.28% |
|  |  | Poisonings | 0.00% | 0.00% | 0.00% |  | 0.45% | 0.00% | 1.79% |  | 1.11% | 0.00% | 5.01% |  | 0.05% | 0.00% | 0.18% |
|  |  | Falls | 0.00% | 0.00% | 0.00% |  | 0.45% | 0.00% | 1.79% |  | 1.11% | 0.00% | 5.01% |  | 0.05% | 0.00% | 0.18% |
|  |  | Fires, heat and hot substances | 0.00% | 0.00% | 0.00% |  | 0.45% | 0.00% | 1.79% |  | 1.11% | 0.00% | 5.01% |  | 0.05% | 0.00% | 0.18% |
|  |  | Drownings | 0.00% | 0.00% | 0.00% |  | 0.45% | 0.00% | 1.79% |  | 1.11% | 0.00% | 5.01% |  | 0.05% | 0.00% | 0.18% |
|  |  | Other unintentional injuries | 0.00% | 0.00% | 0.00% |  | 0.45% | 0.00% | 1.79% |  | 1.11% | 0.00% | 5.01% |  | 0.05% | 0.00% | 0.18% |
|  | Intentional injuries | |  |  |  |  |  |  |  |  |  |  |  |  |  |  |  |
|  |  | Self-inflicted injuries | 0.00% | 0.00% | 0.00% |  | 0.45% | 0.00% | 1.79% |  | 1.11% | 0.00% | 5.01% |  | 0.05% | 0.00% | 0.18% |
|  |  | Violence | 0.00% | 0.00% | 0.00% |  | 0.00% | 0.00% | 0.00% |  | 0.00% | 0.00% | 0.00% |  | 0.00% | 0.00% | 0.00% |
|  |  | Other intentional injuries | 0.00% | 0.00% | 0.00% |  | 0.45% | 0.00% | 1.79% |  | 1.11% | 0.00% | 5.01% |  | 0.05% | 0.00% | 0.18% |
| Men | |  |  |  |  |  |  |  |  |  |  |  |  |  |  |  |  |
| Injuries | |  |  |  |  |  |  |  |  |  |  |  |  |  |  |  |  |
|  | Unintentional injuries | |  |  |  |  |  |  |  |  |  |  |  |  |  |  |  |
|  |  | Transport injuries | 0.00% | 0.00% | 0.00% |  | 3.72% | 2.20% | 5.25% |  | 10.41% | 0.00% | 24.73% |  | 1.58% | 0.06% | 3.10% |
|  |  | Poisonings | 0.00% | 0.00% | 0.00% |  | 5.26% | 1.58% | 8.93% |  | 21.78% | 5.74% | 37.82% |  | 6.69% | 1.39% | 11.98% |
|  |  | Falls | 0.00% | 0.00% | 0.00% |  | 5.26% | 1.58% | 8.93% |  | 21.78% | 5.74% | 37.82% |  | 6.69% | 1.39% | 11.98% |
|  |  | Fires, heat and hot substances | 0.00% | 0.00% | 0.00% |  | 5.26% | 1.58% | 8.93% |  | 21.78% | 5.74% | 37.82% |  | 6.69% | 1.39% | 11.98% |
|  |  | Drownings | 0.00% | 0.00% | 0.00% |  | 5.26% | 1.58% | 8.93% |  | 21.78% | 5.74% | 37.82% |  | 6.69% | 1.39% | 11.98% |
|  |  | Other unintentional injuries | 0.00% | 0.00% | 0.00% |  | 5.26% | 1.58% | 8.93% |  | 21.78% | 5.74% | 37.82% |  | 6.69% | 1.39% | 11.98% |
|  | Intentional injuries | |  |  |  |  |  |  |  |  |  |  |  |  |  |  |  |
|  |  | Self-inflicted injuries | 0.00% | 0.00% | 0.00% |  | 5.26% | 1.58% | 8.93% |  | 21.78% | 5.74% | 37.82% |  | 6.69% | 1.39% | 11.98% |
|  |  | Violence | 0.00% | 0.00% | 0.00% |  | 0.00% | 0.00% | 0.00% |  | 0.00% | 0.00% | 0.00% |  | 0.00% | 0.00% | 0.00% |
|  |  | Other intentional injuries | 0.00% | 0.00% | 0.00% |  | 5.26% | 1.58% | 8.93% |  | 21.78% | 5.74% | 37.82% |  | 6.69% | 1.39% | 11.98% |

Table 27: Mortality Alcohol-Attributable Fractions for injuries (without harms to others included): Australasia

|  |  |  | 0 to 14 years of age | | |  | 15 to 34 years of age | | |  | 35 to 64 years of age | | |  | 65 years of age and older | | |
| --- | --- | --- | --- | --- | --- | --- | --- | --- | --- | --- | --- | --- | --- | --- | --- | --- | --- |
|  |  |  | Point estimate | Lower 95% CI | Upper 95% CI |  | Point estimate | Lower 95% CI | Upper 95% CI |  | Point estimate | Lower 95% CI | Upper 95% CI |  | Point estimate | Lower 95% CI | Upper 95% CI |
| Women | |  |  |  |  |  |  |  |  |  |  |  |  |  |  |  |  |
| Injuries | |  |  |  |  |  |  |  |  |  |  |  |  |  |  |  |  |
|  | Unintentional injuries | |  |  |  |  |  |  |  |  |  |  |  |  |  |  |  |
|  |  | Transport injuries | 0.00% | 0.00% | 0.00% |  | 2.88% | 1.77% | 3.98% |  | 3.41% | 1.87% | 4.96% |  | 1.31% | 0.61% | 2.02% |
|  |  | Poisonings | 0.00% | 0.00% | 0.00% |  | 6.84% | 3.28% | 10.39% |  | 6.40% | 2.99% | 9.81% |  | 3.03% | 1.43% | 4.64% |
|  |  | Falls | 0.00% | 0.00% | 0.00% |  | 6.84% | 3.28% | 10.39% |  | 6.40% | 2.99% | 9.81% |  | 3.03% | 1.43% | 4.64% |
|  |  | Fires, heat and hot substances | 0.00% | 0.00% | 0.00% |  | 6.84% | 3.28% | 10.39% |  | 6.40% | 2.99% | 9.81% |  | 3.03% | 1.43% | 4.64% |
|  |  | Drownings | 0.00% | 0.00% | 0.00% |  | 6.84% | 3.28% | 10.39% |  | 6.40% | 2.99% | 9.81% |  | 3.03% | 1.43% | 4.64% |
|  |  | Other unintentional injuries | 0.00% | 0.00% | 0.00% |  | 6.84% | 3.28% | 10.39% |  | 6.40% | 2.99% | 9.81% |  | 3.03% | 1.43% | 4.64% |
|  | Intentional injuries | |  |  |  |  |  |  |  |  |  |  |  |  |  |  |  |
|  |  | Self-inflicted injuries | 0.00% | 0.00% | 0.00% |  | 6.84% | 3.28% | 10.39% |  | 6.40% | 2.99% | 9.81% |  | 3.03% | 1.43% | 4.64% |
|  |  | Violence | 0.00% | 0.00% | 0.00% |  | 0.00% | 0.00% | 0.00% |  | 0.00% | 0.00% | 0.00% |  | 0.00% | 0.00% | 0.00% |
|  |  | Other intentional injuries | 0.00% | 0.00% | 0.00% |  | 6.84% | 3.28% | 10.39% |  | 6.40% | 2.99% | 9.81% |  | 3.03% | 1.43% | 4.64% |
| Men | |  |  |  |  |  |  |  |  |  |  |  |  |  |  |  |  |
| Injuries | |  |  |  |  |  |  |  |  |  |  |  |  |  |  |  |  |
|  | Unintentional injuries | |  |  |  |  |  |  |  |  |  |  |  |  |  |  |  |
|  |  | Transport injuries | 0.00% | 0.00% | 0.00% |  | 7.11% | 4.38% | 9.84% |  | 8.44% | 4.61% | 12.26% |  | 3.25% | 1.51% | 4.99% |
|  |  | Poisonings | 0.00% | 0.00% | 0.00% |  | 19.41% | 9.05% | 29.76% |  | 27.08% | 13.03% | 41.12% |  | 16.39% | 7.26% | 25.53% |
|  |  | Falls | 0.00% | 0.00% | 0.00% |  | 19.41% | 9.05% | 29.76% |  | 27.08% | 13.03% | 41.12% |  | 16.39% | 7.26% | 25.53% |
|  |  | Fires, heat and hot substances | 0.00% | 0.00% | 0.00% |  | 19.41% | 9.05% | 29.76% |  | 27.08% | 13.03% | 41.12% |  | 16.39% | 7.26% | 25.53% |
|  |  | Drownings | 0.00% | 0.00% | 0.00% |  | 19.41% | 9.05% | 29.76% |  | 27.08% | 13.03% | 41.12% |  | 16.39% | 7.26% | 25.53% |
|  |  | Other unintentional injuries | 0.00% | 0.00% | 0.00% |  | 19.41% | 9.05% | 29.76% |  | 27.08% | 13.03% | 41.12% |  | 16.39% | 7.26% | 25.53% |
|  | Intentional injuries | |  |  |  |  |  |  |  |  |  |  |  |  |  |  |  |
|  |  | Self-inflicted injuries | 0.00% | 0.00% | 0.00% |  | 19.41% | 9.05% | 29.76% |  | 27.08% | 13.03% | 41.12% |  | 16.39% | 7.26% | 25.53% |
|  |  | Violence | 0.00% | 0.00% | 0.00% |  | 0.00% | 0.00% | 0.00% |  | 0.00% | 0.00% | 0.00% |  | 0.00% | 0.00% | 0.00% |
|  |  | Other intentional injuries | 0.00% | 0.00% | 0.00% |  | 19.41% | 9.05% | 29.76% |  | 27.08% | 13.03% | 41.12% |  | 16.39% | 7.26% | 25.53% |

Table 28: Mortality Alcohol-Attributable Fractions for injuries (without harms to others included): Caribbean

|  |  |  | 0 to 14 years of age | | |  | 15 to 34 years of age | | |  | 35 to 64 years of age | | |  | 65 years of age and older | | |
| --- | --- | --- | --- | --- | --- | --- | --- | --- | --- | --- | --- | --- | --- | --- | --- | --- | --- |
|  |  |  | Point estimate | Lower 95% CI | Upper 95% CI |  | Point estimate | Lower 95% CI | Upper 95% CI |  | Point estimate | Lower 95% CI | Upper 95% CI |  | Point estimate | Lower 95% CI | Upper 95% CI |
| Women | |  |  |  |  |  |  |  |  |  |  |  |  |  |  |  |  |
| Injuries | |  |  |  |  |  |  |  |  |  |  |  |  |  |  |  |  |
|  | Unintentional injuries | |  |  |  |  |  |  |  |  |  |  |  |  |  |  |  |
|  |  | Transport injuries | 0.00% | 0.00% | 0.00% |  | 2.10% | 1.29% | 2.91% |  | 2.22% | 1.32% | 3.12% |  | 0.63% | 0.33% | 0.94% |
|  |  | Poisonings | 0.00% | 0.00% | 0.00% |  | 5.71% | 0.74% | 10.68% |  | 1.56% | 0.52% | 2.61% |  | 0.24% | 0.06% | 0.42% |
|  |  | Falls | 0.00% | 0.00% | 0.00% |  | 5.71% | 0.74% | 10.68% |  | 1.56% | 0.52% | 2.61% |  | 0.24% | 0.06% | 0.42% |
|  |  | Fires, heat and hot substances | 0.00% | 0.00% | 0.00% |  | 5.71% | 0.74% | 10.68% |  | 1.56% | 0.52% | 2.61% |  | 0.24% | 0.06% | 0.42% |
|  |  | Drownings | 0.00% | 0.00% | 0.00% |  | 5.71% | 0.74% | 10.68% |  | 1.56% | 0.52% | 2.61% |  | 0.24% | 0.06% | 0.42% |
|  |  | Other unintentional injuries | 0.00% | 0.00% | 0.00% |  | 5.71% | 0.74% | 10.68% |  | 1.56% | 0.52% | 2.61% |  | 0.24% | 0.06% | 0.42% |
|  | Intentional injuries | |  |  |  |  |  |  |  |  |  |  |  |  |  |  |  |
|  |  | Self-inflicted injuries | 0.00% | 0.00% | 0.00% |  | 5.71% | 0.74% | 10.68% |  | 1.56% | 0.52% | 2.61% |  | 0.24% | 0.06% | 0.42% |
|  |  | Violence | 0.00% | 0.00% | 0.00% |  | 0.00% | 0.00% | 0.00% |  | 0.00% | 0.00% | 0.00% |  | 0.00% | 0.00% | 0.00% |
|  |  | Other intentional injuries | 0.00% | 0.00% | 0.00% |  | 5.71% | 0.74% | 10.68% |  | 1.56% | 0.52% | 2.61% |  | 0.24% | 0.06% | 0.42% |
| Men | |  |  |  |  |  |  |  |  |  |  |  |  |  |  |  |  |
| Injuries | |  |  |  |  |  |  |  |  |  |  |  |  |  |  |  |  |
|  | Unintentional injuries | |  |  |  |  |  |  |  |  |  |  |  |  |  |  |  |
|  |  | Transport injuries | 0.00% | 0.00% | 0.00% |  | 7.16% | 4.39% | 9.93% |  | 7.57% | 4.49% | 10.65% |  | 2.16% | 1.12% | 3.21% |
|  |  | Poisonings | 0.00% | 0.00% | 0.00% |  | 12.91% | 5.24% | 20.58% |  | 17.47% | 7.17% | 27.76% |  | 7.63% | 2.70% | 12.56% |
|  |  | Falls | 0.00% | 0.00% | 0.00% |  | 12.91% | 5.24% | 20.58% |  | 17.47% | 7.17% | 27.76% |  | 7.63% | 2.70% | 12.56% |
|  |  | Fires, heat and hot substances | 0.00% | 0.00% | 0.00% |  | 12.91% | 5.24% | 20.58% |  | 17.47% | 7.17% | 27.76% |  | 7.63% | 2.70% | 12.56% |
|  |  | Drownings | 0.00% | 0.00% | 0.00% |  | 12.91% | 5.24% | 20.58% |  | 17.47% | 7.17% | 27.76% |  | 7.63% | 2.70% | 12.56% |
|  |  | Other unintentional injuries | 0.00% | 0.00% | 0.00% |  | 12.91% | 5.24% | 20.58% |  | 17.47% | 7.17% | 27.76% |  | 7.63% | 2.70% | 12.56% |
|  | Intentional injuries | |  |  |  |  |  |  |  |  |  |  |  |  |  |  |  |
|  |  | Self-inflicted injuries | 0.00% | 0.00% | 0.00% |  | 12.91% | 5.24% | 20.58% |  | 17.47% | 7.17% | 27.76% |  | 7.63% | 2.70% | 12.56% |
|  |  | Violence | 0.00% | 0.00% | 0.00% |  | 0.00% | 0.00% | 0.00% |  | 0.00% | 0.00% | 0.00% |  | 0.00% | 0.00% | 0.00% |
|  |  | Other intentional injuries | 0.00% | 0.00% | 0.00% |  | 12.91% | 5.24% | 20.58% |  | 17.47% | 7.17% | 27.76% |  | 7.63% | 2.70% | 12.56% |

Table 29: Mortality Alcohol-Attributable Fractions for injuries (without harms to others included): Europe Central

|  |  |  | 0 to 14 years of age | | |  | 15 to 34 years of age | | |  | 35 to 64 years of age | | |  | 65 years of age and older | | |
| --- | --- | --- | --- | --- | --- | --- | --- | --- | --- | --- | --- | --- | --- | --- | --- | --- | --- |
|  |  |  | Point estimate | Lower 95% CI | Upper 95% CI |  | Point estimate | Lower 95% CI | Upper 95% CI |  | Point estimate | Lower 95% CI | Upper 95% CI |  | Point estimate | Lower 95% CI | Upper 95% CI |
| Women | |  |  |  |  |  |  |  |  |  |  |  |  |  |  |  |  |
| Injuries | |  |  |  |  |  |  |  |  |  |  |  |  |  |  |  |  |
|  | Unintentional injuries | |  |  |  |  |  |  |  |  |  |  |  |  |  |  |  |
|  |  | Transport injuries | 0.00% | 0.00% | 0.00% |  | 7.38% | 4.51% | 10.25% |  | 12.39% | 3.63% | 21.16% |  | 5.42% | 1.37% | 9.46% |
|  |  | Poisonings | 0.00% | 0.00% | 0.00% |  | 11.46% | 0.15% | 22.77% |  | 13.90% | 0.53% | 27.28% |  | 1.02% | 0.19% | 1.84% |
|  |  | Falls | 0.00% | 0.00% | 0.00% |  | 11.46% | 0.15% | 22.77% |  | 13.90% | 0.53% | 27.28% |  | 1.02% | 0.19% | 1.84% |
|  |  | Fires, heat and hot substances | 0.00% | 0.00% | 0.00% |  | 11.46% | 0.15% | 22.77% |  | 13.90% | 0.53% | 27.28% |  | 1.02% | 0.19% | 1.84% |
|  |  | Drownings | 0.00% | 0.00% | 0.00% |  | 11.46% | 0.15% | 22.77% |  | 13.90% | 0.53% | 27.28% |  | 1.02% | 0.19% | 1.84% |
|  |  | Other unintentional injuries | 0.00% | 0.00% | 0.00% |  | 11.46% | 0.15% | 22.77% |  | 13.90% | 0.53% | 27.28% |  | 1.02% | 0.19% | 1.84% |
|  | Intentional injuries | |  |  |  |  |  |  |  |  |  |  |  |  |  |  |  |
|  |  | Self-inflicted injuries | 0.00% | 0.00% | 0.00% |  | 11.46% | 0.15% | 22.77% |  | 13.90% | 0.53% | 27.28% |  | 1.02% | 0.19% | 1.84% |
|  |  | Violence | 0.00% | 0.00% | 0.00% |  | 0.00% | 0.00% | 0.00% |  | 0.00% | 0.00% | 0.00% |  | 0.00% | 0.00% | 0.00% |
|  |  | Other intentional injuries | 0.00% | 0.00% | 0.00% |  | 11.46% | 0.15% | 22.77% |  | 13.90% | 0.53% | 27.28% |  | 1.02% | 0.19% | 1.84% |
| Men | |  |  |  |  |  |  |  |  |  |  |  |  |  |  |  |  |
| Injuries | |  |  |  |  |  |  |  |  |  |  |  |  |  |  |  |  |
|  | Unintentional injuries | |  |  |  |  |  |  |  |  |  |  |  |  |  |  |  |
|  |  | Transport injuries | 0.00% | 0.00% | 0.00% |  | 24.02% | 14.68% | 33.36% |  | 40.33% | 11.81% | 68.86% |  | 17.63% | 4.45% | 30.80% |
|  |  | Poisonings | 0.00% | 0.00% | 0.00% |  | 38.23% | 17.56% | 58.91% |  | 66.75% | 33.38% | 100.11% |  | 41.57% | 18.08% | 65.07% |
|  |  | Falls | 0.00% | 0.00% | 0.00% |  | 38.23% | 17.56% | 58.91% |  | 66.75% | 33.38% | 100.11% |  | 41.57% | 18.08% | 65.07% |
|  |  | Fires, heat and hot substances | 0.00% | 0.00% | 0.00% |  | 38.23% | 17.56% | 58.91% |  | 66.75% | 33.38% | 100.11% |  | 41.57% | 18.08% | 65.07% |
|  |  | Drownings | 0.00% | 0.00% | 0.00% |  | 38.23% | 17.56% | 58.91% |  | 66.75% | 33.38% | 100.11% |  | 41.57% | 18.08% | 65.07% |
|  |  | Other unintentional injuries | 0.00% | 0.00% | 0.00% |  | 38.23% | 17.56% | 58.91% |  | 66.75% | 33.38% | 100.11% |  | 41.57% | 18.08% | 65.07% |
|  | Intentional injuries | |  |  |  |  |  |  |  |  |  |  |  |  |  |  |  |
|  |  | Self-inflicted injuries | 0.00% | 0.00% | 0.00% |  | 38.23% | 17.56% | 58.91% |  | 66.75% | 33.38% | 100.11% |  | 41.57% | 18.08% | 65.07% |
|  |  | Violence | 0.00% | 0.00% | 0.00% |  | 0.00% | 0.00% | 0.00% |  | 0.00% | 0.00% | 0.00% |  | 0.00% | 0.00% | 0.00% |
|  |  | Other intentional injuries | 0.00% | 0.00% | 0.00% |  | 38.23% | 17.56% | 58.91% |  | 66.75% | 33.38% | 100.11% |  | 41.57% | 18.08% | 65.07% |

Table 30: Mortality Alcohol-Attributable Fractions for injuries (without harms to others included): Europe Eastern

|  |  |  | 0 to 14 years of age | | |  | 15 to 34 years of age | | |  | 35 to 64 years of age | | |  | 65 years of age and older | | |
| --- | --- | --- | --- | --- | --- | --- | --- | --- | --- | --- | --- | --- | --- | --- | --- | --- | --- |
|  |  |  | Point estimate | Lower 95% CI | Upper 95% CI |  | Point estimate | Lower 95% CI | Upper 95% CI |  | Point estimate | Lower 95% CI | Upper 95% CI |  | Point estimate | Lower 95% CI | Upper 95% CI |
| Women | |  |  |  |  |  |  |  |  |  |  |  |  |  |  |  |  |
| Injuries | |  |  |  |  |  |  |  |  |  |  |  |  |  |  |  |  |
|  | Unintentional injuries | |  |  |  |  |  |  |  |  |  |  |  |  |  |  |  |
|  |  | Transport injuries | 0.00% | 0.00% | 0.00% |  | 28.83% | 28.83% | 28.83% |  | 28.83% | 28.83% | 28.83% |  | 27.34% | 20.98% | 28.83% |
|  |  | Poisonings | 0.00% | 0.00% | 0.00% |  | 29.43% | 3.32% | 55.55% |  | 23.70% | 1.71% | 45.68% |  | 2.87% | 0.00% | 6.15% |
|  |  | Falls | 0.00% | 0.00% | 0.00% |  | 29.43% | 3.32% | 55.55% |  | 23.70% | 1.71% | 45.68% |  | 2.87% | 0.00% | 6.15% |
|  |  | Fires, heat and hot substances | 0.00% | 0.00% | 0.00% |  | 29.43% | 3.32% | 55.55% |  | 23.70% | 1.71% | 45.68% |  | 2.87% | 0.00% | 6.15% |
|  |  | Drownings | 0.00% | 0.00% | 0.00% |  | 29.43% | 3.32% | 55.55% |  | 23.70% | 1.71% | 45.68% |  | 2.87% | 0.00% | 6.15% |
|  |  | Other unintentional injuries | 0.00% | 0.00% | 0.00% |  | 29.43% | 3.32% | 55.55% |  | 23.70% | 1.71% | 45.68% |  | 2.87% | 0.00% | 6.15% |
|  | Intentional injuries | |  |  |  |  |  |  |  |  |  |  |  |  |  |  |  |
|  |  | Self-inflicted injuries | 0.00% | 0.00% | 0.00% |  | 29.43% | 3.32% | 55.55% |  | 23.70% | 1.71% | 45.68% |  | 2.87% | 0.00% | 6.15% |
|  |  | Violence | 0.00% | 0.00% | 0.00% |  | 0.00% | 0.00% | 0.00% |  | 0.00% | 0.00% | 0.00% |  | 0.00% | 0.00% | 0.00% |
|  |  | Other intentional injuries | 0.00% | 0.00% | 0.00% |  | 29.43% | 3.32% | 55.55% |  | 23.70% | 1.71% | 45.68% |  | 2.87% | 0.00% | 6.15% |
| Men | |  |  |  |  |  |  |  |  |  |  |  |  |  |  |  |  |
| Injuries | |  |  |  |  |  |  |  |  |  |  |  |  |  |  |  |  |
|  | Unintentional injuries | |  |  |  |  |  |  |  |  |  |  |  |  |  |  |  |
|  |  | Transport injuries | 0.00% | 0.00% | 0.00% |  | 90.00% | 90.00% | 90.00% |  | 90.00% | 90.00% | 90.00% |  | 85.37% | 65.49% | 90.00% |
|  |  | Poisonings | 0.00% | 0.00% | 0.00% |  | 83.36% | 46.51% | 120.21% |  | 74.89% | 41.37% | 108.42% |  | 43.04% | 19.95% | 66.13% |
|  |  | Falls | 0.00% | 0.00% | 0.00% |  | 83.36% | 46.51% | 120.21% |  | 74.89% | 41.37% | 108.42% |  | 43.04% | 19.95% | 66.13% |
|  |  | Fires, heat and hot substances | 0.00% | 0.00% | 0.00% |  | 83.36% | 46.51% | 120.21% |  | 74.89% | 41.37% | 108.42% |  | 43.04% | 19.95% | 66.13% |
|  |  | Drownings | 0.00% | 0.00% | 0.00% |  | 83.36% | 46.51% | 120.21% |  | 74.89% | 41.37% | 108.42% |  | 43.04% | 19.95% | 66.13% |
|  |  | Other unintentional injuries | 0.00% | 0.00% | 0.00% |  | 83.36% | 46.51% | 120.21% |  | 74.89% | 41.37% | 108.42% |  | 43.04% | 19.95% | 66.13% |
|  | Intentional injuries | |  |  |  |  |  |  |  |  |  |  |  |  |  |  |  |
|  |  | Self-inflicted injuries | 0.00% | 0.00% | 0.00% |  | 83.36% | 46.51% | 120.21% |  | 74.89% | 41.37% | 108.42% |  | 43.04% | 19.95% | 66.13% |
|  |  | Violence | 0.00% | 0.00% | 0.00% |  | 0.00% | 0.00% | 0.00% |  | 0.00% | 0.00% | 0.00% |  | 0.00% | 0.00% | 0.00% |
|  |  | Other intentional injuries | 0.00% | 0.00% | 0.00% |  | 83.36% | 46.51% | 120.21% |  | 74.89% | 41.37% | 108.42% |  | 43.04% | 19.95% | 66.13% |

Table 31: Mortality Alcohol-Attributable Fractions for injuries (without harms to others included): Europe Western

|  |  |  | 0 to 14 years of age | | |  | 15 to 34 years of age | | |  | 35 to 64 years of age | | |  | 65 years of age and older | | |
| --- | --- | --- | --- | --- | --- | --- | --- | --- | --- | --- | --- | --- | --- | --- | --- | --- | --- |
|  |  |  | Point estimate | Lower 95% CI | Upper 95% CI |  | Point estimate | Lower 95% CI | Upper 95% CI |  | Point estimate | Lower 95% CI | Upper 95% CI |  | Point estimate | Lower 95% CI | Upper 95% CI |
| Women | |  |  |  |  |  |  |  |  |  |  |  |  |  |  |  |  |
| Injuries | |  |  |  |  |  |  |  |  |  |  |  |  |  |  |  |  |
|  | Unintentional injuries | |  |  |  |  |  |  |  |  |  |  |  |  |  |  |  |
|  |  | Transport injuries | 0.00% | 0.00% | 0.00% |  | 4.14% | 2.25% | 6.03% |  | 5.54% | 2.17% | 8.90% |  | 1.87% | 0.74% | 3.00% |
|  |  | Poisonings | 0.00% | 0.00% | 0.00% |  | 6.32% | 2.28% | 10.35% |  | 11.06% | 1.28% | 20.84% |  | 3.96% | 1.44% | 6.48% |
|  |  | Falls | 0.00% | 0.00% | 0.00% |  | 6.32% | 2.28% | 10.35% |  | 11.06% | 1.28% | 20.84% |  | 3.96% | 1.44% | 6.48% |
|  |  | Fires, heat and hot substances | 0.00% | 0.00% | 0.00% |  | 6.32% | 2.28% | 10.35% |  | 11.06% | 1.28% | 20.84% |  | 3.96% | 1.44% | 6.48% |
|  |  | Drownings | 0.00% | 0.00% | 0.00% |  | 6.32% | 2.28% | 10.35% |  | 11.06% | 1.28% | 20.84% |  | 3.96% | 1.44% | 6.48% |
|  |  | Other unintentional injuries | 0.00% | 0.00% | 0.00% |  | 6.32% | 2.28% | 10.35% |  | 11.06% | 1.28% | 20.84% |  | 3.96% | 1.44% | 6.48% |
|  | Intentional injuries | |  |  |  |  |  |  |  |  |  |  |  |  |  |  |  |
|  |  | Self-inflicted injuries | 0.00% | 0.00% | 0.00% |  | 6.32% | 2.28% | 10.35% |  | 11.06% | 1.28% | 20.84% |  | 3.96% | 1.44% | 6.48% |
|  |  | Violence | 0.00% | 0.00% | 0.00% |  | 0.00% | 0.00% | 0.00% |  | 0.00% | 0.00% | 0.00% |  | 0.00% | 0.00% | 0.00% |
|  |  | Other intentional injuries | 0.00% | 0.00% | 0.00% |  | 6.32% | 2.28% | 10.35% |  | 11.06% | 1.28% | 20.84% |  | 3.96% | 1.44% | 6.48% |
| Men | |  |  |  |  |  |  |  |  |  |  |  |  |  |  |  |  |
| Injuries | |  |  |  |  |  |  |  |  |  |  |  |  |  |  |  |  |
|  | Unintentional injuries | |  |  |  |  |  |  |  |  |  |  |  |  |  |  |  |
|  |  | Transport injuries | 0.00% | 0.00% | 0.00% |  | 10.36% | 5.64% | 15.08% |  | 13.84% | 5.42% | 22.27% |  | 4.67% | 1.85% | 7.50% |
|  |  | Poisonings | 0.00% | 0.00% | 0.00% |  | 27.79% | 12.47% | 43.10% |  | 39.40% | 18.48% | 60.33% |  | 20.52% | 8.60% | 32.44% |
|  |  | Falls | 0.00% | 0.00% | 0.00% |  | 27.79% | 12.47% | 43.10% |  | 39.40% | 18.48% | 60.33% |  | 20.52% | 8.60% | 32.44% |
|  |  | Fires, heat and hot substances | 0.00% | 0.00% | 0.00% |  | 27.79% | 12.47% | 43.10% |  | 39.40% | 18.48% | 60.33% |  | 20.52% | 8.60% | 32.44% |
|  |  | Drownings | 0.00% | 0.00% | 0.00% |  | 27.79% | 12.47% | 43.10% |  | 39.40% | 18.48% | 60.33% |  | 20.52% | 8.60% | 32.44% |
|  |  | Other unintentional injuries | 0.00% | 0.00% | 0.00% |  | 27.79% | 12.47% | 43.10% |  | 39.40% | 18.48% | 60.33% |  | 20.52% | 8.60% | 32.44% |
|  | Intentional injuries | |  |  |  |  |  |  |  |  |  |  |  |  |  |  |  |
|  |  | Self-inflicted injuries | 0.00% | 0.00% | 0.00% |  | 27.79% | 12.47% | 43.10% |  | 39.40% | 18.48% | 60.33% |  | 20.52% | 8.60% | 32.44% |
|  |  | Violence | 0.00% | 0.00% | 0.00% |  | 0.00% | 0.00% | 0.00% |  | 0.00% | 0.00% | 0.00% |  | 0.00% | 0.00% | 0.00% |
|  |  | Other intentional injuries | 0.00% | 0.00% | 0.00% |  | 27.79% | 12.47% | 43.10% |  | 39.40% | 18.48% | 60.33% |  | 20.52% | 8.60% | 32.44% |

Table 32: Mortality Alcohol-Attributable Fractions for injuries (without harms to others included): Latin America Andean

|  |  |  | 0 to 14 years of age | | |  | 15 to 34 years of age | | |  | 35 to 64 years of age | | |  | 65 years of age and older | | |
| --- | --- | --- | --- | --- | --- | --- | --- | --- | --- | --- | --- | --- | --- | --- | --- | --- | --- |
|  |  |  | Point estimate | Lower 95% CI | Upper 95% CI |  | Point estimate | Lower 95% CI | Upper 95% CI |  | Point estimate | Lower 95% CI | Upper 95% CI |  | Point estimate | Lower 95% CI | Upper 95% CI |
| Women | |  |  |  |  |  |  |  |  |  |  |  |  |  |  |  |  |
| Injuries | |  |  |  |  |  |  |  |  |  |  |  |  |  |  |  |  |
|  | Unintentional injuries | |  |  |  |  |  |  |  |  |  |  |  |  |  |  |  |
|  |  | Transport injuries | 0.00% | 0.00% | 0.00% |  | 3.35% | 2.03% | 4.68% |  | 4.47% | 2.13% | 6.80% |  | 0.54% | 0.30% | 0.78% |
|  |  | Poisonings | 0.00% | 0.00% | 0.00% |  | 4.87% | 0.00% | 12.71% |  | 3.41% | 0.00% | 7.44% |  | 1.70% | 0.00% | 3.98% |
|  |  | Falls | 0.00% | 0.00% | 0.00% |  | 4.87% | 0.00% | 12.71% |  | 3.41% | 0.00% | 7.44% |  | 1.70% | 0.00% | 3.98% |
|  |  | Fires, heat and hot substances | 0.00% | 0.00% | 0.00% |  | 4.87% | 0.00% | 12.71% |  | 3.41% | 0.00% | 7.44% |  | 1.70% | 0.00% | 3.98% |
|  |  | Drownings | 0.00% | 0.00% | 0.00% |  | 4.87% | 0.00% | 12.71% |  | 3.41% | 0.00% | 7.44% |  | 1.70% | 0.00% | 3.98% |
|  |  | Other unintentional injuries | 0.00% | 0.00% | 0.00% |  | 4.87% | 0.00% | 12.71% |  | 3.41% | 0.00% | 7.44% |  | 1.70% | 0.00% | 3.98% |
|  | Intentional injuries | |  |  |  |  |  |  |  |  |  |  |  |  |  |  |  |
|  |  | Self-inflicted injuries | 0.00% | 0.00% | 0.00% |  | 4.87% | 0.00% | 12.71% |  | 3.41% | 0.00% | 7.44% |  | 1.70% | 0.00% | 3.98% |
|  |  | Violence | 0.00% | 0.00% | 0.00% |  | 0.00% | 0.00% | 0.00% |  | 0.00% | 0.00% | 0.00% |  | 0.00% | 0.00% | 0.00% |
|  |  | Other intentional injuries | 0.00% | 0.00% | 0.00% |  | 4.87% | 0.00% | 12.71% |  | 3.41% | 0.00% | 7.44% |  | 1.70% | 0.00% | 3.98% |
| Men | |  |  |  |  |  |  |  |  |  |  |  |  |  |  |  |  |
| Injuries | |  |  |  |  |  |  |  |  |  |  |  |  |  |  |  |  |
|  | Unintentional injuries | |  |  |  |  |  |  |  |  |  |  |  |  |  |  |  |
|  |  | Transport injuries | 0.00% | 0.00% | 0.00% |  | 11.11% | 6.72% | 15.49% |  | 14.78% | 7.05% | 22.52% |  | 1.79% | 0.98% | 2.59% |
|  |  | Poisonings | 0.00% | 0.00% | 0.00% |  | 16.79% | 4.82% | 28.76% |  | 28.46% | 8.18% | 48.74% |  | 2.90% | 0.74% | 5.06% |
|  |  | Falls | 0.00% | 0.00% | 0.00% |  | 16.79% | 4.82% | 28.76% |  | 28.46% | 8.18% | 48.74% |  | 2.90% | 0.74% | 5.06% |
|  |  | Fires, heat and hot substances | 0.00% | 0.00% | 0.00% |  | 16.79% | 4.82% | 28.76% |  | 28.46% | 8.18% | 48.74% |  | 2.90% | 0.74% | 5.06% |
|  |  | Drownings | 0.00% | 0.00% | 0.00% |  | 16.79% | 4.82% | 28.76% |  | 28.46% | 8.18% | 48.74% |  | 2.90% | 0.74% | 5.06% |
|  |  | Other unintentional injuries | 0.00% | 0.00% | 0.00% |  | 16.79% | 4.82% | 28.76% |  | 28.46% | 8.18% | 48.74% |  | 2.90% | 0.74% | 5.06% |
|  | Intentional injuries | |  |  |  |  |  |  |  |  |  |  |  |  |  |  |  |
|  |  | Self-inflicted injuries | 0.00% | 0.00% | 0.00% |  | 16.79% | 4.82% | 28.76% |  | 28.46% | 8.18% | 48.74% |  | 2.90% | 0.74% | 5.06% |
|  |  | Violence | 0.00% | 0.00% | 0.00% |  | 0.00% | 0.00% | 0.00% |  | 0.00% | 0.00% | 0.00% |  | 0.00% | 0.00% | 0.00% |
|  |  | Other intentional injuries | 0.00% | 0.00% | 0.00% |  | 16.79% | 4.82% | 28.76% |  | 28.46% | 8.18% | 48.74% |  | 2.90% | 0.74% | 5.06% |

Table 33: Mortality Alcohol-Attributable Fractions for injuries (without harms to others included): Latin America Central

|  |  |  | 0 to 14 years of age | | |  | 15 to 34 years of age | | |  | 35 to 64 years of age | | |  | 65 years of age and older | | |
| --- | --- | --- | --- | --- | --- | --- | --- | --- | --- | --- | --- | --- | --- | --- | --- | --- | --- |
|  |  |  | Point estimate | Lower 95% CI | Upper 95% CI |  | Point estimate | Lower 95% CI | Upper 95% CI |  | Point estimate | Lower 95% CI | Upper 95% CI |  | Point estimate | Lower 95% CI | Upper 95% CI |
| Women | |  |  |  |  |  |  |  |  |  |  |  |  |  |  |  |  |
| Injuries | |  |  |  |  |  |  |  |  |  |  |  |  |  |  |  |  |
|  | Unintentional injuries | |  |  |  |  |  |  |  |  |  |  |  |  |  |  |  |
|  |  | Transport injuries | 0.00% | 0.00% | 0.00% |  | 8.99% | 5.74% | 12.24% |  | 10.97% | 7.25% | 14.70% |  | 5.80% | 3.42% | 8.18% |
|  |  | Poisonings | 0.00% | 0.00% | 0.00% |  | 5.07% | 0.00% | 12.05% |  | 3.27% | 0.00% | 7.58% |  | 0.90% | 0.05% | 1.76% |
|  |  | Falls | 0.00% | 0.00% | 0.00% |  | 5.07% | 0.00% | 12.05% |  | 3.27% | 0.00% | 7.58% |  | 0.90% | 0.05% | 1.76% |
|  |  | Fires, heat and hot substances | 0.00% | 0.00% | 0.00% |  | 5.07% | 0.00% | 12.05% |  | 3.27% | 0.00% | 7.58% |  | 0.90% | 0.05% | 1.76% |
|  |  | Drownings | 0.00% | 0.00% | 0.00% |  | 5.07% | 0.00% | 12.05% |  | 3.27% | 0.00% | 7.58% |  | 0.90% | 0.05% | 1.76% |
|  |  | Other unintentional injuries | 0.00% | 0.00% | 0.00% |  | 5.07% | 0.00% | 12.05% |  | 3.27% | 0.00% | 7.58% |  | 0.90% | 0.05% | 1.76% |
|  | Intentional injuries | |  |  |  |  |  |  |  |  |  |  |  |  |  |  |  |
|  |  | Self-inflicted injuries | 0.00% | 0.00% | 0.00% |  | 5.07% | 0.00% | 12.05% |  | 3.27% | 0.00% | 7.58% |  | 0.90% | 0.05% | 1.76% |
|  |  | Violence | 0.00% | 0.00% | 0.00% |  | 0.00% | 0.00% | 0.00% |  | 0.00% | 0.00% | 0.00% |  | 0.00% | 0.00% | 0.00% |
|  |  | Other intentional injuries | 0.00% | 0.00% | 0.00% |  | 5.07% | 0.00% | 12.05% |  | 3.27% | 0.00% | 7.58% |  | 0.90% | 0.05% | 1.76% |
| Men | |  |  |  |  |  |  |  |  |  |  |  |  |  |  |  |  |
| Injuries | |  |  |  |  |  |  |  |  |  |  |  |  |  |  |  |  |
|  | Unintentional injuries | |  |  |  |  |  |  |  |  |  |  |  |  |  |  |  |
|  |  | Transport injuries | 0.00% | 0.00% | 0.00% |  | 32.66% | 20.85% | 44.46% |  | 39.86% | 26.33% | 53.40% |  | 21.07% | 12.41% | 29.73% |
|  |  | Poisonings | 0.00% | 0.00% | 0.00% |  | 23.72% | 9.65% | 37.79% |  | 31.75% | 13.55% | 49.96% |  | 19.22% | 6.90% | 31.55% |
|  |  | Falls | 0.00% | 0.00% | 0.00% |  | 23.72% | 9.65% | 37.79% |  | 31.75% | 13.55% | 49.96% |  | 19.22% | 6.90% | 31.55% |
|  |  | Fires, heat and hot substances | 0.00% | 0.00% | 0.00% |  | 23.72% | 9.65% | 37.79% |  | 31.75% | 13.55% | 49.96% |  | 19.22% | 6.90% | 31.55% |
|  |  | Drownings | 0.00% | 0.00% | 0.00% |  | 23.72% | 9.65% | 37.79% |  | 31.75% | 13.55% | 49.96% |  | 19.22% | 6.90% | 31.55% |
|  |  | Other unintentional injuries | 0.00% | 0.00% | 0.00% |  | 23.72% | 9.65% | 37.79% |  | 31.75% | 13.55% | 49.96% |  | 19.22% | 6.90% | 31.55% |
|  | Intentional injuries | |  |  |  |  |  |  |  |  |  |  |  |  |  |  |  |
|  |  | Self-inflicted injuries | 0.00% | 0.00% | 0.00% |  | 23.72% | 9.65% | 37.79% |  | 31.75% | 13.55% | 49.96% |  | 19.22% | 6.90% | 31.55% |
|  |  | Violence | 0.00% | 0.00% | 0.00% |  | 0.00% | 0.00% | 0.00% |  | 0.00% | 0.00% | 0.00% |  | 0.00% | 0.00% | 0.00% |
|  |  | Other intentional injuries | 0.00% | 0.00% | 0.00% |  | 23.72% | 9.65% | 37.79% |  | 31.75% | 13.55% | 49.96% |  | 19.22% | 6.90% | 31.55% |

Table 34: Mortality Alcohol-Attributable Fractions for injuries (without harms to others included): Latin America Southern

|  |  |  | 0 to 14 years of age | | |  | 15 to 34 years of age | | |  | 35 to 64 years of age | | |  | 65 years of age and older | | |
| --- | --- | --- | --- | --- | --- | --- | --- | --- | --- | --- | --- | --- | --- | --- | --- | --- | --- |
|  |  |  | Point estimate | Lower 95% CI | Upper 95% CI |  | Point estimate | Lower 95% CI | Upper 95% CI |  | Point estimate | Lower 95% CI | Upper 95% CI |  | Point estimate | Lower 95% CI | Upper 95% CI |
| Women | |  |  |  |  |  |  |  |  |  |  |  |  |  |  |  |  |
| Injuries | |  |  |  |  |  |  |  |  |  |  |  |  |  |  |  |  |
|  | Unintentional injuries | |  |  |  |  |  |  |  |  |  |  |  |  |  |  |  |
|  |  | Transport injuries | 0.00% | 0.00% | 0.00% |  | 4.10% | 2.47% | 5.73% |  | 3.00% | 1.52% | 4.48% |  | 1.50% | 0.73% | 2.28% |
|  |  | Poisonings | 0.00% | 0.00% | 0.00% |  | 4.28% | 1.12% | 7.45% |  | 7.74% | 0.00% | 16.42% |  | 2.87% | 0.72% | 5.03% |
|  |  | Falls | 0.00% | 0.00% | 0.00% |  | 4.28% | 1.12% | 7.45% |  | 7.74% | 0.00% | 16.42% |  | 2.87% | 0.72% | 5.03% |
|  |  | Fires, heat and hot substances | 0.00% | 0.00% | 0.00% |  | 4.28% | 1.12% | 7.45% |  | 7.74% | 0.00% | 16.42% |  | 2.87% | 0.72% | 5.03% |
|  |  | Drownings | 0.00% | 0.00% | 0.00% |  | 4.28% | 1.12% | 7.45% |  | 7.74% | 0.00% | 16.42% |  | 2.87% | 0.72% | 5.03% |
|  |  | Other unintentional injuries | 0.00% | 0.00% | 0.00% |  | 4.28% | 1.12% | 7.45% |  | 7.74% | 0.00% | 16.42% |  | 2.87% | 0.72% | 5.03% |
|  | Intentional injuries | |  |  |  |  |  |  |  |  |  |  |  |  |  |  |  |
|  |  | Self-inflicted injuries | 0.00% | 0.00% | 0.00% |  | 4.28% | 1.12% | 7.45% |  | 7.74% | 0.00% | 16.42% |  | 2.87% | 0.72% | 5.03% |
|  |  | Violence | 0.00% | 0.00% | 0.00% |  | 0.00% | 0.00% | 0.00% |  | 0.00% | 0.00% | 0.00% |  | 0.00% | 0.00% | 0.00% |
|  |  | Other intentional injuries | 0.00% | 0.00% | 0.00% |  | 4.28% | 1.12% | 7.45% |  | 7.74% | 0.00% | 16.42% |  | 2.87% | 0.72% | 5.03% |
| Men | |  |  |  |  |  |  |  |  |  |  |  |  |  |  |  |  |
| Injuries | |  |  |  |  |  |  |  |  |  |  |  |  |  |  |  |  |
|  | Unintentional injuries | |  |  |  |  |  |  |  |  |  |  |  |  |  |  |  |
|  |  | Transport injuries | 0.00% | 0.00% | 0.00% |  | 10.80% | 6.51% | 15.10% |  | 7.91% | 4.01% | 11.82% |  | 3.96% | 1.91% | 6.01% |
|  |  | Poisonings | 0.00% | 0.00% | 0.00% |  | 22.98% | 9.38% | 36.57% |  | 23.33% | 9.25% | 37.41% |  | 14.69% | 5.27% | 24.10% |
|  |  | Falls | 0.00% | 0.00% | 0.00% |  | 22.98% | 9.38% | 36.57% |  | 23.33% | 9.25% | 37.41% |  | 14.69% | 5.27% | 24.10% |
|  |  | Fires, heat and hot substances | 0.00% | 0.00% | 0.00% |  | 22.98% | 9.38% | 36.57% |  | 23.33% | 9.25% | 37.41% |  | 14.69% | 5.27% | 24.10% |
|  |  | Drownings | 0.00% | 0.00% | 0.00% |  | 22.98% | 9.38% | 36.57% |  | 23.33% | 9.25% | 37.41% |  | 14.69% | 5.27% | 24.10% |
|  |  | Other unintentional injuries | 0.00% | 0.00% | 0.00% |  | 22.98% | 9.38% | 36.57% |  | 23.33% | 9.25% | 37.41% |  | 14.69% | 5.27% | 24.10% |
|  | Intentional injuries | |  |  |  |  |  |  |  |  |  |  |  |  |  |  |  |
|  |  | Self-inflicted injuries | 0.00% | 0.00% | 0.00% |  | 22.98% | 9.38% | 36.57% |  | 23.33% | 9.25% | 37.41% |  | 14.69% | 5.27% | 24.10% |
|  |  | Violence | 0.00% | 0.00% | 0.00% |  | 0.00% | 0.00% | 0.00% |  | 0.00% | 0.00% | 0.00% |  | 0.00% | 0.00% | 0.00% |
|  |  | Other intentional injuries | 0.00% | 0.00% | 0.00% |  | 22.98% | 9.38% | 36.57% |  | 23.33% | 9.25% | 37.41% |  | 14.69% | 5.27% | 24.10% |

Table 35: Mortality Alcohol-Attributable Fractions for injuries (without harms to others included): Latin America Tropical

|  |  |  | 0 to 14 years of age | | |  | 15 to 34 years of age | | |  | 35 to 64 years of age | | |  | 65 years of age and older | | |
| --- | --- | --- | --- | --- | --- | --- | --- | --- | --- | --- | --- | --- | --- | --- | --- | --- | --- |
|  |  |  | Point estimate | Lower 95% CI | Upper 95% CI |  | Point estimate | Lower 95% CI | Upper 95% CI |  | Point estimate | Lower 95% CI | Upper 95% CI |  | Point estimate | Lower 95% CI | Upper 95% CI |
| Women | |  |  |  |  |  |  |  |  |  |  |  |  |  |  |  |  |
| Injuries | |  |  |  |  |  |  |  |  |  |  |  |  |  |  |  |  |
|  | Unintentional injuries | |  |  |  |  |  |  |  |  |  |  |  |  |  |  |  |
|  |  | Transport injuries | 0.00% | 0.00% | 0.00% |  | 6.17% | 3.10% | 9.24% |  | 4.90% | 1.05% | 8.75% |  | 0.53% | 0.25% | 0.82% |
|  |  | Poisonings | 0.00% | 0.00% | 0.00% |  | 6.37% | 0.00% | 13.64% |  | 9.45% | 0.00% | 20.55% |  | 0.83% | 0.02% | 1.64% |
|  |  | Falls | 0.00% | 0.00% | 0.00% |  | 6.37% | 0.00% | 13.64% |  | 9.45% | 0.00% | 20.55% |  | 0.83% | 0.02% | 1.64% |
|  |  | Fires, heat and hot substances | 0.00% | 0.00% | 0.00% |  | 6.37% | 0.00% | 13.64% |  | 9.45% | 0.00% | 20.55% |  | 0.83% | 0.02% | 1.64% |
|  |  | Drownings | 0.00% | 0.00% | 0.00% |  | 6.37% | 0.00% | 13.64% |  | 9.45% | 0.00% | 20.55% |  | 0.83% | 0.02% | 1.64% |
|  |  | Other unintentional injuries | 0.00% | 0.00% | 0.00% |  | 6.37% | 0.00% | 13.64% |  | 9.45% | 0.00% | 20.55% |  | 0.83% | 0.02% | 1.64% |
|  | Intentional injuries | |  |  |  |  |  |  |  |  |  |  |  |  |  |  |  |
|  |  | Self-inflicted injuries | 0.00% | 0.00% | 0.00% |  | 6.37% | 0.00% | 13.64% |  | 9.45% | 0.00% | 20.55% |  | 0.83% | 0.02% | 1.64% |
|  |  | Violence | 0.00% | 0.00% | 0.00% |  | 0.00% | 0.00% | 0.00% |  | 0.00% | 0.00% | 0.00% |  | 0.00% | 0.00% | 0.00% |
|  |  | Other intentional injuries | 0.00% | 0.00% | 0.00% |  | 6.37% | 0.00% | 13.64% |  | 9.45% | 0.00% | 20.55% |  | 0.83% | 0.02% | 1.64% |
| Men | |  |  |  |  |  |  |  |  |  |  |  |  |  |  |  |  |
| Injuries | |  |  |  |  |  |  |  |  |  |  |  |  |  |  |  |  |
|  | Unintentional injuries | |  |  |  |  |  |  |  |  |  |  |  |  |  |  |  |
|  |  | Transport injuries | 0.00% | 0.00% | 0.00% |  | 19.85% | 9.98% | 29.72% |  | 15.76% | 3.38% | 28.15% |  | 1.72% | 0.79% | 2.64% |
|  |  | Poisonings | 0.00% | 0.00% | 0.00% |  | 35.44% | 13.95% | 56.94% |  | 33.25% | 11.80% | 54.70% |  | 5.55% | 1.45% | 9.66% |
|  |  | Falls | 0.00% | 0.00% | 0.00% |  | 35.44% | 13.95% | 56.94% |  | 33.25% | 11.80% | 54.70% |  | 5.55% | 1.45% | 9.66% |
|  |  | Fires, heat and hot substances | 0.00% | 0.00% | 0.00% |  | 35.44% | 13.95% | 56.94% |  | 33.25% | 11.80% | 54.70% |  | 5.55% | 1.45% | 9.66% |
|  |  | Drownings | 0.00% | 0.00% | 0.00% |  | 35.44% | 13.95% | 56.94% |  | 33.25% | 11.80% | 54.70% |  | 5.55% | 1.45% | 9.66% |
|  |  | Other unintentional injuries | 0.00% | 0.00% | 0.00% |  | 35.44% | 13.95% | 56.94% |  | 33.25% | 11.80% | 54.70% |  | 5.55% | 1.45% | 9.66% |
|  | Intentional injuries | |  |  |  |  |  |  |  |  |  |  |  |  |  |  |  |
|  |  | Self-inflicted injuries | 0.00% | 0.00% | 0.00% |  | 35.44% | 13.95% | 56.94% |  | 33.25% | 11.80% | 54.70% |  | 5.55% | 1.45% | 9.66% |
|  |  | Violence | 0.00% | 0.00% | 0.00% |  | 0.00% | 0.00% | 0.00% |  | 0.00% | 0.00% | 0.00% |  | 0.00% | 0.00% | 0.00% |
|  |  | Other intentional injuries | 0.00% | 0.00% | 0.00% |  | 35.44% | 13.95% | 56.94% |  | 33.25% | 11.80% | 54.70% |  | 5.55% | 1.45% | 9.66% |

Table 36: Mortality Alcohol-Attributable Fractions for injuries (without harms to others included): North Africa Middle East

|  |  |  | 0 to 14 years of age | | |  | 15 to 34 years of age | | |  | 35 to 64 years of age | | |  | 65 years of age and older | | |
| --- | --- | --- | --- | --- | --- | --- | --- | --- | --- | --- | --- | --- | --- | --- | --- | --- | --- |
|  |  |  | Point estimate | Lower 95% CI | Upper 95% CI |  | Point estimate | Lower 95% CI | Upper 95% CI |  | Point estimate | Lower 95% CI | Upper 95% CI |  | Point estimate | Lower 95% CI | Upper 95% CI |
| Women | |  |  |  |  |  |  |  |  |  |  |  |  |  |  |  |  |
| Injuries | |  |  |  |  |  |  |  |  |  |  |  |  |  |  |  |  |
|  | Unintentional injuries | |  |  |  |  |  |  |  |  |  |  |  |  |  |  |  |
|  |  | Transport injuries | 0.00% | 0.00% | 0.00% |  | 0.52% | 0.00% | 2.87% |  | 0.10% | 0.00% | 0.31% |  | 0.01% | 0.00% | 0.02% |
|  |  | Poisonings | 0.00% | 0.00% | 0.00% |  | 0.49% | 0.00% | 3.27% |  | 0.29% | 0.00% | 4.26% |  | 0.04% | 0.00% | 1.27% |
|  |  | Falls | 0.00% | 0.00% | 0.00% |  | 0.49% | 0.00% | 3.27% |  | 0.29% | 0.00% | 4.26% |  | 0.04% | 0.00% | 1.27% |
|  |  | Fires, heat and hot substances | 0.00% | 0.00% | 0.00% |  | 0.49% | 0.00% | 3.27% |  | 0.29% | 0.00% | 4.26% |  | 0.04% | 0.00% | 1.27% |
|  |  | Drownings | 0.00% | 0.00% | 0.00% |  | 0.49% | 0.00% | 3.27% |  | 0.29% | 0.00% | 4.26% |  | 0.04% | 0.00% | 1.27% |
|  |  | Other unintentional injuries | 0.00% | 0.00% | 0.00% |  | 0.49% | 0.00% | 3.27% |  | 0.29% | 0.00% | 4.26% |  | 0.04% | 0.00% | 1.27% |
|  | Intentional injuries | |  |  |  |  |  |  |  |  |  |  |  |  |  |  |  |
|  |  | Self-inflicted injuries | 0.00% | 0.00% | 0.00% |  | 0.49% | 0.00% | 3.27% |  | 0.29% | 0.00% | 4.26% |  | 0.04% | 0.00% | 1.27% |
|  |  | Violence | 0.00% | 0.00% | 0.00% |  | 0.00% | 0.00% | 0.00% |  | 0.00% | 0.00% | 0.00% |  | 0.00% | 0.00% | 0.00% |
|  |  | Other intentional injuries |  |  |  |  |  |  |  |  |  |  |  |  |  |  |  |
| Men | |  |  |  |  |  |  |  |  |  |  |  |  |  |  |  |  |
| Injuries | |  |  |  |  |  |  |  |  |  |  |  |  |  |  |  |  |
|  | Unintentional injuries | |  |  |  |  |  |  |  |  |  |  |  |  |  |  |  |
|  |  | Transport injuries | 0.00% | 0.00% | 0.00% |  | 4.04% | 0.00% | 22.24% |  | 0.81% | 0.00% | 2.43% |  | 0.08% | 0.02% | 0.15% |
|  |  | Poisonings | 0.00% | 0.00% | 0.00% |  | 7.91% | 0.16% | 15.66% |  | 3.26% | 0.00% | 6.99% |  | 0.08% | 0.01% | 0.15% |
|  |  | Falls | 0.00% | 0.00% | 0.00% |  | 7.91% | 0.16% | 15.66% |  | 3.26% | 0.00% | 6.99% |  | 0.08% | 0.01% | 0.15% |
|  |  | Fires, heat and hot substances | 0.00% | 0.00% | 0.00% |  | 7.91% | 0.16% | 15.66% |  | 3.26% | 0.00% | 6.99% |  | 0.08% | 0.01% | 0.15% |
|  |  | Drownings | 0.00% | 0.00% | 0.00% |  | 7.91% | 0.16% | 15.66% |  | 3.26% | 0.00% | 6.99% |  | 0.08% | 0.01% | 0.15% |
|  |  | Other unintentional injuries | 0.00% | 0.00% | 0.00% |  | 7.91% | 0.16% | 15.66% |  | 3.26% | 0.00% | 6.99% |  | 0.08% | 0.01% | 0.15% |
|  | Intentional injuries | |  |  |  |  |  |  |  |  |  |  |  |  |  |  |  |
|  |  | Self-inflicted injuries | 0.00% | 0.00% | 0.00% |  | 7.91% | 0.16% | 15.66% |  | 3.26% | 0.00% | 6.99% |  | 0.08% | 0.01% | 0.15% |
|  |  | Violence | 0.00% | 0.00% | 0.00% |  | 0.00% | 0.00% | 0.00% |  | 0.00% | 0.00% | 0.00% |  | 0.00% | 0.00% | 0.00% |
|  |  | Other intentional injuries | 0.00% | 0.00% | 0.00% |  | 7.91% | 0.16% | 15.66% |  | 3.26% | 0.00% | 6.99% |  | 0.08% | 0.01% | 0.15% |

Table 37: Mortality Alcohol-Attributable Fractions for injuries (without harms to others included): North America High Income

|  |  |  | 0 to 14 years of age | | |  | 15 to 34 years of age | | |  | 35 to 64 years of age | | |  | 65 years of age and older | | |
| --- | --- | --- | --- | --- | --- | --- | --- | --- | --- | --- | --- | --- | --- | --- | --- | --- | --- |
|  |  |  | Point estimate | Lower 95% CI | Upper 95% CI |  | Point estimate | Lower 95% CI | Upper 95% CI |  | Point estimate | Lower 95% CI | Upper 95% CI |  | Point estimate | Lower 95% CI | Upper 95% CI |
| Women | |  |  |  |  |  |  |  |  |  |  |  |  |  |  |  |  |
| Injuries | |  |  |  |  |  |  |  |  |  |  |  |  |  |  |  |  |
|  | Unintentional injuries | |  |  |  |  |  |  |  |  |  |  |  |  |  |  |  |
|  |  | Transport injuries | 0.00% | 0.00% | 0.00% |  | 5.72% | 2.60% | 8.84% |  | 2.41% | 1.10% | 3.72% |  | 0.83% | 0.41% | 1.25% |
|  |  | Poisonings | 0.00% | 0.00% | 0.00% |  | 8.00% | 2.31% | 13.69% |  | 5.58% | 1.91% | 9.26% |  | 1.52% | 0.54% | 2.49% |
|  |  | Falls | 0.00% | 0.00% | 0.00% |  | 8.00% | 2.31% | 13.69% |  | 5.58% | 1.91% | 9.26% |  | 1.52% | 0.54% | 2.49% |
|  |  | Fires, heat and hot substances | 0.00% | 0.00% | 0.00% |  | 8.00% | 2.31% | 13.69% |  | 5.58% | 1.91% | 9.26% |  | 1.52% | 0.54% | 2.49% |
|  |  | Drownings | 0.00% | 0.00% | 0.00% |  | 8.00% | 2.31% | 13.69% |  | 5.58% | 1.91% | 9.26% |  | 1.52% | 0.54% | 2.49% |
|  |  | Other unintentional injuries | 0.00% | 0.00% | 0.00% |  | 8.00% | 2.31% | 13.69% |  | 5.58% | 1.91% | 9.26% |  | 1.52% | 0.54% | 2.49% |
|  | Intentional injuries | |  |  |  |  |  |  |  |  |  |  |  |  |  |  |  |
|  |  | Self-inflicted injuries | 0.00% | 0.00% | 0.00% |  | 8.00% | 2.31% | 13.69% |  | 5.58% | 1.91% | 9.26% |  | 1.52% | 0.54% | 2.49% |
|  |  | Violence | 0.00% | 0.00% | 0.00% |  | 0.00% | 0.00% | 0.00% |  | 0.00% | 0.00% | 0.00% |  | 0.00% | 0.00% | 0.00% |
|  |  | Other intentional injuries | 0.00% | 0.00% | 0.00% |  | 8.00% | 2.31% | 13.69% |  | 5.58% | 1.91% | 9.26% |  | 1.52% | 0.54% | 2.49% |
| Men | |  |  |  |  |  |  |  |  |  |  |  |  |  |  |  |  |
| Injuries | |  |  |  |  |  |  |  |  |  |  |  |  |  |  |  |  |
|  | Unintentional injuries | |  |  |  |  |  |  |  |  |  |  |  |  |  |  |  |
|  |  | Transport injuries | 0.00% | 0.00% | 0.00% |  | 16.28% | 7.41% | 25.15% |  | 6.86% | 3.13% | 10.58% |  | 2.36% | 1.17% | 3.55% |
|  |  | Poisonings | 0.00% | 0.00% | 0.00% |  | 38.33% | 17.70% | 58.96% |  | 23.29% | 10.00% | 36.59% |  | 10.29% | 3.86% | 16.73% |
|  |  | Falls | 0.00% | 0.00% | 0.00% |  | 38.33% | 17.70% | 58.96% |  | 23.29% | 10.00% | 36.59% |  | 10.29% | 3.86% | 16.73% |
|  |  | Fires, heat and hot substances | 0.00% | 0.00% | 0.00% |  | 38.33% | 17.70% | 58.96% |  | 23.29% | 10.00% | 36.59% |  | 10.29% | 3.86% | 16.73% |
|  |  | Drownings | 0.00% | 0.00% | 0.00% |  | 38.33% | 17.70% | 58.96% |  | 23.29% | 10.00% | 36.59% |  | 10.29% | 3.86% | 16.73% |
|  |  | Other unintentional injuries | 0.00% | 0.00% | 0.00% |  | 38.33% | 17.70% | 58.96% |  | 23.29% | 10.00% | 36.59% |  | 10.29% | 3.86% | 16.73% |
|  | Intentional injuries | |  |  |  |  |  |  |  |  |  |  |  |  |  |  |  |
|  |  | Self-inflicted injuries | 0.00% | 0.00% | 0.00% |  | 38.33% | 17.70% | 58.96% |  | 23.29% | 10.00% | 36.59% |  | 10.29% | 3.86% | 16.73% |
|  |  | Violence | 0.00% | 0.00% | 0.00% |  | 0.00% | 0.00% | 0.00% |  | 0.00% | 0.00% | 0.00% |  | 0.00% | 0.00% | 0.00% |
|  |  | Other intentional injuries | 0.00% | 0.00% | 0.00% |  | 38.33% | 17.70% | 58.96% |  | 23.29% | 10.00% | 36.59% |  | 10.29% | 3.86% | 16.73% |

Table 38: Mortality Alcohol-Attributable Fractions for injuries (without harms to others included): Oceania

|  |  |  | 0 to 14 years of age | | |  | 15 to 34 years of age | | |  | 35 to 64 years of age | | |  | 65 years of age and older | | |
| --- | --- | --- | --- | --- | --- | --- | --- | --- | --- | --- | --- | --- | --- | --- | --- | --- | --- |
|  |  |  | Point estimate | Lower 95% CI | Upper 95% CI |  | Point estimate | Lower 95% CI | Upper 95% CI |  | Point estimate | Lower 95% CI | Upper 95% CI |  | Point estimate | Lower 95% CI | Upper 95% CI |
| Women | |  |  |  |  |  |  |  |  |  |  |  |  |  |  |  |  |
| Injuries | |  |  |  |  |  |  |  |  |  |  |  |  |  |  |  |  |
|  | Unintentional injuries | |  |  |  |  |  |  |  |  |  |  |  |  |  |  |  |
|  |  | Transport injuries | 0.00% | 0.00% | 0.00% |  | 2.70% | 1.72% | 3.69% |  | 2.11% | 1.31% | 2.90% |  | 0.98% | 0.58% | 1.39% |
|  |  | Poisonings | 0.00% | 0.00% | 0.00% |  | 3.33% | 1.24% | 5.43% |  | 1.81% | 0.46% | 3.17% |  | 0.82% | 0.20% | 1.45% |
|  |  | Falls | 0.00% | 0.00% | 0.00% |  | 3.33% | 1.24% | 5.43% |  | 1.81% | 0.46% | 3.17% |  | 0.82% | 0.20% | 1.45% |
|  |  | Fires, heat and hot substances | 0.00% | 0.00% | 0.00% |  | 3.33% | 1.24% | 5.43% |  | 1.81% | 0.46% | 3.17% |  | 0.82% | 0.20% | 1.45% |
|  |  | Drownings | 0.00% | 0.00% | 0.00% |  | 3.33% | 1.24% | 5.43% |  | 1.81% | 0.46% | 3.17% |  | 0.82% | 0.20% | 1.45% |
|  |  | Other unintentional injuries | 0.00% | 0.00% | 0.00% |  | 3.33% | 1.24% | 5.43% |  | 1.81% | 0.46% | 3.17% |  | 0.82% | 0.20% | 1.45% |
|  | Intentional injuries | |  |  |  |  |  |  |  |  |  |  |  |  |  |  |  |
|  |  | Self-inflicted injuries | 0.00% | 0.00% | 0.00% |  | 3.33% | 1.24% | 5.43% |  | 1.81% | 0.46% | 3.17% |  | 0.82% | 0.20% | 1.45% |
|  |  | Violence | 0.00% | 0.00% | 0.00% |  | 0.00% | 0.00% | 0.00% |  | 0.00% | 0.00% | 0.00% |  | 0.00% | 0.00% | 0.00% |
|  |  | Other intentional injuries | 0.00% | 0.00% | 0.00% |  | 3.33% | 1.24% | 5.43% |  | 1.81% | 0.46% | 3.17% |  | 0.82% | 0.20% | 1.45% |
| Men | |  |  |  |  |  |  |  |  |  |  |  |  |  |  |  |  |
| Injuries | |  |  |  |  |  |  |  |  |  |  |  |  |  |  |  |  |
|  | Unintentional injuries | |  |  |  |  |  |  |  |  |  |  |  |  |  |  |  |
|  |  | Transport injuries | 0.00% | 0.00% | 0.00% |  | 15.91% | 10.11% | 21.72% |  | 12.40% | 7.73% | 17.07% |  | 5.80% | 3.39% | 8.21% |
|  |  | Poisonings | 0.00% | 0.00% | 0.00% |  | 11.32% | 5.38% | 17.27% |  | 10.31% | 4.82% | 15.80% |  | 5.08% | 2.32% | 7.83% |
|  |  | Falls | 0.00% | 0.00% | 0.00% |  | 11.32% | 5.38% | 17.27% |  | 10.31% | 4.82% | 15.80% |  | 5.08% | 2.32% | 7.83% |
|  |  | Fires, heat and hot substances | 0.00% | 0.00% | 0.00% |  | 11.32% | 5.38% | 17.27% |  | 10.31% | 4.82% | 15.80% |  | 5.08% | 2.32% | 7.83% |
|  |  | Drownings | 0.00% | 0.00% | 0.00% |  | 11.32% | 5.38% | 17.27% |  | 10.31% | 4.82% | 15.80% |  | 5.08% | 2.32% | 7.83% |
|  |  | Other unintentional injuries | 0.00% | 0.00% | 0.00% |  | 11.32% | 5.38% | 17.27% |  | 10.31% | 4.82% | 15.80% |  | 5.08% | 2.32% | 7.83% |
|  | Intentional injuries | |  |  |  |  |  |  |  |  |  |  |  |  |  |  |  |
|  |  | Self-inflicted injuries | 0.00% | 0.00% | 0.00% |  | 11.32% | 5.38% | 17.27% |  | 10.31% | 4.82% | 15.80% |  | 5.08% | 2.32% | 7.83% |
|  |  | Violence | 0.00% | 0.00% | 0.00% |  | 0.00% | 0.00% | 0.00% |  | 0.00% | 0.00% | 0.00% |  | 0.00% | 0.00% | 0.00% |
|  |  | Other intentional injuries | 0.00% | 0.00% | 0.00% |  | 11.32% | 5.38% | 17.27% |  | 10.31% | 4.82% | 15.80% |  | 5.08% | 2.32% | 7.83% |

Table 39: Mortality Alcohol-Attributable Fractions for injuries (without harms to others included): Sub-Saharan Africa Central

|  |  |  | 0 to 14 years of age | | |  | 15 to 34 years of age | | |  | 35 to 64 years of age | | |  | 65 years of age and older | | |
| --- | --- | --- | --- | --- | --- | --- | --- | --- | --- | --- | --- | --- | --- | --- | --- | --- | --- |
|  |  |  | Point estimate | Lower 95% CI | Upper 95% CI |  | Point estimate | Lower 95% CI | Upper 95% CI |  | Point estimate | Lower 95% CI | Upper 95% CI |  | Point estimate | Lower 95% CI | Upper 95% CI |
| Women | |  |  |  |  |  |  |  |  |  |  |  |  |  |  |  |  |
| Injuries | |  |  |  |  |  |  |  |  |  |  |  |  |  |  |  |  |
|  | Unintentional injuries | |  |  |  |  |  |  |  |  |  |  |  |  |  |  |  |
|  |  | Transport injuries | 0.00% | 0.00% | 0.00% |  | 4.36% | 2.73% | 5.98% |  | 4.96% | 3.13% | 6.78% |  | 1.37% | 0.78% | 1.96% |
|  |  | Poisonings | 0.00% | 0.00% | 0.00% |  | 4.10% | 1.41% | 6.78% |  | 4.25% | 0.83% | 7.66% |  | 0.92% | 0.24% | 1.61% |
|  |  | Falls | 0.00% | 0.00% | 0.00% |  | 4.10% | 1.41% | 6.78% |  | 4.25% | 0.83% | 7.66% |  | 0.92% | 0.24% | 1.61% |
|  |  | Fires, heat and hot substances | 0.00% | 0.00% | 0.00% |  | 4.10% | 1.41% | 6.78% |  | 4.25% | 0.83% | 7.66% |  | 0.92% | 0.24% | 1.61% |
|  |  | Drownings | 0.00% | 0.00% | 0.00% |  | 4.10% | 1.41% | 6.78% |  | 4.25% | 0.83% | 7.66% |  | 0.92% | 0.24% | 1.61% |
|  |  | Other unintentional injuries | 0.00% | 0.00% | 0.00% |  | 4.10% | 1.41% | 6.78% |  | 4.25% | 0.83% | 7.66% |  | 0.92% | 0.24% | 1.61% |
|  | Intentional injuries | |  |  |  |  |  |  |  |  |  |  |  |  |  |  |  |
|  |  | Self-inflicted injuries | 0.00% | 0.00% | 0.00% |  | 4.10% | 1.41% | 6.78% |  | 4.25% | 0.83% | 7.66% |  | 0.92% | 0.24% | 1.61% |
|  |  | Violence | 0.00% | 0.00% | 0.00% |  | 0.00% | 0.00% | 0.00% |  | 0.00% | 0.00% | 0.00% |  | 0.00% | 0.00% | 0.00% |
|  |  | Other intentional injuries | 0.00% | 0.00% | 0.00% |  | 4.10% | 1.41% | 6.78% |  | 4.25% | 0.83% | 7.66% |  | 0.92% | 0.24% | 1.61% |
| Men | |  |  |  |  |  |  |  |  |  |  |  |  |  |  |  |  |
| Injuries | |  |  |  |  |  |  |  |  |  |  |  |  |  |  |  |  |
|  | Unintentional injuries | |  |  |  |  |  |  |  |  |  |  |  |  |  |  |  |
|  |  | Transport injuries | 0.00% | 0.00% | 0.00% |  | 11.67% | 7.32% | 16.01% |  | 13.26% | 8.38% | 18.15% |  | 3.67% | 2.10% | 5.25% |
|  |  | Poisonings | 0.00% | 0.00% | 0.00% |  | 10.39% | 4.58% | 16.20% |  | 12.00% | 5.05% | 18.96% |  | 3.13% | 1.33% | 4.92% |
|  |  | Falls | 0.00% | 0.00% | 0.00% |  | 10.39% | 4.58% | 16.20% |  | 12.00% | 5.05% | 18.96% |  | 3.13% | 1.33% | 4.92% |
|  |  | Fires, heat and hot substances | 0.00% | 0.00% | 0.00% |  | 10.39% | 4.58% | 16.20% |  | 12.00% | 5.05% | 18.96% |  | 3.13% | 1.33% | 4.92% |
|  |  | Drownings | 0.00% | 0.00% | 0.00% |  | 10.39% | 4.58% | 16.20% |  | 12.00% | 5.05% | 18.96% |  | 3.13% | 1.33% | 4.92% |
|  |  | Other unintentional injuries | 0.00% | 0.00% | 0.00% |  | 10.39% | 4.58% | 16.20% |  | 12.00% | 5.05% | 18.96% |  | 3.13% | 1.33% | 4.92% |
|  | Intentional injuries | |  |  |  |  |  |  |  |  |  |  |  |  |  |  |  |
|  |  | Self-inflicted injuries | 0.00% | 0.00% | 0.00% |  | 10.39% | 4.58% | 16.20% |  | 12.00% | 5.05% | 18.96% |  | 3.13% | 1.33% | 4.92% |
|  |  | Violence | 0.00% | 0.00% | 0.00% |  | 0.00% | 0.00% | 0.00% |  | 0.00% | 0.00% | 0.00% |  | 0.00% | 0.00% | 0.00% |
|  |  | Other intentional injuries | 0.00% | 0.00% | 0.00% |  | 10.39% | 4.58% | 16.20% |  | 12.00% | 5.05% | 18.96% |  | 3.13% | 1.33% | 4.92% |

Table 40: Mortality Alcohol-Attributable Fractions for injuries (without harms to others included): Sub-Saharan Africa East

|  |  |  | 0 to 14 years of age | | |  | 15 to 34 years of age | | |  | 35 to 64 years of age | | |  | 65 years of age and older | | |
| --- | --- | --- | --- | --- | --- | --- | --- | --- | --- | --- | --- | --- | --- | --- | --- | --- | --- |
|  |  |  | Point estimate | Lower 95% CI | Upper 95% CI |  | Point estimate | Lower 95% CI | Upper 95% CI |  | Point estimate | Lower 95% CI | Upper 95% CI |  | Point estimate | Lower 95% CI | Upper 95% CI |
| Women | |  |  |  |  |  |  |  |  |  |  |  |  |  |  |  |  |
| Injuries | |  |  |  |  |  |  |  |  |  |  |  |  |  |  |  |  |
|  | Unintentional injuries | |  |  |  |  |  |  |  |  |  |  |  |  |  |  |  |
|  |  | Transport injuries | 0.00% | 0.00% | 0.00% |  | 1.68% | 0.71% | 2.66% |  | 5.64% | 0.00% | 11.55% |  | 1.44% | 0.59% | 2.28% |
|  |  | Poisonings | 0.00% | 0.00% | 0.00% |  | 2.00% | 0.00% | 5.33% |  | 6.40% | 0.00% | 16.01% |  | 1.83% | 0.00% | 4.62% |
|  |  | Falls | 0.00% | 0.00% | 0.00% |  | 2.00% | 0.00% | 5.33% |  | 6.40% | 0.00% | 16.01% |  | 1.83% | 0.00% | 4.62% |
|  |  | Fires, heat and hot substances | 0.00% | 0.00% | 0.00% |  | 2.00% | 0.00% | 5.33% |  | 6.40% | 0.00% | 16.01% |  | 1.83% | 0.00% | 4.62% |
|  |  | Drownings | 0.00% | 0.00% | 0.00% |  | 2.00% | 0.00% | 5.33% |  | 6.40% | 0.00% | 16.01% |  | 1.83% | 0.00% | 4.62% |
|  |  | Other unintentional injuries | 0.00% | 0.00% | 0.00% |  | 2.00% | 0.00% | 5.33% |  | 6.40% | 0.00% | 16.01% |  | 1.83% | 0.00% | 4.62% |
|  | Intentional injuries | |  |  |  |  |  |  |  |  |  |  |  |  |  |  |  |
|  |  | Self-inflicted injuries | 0.00% | 0.00% | 0.00% |  | 2.00% | 0.00% | 5.33% |  | 6.40% | 0.00% | 16.01% |  | 1.83% | 0.00% | 4.62% |
|  |  | Violence | 0.00% | 0.00% | 0.00% |  | 0.00% | 0.00% | 0.00% |  | 0.00% | 0.00% | 0.00% |  | 0.00% | 0.00% | 0.00% |
|  |  | Other intentional injuries | 0.00% | 0.00% | 0.00% |  | 2.00% | 0.00% | 5.33% |  | 6.40% | 0.00% | 16.01% |  | 1.83% | 0.00% | 4.62% |
| Men | |  |  |  |  |  |  |  |  |  |  |  |  |  |  |  |  |
| Injuries | |  |  |  |  |  |  |  |  |  |  |  |  |  |  |  |  |
|  | Unintentional injuries | |  |  |  |  |  |  |  |  |  |  |  |  |  |  |  |
|  |  | Transport injuries | 0.00% | 0.00% | 0.00% |  | 5.66% | 2.37% | 8.95% |  | 18.97% | 0.00% | 38.81% |  | 4.83% | 1.98% | 7.68% |
|  |  | Poisonings | 0.00% | 0.00% | 0.00% |  | 11.43% | 2.93% | 19.92% |  | 33.49% | 9.93% | 57.05% |  | 12.20% | 2.96% | 21.43% |
|  |  | Falls | 0.00% | 0.00% | 0.00% |  | 11.43% | 2.93% | 19.92% |  | 33.49% | 9.93% | 57.05% |  | 12.20% | 2.96% | 21.43% |
|  |  | Fires, heat and hot substances | 0.00% | 0.00% | 0.00% |  | 11.43% | 2.93% | 19.92% |  | 33.49% | 9.93% | 57.05% |  | 12.20% | 2.96% | 21.43% |
|  |  | Drownings | 0.00% | 0.00% | 0.00% |  | 11.43% | 2.93% | 19.92% |  | 33.49% | 9.93% | 57.05% |  | 12.20% | 2.96% | 21.43% |
|  |  | Other unintentional injuries | 0.00% | 0.00% | 0.00% |  | 11.43% | 2.93% | 19.92% |  | 33.49% | 9.93% | 57.05% |  | 12.20% | 2.96% | 21.43% |
|  | Intentional injuries | |  |  |  |  |  |  |  |  |  |  |  |  |  |  |  |
|  |  | Self-inflicted injuries | 0.00% | 0.00% | 0.00% |  | 11.43% | 2.93% | 19.92% |  | 33.49% | 9.93% | 57.05% |  | 12.20% | 2.96% | 21.43% |
|  |  | Violence | 0.00% | 0.00% | 0.00% |  | 0.00% | 0.00% | 0.00% |  | 0.00% | 0.00% | 0.00% |  | 0.00% | 0.00% | 0.00% |
|  |  | Other intentional injuries | 0.00% | 0.00% | 0.00% |  | 11.43% | 2.93% | 19.92% |  | 33.49% | 9.93% | 57.05% |  | 12.20% | 2.96% | 21.43% |

Table 41: Mortality Alcohol-Attributable Fractions for injuries (without harms to others included): Sub-Saharan Africa Southern

|  |  |  | 0 to 14 years of age | | |  | 15 to 34 years of age | | |  | 35 to 64 years of age | | |  | 65 years of age and older | | |
| --- | --- | --- | --- | --- | --- | --- | --- | --- | --- | --- | --- | --- | --- | --- | --- | --- | --- |
|  |  |  | Point estimate | Lower 95% CI | Upper 95% CI |  | Point estimate | Lower 95% CI | Upper 95% CI |  | Point estimate | Lower 95% CI | Upper 95% CI |  | Point estimate | Lower 95% CI | Upper 95% CI |
| Women | |  |  |  |  |  |  |  |  |  |  |  |  |  |  |  |  |
| Injuries | |  |  |  |  |  |  |  |  |  |  |  |  |  |  |  |  |
|  | Unintentional injuries | |  |  |  |  |  |  |  |  |  |  |  |  |  |  |  |
|  |  | Transport injuries | 0.00% | 0.00% | 0.00% |  | 12.05% | 5.76% | 18.34% |  | 11.53% | 4.90% | 18.16% |  | 1.42% | 0.67% | 2.17% |
|  |  | Poisonings | 0.00% | 0.00% | 0.00% |  | 13.00% | 0.00% | 29.88% |  | 10.28% | 0.00% | 23.77% |  | 1.87% | 0.00% | 4.06% |
|  |  | Falls | 0.00% | 0.00% | 0.00% |  | 13.00% | 0.00% | 29.88% |  | 10.28% | 0.00% | 23.77% |  | 1.87% | 0.00% | 4.06% |
|  |  | Fires, heat and hot substances | 0.00% | 0.00% | 0.00% |  | 13.00% | 0.00% | 29.88% |  | 10.28% | 0.00% | 23.77% |  | 1.87% | 0.00% | 4.06% |
|  |  | Drownings | 0.00% | 0.00% | 0.00% |  | 13.00% | 0.00% | 29.88% |  | 10.28% | 0.00% | 23.77% |  | 1.87% | 0.00% | 4.06% |
|  |  | Other unintentional injuries | 0.00% | 0.00% | 0.00% |  | 13.00% | 0.00% | 29.88% |  | 10.28% | 0.00% | 23.77% |  | 1.87% | 0.00% | 4.06% |
|  | Intentional injuries | |  |  |  |  |  |  |  |  |  |  |  |  |  |  |  |
|  |  | Self-inflicted injuries | 0.00% | 0.00% | 0.00% |  | 13.00% | 0.00% | 29.88% |  | 10.28% | 0.00% | 23.77% |  | 1.87% | 0.00% | 4.06% |
|  |  | Violence | 0.00% | 0.00% | 0.00% |  | 0.00% | 0.00% | 0.00% |  | 0.00% | 0.00% | 0.00% |  | 0.00% | 0.00% | 0.00% |
|  |  | Other intentional injuries | 0.00% | 0.00% | 0.00% |  | 13.00% | 0.00% | 29.88% |  | 10.28% | 0.00% | 23.77% |  | 1.87% | 0.00% | 4.06% |
| Men | |  |  |  |  |  |  |  |  |  |  |  |  |  |  |  |  |
| Injuries | |  |  |  |  |  |  |  |  |  |  |  |  |  |  |  |  |
|  | Unintentional injuries | |  |  |  |  |  |  |  |  |  |  |  |  |  |  |  |
|  |  | Transport injuries | 0.00% | 0.00% | 0.00% |  | 56.00% | 26.78% | 85.22% |  | 53.56% | 22.75% | 84.37% |  | 6.61% | 3.13% | 10.09% |
|  |  | Poisonings | 0.00% | 0.00% | 0.00% |  | 51.35% | 21.79% | 80.91% |  | 51.15% | 21.29% | 81.01% |  | 11.55% | 3.53% | 19.57% |
|  |  | Falls | 0.00% | 0.00% | 0.00% |  | 51.35% | 21.79% | 80.91% |  | 51.15% | 21.29% | 81.01% |  | 11.55% | 3.53% | 19.57% |
|  |  | Fires, heat and hot substances | 0.00% | 0.00% | 0.00% |  | 51.35% | 21.79% | 80.91% |  | 51.15% | 21.29% | 81.01% |  | 11.55% | 3.53% | 19.57% |
|  |  | Drownings | 0.00% | 0.00% | 0.00% |  | 51.35% | 21.79% | 80.91% |  | 51.15% | 21.29% | 81.01% |  | 11.55% | 3.53% | 19.57% |
|  |  | Other unintentional injuries | 0.00% | 0.00% | 0.00% |  | 51.35% | 21.79% | 80.91% |  | 51.15% | 21.29% | 81.01% |  | 11.55% | 3.53% | 19.57% |
|  | Intentional injuries | |  |  |  |  |  |  |  |  |  |  |  |  |  |  |  |
|  |  | Self-inflicted injuries | 0.00% | 0.00% | 0.00% |  | 51.35% | 21.79% | 80.91% |  | 51.15% | 21.29% | 81.01% |  | 11.55% | 3.53% | 19.57% |
|  |  | Violence | 0.00% | 0.00% | 0.00% |  | 0.00% | 0.00% | 0.00% |  | 0.00% | 0.00% | 0.00% |  | 0.00% | 0.00% | 0.00% |
|  |  | Other intentional injuries | 0.00% | 0.00% | 0.00% |  | 51.35% | 21.79% | 80.91% |  | 51.15% | 21.29% | 81.01% |  | 11.55% | 3.53% | 19.57% |

Table 42: Mortality Alcohol-Attributable Fractions for injuries (without harms to others included): Sub-Saharan Africa West

|  |  |  | 0 to 14 years of age | | |  | 15 to 34 years of age | | |  | 35 to 64 years of age | | |  | 65 years of age and older | | |
| --- | --- | --- | --- | --- | --- | --- | --- | --- | --- | --- | --- | --- | --- | --- | --- | --- | --- |
|  |  |  | Point estimate | Lower 95% CI | Upper 95% CI |  | Point estimate | Lower 95% CI | Upper 95% CI |  | Point estimate | Lower 95% CI | Upper 95% CI |  | Point estimate | Lower 95% CI | Upper 95% CI |
| Women | |  |  |  |  |  |  |  |  |  |  |  |  |  |  |  |  |
| Injuries | |  |  |  |  |  |  |  |  |  |  |  |  |  |  |  |  |
|  | Unintentional injuries | |  |  |  |  |  |  |  |  |  |  |  |  |  |  |  |
|  |  | Transport injuries | 0.00% | 0.00% | 0.00% |  | 2.15% | 0.78% | 3.53% |  | 5.70% | 0.51% | 10.88% |  | 1.39% | 0.88% | 1.89% |
|  |  | Poisonings | 0.00% | 0.00% | 0.00% |  | 4.41% | 0.09% | 8.72% |  | 13.71% | 1.39% | 26.04% |  | 3.54% | 0.85% | 6.23% |
|  |  | Falls | 0.00% | 0.00% | 0.00% |  | 4.41% | 0.09% | 8.72% |  | 13.71% | 1.39% | 26.04% |  | 3.54% | 0.85% | 6.23% |
|  |  | Fires, heat and hot substances | 0.00% | 0.00% | 0.00% |  | 4.41% | 0.09% | 8.72% |  | 13.71% | 1.39% | 26.04% |  | 3.54% | 0.85% | 6.23% |
|  |  | Drownings | 0.00% | 0.00% | 0.00% |  | 4.41% | 0.09% | 8.72% |  | 13.71% | 1.39% | 26.04% |  | 3.54% | 0.85% | 6.23% |
|  |  | Other unintentional injuries | 0.00% | 0.00% | 0.00% |  | 4.41% | 0.09% | 8.72% |  | 13.71% | 1.39% | 26.04% |  | 3.54% | 0.85% | 6.23% |
|  | Intentional injuries | |  |  |  |  |  |  |  |  |  |  |  |  |  |  |  |
|  |  | Self-inflicted injuries | 0.00% | 0.00% | 0.00% |  | 4.41% | 0.09% | 8.72% |  | 13.71% | 1.39% | 26.04% |  | 3.54% | 0.85% | 6.23% |
|  |  | Violence | 0.00% | 0.00% | 0.00% |  | 0.00% | 0.00% | 0.00% |  | 0.00% | 0.00% | 0.00% |  | 0.00% | 0.00% | 0.00% |
|  |  | Other intentional injuries | 0.00% | 0.00% | 0.00% |  | 4.41% | 0.09% | 8.72% |  | 13.71% | 1.39% | 26.04% |  | 3.54% | 0.85% | 6.23% |
| Men | |  |  |  |  |  |  |  |  |  |  |  |  |  |  |  |  |
| Injuries | |  |  |  |  |  |  |  |  |  |  |  |  |  |  |  |  |
|  | Unintentional injuries | |  |  |  |  |  |  |  |  |  |  |  |  |  |  |  |
|  |  | Transport injuries | 0.00% | 0.00% | 0.00% |  | 6.39% | 2.31% | 10.47% |  | 16.90% | 1.51% | 32.28% |  | 4.11% | 2.61% | 5.61% |
|  |  | Poisonings | 0.00% | 0.00% | 0.00% |  | 17.33% | 6.01% | 28.64% |  | 36.97% | 14.12% | 59.82% |  | 9.39% | 3.41% | 15.37% |
|  |  | Falls | 0.00% | 0.00% | 0.00% |  | 17.33% | 6.01% | 28.64% |  | 36.97% | 14.12% | 59.82% |  | 9.39% | 3.41% | 15.37% |
|  |  | Fires, heat and hot substances | 0.00% | 0.00% | 0.00% |  | 17.33% | 6.01% | 28.64% |  | 36.97% | 14.12% | 59.82% |  | 9.39% | 3.41% | 15.37% |
|  |  | Drownings | 0.00% | 0.00% | 0.00% |  | 17.33% | 6.01% | 28.64% |  | 36.97% | 14.12% | 59.82% |  | 9.39% | 3.41% | 15.37% |
|  |  | Other unintentional injuries | 0.00% | 0.00% | 0.00% |  | 17.33% | 6.01% | 28.64% |  | 36.97% | 14.12% | 59.82% |  | 9.39% | 3.41% | 15.37% |
|  | Intentional injuries | |  |  |  |  |  |  |  |  |  |  |  |  |  |  |  |
|  |  | Self-inflicted injuries | 0.00% | 0.00% | 0.00% |  | 17.33% | 6.01% | 28.64% |  | 36.97% | 14.12% | 59.82% |  | 9.39% | 3.41% | 15.37% |
|  |  | Violence | 0.00% | 0.00% | 0.00% |  | 0.00% | 0.00% | 0.00% |  | 0.00% | 0.00% | 0.00% |  | 0.00% | 0.00% | 0.00% |
|  |  | Other intentional injuries | 0.00% | 0.00% | 0.00% |  | 17.33% | 6.01% | 28.64% |  | 36.97% | 14.12% | 59.82% |  | 9.39% | 3.41% | 15.37% |
