# Supplementary material for: Global burden of injuries attributable to alcohol consumption in 2004: a novel way of calculating the burden of injuries attributable to alcohol consumption
Source: Popul Health Metr. 2012 May 18;10:9. doi: 10.1186/1478-7954-10-9 (PMC3463441; doi:10.1186/1478-7954-10-9)
Supplement: Additional file 2 — Alcohol-Attributable Fractions for injuries. [file 1478-7954-10-9-S2.docx]

Appendix 2: Alcohol-Attributable Fractions for injuries (with harms to others included)

Table 1: Morbidity Alcohol-Attributable Fractions for injuries (with harms to others included): Asia Pacific [High Income]

|  |  |  | 0 to 14 years of age | | |  | 15 to 34 years of age | | |  | 35 to 64 years of age | | |  | 65 years of age and older | | |
| --- | --- | --- | --- | --- | --- | --- | --- | --- | --- | --- | --- | --- | --- | --- | --- | --- | --- |
|  |  |  | Point estimate | Lower 95% CI | Upper 95% CI |  | Point estimate | Lower 95% CI | Upper 95% CI |  | Point estimate | Lower 95% CI | Upper 95% CI |  | Point estimate | Lower 95% CI | Upper 95% CI |
| Women | | |  |  |  |  |  |  |  |  |  |  |  |  |  |  |  |
| Injuries | | |  |  |  |  |  |  |  |  |  |  |  |  |  |  |  |
|  | Unintentional injuries | |  |  |  |  |  |  |  |  |  |  |  |  |  |  |  |
|  |  | Transport injuries | 2.97% | 2.94% | 3.00% |  | 4.90% | 4.36% | 5.44% |  | 5.37% | 3.83% | 6.91% |  | 1.35% | 1.00% | 1.69% |
|  |  | Poisonings | 0.00% | 0.00% | 0.00% |  | 1.82% | 0.79% | 2.85% |  | 2.83% | 1.09% | 4.57% |  | 0.83% | 0.36% | 1.30% |
|  |  | Falls | 0.00% | 0.00% | 0.00% |  | 1.82% | 0.79% | 2.85% |  | 2.83% | 1.09% | 4.57% |  | 0.83% | 0.36% | 1.30% |
|  |  | Fires, heat and hot substances | 0.00% | 0.00% | 0.00% |  | 1.82% | 0.79% | 2.85% |  | 2.83% | 1.09% | 4.57% |  | 0.83% | 0.36% | 1.30% |
|  |  | Drownings | 0.00% | 0.00% | 0.00% |  | 1.82% | 0.79% | 2.85% |  | 2.83% | 1.09% | 4.57% |  | 0.83% | 0.36% | 1.30% |
|  |  | Other unintentional injuries | 0.00% | 0.00% | 0.00% |  | 1.82% | 0.79% | 2.85% |  | 2.83% | 1.09% | 4.57% |  | 0.83% | 0.36% | 1.30% |
|  | Intentional injuries | |  |  |  |  |  |  |  |  |  |  |  |  |  |  |  |
|  |  | Self-inflicted injuries | 0.00% | 0.00% | 0.00% |  | 1.82% | 0.79% | 2.85% |  | 2.83% | 1.09% | 4.57% |  | 0.83% | 0.36% | 1.30% |
|  |  | Violence | 4.36% | 2.61% | 6.11% |  | 12.51% | 7.49% | 17.52% |  | 7.25% | 4.34% | 10.15% |  | 3.26% | 1.95% | 4.57% |
|  |  | Other intentional injuries | 0.00% | 0.00% | 0.00% |  | 1.82% | 0.79% | 2.85% |  | 2.83% | 1.09% | 4.57% |  | 0.83% | 0.36% | 1.30% |
| Men | |  |  |  |  |  |  |  |  |  |  |  |  |  |  |  |  |
| Injuries | | |  |  |  |  |  |  |  |  |  |  |  |  |  |  |  |
|  | Unintentional injuries | |  |  |  |  |  |  |  |  |  |  |  |  |  |  |  |
|  |  | Transport injuries | 2.97% | 2.62% | 3.33% |  | 7.68% | 5.59% | 9.77% |  | 12.46% | 7.27% | 17.65% |  | 3.24% | 2.10% | 4.39% |
|  |  | Poisonings | 0.00% | 0.00% | 0.00% |  | 7.33% | 3.07% | 11.59% |  | 16.65% | 7.93% | 25.37% |  | 5.58% | 2.24% | 8.91% |
|  |  | Falls | 0.00% | 0.00% | 0.00% |  | 7.33% | 3.07% | 11.59% |  | 16.65% | 7.93% | 25.37% |  | 5.58% | 2.24% | 8.91% |
|  |  | Fires, heat and hot substances | 0.00% | 0.00% | 0.00% |  | 7.33% | 3.07% | 11.59% |  | 16.65% | 7.93% | 25.37% |  | 5.58% | 2.24% | 8.91% |
|  |  | Drownings | 0.00% | 0.00% | 0.00% |  | 7.33% | 3.07% | 11.59% |  | 16.65% | 7.93% | 25.37% |  | 5.58% | 2.24% | 8.91% |
|  |  | Other unintentional injuries | 0.00% | 0.00% | 0.00% |  | 7.33% | 3.07% | 11.59% |  | 16.65% | 7.93% | 25.37% |  | 5.58% | 2.24% | 8.91% |
|  | Intentional injuries | |  |  |  |  |  |  |  |  |  |  |  |  |  |  |  |
|  |  | Self-inflicted injuries | 0.00% | 0.00% | 0.00% |  | 7.33% | 3.07% | 11.59% |  | 16.65% | 7.93% | 25.37% |  | 5.58% | 2.24% | 8.91% |
|  |  | Violence | 4.36% | 2.61% | 6.11% |  | 12.51% | 7.49% | 17.52% |  | 7.25% | 4.34% | 10.15% |  | 3.26% | 1.95% | 4.57% |
|  |  | Other intentional injuries | 0.00% | 0.00% | 0.00% |  | 7.33% | 3.07% | 11.59% |  | 16.65% | 7.93% | 25.37% |  | 5.58% | 2.24% | 8.91% |

Table 2: Morbidity Alcohol-Attributable Fractions for injuries (with harms to others included): Asia Central

|  |  |  | 0 to 14 years of age | | |  | 15 to 34 years of age | | |  | 35 to 64 years of age | | |  | 65 years of age and older | | |
| --- | --- | --- | --- | --- | --- | --- | --- | --- | --- | --- | --- | --- | --- | --- | --- | --- | --- |
|  |  |  | Point estimate | Lower 95% CI | Upper 95% CI |  | Point estimate | Lower 95% CI | Upper 95% CI |  | Point estimate | Lower 95% CI | Upper 95% CI |  | Point estimate | Lower 95% CI | Upper 95% CI |
| Women | | |  |  |  |  |  |  |  |  |  |  |  |  |  |  |  |
| Injuries | | |  |  |  |  |  |  |  |  |  |  |  |  |  |  |  |
|  | Unintentional injuries | |  |  |  |  |  |  |  |  |  |  |  |  |  |  |  |
|  |  | Transport injuries | 7.00% | 6.86% | 7.13% |  | 12.33% | 10.77% | 13.89% |  | 11.08% | 9.11% | 13.04% |  | 4.26% | 3.15% | 5.38% |
|  |  | Poisonings | 0.00% | 0.00% | 0.00% |  | 1.40% | 0.16% | 2.63% |  | 3.37% | 0.48% | 6.26% |  | 0.39% | 0.00% | 0.80% |
|  |  | Falls | 0.00% | 0.00% | 0.00% |  | 1.40% | 0.16% | 2.63% |  | 3.37% | 0.48% | 6.26% |  | 0.39% | 0.00% | 0.80% |
|  |  | Fires, heat and hot substances | 0.00% | 0.00% | 0.00% |  | 1.40% | 0.16% | 2.63% |  | 3.37% | 0.48% | 6.26% |  | 0.39% | 0.00% | 0.80% |
|  |  | Drownings | 0.00% | 0.00% | 0.00% |  | 1.40% | 0.16% | 2.63% |  | 3.37% | 0.48% | 6.26% |  | 0.39% | 0.00% | 0.80% |
|  |  | Other unintentional injuries | 0.00% | 0.00% | 0.00% |  | 1.40% | 0.16% | 2.63% |  | 3.37% | 0.48% | 6.26% |  | 0.39% | 0.00% | 0.80% |
|  | Intentional injuries | |  |  |  |  |  |  |  |  |  |  |  |  |  |  |  |
|  |  | Self-inflicted injuries | 0.00% | 0.00% | 0.00% |  | 1.40% | 0.16% | 2.63% |  | 3.37% | 0.48% | 6.26% |  | 0.39% | 0.00% | 0.80% |
|  |  | Violence | 3.78% | 2.39% | 5.17% |  | 10.84% | 6.86% | 14.81% |  | 6.28% | 3.97% | 8.59% |  | 2.83% | 1.79% | 3.86% |
|  |  | Other intentional injuries | 0.00% | 0.00% | 0.00% |  | 1.40% | 0.16% | 2.63% |  | 3.37% | 0.48% | 6.26% |  | 0.39% | 0.00% | 0.80% |
| Men | |  |  |  |  |  |  |  |  |  |  |  |  |  |  |  |  |
| Injuries | | |  |  |  |  |  |  |  |  |  |  |  |  |  |  |  |
|  | Unintentional injuries | |  |  |  |  |  |  |  |  |  |  |  |  |  |  |  |
|  |  | Transport injuries | 7.00% | 5.44% | 8.55% |  | 20.79% | 14.47% | 27.11% |  | 24.07% | 17.02% | 31.12% |  | 11.18% | 7.45% | 14.91% |
|  |  | Poisonings | 0.00% | 0.00% | 0.00% |  | 9.73% | 3.90% | 15.56% |  | 11.78% | 5.46% | 18.09% |  | 5.71% | 2.48% | 8.95% |
|  |  | Falls | 0.00% | 0.00% | 0.00% |  | 9.73% | 3.90% | 15.56% |  | 11.78% | 5.46% | 18.09% |  | 5.71% | 2.48% | 8.95% |
|  |  | Fires, heat and hot substances | 0.00% | 0.00% | 0.00% |  | 9.73% | 3.90% | 15.56% |  | 11.78% | 5.46% | 18.09% |  | 5.71% | 2.48% | 8.95% |
|  |  | Drownings | 0.00% | 0.00% | 0.00% |  | 9.73% | 3.90% | 15.56% |  | 11.78% | 5.46% | 18.09% |  | 5.71% | 2.48% | 8.95% |
|  |  | Other unintentional injuries | 0.00% | 0.00% | 0.00% |  | 9.73% | 3.90% | 15.56% |  | 11.78% | 5.46% | 18.09% |  | 5.71% | 2.48% | 8.95% |
|  | Intentional injuries | |  |  |  |  |  |  |  |  |  |  |  |  |  |  |  |
|  |  | Self-inflicted injuries | 0.00% | 0.00% | 0.00% |  | 9.73% | 3.90% | 15.56% |  | 11.78% | 5.46% | 18.09% |  | 5.71% | 2.48% | 8.95% |
|  |  | Violence | 3.78% | 2.39% | 5.17% |  | 10.84% | 6.86% | 14.81% |  | 6.28% | 3.97% | 8.59% |  | 2.83% | 1.79% | 3.86% |
|  |  | Other intentional injuries | 0.00% | 0.00% | 0.00% |  | 9.73% | 3.90% | 15.56% |  | 11.78% | 5.46% | 18.09% |  | 5.71% | 2.48% | 8.95% |

Table 3: Morbidity Alcohol-Attributable Fractions for injuries (with harms to others included): Asia East

|  |  |  | 0 to 14 years of age | | |  | 15 to 34 years of age | | |  | 35 to 64 years of age | | |  | 65 years of age and older | | |
| --- | --- | --- | --- | --- | --- | --- | --- | --- | --- | --- | --- | --- | --- | --- | --- | --- | --- |
|  |  |  | Point estimate | Lower 95% CI | Upper 95% CI |  | Point estimate | Lower 95% CI | Upper 95% CI |  | Point estimate | Lower 95% CI | Upper 95% CI |  | Point estimate | Lower 95% CI | Upper 95% CI |
| Women | | |  |  |  |  |  |  |  |  |  |  |  |  |  |  |  |
| Injuries | | |  |  |  |  |  |  |  |  |  |  |  |  |  |  |  |
|  | Unintentional injuries | |  |  |  |  |  |  |  |  |  |  |  |  |  |  |  |
|  |  | Transport injuries | 1.56% | 1.55% | 1.56% |  | 2.29% | 2.12% | 2.45% |  | 2.49% | 1.76% | 3.22% |  | 0.78% | 0.51% | 1.04% |
|  |  | Poisonings | 0.00% | 0.00% | 0.00% |  | 0.27% | 0.04% | 0.49% |  | 1.32% | 0.00% | 3.02% |  | 0.54% | 0.00% | 1.09% |
|  |  | Falls | 0.00% | 0.00% | 0.00% |  | 0.27% | 0.04% | 0.49% |  | 1.32% | 0.00% | 3.02% |  | 0.54% | 0.00% | 1.09% |
|  |  | Fires, heat and hot substances | 0.00% | 0.00% | 0.00% |  | 0.27% | 0.04% | 0.49% |  | 1.32% | 0.00% | 3.02% |  | 0.54% | 0.00% | 1.09% |
|  |  | Drownings | 0.00% | 0.00% | 0.00% |  | 0.27% | 0.04% | 0.49% |  | 1.32% | 0.00% | 3.02% |  | 0.54% | 0.00% | 1.09% |
|  |  | Other unintentional injuries | 0.00% | 0.00% | 0.00% |  | 0.27% | 0.04% | 0.49% |  | 1.32% | 0.00% | 3.02% |  | 0.54% | 0.00% | 1.09% |
|  | Intentional injuries | |  |  |  |  |  |  |  |  |  |  |  |  |  |  |  |
|  |  | Self-inflicted injuries | 0.00% | 0.00% | 0.00% |  | 0.27% | 0.04% | 0.49% |  | 1.32% | 0.00% | 3.02% |  | 0.54% | 0.00% | 1.09% |
|  |  | Violence | 2.61% | 1.24% | 3.98% |  | 7.47% | 3.55% | 11.39% |  | 4.33% | 2.06% | 6.60% |  | 1.95% | 0.93% | 2.97% |
|  |  | Other intentional injuries | 0.00% | 0.00% | 0.00% |  | 0.27% | 0.04% | 0.49% |  | 1.32% | 0.00% | 3.02% |  | 0.54% | 0.00% | 1.09% |
| Men | |  |  |  |  |  |  |  |  |  |  |  |  |  |  |  |  |
| Injuries | | |  |  |  |  |  |  |  |  |  |  |  |  |  |  |  |
|  | Unintentional injuries | |  |  |  |  |  |  |  |  |  |  |  |  |  |  |  |
|  |  | Transport injuries | 1.56% | 1.45% | 1.67% |  | 3.73% | 2.81% | 4.65% |  | 7.77% | 4.05% | 11.49% |  | 2.84% | 1.50% | 4.17% |
|  |  | Poisonings | 0.00% | 0.00% | 0.00% |  | 2.28% | 1.04% | 3.52% |  | 11.66% | 4.42% | 18.90% |  | 5.92% | 1.91% | 9.92% |
|  |  | Falls | 0.00% | 0.00% | 0.00% |  | 2.28% | 1.04% | 3.52% |  | 11.66% | 4.42% | 18.90% |  | 5.92% | 1.91% | 9.92% |
|  |  | Fires, heat and hot substances | 0.00% | 0.00% | 0.00% |  | 2.28% | 1.04% | 3.52% |  | 11.66% | 4.42% | 18.90% |  | 5.92% | 1.91% | 9.92% |
|  |  | Drownings | 0.00% | 0.00% | 0.00% |  | 2.28% | 1.04% | 3.52% |  | 11.66% | 4.42% | 18.90% |  | 5.92% | 1.91% | 9.92% |
|  |  | Other unintentional injuries | 0.00% | 0.00% | 0.00% |  | 2.28% | 1.04% | 3.52% |  | 11.66% | 4.42% | 18.90% |  | 5.92% | 1.91% | 9.92% |
|  | Intentional injuries | |  |  |  |  |  |  |  |  |  |  |  |  |  |  |  |
|  |  | Self-inflicted injuries | 0.00% | 0.00% | 0.00% |  | 2.28% | 1.04% | 3.52% |  | 11.66% | 4.42% | 18.90% |  | 5.92% | 1.91% | 9.92% |
|  |  | Violence | 2.61% | 1.24% | 3.98% |  | 7.47% | 3.55% | 11.39% |  | 4.33% | 2.06% | 6.60% |  | 1.95% | 0.93% | 2.97% |
|  |  | Other intentional injuries | 0.00% | 0.00% | 0.00% |  | 2.28% | 1.04% | 3.52% |  | 11.66% | 4.42% | 18.90% |  | 5.92% | 1.91% | 9.92% |

Table 4: Morbidity Alcohol-Attributable Fractions for injuries (with harms to others included): Asia South

|  |  |  | 0 to 14 years of age | | |  | 15 to 34 years of age | | |  | 35 to 64 years of age | | |  | 65 years of age and older | | |
| --- | --- | --- | --- | --- | --- | --- | --- | --- | --- | --- | --- | --- | --- | --- | --- | --- | --- |
|  |  |  | Point estimate | Lower 95% CI | Upper 95% CI |  | Point estimate | Lower 95% CI | Upper 95% CI |  | Point estimate | Lower 95% CI | Upper 95% CI |  | Point estimate | Lower 95% CI | Upper 95% CI |
| Women | | |  |  |  |  |  |  |  |  |  |  |  |  |  |  |  |
| Injuries | | |  |  |  |  |  |  |  |  |  |  |  |  |  |  |  |
|  | Unintentional injuries | |  |  |  |  |  |  |  |  |  |  |  |  |  |  |  |
|  |  | Transport injuries | 1.92% | 1.90% | 1.94% |  | 2.57% | 2.44% | 2.69% |  | 1.96% | 1.58% | 2.35% |  | 0.50% | 0.33% | 0.68% |
|  |  | Poisonings | 0.00% | 0.00% | 0.00% |  | 0.05% | 0.00% | 1.16% |  | 0.47% | 0.00% | 8.75% |  | 0.01% | 0.00% | 1.24% |
|  |  | Falls | 0.00% | 0.00% | 0.00% |  | 0.05% | 0.00% | 1.16% |  | 0.47% | 0.00% | 8.75% |  | 0.01% | 0.00% | 1.24% |
|  |  | Fires, heat and hot substances | 0.00% | 0.00% | 0.00% |  | 0.05% | 0.00% | 1.16% |  | 0.47% | 0.00% | 8.75% |  | 0.01% | 0.00% | 1.24% |
|  |  | Drownings | 0.00% | 0.00% | 0.00% |  | 0.05% | 0.00% | 1.16% |  | 0.47% | 0.00% | 8.75% |  | 0.01% | 0.00% | 1.24% |
|  |  | Other unintentional injuries | 0.00% | 0.00% | 0.00% |  | 0.05% | 0.00% | 1.16% |  | 0.47% | 0.00% | 8.75% |  | 0.01% | 0.00% | 1.24% |
|  | Intentional injuries | |  |  |  |  |  |  |  |  |  |  |  |  |  |  |  |
|  |  | Self-inflicted injuries | 0.00% | 0.00% | 0.00% |  | 0.05% | 0.00% | 1.16% |  | 0.47% | 0.00% | 8.75% |  | 0.01% | 0.00% | 1.24% |
|  |  | Violence | 1.49% | 0.00% | 7.07% |  | 4.27% | 0.00% | 20.26% |  | 2.47% | 0.00% | 11.74% |  | 1.11% | 0.00% | 5.28% |
|  |  | Other intentional injuries | 0.00% | 0.00% | 0.00% |  | 0.05% | 0.00% | 1.16% |  | 0.47% | 0.00% | 8.75% |  | 0.01% | 0.00% | 1.24% |
| Men | |  |  |  |  |  |  |  |  |  |  |  |  |  |  |  |  |
| Injuries | | |  |  |  |  |  |  |  |  |  |  |  |  |  |  |  |
|  | Unintentional injuries | |  |  |  |  |  |  |  |  |  |  |  |  |  |  |  |
|  |  | Transport injuries | 1.92% | 1.72% | 2.12% |  | 5.28% | 3.47% | 7.09% |  | 9.20% | 3.23% | 15.16% |  | 3.02% | 0.31% | 5.73% |
|  |  | Poisonings | 0.00% | 0.00% | 0.00% |  | 2.62% | 0.39% | 4.84% |  | 6.97% | 1.01% | 12.94% |  | 3.34% | 0.01% | 6.68% |
|  |  | Falls | 0.00% | 0.00% | 0.00% |  | 2.62% | 0.39% | 4.84% |  | 6.97% | 1.01% | 12.94% |  | 3.34% | 0.01% | 6.68% |
|  |  | Fires, heat and hot substances | 0.00% | 0.00% | 0.00% |  | 2.62% | 0.39% | 4.84% |  | 6.97% | 1.01% | 12.94% |  | 3.34% | 0.01% | 6.68% |
|  |  | Drownings | 0.00% | 0.00% | 0.00% |  | 2.62% | 0.39% | 4.84% |  | 6.97% | 1.01% | 12.94% |  | 3.34% | 0.01% | 6.68% |
|  |  | Other unintentional injuries | 0.00% | 0.00% | 0.00% |  | 2.62% | 0.39% | 4.84% |  | 6.97% | 1.01% | 12.94% |  | 3.34% | 0.01% | 6.68% |
|  | Intentional injuries | |  |  |  |  |  |  |  |  |  |  |  |  |  |  |  |
|  |  | Self-inflicted injuries | 0.00% | 0.00% | 0.00% |  | 2.62% | 0.39% | 4.84% |  | 6.97% | 1.01% | 12.94% |  | 3.34% | 0.01% | 6.68% |
|  |  | Violence | 1.49% | 0.00% | 7.07% |  | 4.27% | 0.00% | 20.26% |  | 2.47% | 0.00% | 11.74% |  | 1.11% | 0.00% | 5.28% |
|  |  | Other intentional injuries | 0.00% | 0.00% | 0.00% |  | 2.62% | 0.39% | 4.84% |  | 6.97% | 1.01% | 12.94% |  | 3.34% | 0.01% | 6.68% |

Table 5: Morbidity Alcohol-Attributable Fractions for injuries (with harms to others included): Asia Southeast

|  |  |  | 0 to 14 years of age | | |  | 15 to 34 years of age | | |  | 35 to 64 years of age | | |  | 65 years of age and older | | |
| --- | --- | --- | --- | --- | --- | --- | --- | --- | --- | --- | --- | --- | --- | --- | --- | --- | --- |
|  |  |  | Point estimate | Lower 95% CI | Upper 95% CI |  | Point estimate | Lower 95% CI | Upper 95% CI |  | Point estimate | Lower 95% CI | Upper 95% CI |  | Point estimate | Lower 95% CI | Upper 95% CI |
| Women | | |  |  |  |  |  |  |  |  |  |  |  |  |  |  |  |
| Injuries | | |  |  |  |  |  |  |  |  |  |  |  |  |  |  |  |
|  | Unintentional injuries | |  |  |  |  |  |  |  |  |  |  |  |  |  |  |  |
|  |  | Transport injuries | 1.24% | 1.23% | 1.25% |  | 1.77% | 1.66% | 1.87% |  | 1.57% | 0.94% | 2.44% |  | 0.31% | 0.22% | 0.40% |
|  |  | Poisonings | 0.00% | 0.00% | 0.00% |  | 0.20% | 0.00% | 0.79% |  | 0.49% | 0.00% | 2.22% |  | 0.02% | 0.00% | 0.08% |
|  |  | Falls | 0.00% | 0.00% | 0.00% |  | 0.20% | 0.00% | 0.79% |  | 0.49% | 0.00% | 2.22% |  | 0.02% | 0.00% | 0.08% |
|  |  | Fires, heat and hot substances | 0.00% | 0.00% | 0.00% |  | 0.20% | 0.00% | 0.79% |  | 0.49% | 0.00% | 2.22% |  | 0.02% | 0.00% | 0.08% |
|  |  | Drownings | 0.00% | 0.00% | 0.00% |  | 0.20% | 0.00% | 0.79% |  | 0.49% | 0.00% | 2.22% |  | 0.02% | 0.00% | 0.08% |
|  |  | Other unintentional injuries | 0.00% | 0.00% | 0.00% |  | 0.20% | 0.00% | 0.79% |  | 0.49% | 0.00% | 2.22% |  | 0.02% | 0.00% | 0.08% |
|  | Intentional injuries | |  |  |  |  |  |  |  |  |  |  |  |  |  |  |  |
|  |  | Self-inflicted injuries | 0.00% | 0.00% | 0.00% |  | 0.20% | 0.00% | 0.79% |  | 0.49% | 0.00% | 2.22% |  | 0.02% | 0.00% | 0.08% |
|  |  | Violence | 1.82% | 0.67% | 2.96% |  | 5.21% | 1.93% | 8.50% |  | 3.02% | 1.12% | 4.92% |  | 1.36% | 0.50% | 2.22% |
|  |  | Other intentional injuries | 0.00% | 0.00% | 0.00% |  | 0.20% | 0.00% | 0.79% |  | 0.49% | 0.00% | 2.22% |  | 0.02% | 0.00% | 0.08% |
| Men | |  |  |  |  |  |  |  |  |  |  |  |  |  |  |  |  |
| Injuries | | |  |  |  |  |  |  |  |  |  |  |  |  |  |  |  |
|  | Unintentional injuries | |  |  |  |  |  |  |  |  |  |  |  |  |  |  |  |
|  |  | Transport injuries | 1.24% | 1.13% | 1.35% |  | 3.99% | 2.82% | 5.15% |  | 7.83% | 0.83% | 17.43% |  | 1.26% | 0.25% | 2.28% |
|  |  | Poisonings | 0.00% | 0.00% | 0.00% |  | 2.34% | 0.70% | 3.97% |  | 9.68% | 2.55% | 16.81% |  | 2.97% | 0.62% | 5.33% |
|  |  | Falls | 0.00% | 0.00% | 0.00% |  | 2.34% | 0.70% | 3.97% |  | 9.68% | 2.55% | 16.81% |  | 2.97% | 0.62% | 5.33% |
|  |  | Fires, heat and hot substances | 0.00% | 0.00% | 0.00% |  | 2.34% | 0.70% | 3.97% |  | 9.68% | 2.55% | 16.81% |  | 2.97% | 0.62% | 5.33% |
|  |  | Drownings | 0.00% | 0.00% | 0.00% |  | 2.34% | 0.70% | 3.97% |  | 9.68% | 2.55% | 16.81% |  | 2.97% | 0.62% | 5.33% |
|  |  | Other unintentional injuries | 0.00% | 0.00% | 0.00% |  | 2.34% | 0.70% | 3.97% |  | 9.68% | 2.55% | 16.81% |  | 2.97% | 0.62% | 5.33% |
|  | Intentional injuries | |  |  |  |  |  |  |  |  |  |  |  |  |  |  |  |
|  |  | Self-inflicted injuries | 0.00% | 0.00% | 0.00% |  | 2.34% | 0.70% | 3.97% |  | 9.68% | 2.55% | 16.81% |  | 2.97% | 0.62% | 5.33% |
|  |  | Violence | 1.82% | 0.67% | 2.96% |  | 5.21% | 1.93% | 8.50% |  | 3.02% | 1.12% | 4.92% |  | 1.36% | 0.50% | 2.22% |
|  |  | Other intentional injuries | 0.00% | 0.00% | 0.00% |  | 2.34% | 0.70% | 3.97% |  | 9.68% | 2.55% | 16.81% |  | 2.97% | 0.62% | 5.33% |

Table 6: Morbidity Alcohol-Attributable Fractions for injuries (with harms to others included): Australasia

|  |  |  | 0 to 14 years of age | | |  | 15 to 34 years of age | | |  | 35 to 64 years of age | | |  | 65 years of age and older | | |
| --- | --- | --- | --- | --- | --- | --- | --- | --- | --- | --- | --- | --- | --- | --- | --- | --- | --- |
|  |  |  | Point estimate | Lower 95% CI | Upper 95% CI |  | Point estimate | Lower 95% CI | Upper 95% CI |  | Point estimate | Lower 95% CI | Upper 95% CI |  | Point estimate | Lower 95% CI | Upper 95% CI |
| Women | | |  |  |  |  |  |  |  |  |  |  |  |  |  |  |  |
| Injuries | | |  |  |  |  |  |  |  |  |  |  |  |  |  |  |  |
|  | Unintentional injuries | |  |  |  |  |  |  |  |  |  |  |  |  |  |  |  |
|  |  | Transport injuries | 2.38% | 2.37% | 2.38% |  | 4.81% | 4.07% | 5.55% |  | 4.05% | 3.02% | 5.09% |  | 1.28% | 0.81% | 1.75% |
|  |  | Poisonings | 0.00% | 0.00% | 0.00% |  | 3.04% | 1.46% | 4.62% |  | 2.84% | 1.33% | 4.36% |  | 1.35% | 0.63% | 2.06% |
|  |  | Falls | 0.00% | 0.00% | 0.00% |  | 3.04% | 1.46% | 4.62% |  | 2.84% | 1.33% | 4.36% |  | 1.35% | 0.63% | 2.06% |
|  |  | Fires, heat and hot substances | 0.00% | 0.00% | 0.00% |  | 3.04% | 1.46% | 4.62% |  | 2.84% | 1.33% | 4.36% |  | 1.35% | 0.63% | 2.06% |
|  |  | Drownings | 0.00% | 0.00% | 0.00% |  | 3.04% | 1.46% | 4.62% |  | 2.84% | 1.33% | 4.36% |  | 1.35% | 0.63% | 2.06% |
|  |  | Other unintentional injuries | 0.00% | 0.00% | 0.00% |  | 3.04% | 1.46% | 4.62% |  | 2.84% | 1.33% | 4.36% |  | 1.35% | 0.63% | 2.06% |
|  | Intentional injuries | |  |  |  |  |  |  |  |  |  |  |  |  |  |  |  |
|  |  | Self-inflicted injuries | 0.00% | 0.00% | 0.00% |  | 3.04% | 1.46% | 4.62% |  | 2.84% | 1.33% | 4.36% |  | 1.35% | 0.63% | 2.06% |
|  |  | Violence | 3.97% | 2.72% | 5.21% |  | 11.37% | 7.81% | 14.94% |  | 6.59% | 4.52% | 8.66% |  | 2.97% | 2.04% | 3.90% |
|  |  | Other intentional injuries | 0.00% | 0.00% | 0.00% |  | 3.04% | 1.46% | 4.62% |  | 2.84% | 1.33% | 4.36% |  | 1.35% | 0.63% | 2.06% |
| Men | |  |  |  |  |  |  |  |  |  |  |  |  |  |  |  |  |
| Injuries | | |  |  |  |  |  |  |  |  |  |  |  |  |  |  |  |
|  | Unintentional injuries | |  |  |  |  |  |  |  |  |  |  |  |  |  |  |  |
|  |  | Transport injuries | 2.38% | 2.33% | 2.43% |  | 7.55% | 5.67% | 9.43% |  | 7.34% | 4.76% | 9.93% |  | 2.57% | 1.40% | 3.73% |
|  |  | Poisonings | 0.00% | 0.00% | 0.00% |  | 8.63% | 4.02% | 13.23% |  | 12.03% | 5.79% | 18.28% |  | 7.29% | 3.22% | 11.35% |
|  |  | Falls | 0.00% | 0.00% | 0.00% |  | 8.63% | 4.02% | 13.23% |  | 12.03% | 5.79% | 18.28% |  | 7.29% | 3.22% | 11.35% |
|  |  | Fires, heat and hot substances | 0.00% | 0.00% | 0.00% |  | 8.63% | 4.02% | 13.23% |  | 12.03% | 5.79% | 18.28% |  | 7.29% | 3.22% | 11.35% |
|  |  | Drownings | 0.00% | 0.00% | 0.00% |  | 8.63% | 4.02% | 13.23% |  | 12.03% | 5.79% | 18.28% |  | 7.29% | 3.22% | 11.35% |
|  |  | Other unintentional injuries | 0.00% | 0.00% | 0.00% |  | 8.63% | 4.02% | 13.23% |  | 12.03% | 5.79% | 18.28% |  | 7.29% | 3.22% | 11.35% |
|  | Intentional injuries | |  |  |  |  |  |  |  |  |  |  |  |  |  |  |  |
|  |  | Self-inflicted injuries | 0.00% | 0.00% | 0.00% |  | 8.63% | 4.02% | 13.23% |  | 12.03% | 5.79% | 18.28% |  | 7.29% | 3.22% | 11.35% |
|  |  | Violence | 3.97% | 2.72% | 5.21% |  | 11.37% | 7.81% | 14.94% |  | 6.59% | 4.52% | 8.66% |  | 2.97% | 2.04% | 3.90% |
|  |  | Other intentional injuries | 0.00% | 0.00% | 0.00% |  | 8.63% | 4.02% | 13.23% |  | 12.03% | 5.79% | 18.28% |  | 7.29% | 3.22% | 11.35% |

Table 7: Morbidity Alcohol-Attributable Fractions for injuries (with harms to others included): Caribbean

|  |  |  | 0 to 14 years of age | | |  | 15 to 34 years of age | | |  | 35 to 64 years of age | | |  | 65 years of age and older | | |
| --- | --- | --- | --- | --- | --- | --- | --- | --- | --- | --- | --- | --- | --- | --- | --- | --- | --- |
|  |  |  | Point estimate | Lower 95% CI | Upper 95% CI |  | Point estimate | Lower 95% CI | Upper 95% CI |  | Point estimate | Lower 95% CI | Upper 95% CI |  | Point estimate | Lower 95% CI | Upper 95% CI |
| Women | | |  |  |  |  |  |  |  |  |  |  |  |  |  |  |  |
| Injuries | | |  |  |  |  |  |  |  |  |  |  |  |  |  |  |  |
|  | Unintentional injuries | |  |  |  |  |  |  |  |  |  |  |  |  |  |  |  |
|  |  | Transport injuries | 2.09% | 2.08% | 2.11% |  | 3.96% | 3.40% | 4.52% |  | 3.06% | 2.45% | 3.67% |  | 0.78% | 0.58% | 0.99% |
|  |  | Poisonings | 0.00% | 0.00% | 0.00% |  | 2.54% | 0.33% | 4.75% |  | 0.70% | 0.23% | 1.16% |  | 0.11% | 0.03% | 0.19% |
|  |  | Falls | 0.00% | 0.00% | 0.00% |  | 2.54% | 0.33% | 4.75% |  | 0.70% | 0.23% | 1.16% |  | 0.11% | 0.03% | 0.19% |
|  |  | Fires, heat and hot substances | 0.00% | 0.00% | 0.00% |  | 2.54% | 0.33% | 4.75% |  | 0.70% | 0.23% | 1.16% |  | 0.11% | 0.03% | 0.19% |
|  |  | Drownings | 0.00% | 0.00% | 0.00% |  | 2.54% | 0.33% | 4.75% |  | 0.70% | 0.23% | 1.16% |  | 0.11% | 0.03% | 0.19% |
|  |  | Other unintentional injuries | 0.00% | 0.00% | 0.00% |  | 2.54% | 0.33% | 4.75% |  | 0.70% | 0.23% | 1.16% |  | 0.11% | 0.03% | 0.19% |
|  | Intentional injuries | |  |  |  |  |  |  |  |  |  |  |  |  |  |  |  |
|  |  | Self-inflicted injuries | 0.00% | 0.00% | 0.00% |  | 2.54% | 0.33% | 4.75% |  | 0.70% | 0.23% | 1.16% |  | 0.11% | 0.03% | 0.19% |
|  |  | Violence | 2.41% | 1.53% | 3.29% |  | 6.90% | 4.39% | 9.42% |  | 4.00% | 2.54% | 5.46% |  | 1.80% | 1.15% | 2.46% |
|  |  | Other intentional injuries | 0.00% | 0.00% | 0.00% |  | 2.54% | 0.33% | 4.75% |  | 0.70% | 0.23% | 1.16% |  | 0.11% | 0.03% | 0.19% |
| Men | |  |  |  |  |  |  |  |  |  |  |  |  |  |  |  |  |
| Injuries | | |  |  |  |  |  |  |  |  |  |  |  |  |  |  |  |
|  | Unintentional injuries | |  |  |  |  |  |  |  |  |  |  |  |  |  |  |  |
|  |  | Transport injuries | 2.09% | 1.93% | 2.25% |  | 7.25% | 5.19% | 9.30% |  | 6.57% | 4.42% | 8.71% |  | 1.80% | 1.09% | 2.51% |
|  |  | Poisonings | 0.00% | 0.00% | 0.00% |  | 5.74% | 2.33% | 9.15% |  | 7.76% | 3.19% | 12.34% |  | 3.39% | 1.20% | 5.58% |
|  |  | Falls | 0.00% | 0.00% | 0.00% |  | 5.74% | 2.33% | 9.15% |  | 7.76% | 3.19% | 12.34% |  | 3.39% | 1.20% | 5.58% |
|  |  | Fires, heat and hot substances | 0.00% | 0.00% | 0.00% |  | 5.74% | 2.33% | 9.15% |  | 7.76% | 3.19% | 12.34% |  | 3.39% | 1.20% | 5.58% |
|  |  | Drownings | 0.00% | 0.00% | 0.00% |  | 5.74% | 2.33% | 9.15% |  | 7.76% | 3.19% | 12.34% |  | 3.39% | 1.20% | 5.58% |
|  |  | Other unintentional injuries | 0.00% | 0.00% | 0.00% |  | 5.74% | 2.33% | 9.15% |  | 7.76% | 3.19% | 12.34% |  | 3.39% | 1.20% | 5.58% |
|  | Intentional injuries | |  |  |  |  |  |  |  |  |  |  |  |  |  |  |  |
|  |  | Self-inflicted injuries | 0.00% | 0.00% | 0.00% |  | 5.74% | 2.33% | 9.15% |  | 7.76% | 3.19% | 12.34% |  | 3.39% | 1.20% | 5.58% |
|  |  | Violence | 2.41% | 1.53% | 3.29% |  | 6.90% | 4.39% | 9.42% |  | 4.00% | 2.54% | 5.46% |  | 1.80% | 1.15% | 2.46% |
|  |  | Other intentional injuries | 0.00% | 0.00% | 0.00% |  | 5.74% | 2.33% | 9.15% |  | 7.76% | 3.19% | 12.34% |  | 3.39% | 1.20% | 5.58% |

Table 8: Morbidity Alcohol-Attributable Fractions for injuries (with harms to others included): Europe Central

|  |  |  | 0 to 14 years of age | | |  | 15 to 34 years of age | | |  | 35 to 64 years of age | | |  | 65 years of age and older | | |
| --- | --- | --- | --- | --- | --- | --- | --- | --- | --- | --- | --- | --- | --- | --- | --- | --- | --- |
|  |  |  | Point estimate | Lower 95% CI | Upper 95% CI |  | Point estimate | Lower 95% CI | Upper 95% CI |  | Point estimate | Lower 95% CI | Upper 95% CI |  | Point estimate | Lower 95% CI | Upper 95% CI |
| Women | | |  |  |  |  |  |  |  |  |  |  |  |  |  |  |  |
| Injuries | | |  |  |  |  |  |  |  |  |  |  |  |  |  |  |  |
|  | Unintentional injuries | |  |  |  |  |  |  |  |  |  |  |  |  |  |  |  |
|  |  | Transport injuries | 9.02% | 8.72% | 9.31% |  | 15.47% | 13.19% | 17.75% |  | 14.65% | 8.64% | 20.66% |  | 5.16% | 2.44% | 7.88% |
|  |  | Poisonings | 0.00% | 0.00% | 0.00% |  | 5.09% | 0.07% | 10.12% |  | 6.18% | 0.23% | 12.12% |  | 0.45% | 0.09% | 0.82% |
|  |  | Falls | 0.00% | 0.00% | 0.00% |  | 5.09% | 0.07% | 10.12% |  | 6.18% | 0.23% | 12.12% |  | 0.45% | 0.09% | 0.82% |
|  |  | Fires, heat and hot substances | 0.00% | 0.00% | 0.00% |  | 5.09% | 0.07% | 10.12% |  | 6.18% | 0.23% | 12.12% |  | 0.45% | 0.09% | 0.82% |
|  |  | Drownings | 0.00% | 0.00% | 0.00% |  | 5.09% | 0.07% | 10.12% |  | 6.18% | 0.23% | 12.12% |  | 0.45% | 0.09% | 0.82% |
|  |  | Other unintentional injuries | 0.00% | 0.00% | 0.00% |  | 5.09% | 0.07% | 10.12% |  | 6.18% | 0.23% | 12.12% |  | 0.45% | 0.09% | 0.82% |
|  | Intentional injuries | |  |  |  |  |  |  |  |  |  |  |  |  |  |  |  |
|  |  | Self-inflicted injuries | 0.00% | 0.00% | 0.00% |  | 5.09% | 0.07% | 10.12% |  | 6.18% | 0.23% | 12.12% |  | 0.45% | 0.09% | 0.82% |
|  |  | Violence | 9.11% | 5.43% | 12.79% |  | 26.11% | 15.56% | 36.66% |  | 15.13% | 9.02% | 21.25% |  | 6.81% | 4.06% | 9.56% |
|  |  | Other intentional injuries | 0.00% | 0.00% | 0.00% |  | 5.09% | 0.07% | 10.12% |  | 6.18% | 0.23% | 12.12% |  | 0.45% | 0.09% | 0.82% |
| Men | |  |  |  |  |  |  |  |  |  |  |  |  |  |  |  |  |
| Injuries | | |  |  |  |  |  |  |  |  |  |  |  |  |  |  |  |
|  | Unintentional injuries | |  |  |  |  |  |  |  |  |  |  |  |  |  |  |  |
|  |  | Transport injuries | 9.02% | 5.72% | 12.32% |  | 25.33% | 15.33% | 35.34% |  | 31.98% | 11.18% | 52.78% |  | 13.17% | 4.19% | 22.15% |
|  |  | Poisonings | 0.00% | 0.00% | 0.00% |  | 16.99% | 7.81% | 26.18% |  | 29.66% | 14.84% | 44.49% |  | 18.48% | 8.03% | 28.92% |
|  |  | Falls | 0.00% | 0.00% | 0.00% |  | 16.99% | 7.81% | 26.18% |  | 29.66% | 14.84% | 44.49% |  | 18.48% | 8.03% | 28.92% |
|  |  | Fires, heat and hot substances | 0.00% | 0.00% | 0.00% |  | 16.99% | 7.81% | 26.18% |  | 29.66% | 14.84% | 44.49% |  | 18.48% | 8.03% | 28.92% |
|  |  | Drownings | 0.00% | 0.00% | 0.00% |  | 16.99% | 7.81% | 26.18% |  | 29.66% | 14.84% | 44.49% |  | 18.48% | 8.03% | 28.92% |
|  |  | Other unintentional injuries | 0.00% | 0.00% | 0.00% |  | 16.99% | 7.81% | 26.18% |  | 29.66% | 14.84% | 44.49% |  | 18.48% | 8.03% | 28.92% |
|  | Intentional injuries | |  |  |  |  |  |  |  |  |  |  |  |  |  |  |  |
|  |  | Self-inflicted injuries | 0.00% | 0.00% | 0.00% |  | 16.99% | 7.81% | 26.18% |  | 29.66% | 14.84% | 44.49% |  | 18.48% | 8.03% | 28.92% |
|  |  | Violence | 9.11% | 5.43% | 12.79% |  | 26.11% | 15.56% | 36.66% |  | 15.13% | 9.02% | 21.25% |  | 6.81% | 4.06% | 9.56% |
|  |  | Other intentional injuries | 0.00% | 0.00% | 0.00% |  | 16.99% | 7.81% | 26.18% |  | 29.66% | 14.84% | 44.49% |  | 18.48% | 8.03% | 28.92% |

Table 9: Morbidity Alcohol-Attributable Fractions for injuries (with harms to others included): Europe Eastern

|  |  |  | 0 to 14 years of age | | |  | 15 to 34 years of age | | |  | 35 to 64 years of age | | |  | 65 years of age and older | | |
| --- | --- | --- | --- | --- | --- | --- | --- | --- | --- | --- | --- | --- | --- | --- | --- | --- | --- |
|  |  |  | Point estimate | Lower 95% CI | Upper 95% CI |  | Point estimate | Lower 95% CI | Upper 95% CI |  | Point estimate | Lower 95% CI | Upper 95% CI |  | Point estimate | Lower 95% CI | Upper 95% CI |
| Women | | |  |  |  |  |  |  |  |  |  |  |  |  |  |  |  |
| Injuries | | |  |  |  |  |  |  |  |  |  |  |  |  |  |  |  |
|  | Unintentional injuries | |  |  |  |  |  |  |  |  |  |  |  |  |  |  |  |
|  |  | Transport injuries | 24.28% | 23.38% | 25.18% |  | 42.86% | 42.13% | 43.58% |  | 34.67% | 34.20% | 35.13% |  | 22.03% | 17.69% | 23.11% |
|  |  | Poisonings | 0.00% | 0.00% | 0.00% |  | 13.08% | 1.48% | 24.69% |  | 10.53% | 0.76% | 20.30% |  | 1.27% | 0.00% | 2.73% |
|  |  | Falls | 0.00% | 0.00% | 0.00% |  | 13.08% | 1.48% | 24.69% |  | 10.53% | 0.76% | 20.30% |  | 1.27% | 0.00% | 2.73% |
|  |  | Fires, heat and hot substances | 0.00% | 0.00% | 0.00% |  | 13.08% | 1.48% | 24.69% |  | 10.53% | 0.76% | 20.30% |  | 1.27% | 0.00% | 2.73% |
|  |  | Drownings | 0.00% | 0.00% | 0.00% |  | 13.08% | 1.48% | 24.69% |  | 10.53% | 0.76% | 20.30% |  | 1.27% | 0.00% | 2.73% |
|  |  | Other unintentional injuries | 0.00% | 0.00% | 0.00% |  | 13.08% | 1.48% | 24.69% |  | 10.53% | 0.76% | 20.30% |  | 1.27% | 0.00% | 2.73% |
|  | Intentional injuries | |  |  |  |  |  |  |  |  |  |  |  |  |  |  |  |
|  |  | Self-inflicted injuries | 0.00% | 0.00% | 0.00% |  | 13.08% | 1.48% | 24.69% |  | 10.53% | 0.76% | 20.30% |  | 1.27% | 0.00% | 2.73% |
|  |  | Violence | 13.64% | 8.94% | 18.35% |  | 39.10% | 25.61% | 52.59% |  | 22.66% | 14.84% | 30.48% |  | 10.20% | 6.68% | 13.72% |
|  |  | Other intentional injuries | 0.00% | 0.00% | 0.00% |  | 13.08% | 1.48% | 24.69% |  | 10.53% | 0.76% | 20.30% |  | 1.27% | 0.00% | 2.73% |
| Men | |  |  |  |  |  |  |  |  |  |  |  |  |  |  |  |  |
| Injuries | | |  |  |  |  |  |  |  |  |  |  |  |  |  |  |  |
|  | Unintentional injuries | |  |  |  |  |  |  |  |  |  |  |  |  |  |  |  |
|  |  | Transport injuries | 24.28% | 13.81% | 34.75% |  | 60.00% | 60.00% | 60.00% |  | 60.00% | 60.00% | 60.00% |  | 58.91% | 44.43% | 60.00% |
|  |  | Poisonings | 0.00% | 0.00% | 0.00% |  | 37.05% | 20.67% | 53.43% |  | 33.29% | 18.39% | 48.18% |  | 19.13% | 8.87% | 29.39% |
|  |  | Falls | 0.00% | 0.00% | 0.00% |  | 37.05% | 20.67% | 53.43% |  | 33.29% | 18.39% | 48.18% |  | 19.13% | 8.87% | 29.39% |
|  |  | Fires, heat and hot substances | 0.00% | 0.00% | 0.00% |  | 37.05% | 20.67% | 53.43% |  | 33.29% | 18.39% | 48.18% |  | 19.13% | 8.87% | 29.39% |
|  |  | Drownings | 0.00% | 0.00% | 0.00% |  | 37.05% | 20.67% | 53.43% |  | 33.29% | 18.39% | 48.18% |  | 19.13% | 8.87% | 29.39% |
|  |  | Other unintentional injuries | 0.00% | 0.00% | 0.00% |  | 37.05% | 20.67% | 53.43% |  | 33.29% | 18.39% | 48.18% |  | 19.13% | 8.87% | 29.39% |
|  | Intentional injuries | |  |  |  |  |  |  |  |  |  |  |  |  |  |  |  |
|  |  | Self-inflicted injuries | 0.00% | 0.00% | 0.00% |  | 37.05% | 20.67% | 53.43% |  | 33.29% | 18.39% | 48.18% |  | 19.13% | 8.87% | 29.39% |
|  |  | Violence | 13.64% | 8.94% | 18.35% |  | 39.10% | 25.61% | 52.59% |  | 22.66% | 14.84% | 30.48% |  | 10.20% | 6.68% | 13.72% |
|  |  | Other intentional injuries | 0.00% | 0.00% | 0.00% |  | 37.05% | 20.67% | 53.43% |  | 33.29% | 18.39% | 48.18% |  | 19.13% | 8.87% | 29.39% |

Table 10: Morbidity Alcohol-Attributable Fractions for injuries (with harms to others included): Europe Western

|  |  |  | 0 to 14 years of age | | |  | 15 to 34 years of age | | |  | 35 to 64 years of age | | |  | 65 years of age and older | | |
| --- | --- | --- | --- | --- | --- | --- | --- | --- | --- | --- | --- | --- | --- | --- | --- | --- | --- |
|  |  |  | Point estimate | Lower 95% CI | Upper 95% CI |  | Point estimate | Lower 95% CI | Upper 95% CI |  | Point estimate | Lower 95% CI | Upper 95% CI |  | Point estimate | Lower 95% CI | Upper 95% CI |
| Women | | |  |  |  |  |  |  |  |  |  |  |  |  |  |  |  |
| Injuries | | |  |  |  |  |  |  |  |  |  |  |  |  |  |  |  |
|  | Unintentional injuries | |  |  |  |  |  |  |  |  |  |  |  |  |  |  |  |
|  |  | Transport injuries | 3.71% | 3.66% | 3.76% |  | 7.23% | 5.90% | 8.56% |  | 6.43% | 4.15% | 8.70% |  | 1.88% | 1.13% | 2.64% |
|  |  | Poisonings | 0.00% | 0.00% | 0.00% |  | 2.81% | 1.02% | 4.60% |  | 4.92% | 0.57% | 9.26% |  | 1.76% | 0.64% | 2.88% |
|  |  | Falls | 0.00% | 0.00% | 0.00% |  | 2.81% | 1.02% | 4.60% |  | 4.92% | 0.57% | 9.26% |  | 1.76% | 0.64% | 2.88% |
|  |  | Fires, heat and hot substances | 0.00% | 0.00% | 0.00% |  | 2.81% | 1.02% | 4.60% |  | 4.92% | 0.57% | 9.26% |  | 1.76% | 0.64% | 2.88% |
|  |  | Drownings | 0.00% | 0.00% | 0.00% |  | 2.81% | 1.02% | 4.60% |  | 4.92% | 0.57% | 9.26% |  | 1.76% | 0.64% | 2.88% |
|  |  | Other unintentional injuries | 0.00% | 0.00% | 0.00% |  | 2.81% | 1.02% | 4.60% |  | 4.92% | 0.57% | 9.26% |  | 1.76% | 0.64% | 2.88% |
|  | Intentional injuries | |  |  |  |  |  |  |  |  |  |  |  |  |  |  |  |
|  |  | Self-inflicted injuries | 0.00% | 0.00% | 0.00% |  | 2.81% | 1.02% | 4.60% |  | 4.92% | 0.57% | 9.26% |  | 1.76% | 0.64% | 2.88% |
|  |  | Violence | 5.62% | 3.61% | 7.63% |  | 16.11% | 10.35% | 21.87% |  | 9.33% | 6.00% | 12.67% |  | 4.20% | 2.70% | 5.70% |
|  |  | Other intentional injuries | 0.00% | 0.00% | 0.00% |  | 2.81% | 1.02% | 4.60% |  | 4.92% | 0.57% | 9.26% |  | 1.76% | 0.64% | 2.88% |
| Men | |  |  |  |  |  |  |  |  |  |  |  |  |  |  |  |  |
| Injuries | | |  |  |  |  |  |  |  |  |  |  |  |  |  |  |  |
|  | Unintentional injuries | |  |  |  |  |  |  |  |  |  |  |  |  |  |  |  |
|  |  | Transport injuries | 3.71% | 3.09% | 4.33% |  | 11.18% | 7.29% | 15.07% |  | 11.81% | 5.87% | 17.75% |  | 3.74% | 1.82% | 5.66% |
|  |  | Poisonings | 0.00% | 0.00% | 0.00% |  | 12.35% | 5.54% | 19.16% |  | 17.51% | 8.21% | 26.81% |  | 9.12% | 3.82% | 14.42% |
|  |  | Falls | 0.00% | 0.00% | 0.00% |  | 12.35% | 5.54% | 19.16% |  | 17.51% | 8.21% | 26.81% |  | 9.12% | 3.82% | 14.42% |
|  |  | Fires, heat and hot substances | 0.00% | 0.00% | 0.00% |  | 12.35% | 5.54% | 19.16% |  | 17.51% | 8.21% | 26.81% |  | 9.12% | 3.82% | 14.42% |
|  |  | Drownings | 0.00% | 0.00% | 0.00% |  | 12.35% | 5.54% | 19.16% |  | 17.51% | 8.21% | 26.81% |  | 9.12% | 3.82% | 14.42% |
|  |  | Other unintentional injuries | 0.00% | 0.00% | 0.00% |  | 12.35% | 5.54% | 19.16% |  | 17.51% | 8.21% | 26.81% |  | 9.12% | 3.82% | 14.42% |
|  | Intentional injuries | |  |  |  |  |  |  |  |  |  |  |  |  |  |  |  |
|  |  | Self-inflicted injuries | 0.00% | 0.00% | 0.00% |  | 12.35% | 5.54% | 19.16% |  | 17.51% | 8.21% | 26.81% |  | 9.12% | 3.82% | 14.42% |
|  |  | Violence | 5.62% | 3.61% | 7.63% |  | 16.11% | 10.35% | 21.87% |  | 9.33% | 6.00% | 12.67% |  | 4.20% | 2.70% | 5.70% |
|  |  | Other intentional injuries | 0.00% | 0.00% | 0.00% |  | 12.35% | 5.54% | 19.16% |  | 17.51% | 8.21% | 26.81% |  | 9.12% | 3.82% | 14.42% |

Table 11: Morbidity Alcohol-Attributable Fractions for injuries (with harms to others included): Latin America Andean

|  |  |  | 0 to 14 years of age | | |  | 15 to 34 years of age | | |  | 35 to 64 years of age | | |  | 65 years of age and older | | |
| --- | --- | --- | --- | --- | --- | --- | --- | --- | --- | --- | --- | --- | --- | --- | --- | --- | --- |
|  |  |  | Point estimate | Lower 95% CI | Upper 95% CI |  | Point estimate | Lower 95% CI | Upper 95% CI |  | Point estimate | Lower 95% CI | Upper 95% CI |  | Point estimate | Lower 95% CI | Upper 95% CI |
| Women | | |  |  |  |  |  |  |  |  |  |  |  |  |  |  |  |
| Injuries | | |  |  |  |  |  |  |  |  |  |  |  |  |  |  |  |
|  | Unintentional injuries | |  |  |  |  |  |  |  |  |  |  |  |  |  |  |  |
|  |  | Transport injuries | 3.77% | 3.72% | 3.81% |  | 6.80% | 5.85% | 7.75% |  | 5.78% | 4.19% | 7.37% |  | 1.01% | 0.85% | 1.18% |
|  |  | Poisonings | 0.00% | 0.00% | 0.00% |  | 2.17% | 0.00% | 5.65% |  | 1.52% | 0.00% | 3.31% |  | 0.76% | 0.00% | 1.77% |
|  |  | Falls | 0.00% | 0.00% | 0.00% |  | 2.17% | 0.00% | 5.65% |  | 1.52% | 0.00% | 3.31% |  | 0.76% | 0.00% | 1.77% |
|  |  | Fires, heat and hot substances | 0.00% | 0.00% | 0.00% |  | 2.17% | 0.00% | 5.65% |  | 1.52% | 0.00% | 3.31% |  | 0.76% | 0.00% | 1.77% |
|  |  | Drownings | 0.00% | 0.00% | 0.00% |  | 2.17% | 0.00% | 5.65% |  | 1.52% | 0.00% | 3.31% |  | 0.76% | 0.00% | 1.77% |
|  |  | Other unintentional injuries | 0.00% | 0.00% | 0.00% |  | 2.17% | 0.00% | 5.65% |  | 1.52% | 0.00% | 3.31% |  | 0.76% | 0.00% | 1.77% |
|  | Intentional injuries | |  |  |  |  |  |  |  |  |  |  |  |  |  |  |  |
|  |  | Self-inflicted injuries | 0.00% | 0.00% | 0.00% |  | 2.17% | 0.00% | 5.65% |  | 1.52% | 0.00% | 3.31% |  | 0.76% | 0.00% | 1.77% |
|  |  | Violence | 3.41% | 1.71% | 5.11% |  | 9.77% | 4.90% | 14.64% |  | 5.66% | 2.84% | 8.48% |  | 2.55% | 1.28% | 3.82% |
|  |  | Other intentional injuries | 0.00% | 0.00% | 0.00% |  | 2.17% | 0.00% | 5.65% |  | 1.52% | 0.00% | 3.31% |  | 0.76% | 0.00% | 1.77% |
| Men | |  |  |  |  |  |  |  |  |  |  |  |  |  |  |  |  |
| Injuries | | |  |  |  |  |  |  |  |  |  |  |  |  |  |  |  |
|  | Unintentional injuries | |  |  |  |  |  |  |  |  |  |  |  |  |  |  |  |
|  |  | Transport injuries | 3.77% | 3.21% | 4.32% |  | 11.72% | 8.12% | 15.32% |  | 12.46% | 7.01% | 17.91% |  | 1.84% | 1.26% | 2.41% |
|  |  | Poisonings | 0.00% | 0.00% | 0.00% |  | 7.46% | 2.14% | 12.78% |  | 12.65% | 3.64% | 21.66% |  | 1.29% | 0.33% | 2.25% |
|  |  | Falls | 0.00% | 0.00% | 0.00% |  | 7.46% | 2.14% | 12.78% |  | 12.65% | 3.64% | 21.66% |  | 1.29% | 0.33% | 2.25% |
|  |  | Fires, heat and hot substances | 0.00% | 0.00% | 0.00% |  | 7.46% | 2.14% | 12.78% |  | 12.65% | 3.64% | 21.66% |  | 1.29% | 0.33% | 2.25% |
|  |  | Drownings | 0.00% | 0.00% | 0.00% |  | 7.46% | 2.14% | 12.78% |  | 12.65% | 3.64% | 21.66% |  | 1.29% | 0.33% | 2.25% |
|  |  | Other unintentional injuries | 0.00% | 0.00% | 0.00% |  | 7.46% | 2.14% | 12.78% |  | 12.65% | 3.64% | 21.66% |  | 1.29% | 0.33% | 2.25% |
|  | Intentional injuries | |  |  |  |  |  |  |  |  |  |  |  |  |  |  |  |
|  |  | Self-inflicted injuries | 0.00% | 0.00% | 0.00% |  | 7.46% | 2.14% | 12.78% |  | 12.65% | 3.64% | 21.66% |  | 1.29% | 0.33% | 2.25% |
|  |  | Violence | 3.41% | 1.71% | 5.11% |  | 9.77% | 4.90% | 14.64% |  | 5.66% | 2.84% | 8.48% |  | 2.55% | 1.28% | 3.82% |
|  |  | Other intentional injuries | 0.00% | 0.00% | 0.00% |  | 7.46% | 2.14% | 12.78% |  | 12.65% | 3.64% | 21.66% |  | 1.29% | 0.33% | 2.25% |

Table 12: Morbidity Alcohol-Attributable Fractions for injuries (with harms to others included): Latin America Central

|  |  |  | 0 to 14 years of age | | |  | 15 to 34 years of age | | |  | 35 to 64 years of age | | |  | 65 years of age and older | | |
| --- | --- | --- | --- | --- | --- | --- | --- | --- | --- | --- | --- | --- | --- | --- | --- | --- | --- |
|  |  |  | Point estimate | Lower 95% CI | Upper 95% CI |  | Point estimate | Lower 95% CI | Upper 95% CI |  | Point estimate | Lower 95% CI | Upper 95% CI |  | Point estimate | Lower 95% CI | Upper 95% CI |
| Women | | |  |  |  |  |  |  |  |  |  |  |  |  |  |  |  |
| Injuries | | |  |  |  |  |  |  |  |  |  |  |  |  |  |  |  |
|  | Unintentional injuries | |  |  |  |  |  |  |  |  |  |  |  |  |  |  |  |
|  |  | Transport injuries | 10.73% | 10.43% | 11.03% |  | 18.38% | 15.85% | 20.91% |  | 15.01% | 12.35% | 17.67% |  | 5.72% | 4.10% | 7.33% |
|  |  | Poisonings | 0.00% | 0.00% | 0.00% |  | 2.25% | 0.00% | 5.35% |  | 1.45% | 0.00% | 3.37% |  | 0.40% | 0.02% | 0.78% |
|  |  | Falls | 0.00% | 0.00% | 0.00% |  | 2.25% | 0.00% | 5.35% |  | 1.45% | 0.00% | 3.37% |  | 0.40% | 0.02% | 0.78% |
|  |  | Fires, heat and hot substances | 0.00% | 0.00% | 0.00% |  | 2.25% | 0.00% | 5.35% |  | 1.45% | 0.00% | 3.37% |  | 0.40% | 0.02% | 0.78% |
|  |  | Drownings | 0.00% | 0.00% | 0.00% |  | 2.25% | 0.00% | 5.35% |  | 1.45% | 0.00% | 3.37% |  | 0.40% | 0.02% | 0.78% |
|  |  | Other unintentional injuries | 0.00% | 0.00% | 0.00% |  | 2.25% | 0.00% | 5.35% |  | 1.45% | 0.00% | 3.37% |  | 0.40% | 0.02% | 0.78% |
|  | Intentional injuries | |  |  |  |  |  |  |  |  |  |  |  |  |  |  |  |
|  |  | Self-inflicted injuries | 0.00% | 0.00% | 0.00% |  | 2.25% | 0.00% | 5.35% |  | 1.45% | 0.00% | 3.37% |  | 0.40% | 0.02% | 0.78% |
|  |  | Violence | 4.25% | 2.55% | 5.95% |  | 12.18% | 7.30% | 17.05% |  | 7.06% | 4.23% | 9.88% |  | 3.18% | 1.90% | 4.45% |
|  |  | Other intentional injuries | 0.00% | 0.00% | 0.00% |  | 2.25% | 0.00% | 5.35% |  | 1.45% | 0.00% | 3.37% |  | 0.40% | 0.02% | 0.78% |
| Men | |  |  |  |  |  |  |  |  |  |  |  |  |  |  |  |  |
| Injuries | | |  |  |  |  |  |  |  |  |  |  |  |  |  |  |  |
|  | Unintentional injuries | |  |  |  |  |  |  |  |  |  |  |  |  |  |  |  |
|  |  | Transport injuries | 10.73% | 7.23% | 14.23% |  | 32.08% | 19.88% | 44.28% |  | 32.67% | 21.51% | 43.83% |  | 15.70% | 9.69% | 21.71% |
|  |  | Poisonings | 0.00% | 0.00% | 0.00% |  | 10.54% | 4.29% | 16.80% |  | 14.11% | 6.02% | 22.20% |  | 8.54% | 3.07% | 14.02% |
|  |  | Falls | 0.00% | 0.00% | 0.00% |  | 10.54% | 4.29% | 16.80% |  | 14.11% | 6.02% | 22.20% |  | 8.54% | 3.07% | 14.02% |
|  |  | Fires, heat and hot substances | 0.00% | 0.00% | 0.00% |  | 10.54% | 4.29% | 16.80% |  | 14.11% | 6.02% | 22.20% |  | 8.54% | 3.07% | 14.02% |
|  |  | Drownings | 0.00% | 0.00% | 0.00% |  | 10.54% | 4.29% | 16.80% |  | 14.11% | 6.02% | 22.20% |  | 8.54% | 3.07% | 14.02% |
|  |  | Other unintentional injuries | 0.00% | 0.00% | 0.00% |  | 10.54% | 4.29% | 16.80% |  | 14.11% | 6.02% | 22.20% |  | 8.54% | 3.07% | 14.02% |
|  | Intentional injuries | |  |  |  |  |  |  |  |  |  |  |  |  |  |  |  |
|  |  | Self-inflicted injuries | 0.00% | 0.00% | 0.00% |  | 10.54% | 4.29% | 16.80% |  | 14.11% | 6.02% | 22.20% |  | 8.54% | 3.07% | 14.02% |
|  |  | Violence | 4.25% | 2.55% | 5.95% |  | 12.18% | 7.30% | 17.05% |  | 7.06% | 4.23% | 9.88% |  | 3.18% | 1.90% | 4.45% |
|  |  | Other intentional injuries | 0.00% | 0.00% | 0.00% |  | 10.54% | 4.29% | 16.80% |  | 14.11% | 6.02% | 22.20% |  | 8.54% | 3.07% | 14.02% |

Table 13: Morbidity Alcohol-Attributable Fractions for injuries (with harms to others included): Latin America Southern

|  |  |  | 0 to 14 years of age | | |  | 15 to 34 years of age | | |  | 35 to 64 years of age | | |  | 65 years of age and older | | |
| --- | --- | --- | --- | --- | --- | --- | --- | --- | --- | --- | --- | --- | --- | --- | --- | --- | --- |
|  |  |  | Point estimate | Lower 95% CI | Upper 95% CI |  | Point estimate | Lower 95% CI | Upper 95% CI |  | Point estimate | Lower 95% CI | Upper 95% CI |  | Point estimate | Lower 95% CI | Upper 95% CI |
| Women | | |  |  |  |  |  |  |  |  |  |  |  |  |  |  |  |
| Injuries | | |  |  |  |  |  |  |  |  |  |  |  |  |  |  |  |
|  | Unintentional injuries | |  |  |  |  |  |  |  |  |  |  |  |  |  |  |  |
|  |  | Transport injuries | 3.26% | 3.23% | 3.30% |  | 6.66% | 5.53% | 7.80% |  | 4.45% | 3.44% | 5.46% |  | 1.56% | 1.04% | 2.08% |
|  |  | Poisonings | 0.00% | 0.00% | 0.00% |  | 1.90% | 0.50% | 3.31% |  | 3.44% | 0.00% | 7.30% |  | 1.28% | 0.32% | 2.23% |
|  |  | Falls | 0.00% | 0.00% | 0.00% |  | 1.90% | 0.50% | 3.31% |  | 3.44% | 0.00% | 7.30% |  | 1.28% | 0.32% | 2.23% |
|  |  | Fires, heat and hot substances | 0.00% | 0.00% | 0.00% |  | 1.90% | 0.50% | 3.31% |  | 3.44% | 0.00% | 7.30% |  | 1.28% | 0.32% | 2.23% |
|  |  | Drownings | 0.00% | 0.00% | 0.00% |  | 1.90% | 0.50% | 3.31% |  | 3.44% | 0.00% | 7.30% |  | 1.28% | 0.32% | 2.23% |
|  |  | Other unintentional injuries | 0.00% | 0.00% | 0.00% |  | 1.90% | 0.50% | 3.31% |  | 3.44% | 0.00% | 7.30% |  | 1.28% | 0.32% | 2.23% |
|  | Intentional injuries | |  |  |  |  |  |  |  |  |  |  |  |  |  |  |  |
|  |  | Self-inflicted injuries | 0.00% | 0.00% | 0.00% |  | 1.90% | 0.50% | 3.31% |  | 3.44% | 0.00% | 7.30% |  | 1.28% | 0.32% | 2.23% |
|  |  | Violence | 3.79% | 2.40% | 5.19% |  | 10.87% | 6.87% | 14.88% |  | 6.30% | 3.98% | 8.62% |  | 2.84% | 1.79% | 3.88% |
|  |  | Other intentional injuries | 0.00% | 0.00% | 0.00% |  | 1.90% | 0.50% | 3.31% |  | 3.44% | 0.00% | 7.30% |  | 1.28% | 0.32% | 2.23% |
| Men | |  |  |  |  |  |  |  |  |  |  |  |  |  |  |  |  |
| Injuries | | |  |  |  |  |  |  |  |  |  |  |  |  |  |  |  |
|  | Unintentional injuries | |  |  |  |  |  |  |  |  |  |  |  |  |  |  |  |
|  |  | Transport injuries | 3.26% | 2.85% | 3.67% |  | 10.95% | 7.59% | 14.32% |  | 7.64% | 4.81% | 10.48% |  | 3.19% | 1.80% | 4.58% |
|  |  | Poisonings | 0.00% | 0.00% | 0.00% |  | 10.21% | 4.17% | 16.26% |  | 10.37% | 4.11% | 16.63% |  | 6.53% | 2.34% | 10.71% |
|  |  | Falls | 0.00% | 0.00% | 0.00% |  | 10.21% | 4.17% | 16.26% |  | 10.37% | 4.11% | 16.63% |  | 6.53% | 2.34% | 10.71% |
|  |  | Fires, heat and hot substances | 0.00% | 0.00% | 0.00% |  | 10.21% | 4.17% | 16.26% |  | 10.37% | 4.11% | 16.63% |  | 6.53% | 2.34% | 10.71% |
|  |  | Drownings | 0.00% | 0.00% | 0.00% |  | 10.21% | 4.17% | 16.26% |  | 10.37% | 4.11% | 16.63% |  | 6.53% | 2.34% | 10.71% |
|  |  | Other unintentional injuries | 0.00% | 0.00% | 0.00% |  | 10.21% | 4.17% | 16.26% |  | 10.37% | 4.11% | 16.63% |  | 6.53% | 2.34% | 10.71% |
|  | Intentional injuries | |  |  |  |  |  |  |  |  |  |  |  |  |  |  |  |
|  |  | Self-inflicted injuries | 0.00% | 0.00% | 0.00% |  | 10.21% | 4.17% | 16.26% |  | 10.37% | 4.11% | 16.63% |  | 6.53% | 2.34% | 10.71% |
|  |  | Violence | 3.79% | 2.40% | 5.19% |  | 10.87% | 6.87% | 14.88% |  | 6.30% | 3.98% | 8.62% |  | 2.84% | 1.79% | 3.88% |
|  |  | Other intentional injuries | 0.00% | 0.00% | 0.00% |  | 10.21% | 4.17% | 16.26% |  | 10.37% | 4.11% | 16.63% |  | 6.53% | 2.34% | 10.71% |

Table 14: Morbidity Alcohol-Attributable Fractions for injuries (with harms to others included): Latin America Tropical

|  |  |  | 0 to 14 years of age | | |  | 15 to 34 years of age | | |  | 35 to 64 years of age | | |  | 65 years of age and older | | |
| --- | --- | --- | --- | --- | --- | --- | --- | --- | --- | --- | --- | --- | --- | --- | --- | --- | --- |
|  |  |  | Point estimate | Lower 95% CI | Upper 95% CI |  | Point estimate | Lower 95% CI | Upper 95% CI |  | Point estimate | Lower 95% CI | Upper 95% CI |  | Point estimate | Lower 95% CI | Upper 95% CI |
| Women | | |  |  |  |  |  |  |  |  |  |  |  |  |  |  |  |
| Injuries | | |  |  |  |  |  |  |  |  |  |  |  |  |  |  |  |
|  | Unintentional injuries | |  |  |  |  |  |  |  |  |  |  |  |  |  |  |  |
|  |  | Transport injuries | 5.97% | 5.83% | 6.11% |  | 11.18% | 8.95% | 13.41% |  | 7.70% | 5.05% | 10.36% |  | 1.40% | 1.20% | 1.60% |
|  |  | Poisonings | 0.00% | 0.00% | 0.00% |  | 2.83% | 0.00% | 6.06% |  | 4.20% | 0.00% | 9.13% |  | 0.37% | 0.01% | 0.73% |
|  |  | Falls | 0.00% | 0.00% | 0.00% |  | 2.83% | 0.00% | 6.06% |  | 4.20% | 0.00% | 9.13% |  | 0.37% | 0.01% | 0.73% |
|  |  | Fires, heat and hot substances | 0.00% | 0.00% | 0.00% |  | 2.83% | 0.00% | 6.06% |  | 4.20% | 0.00% | 9.13% |  | 0.37% | 0.01% | 0.73% |
|  |  | Drownings | 0.00% | 0.00% | 0.00% |  | 2.83% | 0.00% | 6.06% |  | 4.20% | 0.00% | 9.13% |  | 0.37% | 0.01% | 0.73% |
|  |  | Other unintentional injuries | 0.00% | 0.00% | 0.00% |  | 2.83% | 0.00% | 6.06% |  | 4.20% | 0.00% | 9.13% |  | 0.37% | 0.01% | 0.73% |
|  | Intentional injuries | |  |  |  |  |  |  |  |  |  |  |  |  |  |  |  |
|  |  | Self-inflicted injuries | 0.00% | 0.00% | 0.00% |  | 2.83% | 0.00% | 6.06% |  | 4.20% | 0.00% | 9.13% |  | 0.37% | 0.01% | 0.73% |
|  |  | Violence | 5.52% | 3.13% | 7.91% |  | 15.82% | 8.98% | 22.66% |  | 9.17% | 5.20% | 13.13% |  | 4.13% | 2.34% | 5.91% |
|  |  | Other intentional injuries | 0.00% | 0.00% | 0.00% |  | 2.83% | 0.00% | 6.06% |  | 4.20% | 0.00% | 9.13% |  | 0.37% | 0.01% | 0.73% |
| Men | |  |  |  |  |  |  |  |  |  |  |  |  |  |  |  |  |
| Injuries | | |  |  |  |  |  |  |  |  |  |  |  |  |  |  |  |
|  | Unintentional injuries | |  |  |  |  |  |  |  |  |  |  |  |  |  |  |  |
|  |  | Transport injuries | 5.97% | 4.32% | 7.61% |  | 19.63% | 11.18% | 28.08% |  | 14.61% | 5.50% | 23.73% |  | 2.18% | 1.46% | 2.90% |
|  |  | Poisonings | 0.00% | 0.00% | 0.00% |  | 15.75% | 6.20% | 25.31% |  | 14.78% | 5.25% | 24.31% |  | 2.47% | 0.64% | 4.29% |
|  |  | Falls | 0.00% | 0.00% | 0.00% |  | 15.75% | 6.20% | 25.31% |  | 14.78% | 5.25% | 24.31% |  | 2.47% | 0.64% | 4.29% |
|  |  | Fires, heat and hot substances | 0.00% | 0.00% | 0.00% |  | 15.75% | 6.20% | 25.31% |  | 14.78% | 5.25% | 24.31% |  | 2.47% | 0.64% | 4.29% |
|  |  | Drownings | 0.00% | 0.00% | 0.00% |  | 15.75% | 6.20% | 25.31% |  | 14.78% | 5.25% | 24.31% |  | 2.47% | 0.64% | 4.29% |
|  |  | Other unintentional injuries | 0.00% | 0.00% | 0.00% |  | 15.75% | 6.20% | 25.31% |  | 14.78% | 5.25% | 24.31% |  | 2.47% | 0.64% | 4.29% |
|  | Intentional injuries | |  |  |  |  |  |  |  |  |  |  |  |  |  |  |  |
|  |  | Self-inflicted injuries | 0.00% | 0.00% | 0.00% |  | 15.75% | 6.20% | 25.31% |  | 14.78% | 5.25% | 24.31% |  | 2.47% | 0.64% | 4.29% |
|  |  | Violence | 5.52% | 3.13% | 7.91% |  | 15.82% | 8.98% | 22.66% |  | 9.17% | 5.20% | 13.13% |  | 4.13% | 2.34% | 5.91% |
|  |  | Other intentional injuries | 0.00% | 0.00% | 0.00% |  | 15.75% | 6.20% | 25.31% |  | 14.78% | 5.25% | 24.31% |  | 2.47% | 0.64% | 4.29% |

Table 15: Morbidity Alcohol-Attributable Fractions for injuries (with harms to others included): North Africa Middle East

|  |  |  | 0 to 14 years of age | | |  | 15 to 34 years of age | | |  | 35 to 64 years of age | | |  | 65 years of age and older | | |
| --- | --- | --- | --- | --- | --- | --- | --- | --- | --- | --- | --- | --- | --- | --- | --- | --- | --- |
|  |  |  | Point estimate | Lower 95% CI | Upper 95% CI |  | Point estimate | Lower 95% CI | Upper 95% CI |  | Point estimate | Lower 95% CI | Upper 95% CI |  | Point estimate | Lower 95% CI | Upper 95% CI |
| Women | | |  |  |  |  |  |  |  |  |  |  |  |  |  |  |  |
| Injuries | | |  |  |  |  |  |  |  |  |  |  |  |  |  |  |  |
|  | Unintentional injuries | |  |  |  |  |  |  |  |  |  |  |  |  |  |  |  |
|  |  | Transport injuries | 1.02% | 0.99% | 1.05% |  | 1.61% | 1.21% | 3.22% |  | 0.85% | 0.76% | 1.01% |  | 0.18% | 0.18% | 0.19% |
|  |  | Poisonings | 0.00% | 0.00% | 0.00% |  | 0.22% | 0.00% | 1.45% |  | 0.13% | 0.00% | 1.89% |  | 0.02% | 0.00% | 0.57% |
|  |  | Falls | 0.00% | 0.00% | 0.00% |  | 0.22% | 0.00% | 1.45% |  | 0.13% | 0.00% | 1.89% |  | 0.02% | 0.00% | 0.57% |
|  |  | Fires, heat and hot substances | 0.00% | 0.00% | 0.00% |  | 0.22% | 0.00% | 1.45% |  | 0.13% | 0.00% | 1.89% |  | 0.02% | 0.00% | 0.57% |
|  |  | Drownings | 0.00% | 0.00% | 0.00% |  | 0.22% | 0.00% | 1.45% |  | 0.13% | 0.00% | 1.89% |  | 0.02% | 0.00% | 0.57% |
|  |  | Other unintentional injuries | 0.00% | 0.00% | 0.00% |  | 0.22% | 0.00% | 1.45% |  | 0.13% | 0.00% | 1.89% |  | 0.02% | 0.00% | 0.57% |
|  | Intentional injuries | |  |  |  |  |  |  |  |  |  |  |  |  |  |  |  |
|  |  | Self-inflicted injuries | 0.00% | 0.00% | 0.00% |  | 0.22% | 0.00% | 1.45% |  | 0.13% | 0.00% | 1.89% |  | 0.02% | 0.00% | 0.57% |
|  |  | Violence | 0.85% | 0.00% | 2.96% |  | 2.44% | 0.00% | 8.48% |  | 1.41% | 0.00% | 4.92% |  | 0.64% | 0.00% | 2.21% |
|  |  | Other intentional injuries | 0.00% | 0.00% | 0.00% |  | 0.22% | 0.00% | 1.45% |  | 0.13% | 0.00% | 1.89% |  | 0.02% | 0.00% | 0.57% |
| Men | |  |  |  |  |  |  |  |  |  |  |  |  |  |  |  |  |
| Injuries | | |  |  |  |  |  |  |  |  |  |  |  |  |  |  |  |
|  | Unintentional injuries | |  |  |  |  |  |  |  |  |  |  |  |  |  |  |  |
|  |  | Transport injuries | 1.02% | 0.64% | 1.39% |  | 3.93% | 0.71% | 16.58% |  | 1.31% | 0.56% | 2.61% |  | 0.23% | 0.17% | 0.29% |
|  |  | Poisonings | 0.00% | 0.00% | 0.00% |  | 3.51% | 0.07% | 6.96% |  | 1.45% | 0.00% | 3.11% |  | 0.04% | 0.01% | 0.07% |
|  |  | Falls | 0.00% | 0.00% | 0.00% |  | 3.51% | 0.07% | 6.96% |  | 1.45% | 0.00% | 3.11% |  | 0.04% | 0.01% | 0.07% |
|  |  | Fires, heat and hot substances | 0.00% | 0.00% | 0.00% |  | 3.51% | 0.07% | 6.96% |  | 1.45% | 0.00% | 3.11% |  | 0.04% | 0.01% | 0.07% |
|  |  | Drownings | 0.00% | 0.00% | 0.00% |  | 3.51% | 0.07% | 6.96% |  | 1.45% | 0.00% | 3.11% |  | 0.04% | 0.01% | 0.07% |
|  |  | Other unintentional injuries | 0.00% | 0.00% | 0.00% |  | 3.51% | 0.07% | 6.96% |  | 1.45% | 0.00% | 3.11% |  | 0.04% | 0.01% | 0.07% |
|  | Intentional injuries | |  |  |  |  |  |  |  |  |  |  |  |  |  |  |  |
|  |  | Self-inflicted injuries | 0.00% | 0.00% | 0.00% |  | 3.51% | 0.07% | 6.96% |  | 1.45% | 0.00% | 3.11% |  | 0.04% | 0.01% | 0.07% |
|  |  | Violence | 0.85% | 0.00% | 2.96% |  | 2.44% | 0.00% | 8.48% |  | 1.41% | 0.00% | 4.92% |  | 0.64% | 0.00% | 2.21% |
|  |  | Other intentional injuries | 0.00% | 0.00% | 0.00% |  | 3.51% | 0.07% | 6.96% |  | 1.45% | 0.00% | 3.11% |  | 0.04% | 0.01% | 0.07% |

Table 16: Morbidity Alcohol-Attributable Fractions for injuries (with harms to others included): North America High Income

|  |  |  | 0 to 14 years of age | | |  | 15 to 34 years of age | | |  | 35 to 64 years of age | | |  | 65 years of age and older | | |
| --- | --- | --- | --- | --- | --- | --- | --- | --- | --- | --- | --- | --- | --- | --- | --- | --- | --- |
|  |  |  | Point estimate | Lower 95% CI | Upper 95% CI |  | Point estimate | Lower 95% CI | Upper 95% CI |  | Point estimate | Lower 95% CI | Upper 95% CI |  | Point estimate | Lower 95% CI | Upper 95% CI |
| Women | | |  |  |  |  |  |  |  |  |  |  |  |  |  |  |  |
| Injuries | | |  |  |  |  |  |  |  |  |  |  |  |  |  |  |  |
|  | Unintentional injuries | |  |  |  |  |  |  |  |  |  |  |  |  |  |  |  |
|  |  | Transport injuries | 3.83% | 3.77% | 3.89% |  | 8.38% | 6.22% | 10.54% |  | 4.50% | 3.59% | 5.41% |  | 1.22% | 0.93% | 1.50% |
|  |  | Poisonings | 0.00% | 0.00% | 0.00% |  | 3.56% | 1.03% | 6.08% |  | 2.48% | 0.85% | 4.12% |  | 0.67% | 0.24% | 1.11% |
|  |  | Falls | 0.00% | 0.00% | 0.00% |  | 3.56% | 1.03% | 6.08% |  | 2.48% | 0.85% | 4.12% |  | 0.67% | 0.24% | 1.11% |
|  |  | Fires, heat and hot substances | 0.00% | 0.00% | 0.00% |  | 3.56% | 1.03% | 6.08% |  | 2.48% | 0.85% | 4.12% |  | 0.67% | 0.24% | 1.11% |
|  |  | Drownings | 0.00% | 0.00% | 0.00% |  | 3.56% | 1.03% | 6.08% |  | 2.48% | 0.85% | 4.12% |  | 0.67% | 0.24% | 1.11% |
|  |  | Other unintentional injuries | 0.00% | 0.00% | 0.00% |  | 3.56% | 1.03% | 6.08% |  | 2.48% | 0.85% | 4.12% |  | 0.67% | 0.24% | 1.11% |
|  | Intentional injuries | |  |  |  |  |  |  |  |  |  |  |  |  |  |  |  |
|  |  | Self-inflicted injuries | 0.00% | 0.00% | 0.00% |  | 3.56% | 1.03% | 6.08% |  | 2.48% | 0.85% | 4.12% |  | 0.67% | 0.24% | 1.11% |
|  |  | Violence | 4.58% | 2.94% | 6.22% |  | 13.13% | 8.43% | 17.83% |  | 7.61% | 4.89% | 10.33% |  | 3.43% | 2.20% | 4.65% |
|  |  | Other intentional injuries | 0.00% | 0.00% | 0.00% |  | 3.56% | 1.03% | 6.08% |  | 2.48% | 0.85% | 4.12% |  | 0.67% | 0.24% | 1.11% |
| Men | |  |  |  |  |  |  |  |  |  |  |  |  |  |  |  |  |
| Injuries | | |  |  |  |  |  |  |  |  |  |  |  |  |  |  |  |
|  | Unintentional injuries | |  |  |  |  |  |  |  |  |  |  |  |  |  |  |  |
|  |  | Transport injuries | 3.83% | 3.16% | 4.50% |  | 15.09% | 8.37% | 21.80% |  | 7.38% | 4.51% | 10.24% |  | 2.23% | 1.39% | 3.07% |
|  |  | Poisonings | 0.00% | 0.00% | 0.00% |  | 17.04% | 7.87% | 26.20% |  | 10.35% | 4.44% | 16.26% |  | 4.58% | 1.71% | 7.44% |
|  |  | Falls | 0.00% | 0.00% | 0.00% |  | 17.04% | 7.87% | 26.20% |  | 10.35% | 4.44% | 16.26% |  | 4.58% | 1.71% | 7.44% |
|  |  | Fires, heat and hot substances | 0.00% | 0.00% | 0.00% |  | 17.04% | 7.87% | 26.20% |  | 10.35% | 4.44% | 16.26% |  | 4.58% | 1.71% | 7.44% |
|  |  | Drownings | 0.00% | 0.00% | 0.00% |  | 17.04% | 7.87% | 26.20% |  | 10.35% | 4.44% | 16.26% |  | 4.58% | 1.71% | 7.44% |
|  |  | Other unintentional injuries | 0.00% | 0.00% | 0.00% |  | 17.04% | 7.87% | 26.20% |  | 10.35% | 4.44% | 16.26% |  | 4.58% | 1.71% | 7.44% |
|  | Intentional injuries | |  |  |  |  |  |  |  |  |  |  |  |  |  |  |  |
|  |  | Self-inflicted injuries | 0.00% | 0.00% | 0.00% |  | 17.04% | 7.87% | 26.20% |  | 10.35% | 4.44% | 16.26% |  | 4.58% | 1.71% | 7.44% |
|  |  | Violence | 4.58% | 2.94% | 6.22% |  | 13.13% | 8.43% | 17.83% |  | 7.61% | 4.89% | 10.33% |  | 3.43% | 2.20% | 4.65% |
|  |  | Other intentional injuries | 0.00% | 0.00% | 0.00% |  | 17.04% | 7.87% | 26.20% |  | 10.35% | 4.44% | 16.26% |  | 4.58% | 1.71% | 7.44% |

Table 17: Morbidity Alcohol-Attributable Fractions for injuries (with harms to others included): Oceania

|  |  |  | 0 to 14 years of age | | |  | 15 to 34 years of age | | |  | 35 to 64 years of age | | |  | 65 years of age and older | | |
| --- | --- | --- | --- | --- | --- | --- | --- | --- | --- | --- | --- | --- | --- | --- | --- | --- | --- |
|  |  |  | Point estimate | Lower 95% CI | Upper 95% CI |  | Point estimate | Lower 95% CI | Upper 95% CI |  | Point estimate | Lower 95% CI | Upper 95% CI |  | Point estimate | Lower 95% CI | Upper 95% CI |
| Women | | |  |  |  |  |  |  |  |  |  |  |  |  |  |  |  |
| Injuries | | |  |  |  |  |  |  |  |  |  |  |  |  |  |  |  |
|  | Unintentional injuries | |  |  |  |  |  |  |  |  |  |  |  |  |  |  |  |
|  |  | Transport injuries | 3.80% | 3.76% | 3.85% |  | 6.43% | 5.71% | 7.15% |  | 4.28% | 3.72% | 4.84% |  | 1.31% | 1.04% | 1.59% |
|  |  | Poisonings | 0.00% | 0.00% | 0.00% |  | 1.48% | 0.55% | 2.42% |  | 0.81% | 0.20% | 1.41% |  | 0.37% | 0.09% | 0.64% |
|  |  | Falls | 0.00% | 0.00% | 0.00% |  | 1.48% | 0.55% | 2.42% |  | 0.81% | 0.20% | 1.41% |  | 0.37% | 0.09% | 0.64% |
|  |  | Fires, heat and hot substances | 0.00% | 0.00% | 0.00% |  | 1.48% | 0.55% | 2.42% |  | 0.81% | 0.20% | 1.41% |  | 0.37% | 0.09% | 0.64% |
|  |  | Drownings | 0.00% | 0.00% | 0.00% |  | 1.48% | 0.55% | 2.42% |  | 0.81% | 0.20% | 1.41% |  | 0.37% | 0.09% | 0.64% |
|  |  | Other unintentional injuries | 0.00% | 0.00% | 0.00% |  | 1.48% | 0.55% | 2.42% |  | 0.81% | 0.20% | 1.41% |  | 0.37% | 0.09% | 0.64% |
|  | Intentional injuries | |  |  |  |  |  |  |  |  |  |  |  |  |  |  |  |
|  |  | Self-inflicted injuries | 0.00% | 0.00% | 0.00% |  | 1.48% | 0.55% | 2.42% |  | 0.81% | 0.20% | 1.41% |  | 0.37% | 0.09% | 0.64% |
|  |  | Violence | 1.83% | 1.23% | 2.43% |  | 5.26% | 3.53% | 6.98% |  | 3.05% | 2.05% | 4.04% |  | 1.37% | 0.92% | 1.82% |
|  |  | Other intentional injuries | 0.00% | 0.00% | 0.00% |  | 1.48% | 0.55% | 2.42% |  | 0.81% | 0.20% | 1.41% |  | 0.37% | 0.09% | 0.64% |
| Men | |  |  |  |  |  |  |  |  |  |  |  |  |  |  |  |  |
| Injuries | | |  |  |  |  |  |  |  |  |  |  |  |  |  |  |  |
|  | Unintentional injuries | |  |  |  |  |  |  |  |  |  |  |  |  |  |  |  |
|  |  | Transport injuries | 3.80% | 3.29% | 4.32% |  | 14.82% | 10.35% | 19.29% |  | 10.94% | 7.55% | 14.34% |  | 4.50% | 2.86% | 6.14% |
|  |  | Poisonings | 0.00% | 0.00% | 0.00% |  | 5.03% | 2.39% | 7.67% |  | 4.58% | 2.14% | 7.02% |  | 2.26% | 1.03% | 3.48% |
|  |  | Falls | 0.00% | 0.00% | 0.00% |  | 5.03% | 2.39% | 7.67% |  | 4.58% | 2.14% | 7.02% |  | 2.26% | 1.03% | 3.48% |
|  |  | Fires, heat and hot substances | 0.00% | 0.00% | 0.00% |  | 5.03% | 2.39% | 7.67% |  | 4.58% | 2.14% | 7.02% |  | 2.26% | 1.03% | 3.48% |
|  |  | Drownings | 0.00% | 0.00% | 0.00% |  | 5.03% | 2.39% | 7.67% |  | 4.58% | 2.14% | 7.02% |  | 2.26% | 1.03% | 3.48% |
|  |  | Other unintentional injuries | 0.00% | 0.00% | 0.00% |  | 5.03% | 2.39% | 7.67% |  | 4.58% | 2.14% | 7.02% |  | 2.26% | 1.03% | 3.48% |
|  | Intentional injuries | |  |  |  |  |  |  |  |  |  |  |  |  |  |  |  |
|  |  | Self-inflicted injuries | 0.00% | 0.00% | 0.00% |  | 5.03% | 2.39% | 7.67% |  | 4.58% | 2.14% | 7.02% |  | 2.26% | 1.03% | 3.48% |
|  |  | Violence | 1.83% | 1.23% | 2.43% |  | 5.26% | 3.53% | 6.98% |  | 3.05% | 2.05% | 4.04% |  | 1.37% | 0.92% | 1.82% |
|  |  | Other intentional injuries | 0.00% | 0.00% | 0.00% |  | 5.03% | 2.39% | 7.67% |  | 4.58% | 2.14% | 7.02% |  | 2.26% | 1.03% | 3.48% |

Table 18: Morbidity Alcohol-Attributable Fractions for injuries (with harms to others included): Sub-Saharan Africa Central

|  |  |  | 0 to 14 years of age | | |  | 15 to 34 years of age | | |  | 35 to 64 years of age | | |  | 65 years of age and older | | |
| --- | --- | --- | --- | --- | --- | --- | --- | --- | --- | --- | --- | --- | --- | --- | --- | --- | --- |
|  |  |  | Point estimate | Lower 95% CI | Upper 95% CI |  | Point estimate | Lower 95% CI | Upper 95% CI |  | Point estimate | Lower 95% CI | Upper 95% CI |  | Point estimate | Lower 95% CI | Upper 95% CI |
| Women | | |  |  |  |  |  |  |  |  |  |  |  |  |  |  |  |
| Injuries | | |  |  |  |  |  |  |  |  |  |  |  |  |  |  |  |
|  | Unintentional injuries | |  |  |  |  |  |  |  |  |  |  |  |  |  |  |  |
|  |  | Transport injuries | 4.13% | 4.08% | 4.18% |  | 7.87% | 6.71% | 9.02% |  | 6.37% | 5.12% | 7.62% |  | 1.63% | 1.23% | 2.02% |
|  |  | Poisonings | 0.00% | 0.00% | 0.00% |  | 1.82% | 0.63% | 3.01% |  | 1.89% | 0.37% | 3.41% |  | 0.41% | 0.11% | 0.71% |
|  |  | Falls | 0.00% | 0.00% | 0.00% |  | 1.82% | 0.63% | 3.01% |  | 1.89% | 0.37% | 3.41% |  | 0.41% | 0.11% | 0.71% |
|  |  | Fires, heat and hot substances | 0.00% | 0.00% | 0.00% |  | 1.82% | 0.63% | 3.01% |  | 1.89% | 0.37% | 3.41% |  | 0.41% | 0.11% | 0.71% |
|  |  | Drownings | 0.00% | 0.00% | 0.00% |  | 1.82% | 0.63% | 3.01% |  | 1.89% | 0.37% | 3.41% |  | 0.41% | 0.11% | 0.71% |
|  |  | Other unintentional injuries | 0.00% | 0.00% | 0.00% |  | 1.82% | 0.63% | 3.01% |  | 1.89% | 0.37% | 3.41% |  | 0.41% | 0.11% | 0.71% |
|  | Intentional injuries | |  |  |  |  |  |  |  |  |  |  |  |  |  |  |  |
|  |  | Self-inflicted injuries | 0.00% | 0.00% | 0.00% |  | 1.82% | 0.63% | 3.01% |  | 1.89% | 0.37% | 3.41% |  | 0.41% | 0.11% | 0.71% |
|  |  | Violence | 1.97% | 1.32% | 2.63% |  | 5.65% | 3.78% | 7.52% |  | 3.27% | 2.19% | 4.36% |  | 1.47% | 0.99% | 1.96% |
|  |  | Other intentional injuries | 0.00% | 0.00% | 0.00% |  | 1.82% | 0.63% | 3.01% |  | 1.89% | 0.37% | 3.41% |  | 0.41% | 0.11% | 0.71% |
| Men | |  |  |  |  |  |  |  |  |  |  |  |  |  |  |  |  |
| Injuries | | |  |  |  |  |  |  |  |  |  |  |  |  |  |  |  |
|  | Unintentional injuries | |  |  |  |  |  |  |  |  |  |  |  |  |  |  |  |
|  |  | Transport injuries | 4.13% | 3.54% | 4.72% |  | 12.49% | 8.89% | 16.09% |  | 11.73% | 8.14% | 15.31% |  | 3.15% | 2.06% | 4.24% |
|  |  | Poisonings | 0.00% | 0.00% | 0.00% |  | 4.62% | 2.03% | 7.20% |  | 5.33% | 2.24% | 8.43% |  | 1.39% | 0.59% | 2.19% |
|  |  | Falls | 0.00% | 0.00% | 0.00% |  | 4.62% | 2.03% | 7.20% |  | 5.33% | 2.24% | 8.43% |  | 1.39% | 0.59% | 2.19% |
|  |  | Fires, heat and hot substances | 0.00% | 0.00% | 0.00% |  | 4.62% | 2.03% | 7.20% |  | 5.33% | 2.24% | 8.43% |  | 1.39% | 0.59% | 2.19% |
|  |  | Drownings | 0.00% | 0.00% | 0.00% |  | 4.62% | 2.03% | 7.20% |  | 5.33% | 2.24% | 8.43% |  | 1.39% | 0.59% | 2.19% |
|  |  | Other unintentional injuries | 0.00% | 0.00% | 0.00% |  | 4.62% | 2.03% | 7.20% |  | 5.33% | 2.24% | 8.43% |  | 1.39% | 0.59% | 2.19% |
|  | Intentional injuries | |  |  |  |  |  |  |  |  |  |  |  |  |  |  |  |
|  |  | Self-inflicted injuries | 0.00% | 0.00% | 0.00% |  | 4.62% | 2.03% | 7.20% |  | 5.33% | 2.24% | 8.43% |  | 1.39% | 0.59% | 2.19% |
|  |  | Violence | 1.97% | 1.32% | 2.63% |  | 5.65% | 3.78% | 7.52% |  | 3.27% | 2.19% | 4.36% |  | 1.47% | 0.99% | 1.96% |
|  |  | Other intentional injuries | 0.00% | 0.00% | 0.00% |  | 4.62% | 2.03% | 7.20% |  | 5.33% | 2.24% | 8.43% |  | 1.39% | 0.59% | 2.19% |

Table 19: Morbidity Alcohol-Attributable Fractions for injuries (with harms to others included): Sub-Saharan Africa East

|  |  |  | 0 to 14 years of age | | |  | 15 to 34 years of age | | |  | 35 to 64 years of age | | |  | 65 years of age and older | | |
| --- | --- | --- | --- | --- | --- | --- | --- | --- | --- | --- | --- | --- | --- | --- | --- | --- | --- |
|  |  |  | Point estimate | Lower 95% CI | Upper 95% CI |  | Point estimate | Lower 95% CI | Upper 95% CI |  | Point estimate | Lower 95% CI | Upper 95% CI |  | Point estimate | Lower 95% CI | Upper 95% CI |
| Women | | |  |  |  |  |  |  |  |  |  |  |  |  |  |  |  |
| Injuries | | |  |  |  |  |  |  |  |  |  |  |  |  |  |  |  |
|  | Unintentional injuries | |  |  |  |  |  |  |  |  |  |  |  |  |  |  |  |
|  |  | Transport injuries | 3.14% | 3.07% | 3.20% |  | 4.97% | 4.23% | 5.72% |  | 6.08% | 2.28% | 10.05% |  | 1.50% | 0.93% | 2.07% |
|  |  | Poisonings | 0.00% | 0.00% | 0.00% |  | 0.89% | 0.00% | 2.37% |  | 2.84% | 0.00% | 7.12% |  | 0.81% | 0.00% | 2.05% |
|  |  | Falls | 0.00% | 0.00% | 0.00% |  | 0.89% | 0.00% | 2.37% |  | 2.84% | 0.00% | 7.12% |  | 0.81% | 0.00% | 2.05% |
|  |  | Fires, heat and hot substances | 0.00% | 0.00% | 0.00% |  | 0.89% | 0.00% | 2.37% |  | 2.84% | 0.00% | 7.12% |  | 0.81% | 0.00% | 2.05% |
|  |  | Drownings | 0.00% | 0.00% | 0.00% |  | 0.89% | 0.00% | 2.37% |  | 2.84% | 0.00% | 7.12% |  | 0.81% | 0.00% | 2.05% |
|  |  | Other unintentional injuries | 0.00% | 0.00% | 0.00% |  | 0.89% | 0.00% | 2.37% |  | 2.84% | 0.00% | 7.12% |  | 0.81% | 0.00% | 2.05% |
|  | Intentional injuries | |  |  |  |  |  |  |  |  |  |  |  |  |  |  |  |
|  |  | Self-inflicted injuries | 0.00% | 0.00% | 0.00% |  | 0.89% | 0.00% | 2.37% |  | 2.84% | 0.00% | 7.12% |  | 0.81% | 0.00% | 2.05% |
|  |  | Violence | 3.12% | 1.47% | 4.77% |  | 8.94% | 4.22% | 13.66% |  | 5.18% | 2.44% | 7.92% |  | 2.33% | 1.10% | 3.56% |
|  |  | Other intentional injuries | 0.00% | 0.00% | 0.00% |  | 0.89% | 0.00% | 2.37% |  | 2.84% | 0.00% | 7.12% |  | 0.81% | 0.00% | 2.05% |
| Men | |  |  |  |  |  |  |  |  |  |  |  |  |  |  |  |  |
| Injuries | | |  |  |  |  |  |  |  |  |  |  |  |  |  |  |  |
|  | Unintentional injuries | |  |  |  |  |  |  |  |  |  |  |  |  |  |  |  |
|  |  | Transport injuries | 3.14% | 2.43% | 3.85% |  | 7.52% | 4.39% | 10.65% |  | 14.75% | 1.76% | 28.32% |  | 3.75% | 1.81% | 5.68% |
|  |  | Poisonings | 0.00% | 0.00% | 0.00% |  | 5.08% | 1.30% | 8.86% |  | 14.88% | 4.41% | 25.36% |  | 5.42% | 1.32% | 9.53% |
|  |  | Falls | 0.00% | 0.00% | 0.00% |  | 5.08% | 1.30% | 8.86% |  | 14.88% | 4.41% | 25.36% |  | 5.42% | 1.32% | 9.53% |
|  |  | Fires, heat and hot substances | 0.00% | 0.00% | 0.00% |  | 5.08% | 1.30% | 8.86% |  | 14.88% | 4.41% | 25.36% |  | 5.42% | 1.32% | 9.53% |
|  |  | Drownings | 0.00% | 0.00% | 0.00% |  | 5.08% | 1.30% | 8.86% |  | 14.88% | 4.41% | 25.36% |  | 5.42% | 1.32% | 9.53% |
|  |  | Other unintentional injuries | 0.00% | 0.00% | 0.00% |  | 5.08% | 1.30% | 8.86% |  | 14.88% | 4.41% | 25.36% |  | 5.42% | 1.32% | 9.53% |
|  | Intentional injuries | |  |  |  |  |  |  |  |  |  |  |  |  |  |  |  |
|  |  | Self-inflicted injuries | 0.00% | 0.00% | 0.00% |  | 5.08% | 1.30% | 8.86% |  | 14.88% | 4.41% | 25.36% |  | 5.42% | 1.32% | 9.53% |
|  |  | Violence | 3.12% | 1.47% | 4.77% |  | 8.94% | 4.22% | 13.66% |  | 5.18% | 2.44% | 7.92% |  | 2.33% | 1.10% | 3.56% |
|  |  | Other intentional injuries | 0.00% | 0.00% | 0.00% |  | 5.08% | 1.30% | 8.86% |  | 14.88% | 4.41% | 25.36% |  | 5.42% | 1.32% | 9.53% |

Table 20: Morbidity Alcohol-Attributable Fractions for injuries (with harms to others included): Sub-Saharan Africa Southern

|  |  |  | 0 to 14 years of age | | |  | 15 to 34 years of age | | |  | 35 to 64 years of age | | |  | 65 years of age and older | | |
| --- | --- | --- | --- | --- | --- | --- | --- | --- | --- | --- | --- | --- | --- | --- | --- | --- | --- |
|  |  |  | Point estimate | Lower 95% CI | Upper 95% CI |  | Point estimate | Lower 95% CI | Upper 95% CI |  | Point estimate | Lower 95% CI | Upper 95% CI |  | Point estimate | Lower 95% CI | Upper 95% CI |
| Women | | |  |  |  |  |  |  |  |  |  |  |  |  |  |  |  |
| Injuries | | |  |  |  |  |  |  |  |  |  |  |  |  |  |  |  |
|  | Unintentional injuries | |  |  |  |  |  |  |  |  |  |  |  |  |  |  |  |
|  |  | Transport injuries | 15.99% | 15.19% | 16.78% |  | 25.96% | 20.86% | 31.06% |  | 19.18% | 14.28% | 24.08% |  | 3.86% | 3.29% | 4.43% |
|  |  | Poisonings | 0.00% | 0.00% | 0.00% |  | 5.78% | 0.00% | 13.28% |  | 4.57% | 0.00% | 10.57% |  | 0.83% | 0.00% | 1.81% |
|  |  | Falls | 0.00% | 0.00% | 0.00% |  | 5.78% | 0.00% | 13.28% |  | 4.57% | 0.00% | 10.57% |  | 0.83% | 0.00% | 1.81% |
|  |  | Fires, heat and hot substances | 0.00% | 0.00% | 0.00% |  | 5.78% | 0.00% | 13.28% |  | 4.57% | 0.00% | 10.57% |  | 0.83% | 0.00% | 1.81% |
|  |  | Drownings | 0.00% | 0.00% | 0.00% |  | 5.78% | 0.00% | 13.28% |  | 4.57% | 0.00% | 10.57% |  | 0.83% | 0.00% | 1.81% |
|  |  | Other unintentional injuries | 0.00% | 0.00% | 0.00% |  | 5.78% | 0.00% | 13.28% |  | 4.57% | 0.00% | 10.57% |  | 0.83% | 0.00% | 1.81% |
|  | Intentional injuries | |  |  |  |  |  |  |  |  |  |  |  |  |  |  |  |
|  |  | Self-inflicted injuries | 0.00% | 0.00% | 0.00% |  | 5.78% | 0.00% | 13.28% |  | 4.57% | 0.00% | 10.57% |  | 0.83% | 0.00% | 1.81% |
|  |  | Violence | 8.62% | 4.71% | 12.54% |  | 24.72% | 13.49% | 35.95% |  | 14.33% | 7.82% | 20.83% |  | 6.45% | 3.52% | 9.38% |
|  |  | Other intentional injuries | 0.00% | 0.00% | 0.00% |  | 5.78% | 0.00% | 13.28% |  | 4.57% | 0.00% | 10.57% |  | 0.83% | 0.00% | 1.81% |
| Men | |  |  |  |  |  |  |  |  |  |  |  |  |  |  |  |  |
| Injuries | | |  |  |  |  |  |  |  |  |  |  |  |  |  |  |  |
|  | Unintentional injuries | |  |  |  |  |  |  |  |  |  |  |  |  |  |  |  |
|  |  | Transport injuries | 15.99% | 6.79% | 25.18% |  | 49.55% | 17.85% | 60.00% |  | 43.71% | 17.12% | 60.00% |  | 7.22% | 4.14% | 10.30% |
|  |  | Poisonings | 0.00% | 0.00% | 0.00% |  | 22.82% | 9.68% | 35.96% |  | 22.73% | 9.46% | 36.01% |  | 5.13% | 1.57% | 8.70% |
|  |  | Falls | 0.00% | 0.00% | 0.00% |  | 22.82% | 9.68% | 35.96% |  | 22.73% | 9.46% | 36.01% |  | 5.13% | 1.57% | 8.70% |
|  |  | Fires, heat and hot substances | 0.00% | 0.00% | 0.00% |  | 22.82% | 9.68% | 35.96% |  | 22.73% | 9.46% | 36.01% |  | 5.13% | 1.57% | 8.70% |
|  |  | Drownings | 0.00% | 0.00% | 0.00% |  | 22.82% | 9.68% | 35.96% |  | 22.73% | 9.46% | 36.01% |  | 5.13% | 1.57% | 8.70% |
|  |  | Other unintentional injuries | 0.00% | 0.00% | 0.00% |  | 22.82% | 9.68% | 35.96% |  | 22.73% | 9.46% | 36.01% |  | 5.13% | 1.57% | 8.70% |
|  | Intentional injuries | |  |  |  |  |  |  |  |  |  |  |  |  |  |  |  |
|  |  | Self-inflicted injuries | 0.00% | 0.00% | 0.00% |  | 22.82% | 9.68% | 35.96% |  | 22.73% | 9.46% | 36.01% |  | 5.13% | 1.57% | 8.70% |
|  |  | Violence | 8.62% | 4.71% | 12.54% |  | 24.72% | 13.49% | 35.95% |  | 14.33% | 7.82% | 20.83% |  | 6.45% | 3.52% | 9.38% |
|  |  | Other intentional injuries | 0.00% | 0.00% | 0.00% |  | 22.82% | 9.68% | 35.96% |  | 22.73% | 9.46% | 36.01% |  | 5.13% | 1.57% | 8.70% |

Table 21: Morbidity Alcohol-Attributable Fractions for injuries (with harms to others included): Sub-Saharan Africa West

|  |  |  | 0 to 14 years of age | | |  | 15 to 34 years of age | | |  | 35 to 64 years of age | | |  | 65 years of age and older | | |
| --- | --- | --- | --- | --- | --- | --- | --- | --- | --- | --- | --- | --- | --- | --- | --- | --- | --- |
|  |  |  | Point estimate | Lower 95% CI | Upper 95% CI |  | Point estimate | Lower 95% CI | Upper 95% CI |  | Point estimate | Lower 95% CI | Upper 95% CI |  | Point estimate | Lower 95% CI | Upper 95% CI |
| Women | | |  |  |  |  |  |  |  |  |  |  |  |  |  |  |  |
| Injuries | | |  |  |  |  |  |  |  |  |  |  |  |  |  |  |  |
|  | Unintentional injuries | |  |  |  |  |  |  |  |  |  |  |  |  |  |  |  |
|  |  | Transport injuries | 3.09% | 3.03% | 3.15% |  | 5.22% | 4.22% | 6.22% |  | 6.08% | 2.59% | 9.57% |  | 1.46% | 1.11% | 1.80% |
|  |  | Poisonings | 0.00% | 0.00% | 0.00% |  | 1.96% | 0.04% | 3.87% |  | 6.10% | 0.62% | 11.57% |  | 1.57% | 0.38% | 2.77% |
|  |  | Falls | 0.00% | 0.00% | 0.00% |  | 1.96% | 0.04% | 3.87% |  | 6.10% | 0.62% | 11.57% |  | 1.57% | 0.38% | 2.77% |
|  |  | Fires, heat and hot substances | 0.00% | 0.00% | 0.00% |  | 1.96% | 0.04% | 3.87% |  | 6.10% | 0.62% | 11.57% |  | 1.57% | 0.38% | 2.77% |
|  |  | Drownings | 0.00% | 0.00% | 0.00% |  | 1.96% | 0.04% | 3.87% |  | 6.10% | 0.62% | 11.57% |  | 1.57% | 0.38% | 2.77% |
|  |  | Other unintentional injuries | 0.00% | 0.00% | 0.00% |  | 1.96% | 0.04% | 3.87% |  | 6.10% | 0.62% | 11.57% |  | 1.57% | 0.38% | 2.77% |
|  | Intentional injuries | |  |  |  |  |  |  |  |  |  |  |  |  |  |  |  |
|  |  | Self-inflicted injuries | 0.00% | 0.00% | 0.00% |  | 1.96% | 0.04% | 3.87% |  | 6.10% | 0.62% | 11.57% |  | 1.57% | 0.38% | 2.77% |
|  |  | Violence | 4.37% | 2.55% | 6.19% |  | 12.52% | 7.31% | 17.73% |  | 7.26% | 4.23% | 10.28% |  | 3.27% | 1.91% | 4.62% |
|  |  | Other intentional injuries | 0.00% | 0.00% | 0.00% |  | 1.96% | 0.04% | 3.87% |  | 6.10% | 0.62% | 11.57% |  | 1.57% | 0.38% | 2.77% |
| Men | |  |  |  |  |  |  |  |  |  |  |  |  |  |  |  |  |
| Injuries | | |  |  |  |  |  |  |  |  |  |  |  |  |  |  |  |
|  | Unintentional injuries | |  |  |  |  |  |  |  |  |  |  |  |  |  |  |  |
|  |  | Transport injuries | 3.09% | 2.44% | 3.74% |  | 7.93% | 4.36% | 11.50% |  | 13.37% | 2.79% | 23.94% |  | 3.26% | 2.23% | 4.30% |
|  |  | Poisonings | 0.00% | 0.00% | 0.00% |  | 7.70% | 2.67% | 12.73% |  | 16.43% | 6.28% | 26.59% |  | 4.17% | 1.51% | 6.83% |
|  |  | Falls | 0.00% | 0.00% | 0.00% |  | 7.70% | 2.67% | 12.73% |  | 16.43% | 6.28% | 26.59% |  | 4.17% | 1.51% | 6.83% |
|  |  | Fires, heat and hot substances | 0.00% | 0.00% | 0.00% |  | 7.70% | 2.67% | 12.73% |  | 16.43% | 6.28% | 26.59% |  | 4.17% | 1.51% | 6.83% |
|  |  | Drownings | 0.00% | 0.00% | 0.00% |  | 7.70% | 2.67% | 12.73% |  | 16.43% | 6.28% | 26.59% |  | 4.17% | 1.51% | 6.83% |
|  |  | Other unintentional injuries | 0.00% | 0.00% | 0.00% |  | 7.70% | 2.67% | 12.73% |  | 16.43% | 6.28% | 26.59% |  | 4.17% | 1.51% | 6.83% |
|  | Intentional injuries | |  |  |  |  |  |  |  |  |  |  |  |  |  |  |  |
|  |  | Self-inflicted injuries | 0.00% | 0.00% | 0.00% |  | 7.70% | 2.67% | 12.73% |  | 16.43% | 6.28% | 26.59% |  | 4.17% | 1.51% | 6.83% |
|  |  | Violence | 4.37% | 2.55% | 6.19% |  | 12.52% | 7.31% | 17.73% |  | 7.26% | 4.23% | 10.28% |  | 3.27% | 1.91% | 4.62% |
|  |  | Other intentional injuries | 0.00% | 0.00% | 0.00% |  | 7.70% | 2.67% | 12.73% |  | 16.43% | 6.28% | 26.59% |  | 4.17% | 1.51% | 6.83% |

Table 22: Mortality Alcohol-Attributable Fractions for injuries (with harms to others included): Asia Pacific [High Income]

|  |  |  | 0 to 14 years of age | | |  | 15 to 34 years of age | | |  | 35 to 64 years of age | | |  | 65 years of age and older | | |
| --- | --- | --- | --- | --- | --- | --- | --- | --- | --- | --- | --- | --- | --- | --- | --- | --- | --- |
|  |  |  | Point estimate | Lower 95% CI | Upper 95% CI |  | Point estimate | Lower 95% CI | Upper 95% CI |  | Point estimate | Lower 95% CI | Upper 95% CI |  | Point estimate | Lower 95% CI | Upper 95% CI |
| Women | | |  |  |  |  |  |  |  |  |  |  |  |  |  |  |  |
| Injuries | | |  |  |  |  |  |  |  |  |  |  |  |  |  |  |  |
|  | Unintentional injuries | |  |  |  |  |  |  |  |  |  |  |  |  |  |  |  |
|  |  | Transport injuries | 5.24% | 1.15% | 9.33% |  | 11.86% | 3.99% | 19.73% |  | 11.87% | 4.29% | 19.45% |  | 3.59% | 0.96% | 6.22% |
|  |  | Poisonings | 0.00% | 0.00% | 0.00% |  | 4.09% | 1.78% | 6.41% |  | 6.38% | 2.46% | 10.29% |  | 1.86% | 0.80% | 2.92% |
|  |  | Falls | 0.00% | 0.00% | 0.00% |  | 4.09% | 1.78% | 6.41% |  | 6.38% | 2.46% | 10.29% |  | 1.86% | 0.80% | 2.92% |
|  |  | Fires, heat and hot substances | 0.00% | 0.00% | 0.00% |  | 4.09% | 1.78% | 6.41% |  | 6.38% | 2.46% | 10.29% |  | 1.86% | 0.80% | 2.92% |
|  |  | Drownings | 0.00% | 0.00% | 0.00% |  | 4.09% | 1.78% | 6.41% |  | 6.38% | 2.46% | 10.29% |  | 1.86% | 0.80% | 2.92% |
|  |  | Other unintentional injuries | 0.00% | 0.00% | 0.00% |  | 4.09% | 1.78% | 6.41% |  | 6.38% | 2.46% | 10.29% |  | 1.86% | 0.80% | 2.92% |
|  | Intentional injuries | |  |  |  |  |  |  |  |  |  |  |  |  |  |  |  |
|  |  | Self-inflicted injuries | 0.00% | 0.00% | 0.00% |  | 4.09% | 1.78% | 6.41% |  | 6.38% | 2.46% | 10.29% |  | 1.86% | 0.80% | 2.92% |
|  |  | Violence | 9.82% | 5.88% | 13.75% |  | 28.14% | 16.86% | 39.41% |  | 16.31% | 9.77% | 22.84% |  | 7.34% | 4.40% | 10.28% |
|  |  | Other intentional injuries | 0.00% | 0.00% | 0.00% |  | 4.09% | 1.78% | 6.41% |  | 6.38% | 2.46% | 10.29% |  | 1.86% | 0.80% | 2.92% |
| Men | |  |  |  |  |  |  |  |  |  |  |  |  |  |  |  |  |
| Injuries | | |  |  |  |  |  |  |  |  |  |  |  |  |  |  |  |
|  | Unintentional injuries | |  |  |  |  |  |  |  |  |  |  |  |  |  |  |  |
|  |  | Transport injuries | 5.24% | 5.15% | 5.34% |  | 15.75% | 13.06% | 18.44% |  | 21.94% | 14.31% | 29.56% |  | 6.39% | 4.68% | 8.10% |
|  |  | Poisonings | 0.00% | 0.00% | 0.00% |  | 16.49% | 6.90% | 26.08% |  | 37.47% | 17.84% | 57.09% |  | 12.55% | 5.04% | 20.06% |
|  |  | Falls | 0.00% | 0.00% | 0.00% |  | 16.49% | 6.90% | 26.08% |  | 37.47% | 17.84% | 57.09% |  | 12.55% | 5.04% | 20.06% |
|  |  | Fires, heat and hot substances | 0.00% | 0.00% | 0.00% |  | 16.49% | 6.90% | 26.08% |  | 37.47% | 17.84% | 57.09% |  | 12.55% | 5.04% | 20.06% |
|  |  | Drownings | 0.00% | 0.00% | 0.00% |  | 16.49% | 6.90% | 26.08% |  | 37.47% | 17.84% | 57.09% |  | 12.55% | 5.04% | 20.06% |
|  |  | Other unintentional injuries | 0.00% | 0.00% | 0.00% |  | 16.49% | 6.90% | 26.08% |  | 37.47% | 17.84% | 57.09% |  | 12.55% | 5.04% | 20.06% |
|  | Intentional injuries | |  |  |  |  |  |  |  |  |  |  |  |  |  |  |  |
|  |  | Self-inflicted injuries | 0.00% | 0.00% | 0.00% |  | 16.49% | 6.90% | 26.08% |  | 37.47% | 17.84% | 57.09% |  | 12.55% | 5.04% | 20.06% |
|  |  | Violence | 9.82% | 5.88% | 13.75% |  | 28.14% | 16.86% | 39.41% |  | 16.31% | 9.77% | 22.84% |  | 7.34% | 4.40% | 10.28% |
|  |  | Other intentional injuries | 0.00% | 0.00% | 0.00% |  | 16.49% | 6.90% | 26.08% |  | 37.47% | 17.84% | 57.09% |  | 12.55% | 5.04% | 20.06% |

Table 23: Mortality Alcohol-Attributable Fractions for injuries (with harms to others included): Asia Central

|  |  |  | 0 to 14 years of age | | |  | 15 to 34 years of age | | |  | 35 to 64 years of age | | |  | 65 years of age and older | | |
| --- | --- | --- | --- | --- | --- | --- | --- | --- | --- | --- | --- | --- | --- | --- | --- | --- | --- |
|  |  |  | Point estimate | Lower 95% CI | Upper 95% CI |  | Point estimate | Lower 95% CI | Upper 95% CI |  | Point estimate | Lower 95% CI | Upper 95% CI |  | Point estimate | Lower 95% CI | Upper 95% CI |
| Women | | |  |  |  |  |  |  |  |  |  |  |  |  |  |  |  |
| Injuries | | |  |  |  |  |  |  |  |  |  |  |  |  |  |  |  |
|  | Unintentional injuries | |  |  |  |  |  |  |  |  |  |  |  |  |  |  |  |
|  |  | Transport injuries | 13.59% | 4.65% | 22.53% |  | 29.77% | 14.49% | 45.05% |  | 26.30% | 13.03% | 39.56% |  | 10.61% | 4.14% | 17.08% |
|  |  | Poisonings | 0.00% | 0.00% | 0.00% |  | 3.15% | 0.37% | 5.92% |  | 7.58% | 1.07% | 14.09% |  | 0.88% | 0.00% | 1.81% |
|  |  | Falls | 0.00% | 0.00% | 0.00% |  | 3.15% | 0.37% | 5.92% |  | 7.58% | 1.07% | 14.09% |  | 0.88% | 0.00% | 1.81% |
|  |  | Fires, heat and hot substances | 0.00% | 0.00% | 0.00% |  | 3.15% | 0.37% | 5.92% |  | 7.58% | 1.07% | 14.09% |  | 0.88% | 0.00% | 1.81% |
|  |  | Drownings | 0.00% | 0.00% | 0.00% |  | 3.15% | 0.37% | 5.92% |  | 7.58% | 1.07% | 14.09% |  | 0.88% | 0.00% | 1.81% |
|  |  | Other unintentional injuries | 0.00% | 0.00% | 0.00% |  | 3.15% | 0.37% | 5.92% |  | 7.58% | 1.07% | 14.09% |  | 0.88% | 0.00% | 1.81% |
|  | Intentional injuries | |  |  |  |  |  |  |  |  |  |  |  |  |  |  |  |
|  |  | Self-inflicted injuries | 0.00% | 0.00% | 0.00% |  | 3.15% | 0.37% | 5.92% |  | 7.58% | 1.07% | 14.09% |  | 0.88% | 0.00% | 1.81% |
|  |  | Violence | 8.51% | 5.38% | 11.63% |  | 24.38% | 15.43% | 33.33% |  | 14.13% | 8.94% | 19.32% |  | 6.36% | 4.02% | 8.69% |
|  |  | Other intentional injuries | 0.00% | 0.00% | 0.00% |  | 3.15% | 0.37% | 5.92% |  | 7.58% | 1.07% | 14.09% |  | 0.88% | 0.00% | 1.81% |
| Men | |  |  |  |  |  |  |  |  |  |  |  |  |  |  |  |  |
| Injuries | | |  |  |  |  |  |  |  |  |  |  |  |  |  |  |  |
|  | Unintentional injuries | |  |  |  |  |  |  |  |  |  |  |  |  |  |  |  |
|  |  | Transport injuries | 13.59% | 13.17% | 14.02% |  | 40.15% | 32.64% | 47.65% |  | 42.99% | 33.34% | 52.64% |  | 20.45% | 14.92% | 25.98% |
|  |  | Poisonings | 0.00% | 0.00% | 0.00% |  | 21.89% | 8.77% | 35.01% |  | 26.50% | 12.29% | 40.70% |  | 12.86% | 5.59% | 20.13% |
|  |  | Falls | 0.00% | 0.00% | 0.00% |  | 21.89% | 8.77% | 35.01% |  | 26.50% | 12.29% | 40.70% |  | 12.86% | 5.59% | 20.13% |
|  |  | Fires, heat and hot substances | 0.00% | 0.00% | 0.00% |  | 21.89% | 8.77% | 35.01% |  | 26.50% | 12.29% | 40.70% |  | 12.86% | 5.59% | 20.13% |
|  |  | Drownings | 0.00% | 0.00% | 0.00% |  | 21.89% | 8.77% | 35.01% |  | 26.50% | 12.29% | 40.70% |  | 12.86% | 5.59% | 20.13% |
|  |  | Other unintentional injuries | 0.00% | 0.00% | 0.00% |  | 21.89% | 8.77% | 35.01% |  | 26.50% | 12.29% | 40.70% |  | 12.86% | 5.59% | 20.13% |
|  | Intentional injuries | |  |  |  |  |  |  |  |  |  |  |  |  |  |  |  |
|  |  | Self-inflicted injuries | 0.00% | 0.00% | 0.00% |  | 21.89% | 8.77% | 35.01% |  | 26.50% | 12.29% | 40.70% |  | 12.86% | 5.59% | 20.13% |
|  |  | Violence | 8.51% | 5.38% | 11.63% |  | 24.38% | 15.43% | 33.33% |  | 14.13% | 8.94% | 19.32% |  | 6.36% | 4.02% | 8.69% |
|  |  | Other intentional injuries | 0.00% | 0.00% | 0.00% |  | 21.89% | 8.77% | 35.01% |  | 26.50% | 12.29% | 40.70% |  | 12.86% | 5.59% | 20.13% |

Table 24: Mortality Alcohol-Attributable Fractions for injuries (with harms to others included): Asia East

|  |  |  | 0 to 14 years of age | | |  | 15 to 34 years of age | | |  | 35 to 64 years of age | | |  | 65 years of age and older | | |
| --- | --- | --- | --- | --- | --- | --- | --- | --- | --- | --- | --- | --- | --- | --- | --- | --- | --- |
|  |  |  | Point estimate | Lower 95% CI | Upper 95% CI |  | Point estimate | Lower 95% CI | Upper 95% CI |  | Point estimate | Lower 95% CI | Upper 95% CI |  | Point estimate | Lower 95% CI | Upper 95% CI |
| Women | | |  |  |  |  |  |  |  |  |  |  |  |  |  |  |  |
| Injuries | | |  |  |  |  |  |  |  |  |  |  |  |  |  |  |  |
|  | Unintentional injuries | |  |  |  |  |  |  |  |  |  |  |  |  |  |  |  |
|  |  | Transport injuries | 3.38% | 0.54% | 6.21% |  | 7.12% | 1.76% | 12.47% |  | 6.71% | 1.83% | 11.59% |  | 2.27% | 0.45% | 4.09% |
|  |  | Poisonings | 0.00% | 0.00% | 0.00% |  | 0.60% | 0.09% | 1.11% |  | 2.98% | 0.00% | 6.80% |  | 1.21% | 0.00% | 2.46% |
|  |  | Falls | 0.00% | 0.00% | 0.00% |  | 0.60% | 0.09% | 1.11% |  | 2.98% | 0.00% | 6.80% |  | 1.21% | 0.00% | 2.46% |
|  |  | Fires, heat and hot substances | 0.00% | 0.00% | 0.00% |  | 0.60% | 0.09% | 1.11% |  | 2.98% | 0.00% | 6.80% |  | 1.21% | 0.00% | 2.46% |
|  |  | Drownings | 0.00% | 0.00% | 0.00% |  | 0.60% | 0.09% | 1.11% |  | 2.98% | 0.00% | 6.80% |  | 1.21% | 0.00% | 2.46% |
|  |  | Other unintentional injuries | 0.00% | 0.00% | 0.00% |  | 0.60% | 0.09% | 1.11% |  | 2.98% | 0.00% | 6.80% |  | 1.21% | 0.00% | 2.46% |
|  | Intentional injuries | |  |  |  |  |  |  |  |  |  |  |  |  |  |  |  |
|  |  | Self-inflicted injuries | 0.00% | 0.00% | 0.00% |  | 0.60% | 0.09% | 1.11% |  | 2.98% | 0.00% | 6.80% |  | 1.21% | 0.00% | 2.46% |
|  |  | Violence | 5.87% | 2.79% | 8.94% |  | 16.82% | 8.00% | 25.64% |  | 9.75% | 4.63% | 14.86% |  | 4.39% | 2.09% | 6.69% |
|  |  | Other intentional injuries | 0.00% | 0.00% | 0.00% |  | 0.60% | 0.09% | 1.11% |  | 2.98% | 0.00% | 6.80% |  | 1.21% | 0.00% | 2.46% |
| Men | |  |  |  |  |  |  |  |  |  |  |  |  |  |  |  |  |
| Injuries | | |  |  |  |  |  |  |  |  |  |  |  |  |  |  |  |
|  | Unintentional injuries | |  |  |  |  |  |  |  |  |  |  |  |  |  |  |  |
|  |  | Transport injuries | 3.38% | 3.33% | 3.42% |  | 9.17% | 7.87% | 10.47% |  | 14.34% | 8.79% | 19.90% |  | 5.32% | 3.32% | 7.32% |
|  |  | Poisonings | 0.00% | 0.00% | 0.00% |  | 5.13% | 2.33% | 7.92% |  | 26.24% | 9.95% | 42.53% |  | 13.32% | 4.31% | 22.33% |
|  |  | Falls | 0.00% | 0.00% | 0.00% |  | 5.13% | 2.33% | 7.92% |  | 26.24% | 9.95% | 42.53% |  | 13.32% | 4.31% | 22.33% |
|  |  | Fires, heat and hot substances | 0.00% | 0.00% | 0.00% |  | 5.13% | 2.33% | 7.92% |  | 26.24% | 9.95% | 42.53% |  | 13.32% | 4.31% | 22.33% |
|  |  | Drownings | 0.00% | 0.00% | 0.00% |  | 5.13% | 2.33% | 7.92% |  | 26.24% | 9.95% | 42.53% |  | 13.32% | 4.31% | 22.33% |
|  |  | Other unintentional injuries | 0.00% | 0.00% | 0.00% |  | 5.13% | 2.33% | 7.92% |  | 26.24% | 9.95% | 42.53% |  | 13.32% | 4.31% | 22.33% |
|  | Intentional injuries | |  |  |  |  |  |  |  |  |  |  |  |  |  |  |  |
|  |  | Self-inflicted injuries | 0.00% | 0.00% | 0.00% |  | 5.13% | 2.33% | 7.92% |  | 26.24% | 9.95% | 42.53% |  | 13.32% | 4.31% | 22.33% |
|  |  | Violence | 5.87% | 2.79% | 8.94% |  | 16.82% | 8.00% | 25.64% |  | 9.75% | 4.63% | 14.86% |  | 4.39% | 2.09% | 6.69% |
|  |  | Other intentional injuries | 0.00% | 0.00% | 0.00% |  | 5.13% | 2.33% | 7.92% |  | 26.24% | 9.95% | 42.53% |  | 13.32% | 4.31% | 22.33% |

Table 25: Mortality Alcohol-Attributable Fractions for injuries (with harms to others included): Asia South

|  |  |  | 0 to 14 years of age | | |  | 15 to 34 years of age | | |  | 35 to 64 years of age | | |  | 65 years of age and older | | |
| --- | --- | --- | --- | --- | --- | --- | --- | --- | --- | --- | --- | --- | --- | --- | --- | --- | --- |
|  |  |  | Point estimate | Lower 95% CI | Upper 95% CI |  | Point estimate | Lower 95% CI | Upper 95% CI |  | Point estimate | Lower 95% CI | Upper 95% CI |  | Point estimate | Lower 95% CI | Upper 95% CI |
| Women | | |  |  |  |  |  |  |  |  |  |  |  |  |  |  |  |
| Injuries | | |  |  |  |  |  |  |  |  |  |  |  |  |  |  |  |
|  | Unintentional injuries | |  |  |  |  |  |  |  |  |  |  |  |  |  |  |  |
|  |  | Transport injuries | 4.45% | 0.12% | 8.79% |  | 8.93% | 0.93% | 16.93% |  | 7.07% | 0.60% | 13.53% |  | 2.26% | 0.00% | 4.69% |
|  |  | Poisonings | 0.00% | 0.00% | 0.00% |  | 0.10% | 0.00% | 2.62% |  | 1.07% | 0.00% | 19.70% |  | 0.03% | 0.00% | 2.79% |
|  |  | Falls | 0.00% | 0.00% | 0.00% |  | 0.10% | 0.00% | 2.62% |  | 1.07% | 0.00% | 19.70% |  | 0.03% | 0.00% | 2.79% |
|  |  | Fires, heat and hot substances | 0.00% | 0.00% | 0.00% |  | 0.10% | 0.00% | 2.62% |  | 1.07% | 0.00% | 19.70% |  | 0.03% | 0.00% | 2.79% |
|  |  | Drownings | 0.00% | 0.00% | 0.00% |  | 0.10% | 0.00% | 2.62% |  | 1.07% | 0.00% | 19.70% |  | 0.03% | 0.00% | 2.79% |
|  |  | Other unintentional injuries | 0.00% | 0.00% | 0.00% |  | 0.10% | 0.00% | 2.62% |  | 1.07% | 0.00% | 19.70% |  | 0.03% | 0.00% | 2.79% |
|  | Intentional injuries | |  |  |  |  |  |  |  |  |  |  |  |  |  |  |  |
|  |  | Self-inflicted injuries | 0.00% | 0.00% | 0.00% |  | 0.10% | 0.00% | 2.62% |  | 1.07% | 0.00% | 19.70% |  | 0.03% | 0.00% | 2.79% |
|  |  | Violence | 3.35% | 0.00% | 15.90% |  | 9.60% | 0.00% | 45.58% |  | 5.57% | 0.00% | 26.42% |  | 2.51% | 0.00% | 11.89% |
|  |  | Other intentional injuries | 0.00% | 0.00% | 0.00% |  | 0.10% | 0.00% | 2.62% |  | 1.07% | 0.00% | 19.70% |  | 0.03% | 0.00% | 2.79% |
| Men | |  |  |  |  |  |  |  |  |  |  |  |  |  |  |  |  |
| Injuries | | |  |  |  |  |  |  |  |  |  |  |  |  |  |  |  |
|  | Unintentional injuries | |  |  |  |  |  |  |  |  |  |  |  |  |  |  |  |
|  |  | Transport injuries | 4.45% | 4.35% | 4.56% |  | 12.74% | 10.13% | 15.35% |  | 17.38% | 8.45% | 26.31% |  | 5.97% | 1.90% | 10.05% |
|  |  | Poisonings | 0.00% | 0.00% | 0.00% |  | 5.89% | 0.87% | 10.90% |  | 15.69% | 2.27% | 29.11% |  | 7.52% | 0.02% | 15.03% |
|  |  | Falls | 0.00% | 0.00% | 0.00% |  | 5.89% | 0.87% | 10.90% |  | 15.69% | 2.27% | 29.11% |  | 7.52% | 0.02% | 15.03% |
|  |  | Fires, heat and hot substances | 0.00% | 0.00% | 0.00% |  | 5.89% | 0.87% | 10.90% |  | 15.69% | 2.27% | 29.11% |  | 7.52% | 0.02% | 15.03% |
|  |  | Drownings | 0.00% | 0.00% | 0.00% |  | 5.89% | 0.87% | 10.90% |  | 15.69% | 2.27% | 29.11% |  | 7.52% | 0.02% | 15.03% |
|  |  | Other unintentional injuries | 0.00% | 0.00% | 0.00% |  | 5.89% | 0.87% | 10.90% |  | 15.69% | 2.27% | 29.11% |  | 7.52% | 0.02% | 15.03% |
|  | Intentional injuries | |  |  |  |  |  |  |  |  |  |  |  |  |  |  |  |
|  |  | Self-inflicted injuries | 0.00% | 0.00% | 0.00% |  | 5.89% | 0.87% | 10.90% |  | 15.69% | 2.27% | 29.11% |  | 7.52% | 0.02% | 15.03% |
|  |  | Violence | 3.35% | 0.00% | 15.90% |  | 9.60% | 0.00% | 45.58% |  | 5.57% | 0.00% | 26.42% |  | 2.51% | 0.00% | 11.89% |
|  |  | Other intentional injuries | 0.00% | 0.00% | 0.00% |  | 5.89% | 0.87% | 10.90% |  | 15.69% | 2.27% | 29.11% |  | 7.52% | 0.02% | 15.03% |

Table 26: Mortality Alcohol-Attributable Fractions for injuries (with harms to others included): Asia Southeast

|  |  |  | 0 to 14 years of age | | |  | 15 to 34 years of age | | |  | 35 to 64 years of age | | |  | 65 years of age and older | | |
| --- | --- | --- | --- | --- | --- | --- | --- | --- | --- | --- | --- | --- | --- | --- | --- | --- | --- |
|  |  |  | Point estimate | Lower 95% CI | Upper 95% CI |  | Point estimate | Lower 95% CI | Upper 95% CI |  | Point estimate | Lower 95% CI | Upper 95% CI |  | Point estimate | Lower 95% CI | Upper 95% CI |
| Women | | |  |  |  |  |  |  |  |  |  |  |  |  |  |  |  |
| Injuries | | |  |  |  |  |  |  |  |  |  |  |  |  |  |  |  |
|  | Unintentional injuries | |  |  |  |  |  |  |  |  |  |  |  |  |  |  |  |
|  |  | Transport injuries | 3.54% | 0.00% | 7.77% |  | 7.23% | 0.20% | 15.17% |  | 5.95% | 0.00% | 13.02% |  | 1.73% | 0.01% | 3.93% |
|  |  | Poisonings | 0.00% | 0.00% | 0.00% |  | 0.45% | 0.00% | 1.79% |  | 1.11% | 0.00% | 5.01% |  | 0.05% | 0.00% | 0.18% |
|  |  | Falls | 0.00% | 0.00% | 0.00% |  | 0.45% | 0.00% | 1.79% |  | 1.11% | 0.00% | 5.01% |  | 0.05% | 0.00% | 0.18% |
|  |  | Fires, heat and hot substances | 0.00% | 0.00% | 0.00% |  | 0.45% | 0.00% | 1.79% |  | 1.11% | 0.00% | 5.01% |  | 0.05% | 0.00% | 0.18% |
|  |  | Drownings | 0.00% | 0.00% | 0.00% |  | 0.45% | 0.00% | 1.79% |  | 1.11% | 0.00% | 5.01% |  | 0.05% | 0.00% | 0.18% |
|  |  | Other unintentional injuries | 0.00% | 0.00% | 0.00% |  | 0.45% | 0.00% | 1.79% |  | 1.11% | 0.00% | 5.01% |  | 0.05% | 0.00% | 0.18% |
|  | Intentional injuries | |  |  |  |  |  |  |  |  |  |  |  |  |  |  |  |
|  |  | Self-inflicted injuries | 0.00% | 0.00% | 0.00% |  | 0.45% | 0.00% | 1.79% |  | 1.11% | 0.00% | 5.01% |  | 0.05% | 0.00% | 0.18% |
|  |  | Violence | 4.09% | 1.52% | 6.67% |  | 11.73% | 4.34% | 19.12% |  | 6.80% | 2.52% | 11.08% |  | 3.06% | 1.13% | 4.99% |
|  |  | Other intentional injuries | 0.00% | 0.00% | 0.00% |  | 0.45% | 0.00% | 1.79% |  | 1.11% | 0.00% | 5.01% |  | 0.05% | 0.00% | 0.18% |
| Men | |  |  |  |  |  |  |  |  |  |  |  |  |  |  |  |  |
| Injuries | | |  |  |  |  |  |  |  |  |  |  |  |  |  |  |  |
|  | Unintentional injuries | |  |  |  |  |  |  |  |  |  |  |  |  |  |  |  |
|  |  | Transport injuries | 3.54% | 3.44% | 3.63% |  | 10.38% | 8.55% | 12.20% |  | 14.94% | 4.38% | 29.41% |  | 3.14% | 1.60% | 4.69% |
|  |  | Poisonings | 0.00% | 0.00% | 0.00% |  | 5.26% | 1.58% | 8.93% |  | 21.78% | 5.74% | 37.82% |  | 6.69% | 1.39% | 11.98% |
|  |  | Falls | 0.00% | 0.00% | 0.00% |  | 5.26% | 1.58% | 8.93% |  | 21.78% | 5.74% | 37.82% |  | 6.69% | 1.39% | 11.98% |
|  |  | Fires, heat and hot substances | 0.00% | 0.00% | 0.00% |  | 5.26% | 1.58% | 8.93% |  | 21.78% | 5.74% | 37.82% |  | 6.69% | 1.39% | 11.98% |
|  |  | Drownings | 0.00% | 0.00% | 0.00% |  | 5.26% | 1.58% | 8.93% |  | 21.78% | 5.74% | 37.82% |  | 6.69% | 1.39% | 11.98% |
|  |  | Other unintentional injuries | 0.00% | 0.00% | 0.00% |  | 5.26% | 1.58% | 8.93% |  | 21.78% | 5.74% | 37.82% |  | 6.69% | 1.39% | 11.98% |
|  | Intentional injuries | |  |  |  |  |  |  |  |  |  |  |  |  |  |  |  |
|  |  | Self-inflicted injuries | 0.00% | 0.00% | 0.00% |  | 5.26% | 1.58% | 8.93% |  | 21.78% | 5.74% | 37.82% |  | 6.69% | 1.39% | 11.98% |
|  |  | Violence | 4.09% | 1.52% | 6.67% |  | 11.73% | 4.34% | 19.12% |  | 6.80% | 2.52% | 11.08% |  | 3.06% | 1.13% | 4.99% |
|  |  | Other intentional injuries | 0.00% | 0.00% | 0.00% |  | 5.26% | 1.58% | 8.93% |  | 21.78% | 5.74% | 37.82% |  | 6.69% | 1.39% | 11.98% |

Table 27: Mortality Alcohol-Attributable Fractions for injuries (with harms to others included): Australasia

|  |  |  | 0 to 14 years of age | | |  | 15 to 34 years of age | | |  | 35 to 64 years of age | | |  | 65 years of age and older | | |
| --- | --- | --- | --- | --- | --- | --- | --- | --- | --- | --- | --- | --- | --- | --- | --- | --- | --- |
|  |  |  | Point estimate | Lower 95% CI | Upper 95% CI |  | Point estimate | Lower 95% CI | Upper 95% CI |  | Point estimate | Lower 95% CI | Upper 95% CI |  | Point estimate | Lower 95% CI | Upper 95% CI |
| Women | | |  |  |  |  |  |  |  |  |  |  |  |  |  |  |  |
| Injuries | | |  |  |  |  |  |  |  |  |  |  |  |  |  |  |  |
|  | Unintentional injuries | |  |  |  |  |  |  |  |  |  |  |  |  |  |  |  |
|  |  | Transport injuries | 4.03% | 2.81% | 5.26% |  | 10.51% | 7.79% | 13.24% |  | 8.98% | 6.03% | 11.92% |  | 3.11% | 1.58% | 4.63% |
|  |  | Poisonings | 0.00% | 0.00% | 0.00% |  | 6.84% | 3.28% | 10.39% |  | 6.40% | 2.99% | 9.81% |  | 3.03% | 1.43% | 4.64% |
|  |  | Falls | 0.00% | 0.00% | 0.00% |  | 6.84% | 3.28% | 10.39% |  | 6.40% | 2.99% | 9.81% |  | 3.03% | 1.43% | 4.64% |
|  |  | Fires, heat and hot substances | 0.00% | 0.00% | 0.00% |  | 6.84% | 3.28% | 10.39% |  | 6.40% | 2.99% | 9.81% |  | 3.03% | 1.43% | 4.64% |
|  |  | Drownings | 0.00% | 0.00% | 0.00% |  | 6.84% | 3.28% | 10.39% |  | 6.40% | 2.99% | 9.81% |  | 3.03% | 1.43% | 4.64% |
|  |  | Other unintentional injuries | 0.00% | 0.00% | 0.00% |  | 6.84% | 3.28% | 10.39% |  | 6.40% | 2.99% | 9.81% |  | 3.03% | 1.43% | 4.64% |
|  | Intentional injuries | |  |  |  |  |  |  |  |  |  |  |  |  |  |  |  |
|  |  | Self-inflicted injuries | 0.00% | 0.00% | 0.00% |  | 6.84% | 3.28% | 10.39% |  | 6.40% | 2.99% | 9.81% |  | 3.03% | 1.43% | 4.64% |
|  |  | Violence | 8.93% | 6.13% | 11.73% |  | 25.59% | 17.56% | 33.62% |  | 14.83% | 10.18% | 19.49% |  | 6.68% | 4.58% | 8.77% |
|  |  | Other intentional injuries | 0.00% | 0.00% | 0.00% |  | 6.84% | 3.28% | 10.39% |  | 6.40% | 2.99% | 9.81% |  | 3.03% | 1.43% | 4.64% |
| Men | |  |  |  |  |  |  |  |  |  |  |  |  |  |  |  |  |
| Injuries | | |  |  |  |  |  |  |  |  |  |  |  |  |  |  |  |
|  | Unintentional injuries | |  |  |  |  |  |  |  |  |  |  |  |  |  |  |  |
|  |  | Transport injuries | 4.03% | 4.03% | 4.04% |  | 14.42% | 11.67% | 17.16% |  | 13.71% | 9.88% | 17.55% |  | 5.01% | 3.27% | 6.75% |
|  |  | Poisonings | 0.00% | 0.00% | 0.00% |  | 19.41% | 9.05% | 29.76% |  | 27.08% | 13.03% | 41.12% |  | 16.39% | 7.26% | 25.53% |
|  |  | Falls | 0.00% | 0.00% | 0.00% |  | 19.41% | 9.05% | 29.76% |  | 27.08% | 13.03% | 41.12% |  | 16.39% | 7.26% | 25.53% |
|  |  | Fires, heat and hot substances | 0.00% | 0.00% | 0.00% |  | 19.41% | 9.05% | 29.76% |  | 27.08% | 13.03% | 41.12% |  | 16.39% | 7.26% | 25.53% |
|  |  | Drownings | 0.00% | 0.00% | 0.00% |  | 19.41% | 9.05% | 29.76% |  | 27.08% | 13.03% | 41.12% |  | 16.39% | 7.26% | 25.53% |
|  |  | Other unintentional injuries | 0.00% | 0.00% | 0.00% |  | 19.41% | 9.05% | 29.76% |  | 27.08% | 13.03% | 41.12% |  | 16.39% | 7.26% | 25.53% |
|  | Intentional injuries | |  |  |  |  |  |  |  |  |  |  |  |  |  |  |  |
|  |  | Self-inflicted injuries | 0.00% | 0.00% | 0.00% |  | 19.41% | 9.05% | 29.76% |  | 27.08% | 13.03% | 41.12% |  | 16.39% | 7.26% | 25.53% |
|  |  | Violence | 8.93% | 6.13% | 11.73% |  | 25.59% | 17.56% | 33.62% |  | 14.83% | 10.18% | 19.49% |  | 6.68% | 4.58% | 8.77% |
|  |  | Other intentional injuries | 0.00% | 0.00% | 0.00% |  | 19.41% | 9.05% | 29.76% |  | 27.08% | 13.03% | 41.12% |  | 16.39% | 7.26% | 25.53% |

Table 28: Mortality Alcohol-Attributable Fractions for injuries (with harms to others included): Caribbean

|  |  |  | 0 to 14 years of age | | |  | 15 to 34 years of age | | |  | 35 to 64 years of age | | |  | 65 years of age and older | | |
| --- | --- | --- | --- | --- | --- | --- | --- | --- | --- | --- | --- | --- | --- | --- | --- | --- | --- |
|  |  |  | Point estimate | Lower 95% CI | Upper 95% CI |  | Point estimate | Lower 95% CI | Upper 95% CI |  | Point estimate | Lower 95% CI | Upper 95% CI |  | Point estimate | Lower 95% CI | Upper 95% CI |
| Women | | |  |  |  |  |  |  |  |  |  |  |  |  |  |  |  |
| Injuries | | |  |  |  |  |  |  |  |  |  |  |  |  |  |  |  |
|  | Unintentional injuries | |  |  |  |  |  |  |  |  |  |  |  |  |  |  |  |
|  |  | Transport injuries | 3.88% | 0.96% | 6.80% |  | 9.52% | 3.57% | 15.46% |  | 7.64% | 2.87% | 12.42% |  | 2.37% | 0.57% | 4.17% |
|  |  | Poisonings | 0.00% | 0.00% | 0.00% |  | 5.71% | 0.74% | 10.68% |  | 1.56% | 0.52% | 2.61% |  | 0.24% | 0.06% | 0.42% |
|  |  | Falls | 0.00% | 0.00% | 0.00% |  | 5.71% | 0.74% | 10.68% |  | 1.56% | 0.52% | 2.61% |  | 0.24% | 0.06% | 0.42% |
|  |  | Fires, heat and hot substances | 0.00% | 0.00% | 0.00% |  | 5.71% | 0.74% | 10.68% |  | 1.56% | 0.52% | 2.61% |  | 0.24% | 0.06% | 0.42% |
|  |  | Drownings | 0.00% | 0.00% | 0.00% |  | 5.71% | 0.74% | 10.68% |  | 1.56% | 0.52% | 2.61% |  | 0.24% | 0.06% | 0.42% |
|  |  | Other unintentional injuries | 0.00% | 0.00% | 0.00% |  | 5.71% | 0.74% | 10.68% |  | 1.56% | 0.52% | 2.61% |  | 0.24% | 0.06% | 0.42% |
|  | Intentional injuries | |  |  |  |  |  |  |  |  |  |  |  |  |  |  |  |
|  |  | Self-inflicted injuries | 0.00% | 0.00% | 0.00% |  | 5.71% | 0.74% | 10.68% |  | 1.56% | 0.52% | 2.61% |  | 0.24% | 0.06% | 0.42% |
|  |  | Violence | 5.42% | 3.45% | 7.39% |  | 15.54% | 9.88% | 21.19% |  | 9.00% | 5.73% | 12.28% |  | 4.05% | 2.58% | 5.53% |
|  |  | Other intentional injuries | 0.00% | 0.00% | 0.00% |  | 5.71% | 0.74% | 10.68% |  | 1.56% | 0.52% | 2.61% |  | 0.24% | 0.06% | 0.42% |
| Men | |  |  |  |  |  |  |  |  |  |  |  |  |  |  |  |  |
| Injuries | | |  |  |  |  |  |  |  |  |  |  |  |  |  |  |  |
|  | Unintentional injuries | |  |  |  |  |  |  |  |  |  |  |  |  |  |  |  |
|  |  | Transport injuries | 3.88% | 3.83% | 3.93% |  | 14.19% | 11.29% | 17.09% |  | 12.70% | 9.54% | 15.85% |  | 3.87% | 2.81% | 4.93% |
|  |  | Poisonings | 0.00% | 0.00% | 0.00% |  | 12.91% | 5.24% | 20.58% |  | 17.47% | 7.17% | 27.76% |  | 7.63% | 2.70% | 12.56% |
|  |  | Falls | 0.00% | 0.00% | 0.00% |  | 12.91% | 5.24% | 20.58% |  | 17.47% | 7.17% | 27.76% |  | 7.63% | 2.70% | 12.56% |
|  |  | Fires, heat and hot substances | 0.00% | 0.00% | 0.00% |  | 12.91% | 5.24% | 20.58% |  | 17.47% | 7.17% | 27.76% |  | 7.63% | 2.70% | 12.56% |
|  |  | Drownings | 0.00% | 0.00% | 0.00% |  | 12.91% | 5.24% | 20.58% |  | 17.47% | 7.17% | 27.76% |  | 7.63% | 2.70% | 12.56% |
|  |  | Other unintentional injuries | 0.00% | 0.00% | 0.00% |  | 12.91% | 5.24% | 20.58% |  | 17.47% | 7.17% | 27.76% |  | 7.63% | 2.70% | 12.56% |
|  | Intentional injuries | |  |  |  |  |  |  |  |  |  |  |  |  |  |  |  |
|  |  | Self-inflicted injuries | 0.00% | 0.00% | 0.00% |  | 12.91% | 5.24% | 20.58% |  | 17.47% | 7.17% | 27.76% |  | 7.63% | 2.70% | 12.56% |
|  |  | Violence | 5.42% | 3.45% | 7.39% |  | 15.54% | 9.88% | 21.19% |  | 9.00% | 5.73% | 12.28% |  | 4.05% | 2.58% | 5.53% |
|  |  | Other intentional injuries | 0.00% | 0.00% | 0.00% |  | 12.91% | 5.24% | 20.58% |  | 17.47% | 7.17% | 27.76% |  | 7.63% | 2.70% | 12.56% |

Table 29: Mortality Alcohol-Attributable Fractions for injuries (with harms to others included): Europe Central

|  |  |  | 0 to 14 years of age | | |  | 15 to 34 years of age | | |  | 35 to 64 years of age | | |  | 65 years of age and older | | |
| --- | --- | --- | --- | --- | --- | --- | --- | --- | --- | --- | --- | --- | --- | --- | --- | --- | --- |
|  |  |  | Point estimate | Lower 95% CI | Upper 95% CI |  | Point estimate | Lower 95% CI | Upper 95% CI |  | Point estimate | Lower 95% CI | Upper 95% CI |  | Point estimate | Lower 95% CI | Upper 95% CI |
| Women | | |  |  |  |  |  |  |  |  |  |  |  |  |  |  |  |
| Injuries | | |  |  |  |  |  |  |  |  |  |  |  |  |  |  |  |
|  | Unintentional injuries | |  |  |  |  |  |  |  |  |  |  |  |  |  |  |  |
|  |  | Transport injuries | 16.68% | 3.10% | 30.25% |  | 35.58% | 13.30% | 57.85% |  | 32.65% | 8.68% | 56.63% |  | 12.79% | 1.64% | 23.95% |
|  |  | Poisonings | 0.00% | 0.00% | 0.00% |  | 11.46% | 0.15% | 22.77% |  | 13.90% | 0.53% | 27.28% |  | 1.02% | 0.19% | 1.84% |
|  |  | Falls | 0.00% | 0.00% | 0.00% |  | 11.46% | 0.15% | 22.77% |  | 13.90% | 0.53% | 27.28% |  | 1.02% | 0.19% | 1.84% |
|  |  | Fires, heat and hot substances | 0.00% | 0.00% | 0.00% |  | 11.46% | 0.15% | 22.77% |  | 13.90% | 0.53% | 27.28% |  | 1.02% | 0.19% | 1.84% |
|  |  | Drownings | 0.00% | 0.00% | 0.00% |  | 11.46% | 0.15% | 22.77% |  | 13.90% | 0.53% | 27.28% |  | 1.02% | 0.19% | 1.84% |
|  |  | Other unintentional injuries | 0.00% | 0.00% | 0.00% |  | 11.46% | 0.15% | 22.77% |  | 13.90% | 0.53% | 27.28% |  | 1.02% | 0.19% | 1.84% |
|  | Intentional injuries | |  |  |  |  |  |  |  |  |  |  |  |  |  |  |  |
|  |  | Self-inflicted injuries | 0.00% | 0.00% | 0.00% |  | 11.46% | 0.15% | 22.77% |  | 13.90% | 0.53% | 27.28% |  | 1.02% | 0.19% | 1.84% |
|  |  | Violence | 20.50% | 12.22% | 28.78% |  | 58.76% | 35.02% | 82.49% |  | 34.05% | 20.29% | 47.81% |  | 15.33% | 9.13% | 21.52% |
|  |  | Other intentional injuries | 0.00% | 0.00% | 0.00% |  | 11.46% | 0.15% | 22.77% |  | 13.90% | 0.53% | 27.28% |  | 1.02% | 0.19% | 1.84% |
| Men | |  |  |  |  |  |  |  |  |  |  |  |  |  |  |  |  |
| Injuries | | |  |  |  |  |  |  |  |  |  |  |  |  |  |  |  |
|  | Unintentional injuries | |  |  |  |  |  |  |  |  |  |  |  |  |  |  |  |
|  |  | Transport injuries | 16.68% | 15.71% | 17.64% |  | 47.15% | 36.45% | 57.85% |  | 54.13% | 24.79% | 83.48% |  | 24.05% | 10.67% | 37.43% |
|  |  | Poisonings | 0.00% | 0.00% | 0.00% |  | 38.23% | 17.56% | 58.91% |  | 66.75% | 33.38% | 90.00% |  | 41.57% | 18.08% | 65.07% |
|  |  | Falls | 0.00% | 0.00% | 0.00% |  | 38.23% | 17.56% | 58.91% |  | 66.75% | 33.38% | 90.00% |  | 41.57% | 18.08% | 65.07% |
|  |  | Fires, heat and hot substances | 0.00% | 0.00% | 0.00% |  | 38.23% | 17.56% | 58.91% |  | 66.75% | 33.38% | 90.00% |  | 41.57% | 18.08% | 65.07% |
|  |  | Drownings | 0.00% | 0.00% | 0.00% |  | 38.23% | 17.56% | 58.91% |  | 66.75% | 33.38% | 90.00% |  | 41.57% | 18.08% | 65.07% |
|  |  | Other unintentional injuries | 0.00% | 0.00% | 0.00% |  | 38.23% | 17.56% | 58.91% |  | 66.75% | 33.38% | 90.00% |  | 41.57% | 18.08% | 65.07% |
|  | Intentional injuries | |  |  |  |  |  |  |  |  |  |  |  |  |  |  |  |
|  |  | Self-inflicted injuries | 0.00% | 0.00% | 0.00% |  | 38.23% | 17.56% | 58.91% |  | 66.75% | 33.38% | 90.00% |  | 41.57% | 18.08% | 65.07% |
|  |  | Violence | 20.50% | 12.22% | 28.78% |  | 58.76% | 35.02% | 82.49% |  | 34.05% | 20.29% | 47.81% |  | 15.33% | 9.13% | 21.52% |
|  |  | Other intentional injuries | 0.00% | 0.00% | 0.00% |  | 38.23% | 17.56% | 58.91% |  | 66.75% | 33.38% | 90.00% |  | 41.57% | 18.08% | 65.07% |

Table 30: Mortality Alcohol-Attributable Fractions for injuries (with harms to others included): Europe Eastern

|  |  |  | 0 to 14 years of age | | |  | 15 to 34 years of age | | |  | 35 to 64 years of age | | |  | 65 years of age and older | | |
| --- | --- | --- | --- | --- | --- | --- | --- | --- | --- | --- | --- | --- | --- | --- | --- | --- | --- |
|  |  |  | Point estimate | Lower 95% CI | Upper 95% CI |  | Point estimate | Lower 95% CI | Upper 95% CI |  | Point estimate | Lower 95% CI | Upper 95% CI |  | Point estimate | Lower 95% CI | Upper 95% CI |
| Women | | |  |  |  |  |  |  |  |  |  |  |  |  |  |  |  |
| Injuries | | |  |  |  |  |  |  |  |  |  |  |  |  |  |  |  |
|  | Unintentional injuries | |  |  |  |  |  |  |  |  |  |  |  |  |  |  |  |
|  |  | Transport injuries | 40.80% | 23.45% | 58.16% |  | 74.92% | 62.34% | 87.50% |  | 66.57% | 53.61% | 79.54% |  | 42.47% | 26.63% | 53.42% |
|  |  | Poisonings | 0.00% | 0.00% | 0.00% |  | 29.43% | 3.32% | 55.55% |  | 23.70% | 1.71% | 45.68% |  | 2.87% | 0.00% | 6.15% |
|  |  | Falls | 0.00% | 0.00% | 0.00% |  | 29.43% | 3.32% | 55.55% |  | 23.70% | 1.71% | 45.68% |  | 2.87% | 0.00% | 6.15% |
|  |  | Fires, heat and hot substances | 0.00% | 0.00% | 0.00% |  | 29.43% | 3.32% | 55.55% |  | 23.70% | 1.71% | 45.68% |  | 2.87% | 0.00% | 6.15% |
|  |  | Drownings | 0.00% | 0.00% | 0.00% |  | 29.43% | 3.32% | 55.55% |  | 23.70% | 1.71% | 45.68% |  | 2.87% | 0.00% | 6.15% |
|  |  | Other unintentional injuries | 0.00% | 0.00% | 0.00% |  | 29.43% | 3.32% | 55.55% |  | 23.70% | 1.71% | 45.68% |  | 2.87% | 0.00% | 6.15% |
|  | Intentional injuries | |  |  |  |  |  |  |  |  |  |  |  |  |  |  |  |
|  |  | Self-inflicted injuries | 0.00% | 0.00% | 0.00% |  | 29.43% | 3.32% | 55.55% |  | 23.70% | 1.71% | 45.68% |  | 2.87% | 0.00% | 6.15% |
|  |  | Violence | 30.69% | 20.11% | 41.28% |  | 87.98% | 57.63% | 90.00% |  | 50.99% | 33.40% | 68.57% |  | 22.95% | 15.03% | 30.86% |
|  |  | Other intentional injuries | 0.00% | 0.00% | 0.00% |  | 29.43% | 3.32% | 55.55% |  | 23.70% | 1.71% | 45.68% |  | 2.87% | 0.00% | 6.15% |
| Men | |  |  |  |  |  |  |  |  |  |  |  |  |  |  |  |  |
| Injuries | | |  |  |  |  |  |  |  |  |  |  |  |  |  |  |  |
|  | Unintentional injuries | |  |  |  |  |  |  |  |  |  |  |  |  |  |  |  |
|  |  | Transport injuries | 40.80% | 39.20% | 42.40% |  | 90.00% | 90.00% | 90.00% |  | 90.00% | 90.00% | 90.00% |  | 88.41% | 68.51% | 90.00% |
|  |  | Poisonings | 0.00% | 0.00% | 0.00% |  | 83.36% | 46.51% | 90.00% |  | 74.89% | 41.37% | 90.00% |  | 43.04% | 19.95% | 66.13% |
|  |  | Falls | 0.00% | 0.00% | 0.00% |  | 83.36% | 46.51% | 90.00% |  | 74.89% | 41.37% | 90.00% |  | 43.04% | 19.95% | 66.13% |
|  |  | Fires, heat and hot substances | 0.00% | 0.00% | 0.00% |  | 83.36% | 46.51% | 90.00% |  | 74.89% | 41.37% | 90.00% |  | 43.04% | 19.95% | 66.13% |
|  |  | Drownings | 0.00% | 0.00% | 0.00% |  | 83.36% | 46.51% | 90.00% |  | 74.89% | 41.37% | 90.00% |  | 43.04% | 19.95% | 66.13% |
|  |  | Other unintentional injuries | 0.00% | 0.00% | 0.00% |  | 83.36% | 46.51% | 90.00% |  | 74.89% | 41.37% | 90.00% |  | 43.04% | 19.95% | 66.13% |
|  | Intentional injuries | |  |  |  |  |  |  |  |  |  |  |  |  |  |  |  |
|  |  | Self-inflicted injuries | 0.00% | 0.00% | 0.00% |  | 83.36% | 46.51% | 90.00% |  | 74.89% | 41.37% | 90.00% |  | 43.04% | 19.95% | 66.13% |
|  |  | Violence | 30.69% | 20.11% | 41.28% |  | 87.98% | 57.63% | 90.00% |  | 50.99% | 33.40% | 68.57% |  | 22.95% | 15.03% | 30.86% |
|  |  | Other intentional injuries | 0.00% | 0.00% | 0.00% |  | 83.36% | 46.51% | 90.00% |  | 74.89% | 41.37% | 90.00% |  | 43.04% | 19.95% | 66.13% |

Table 31: Mortality Alcohol-Attributable Fractions for injuries (with harms to others included): Europe Western

|  |  |  | 0 to 14 years of age | | |  | 15 to 34 years of age | | |  | 35 to 64 years of age | | |  | 65 years of age and older | | |
| --- | --- | --- | --- | --- | --- | --- | --- | --- | --- | --- | --- | --- | --- | --- | --- | --- | --- |
|  |  |  | Point estimate | Lower 95% CI | Upper 95% CI |  | Point estimate | Lower 95% CI | Upper 95% CI |  | Point estimate | Lower 95% CI | Upper 95% CI |  | Point estimate | Lower 95% CI | Upper 95% CI |
| Women | | |  |  |  |  |  |  |  |  |  |  |  |  |  |  |  |
| Injuries | | |  |  |  |  |  |  |  |  |  |  |  |  |  |  |  |
|  | Unintentional injuries | |  |  |  |  |  |  |  |  |  |  |  |  |  |  |  |
|  |  | Transport injuries | 6.16% | 1.03% | 11.30% |  | 15.54% | 5.04% | 26.04% |  | 13.81% | 3.92% | 23.71% |  | 4.61% | 0.90% | 8.32% |
|  |  | Poisonings | 0.00% | 0.00% | 0.00% |  | 6.32% | 2.28% | 10.35% |  | 11.06% | 1.28% | 20.84% |  | 3.96% | 1.44% | 6.48% |
|  |  | Falls | 0.00% | 0.00% | 0.00% |  | 6.32% | 2.28% | 10.35% |  | 11.06% | 1.28% | 20.84% |  | 3.96% | 1.44% | 6.48% |
|  |  | Fires, heat and hot substances | 0.00% | 0.00% | 0.00% |  | 6.32% | 2.28% | 10.35% |  | 11.06% | 1.28% | 20.84% |  | 3.96% | 1.44% | 6.48% |
|  |  | Drownings | 0.00% | 0.00% | 0.00% |  | 6.32% | 2.28% | 10.35% |  | 11.06% | 1.28% | 20.84% |  | 3.96% | 1.44% | 6.48% |
|  |  | Other unintentional injuries | 0.00% | 0.00% | 0.00% |  | 6.32% | 2.28% | 10.35% |  | 11.06% | 1.28% | 20.84% |  | 3.96% | 1.44% | 6.48% |
|  | Intentional injuries | |  |  |  |  |  |  |  |  |  |  |  |  |  |  |  |
|  |  | Self-inflicted injuries | 0.00% | 0.00% | 0.00% |  | 6.32% | 2.28% | 10.35% |  | 11.06% | 1.28% | 20.84% |  | 3.96% | 1.44% | 6.48% |
|  |  | Violence | 12.64% | 8.12% | 17.17% |  | 36.24% | 23.28% | 49.20% |  | 21.00% | 13.49% | 28.51% |  | 9.45% | 6.07% | 12.83% |
|  |  | Other intentional injuries | 0.00% | 0.00% | 0.00% |  | 6.32% | 2.28% | 10.35% |  | 11.06% | 1.28% | 20.84% |  | 3.96% | 1.44% | 6.48% |
| Men | |  |  |  |  |  |  |  |  |  |  |  |  |  |  |  |  |
| Injuries | | |  |  |  |  |  |  |  |  |  |  |  |  |  |  |  |
|  | Unintentional injuries | |  |  |  |  |  |  |  |  |  |  |  |  |  |  |  |
|  |  | Transport injuries | 6.16% | 6.02% | 6.31% |  | 21.02% | 15.95% | 26.09% |  | 21.39% | 12.77% | 30.01% |  | 7.34% | 4.48% | 10.19% |
|  |  | Poisonings | 0.00% | 0.00% | 0.00% |  | 27.79% | 12.47% | 43.10% |  | 39.40% | 18.48% | 60.33% |  | 20.52% | 8.60% | 32.44% |
|  |  | Falls | 0.00% | 0.00% | 0.00% |  | 27.79% | 12.47% | 43.10% |  | 39.40% | 18.48% | 60.33% |  | 20.52% | 8.60% | 32.44% |
|  |  | Fires, heat and hot substances | 0.00% | 0.00% | 0.00% |  | 27.79% | 12.47% | 43.10% |  | 39.40% | 18.48% | 60.33% |  | 20.52% | 8.60% | 32.44% |
|  |  | Drownings | 0.00% | 0.00% | 0.00% |  | 27.79% | 12.47% | 43.10% |  | 39.40% | 18.48% | 60.33% |  | 20.52% | 8.60% | 32.44% |
|  |  | Other unintentional injuries | 0.00% | 0.00% | 0.00% |  | 27.79% | 12.47% | 43.10% |  | 39.40% | 18.48% | 60.33% |  | 20.52% | 8.60% | 32.44% |
|  | Intentional injuries | |  |  |  |  |  |  |  |  |  |  |  |  |  |  |  |
|  |  | Self-inflicted injuries | 0.00% | 0.00% | 0.00% |  | 27.79% | 12.47% | 43.10% |  | 39.40% | 18.48% | 60.33% |  | 20.52% | 8.60% | 32.44% |
|  |  | Violence | 12.64% | 8.12% | 17.17% |  | 36.24% | 23.28% | 49.20% |  | 21.00% | 13.49% | 28.51% |  | 9.45% | 6.07% | 12.83% |
|  |  | Other intentional injuries | 0.00% | 0.00% | 0.00% |  | 27.79% | 12.47% | 43.10% |  | 39.40% | 18.48% | 60.33% |  | 20.52% | 8.60% | 32.44% |

Table 32: Mortality Alcohol-Attributable Fractions for injuries (with harms to others included): Latin America Andean

|  |  |  | 0 to 14 years of age | | |  | 15 to 34 years of age | | |  | 35 to 64 years of age | | |  | 65 years of age and older | | |
| --- | --- | --- | --- | --- | --- | --- | --- | --- | --- | --- | --- | --- | --- | --- | --- | --- | --- |
|  |  |  | Point estimate | Lower 95% CI | Upper 95% CI |  | Point estimate | Lower 95% CI | Upper 95% CI |  | Point estimate | Lower 95% CI | Upper 95% CI |  | Point estimate | Lower 95% CI | Upper 95% CI |
| Women | | |  |  |  |  |  |  |  |  |  |  |  |  |  |  |  |
| Injuries | | |  |  |  |  |  |  |  |  |  |  |  |  |  |  |  |
|  | Unintentional injuries | |  |  |  |  |  |  |  |  |  |  |  |  |  |  |  |
|  |  | Transport injuries | 6.61% | 1.42% | 11.80% |  | 15.65% | 5.59% | 25.71% |  | 13.43% | 4.46% | 22.41% |  | 3.52% | 0.58% | 6.47% |
|  |  | Poisonings | 0.00% | 0.00% | 0.00% |  | 4.87% | 0.00% | 12.71% |  | 3.41% | 0.00% | 7.44% |  | 1.70% | 0.00% | 3.98% |
|  |  | Falls | 0.00% | 0.00% | 0.00% |  | 4.87% | 0.00% | 12.71% |  | 3.41% | 0.00% | 7.44% |  | 1.70% | 0.00% | 3.98% |
|  |  | Fires, heat and hot substances | 0.00% | 0.00% | 0.00% |  | 4.87% | 0.00% | 12.71% |  | 3.41% | 0.00% | 7.44% |  | 1.70% | 0.00% | 3.98% |
|  |  | Drownings | 0.00% | 0.00% | 0.00% |  | 4.87% | 0.00% | 12.71% |  | 3.41% | 0.00% | 7.44% |  | 1.70% | 0.00% | 3.98% |
|  |  | Other unintentional injuries | 0.00% | 0.00% | 0.00% |  | 4.87% | 0.00% | 12.71% |  | 3.41% | 0.00% | 7.44% |  | 1.70% | 0.00% | 3.98% |
|  | Intentional injuries | |  |  |  |  |  |  |  |  |  |  |  |  |  |  |  |
|  |  | Self-inflicted injuries | 0.00% | 0.00% | 0.00% |  | 4.87% | 0.00% | 12.71% |  | 3.41% | 0.00% | 7.44% |  | 1.70% | 0.00% | 3.98% |
|  |  | Violence | 7.67% | 3.84% | 11.49% |  | 21.97% | 11.02% | 32.93% |  | 12.73% | 6.38% | 19.08% |  | 5.73% | 2.87% | 8.59% |
|  |  | Other intentional injuries | 0.00% | 0.00% | 0.00% |  | 4.87% | 0.00% | 12.71% |  | 3.41% | 0.00% | 7.44% |  | 1.70% | 0.00% | 3.98% |
| Men | |  |  |  |  |  |  |  |  |  |  |  |  |  |  |  |  |
| Injuries | | |  |  |  |  |  |  |  |  |  |  |  |  |  |  |  |
|  | Unintentional injuries | |  |  |  |  |  |  |  |  |  |  |  |  |  |  |  |
|  |  | Transport injuries | 6.61% | 6.46% | 6.76% |  | 22.41% | 17.68% | 27.14% |  | 22.78% | 14.85% | 30.71% |  | 4.73% | 3.89% | 5.57% |
|  |  | Poisonings | 0.00% | 0.00% | 0.00% |  | 16.79% | 4.82% | 28.76% |  | 28.46% | 8.18% | 48.74% |  | 2.90% | 0.74% | 5.06% |
|  |  | Falls | 0.00% | 0.00% | 0.00% |  | 16.79% | 4.82% | 28.76% |  | 28.46% | 8.18% | 48.74% |  | 2.90% | 0.74% | 5.06% |
|  |  | Fires, heat and hot substances | 0.00% | 0.00% | 0.00% |  | 16.79% | 4.82% | 28.76% |  | 28.46% | 8.18% | 48.74% |  | 2.90% | 0.74% | 5.06% |
|  |  | Drownings | 0.00% | 0.00% | 0.00% |  | 16.79% | 4.82% | 28.76% |  | 28.46% | 8.18% | 48.74% |  | 2.90% | 0.74% | 5.06% |
|  |  | Other unintentional injuries | 0.00% | 0.00% | 0.00% |  | 16.79% | 4.82% | 28.76% |  | 28.46% | 8.18% | 48.74% |  | 2.90% | 0.74% | 5.06% |
|  | Intentional injuries | |  |  |  |  |  |  |  |  |  |  |  |  |  |  |  |
|  |  | Self-inflicted injuries | 0.00% | 0.00% | 0.00% |  | 16.79% | 4.82% | 28.76% |  | 28.46% | 8.18% | 48.74% |  | 2.90% | 0.74% | 5.06% |
|  |  | Violence | 7.67% | 3.84% | 11.49% |  | 21.97% | 11.02% | 32.93% |  | 12.73% | 6.38% | 19.08% |  | 5.73% | 2.87% | 8.59% |
|  |  | Other intentional injuries | 0.00% | 0.00% | 0.00% |  | 16.79% | 4.82% | 28.76% |  | 28.46% | 8.18% | 48.74% |  | 2.90% | 0.74% | 5.06% |

Table 33: Mortality Alcohol-Attributable Fractions for injuries (with harms to others included): Latin America Central

|  |  |  | 0 to 14 years of age | | |  | 15 to 34 years of age | | |  | 35 to 64 years of age | | |  | 65 years of age and older | | |
| --- | --- | --- | --- | --- | --- | --- | --- | --- | --- | --- | --- | --- | --- | --- | --- | --- | --- |
|  |  |  | Point estimate | Lower 95% CI | Upper 95% CI |  | Point estimate | Lower 95% CI | Upper 95% CI |  | Point estimate | Lower 95% CI | Upper 95% CI |  | Point estimate | Lower 95% CI | Upper 95% CI |
| Women | | |  |  |  |  |  |  |  |  |  |  |  |  |  |  |  |
| Injuries | | |  |  |  |  |  |  |  |  |  |  |  |  |  |  |  |
|  | Unintentional injuries | |  |  |  |  |  |  |  |  |  |  |  |  |  |  |  |
|  |  | Transport injuries | 18.65% | 6.85% | 30.44% |  | 39.64% | 20.59% | 58.69% |  | 33.88% | 17.11% | 50.65% |  | 14.07% | 5.21% | 22.93% |
|  |  | Poisonings | 0.00% | 0.00% | 0.00% |  | 5.07% | 0.00% | 12.05% |  | 3.27% | 0.00% | 7.58% |  | 0.90% | 0.05% | 1.76% |
|  |  | Falls | 0.00% | 0.00% | 0.00% |  | 5.07% | 0.00% | 12.05% |  | 3.27% | 0.00% | 7.58% |  | 0.90% | 0.05% | 1.76% |
|  |  | Fires, heat and hot substances | 0.00% | 0.00% | 0.00% |  | 5.07% | 0.00% | 12.05% |  | 3.27% | 0.00% | 7.58% |  | 0.90% | 0.05% | 1.76% |
|  |  | Drownings | 0.00% | 0.00% | 0.00% |  | 5.07% | 0.00% | 12.05% |  | 3.27% | 0.00% | 7.58% |  | 0.90% | 0.05% | 1.76% |
|  |  | Other unintentional injuries | 0.00% | 0.00% | 0.00% |  | 5.07% | 0.00% | 12.05% |  | 3.27% | 0.00% | 7.58% |  | 0.90% | 0.05% | 1.76% |
|  | Intentional injuries | |  |  |  |  |  |  |  |  |  |  |  |  |  |  |  |
|  |  | Self-inflicted injuries | 0.00% | 0.00% | 0.00% |  | 5.07% | 0.00% | 12.05% |  | 3.27% | 0.00% | 7.58% |  | 0.90% | 0.05% | 1.76% |
|  |  | Violence | 9.56% | 5.73% | 13.38% |  | 27.40% | 16.43% | 38.36% |  | 15.88% | 9.52% | 22.23% |  | 7.15% | 4.29% | 10.01% |
|  |  | Other intentional injuries | 0.00% | 0.00% | 0.00% |  | 5.07% | 0.00% | 12.05% |  | 3.27% | 0.00% | 7.58% |  | 0.90% | 0.05% | 1.76% |
| Men | |  |  |  |  |  |  |  |  |  |  |  |  |  |  |  |  |
| Injuries | | |  |  |  |  |  |  |  |  |  |  |  |  |  |  |  |
|  | Unintentional injuries | |  |  |  |  |  |  |  |  |  |  |  |  |  |  |  |
|  |  | Transport injuries | 18.65% | 17.90% | 19.39% |  | 55.33% | 42.72% | 67.95% |  | 55.34% | 41.32% | 69.36% |  | 28.00% | 19.18% | 36.82% |
|  |  | Poisonings | 0.00% | 0.00% | 0.00% |  | 23.72% | 9.65% | 37.79% |  | 31.75% | 13.55% | 49.96% |  | 19.22% | 6.90% | 31.55% |
|  |  | Falls | 0.00% | 0.00% | 0.00% |  | 23.72% | 9.65% | 37.79% |  | 31.75% | 13.55% | 49.96% |  | 19.22% | 6.90% | 31.55% |
|  |  | Fires, heat and hot substances | 0.00% | 0.00% | 0.00% |  | 23.72% | 9.65% | 37.79% |  | 31.75% | 13.55% | 49.96% |  | 19.22% | 6.90% | 31.55% |
|  |  | Drownings | 0.00% | 0.00% | 0.00% |  | 23.72% | 9.65% | 37.79% |  | 31.75% | 13.55% | 49.96% |  | 19.22% | 6.90% | 31.55% |
|  |  | Other unintentional injuries | 0.00% | 0.00% | 0.00% |  | 23.72% | 9.65% | 37.79% |  | 31.75% | 13.55% | 49.96% |  | 19.22% | 6.90% | 31.55% |
|  | Intentional injuries | |  |  |  |  |  |  |  |  |  |  |  |  |  |  |  |
|  |  | Self-inflicted injuries | 0.00% | 0.00% | 0.00% |  | 23.72% | 9.65% | 37.79% |  | 31.75% | 13.55% | 49.96% |  | 19.22% | 6.90% | 31.55% |
|  |  | Violence | 9.56% | 5.73% | 13.38% |  | 27.40% | 16.43% | 38.36% |  | 15.88% | 9.52% | 22.23% |  | 7.15% | 4.29% | 10.01% |
|  |  | Other intentional injuries | 0.00% | 0.00% | 0.00% |  | 23.72% | 9.65% | 37.79% |  | 31.75% | 13.55% | 49.96% |  | 19.22% | 6.90% | 31.55% |

Table 34: Mortality Alcohol-Attributable Fractions for injuries (with harms to others included): Latin America Southern

|  |  |  | 0 to 14 years of age | | |  | 15 to 34 years of age | | |  | 35 to 64 years of age | | |  | 65 years of age and older | | |
| --- | --- | --- | --- | --- | --- | --- | --- | --- | --- | --- | --- | --- | --- | --- | --- | --- | --- |
|  |  |  | Point estimate | Lower 95% CI | Upper 95% CI |  | Point estimate | Lower 95% CI | Upper 95% CI |  | Point estimate | Lower 95% CI | Upper 95% CI |  | Point estimate | Lower 95% CI | Upper 95% CI |
| Women | | |  |  |  |  |  |  |  |  |  |  |  |  |  |  |  |
| Injuries | | |  |  |  |  |  |  |  |  |  |  |  |  |  |  |  |
|  | Unintentional injuries | |  |  |  |  |  |  |  |  |  |  |  |  |  |  |  |
|  |  | Transport injuries | 5.08% | 1.14% | 9.01% |  | 13.54% | 5.21% | 21.87% |  | 10.02% | 3.38% | 16.66% |  | 3.76% | 0.99% | 6.54% |
|  |  | Poisonings | 0.00% | 0.00% | 0.00% |  | 4.28% | 1.12% | 7.45% |  | 7.74% | 0.00% | 16.42% |  | 2.87% | 0.72% | 5.03% |
|  |  | Falls | 0.00% | 0.00% | 0.00% |  | 4.28% | 1.12% | 7.45% |  | 7.74% | 0.00% | 16.42% |  | 2.87% | 0.72% | 5.03% |
|  |  | Fires, heat and hot substances | 0.00% | 0.00% | 0.00% |  | 4.28% | 1.12% | 7.45% |  | 7.74% | 0.00% | 16.42% |  | 2.87% | 0.72% | 5.03% |
|  |  | Drownings | 0.00% | 0.00% | 0.00% |  | 4.28% | 1.12% | 7.45% |  | 7.74% | 0.00% | 16.42% |  | 2.87% | 0.72% | 5.03% |
|  |  | Other unintentional injuries | 0.00% | 0.00% | 0.00% |  | 4.28% | 1.12% | 7.45% |  | 7.74% | 0.00% | 16.42% |  | 2.87% | 0.72% | 5.03% |
|  | Intentional injuries | |  |  |  |  |  |  |  |  |  |  |  |  |  |  |  |
|  |  | Self-inflicted injuries | 0.00% | 0.00% | 0.00% |  | 4.28% | 1.12% | 7.45% |  | 7.74% | 0.00% | 16.42% |  | 2.87% | 0.72% | 5.03% |
|  |  | Violence | 8.53% | 5.39% | 11.68% |  | 24.46% | 15.45% | 33.47% |  | 14.18% | 8.95% | 19.40% |  | 6.38% | 4.03% | 8.73% |
|  |  | Other intentional injuries | 0.00% | 0.00% | 0.00% |  | 4.28% | 1.12% | 7.45% |  | 7.74% | 0.00% | 16.42% |  | 2.87% | 0.72% | 5.03% |
| Men | |  |  |  |  |  |  |  |  |  |  |  |  |  |  |  |  |
| Injuries | | |  |  |  |  |  |  |  |  |  |  |  |  |  |  |  |
|  | Unintentional injuries | |  |  |  |  |  |  |  |  |  |  |  |  |  |  |  |
|  |  | Transport injuries | 5.08% | 4.99% | 5.16% |  | 19.59% | 15.08% | 24.09% |  | 14.58% | 10.54% | 18.61% |  | 6.16% | 4.09% | 8.23% |
|  |  | Poisonings | 0.00% | 0.00% | 0.00% |  | 22.98% | 9.38% | 36.57% |  | 23.33% | 9.25% | 37.41% |  | 14.69% | 5.27% | 24.10% |
|  |  | Falls | 0.00% | 0.00% | 0.00% |  | 22.98% | 9.38% | 36.57% |  | 23.33% | 9.25% | 37.41% |  | 14.69% | 5.27% | 24.10% |
|  |  | Fires, heat and hot substances | 0.00% | 0.00% | 0.00% |  | 22.98% | 9.38% | 36.57% |  | 23.33% | 9.25% | 37.41% |  | 14.69% | 5.27% | 24.10% |
|  |  | Drownings | 0.00% | 0.00% | 0.00% |  | 22.98% | 9.38% | 36.57% |  | 23.33% | 9.25% | 37.41% |  | 14.69% | 5.27% | 24.10% |
|  |  | Other unintentional injuries | 0.00% | 0.00% | 0.00% |  | 22.98% | 9.38% | 36.57% |  | 23.33% | 9.25% | 37.41% |  | 14.69% | 5.27% | 24.10% |
|  | Intentional injuries | |  |  |  |  |  |  |  |  |  |  |  |  |  |  |  |
|  |  | Self-inflicted injuries | 0.00% | 0.00% | 0.00% |  | 22.98% | 9.38% | 36.57% |  | 23.33% | 9.25% | 37.41% |  | 14.69% | 5.27% | 24.10% |
|  |  | Violence | 8.53% | 5.39% | 11.68% |  | 24.46% | 15.45% | 33.47% |  | 14.18% | 8.95% | 19.40% |  | 6.38% | 4.03% | 8.73% |
|  |  | Other intentional injuries | 0.00% | 0.00% | 0.00% |  | 22.98% | 9.38% | 36.57% |  | 23.33% | 9.25% | 37.41% |  | 14.69% | 5.27% | 24.10% |

Table 35: Mortality Alcohol-Attributable Fractions for injuries (with harms to others included): Latin America Tropical

|  |  |  | 0 to 14 years of age | | |  | 15 to 34 years of age | | |  | 35 to 64 years of age | | |  | 65 years of age and older | | |
| --- | --- | --- | --- | --- | --- | --- | --- | --- | --- | --- | --- | --- | --- | --- | --- | --- | --- |
|  |  |  | Point estimate | Lower 95% CI | Upper 95% CI |  | Point estimate | Lower 95% CI | Upper 95% CI |  | Point estimate | Lower 95% CI | Upper 95% CI |  | Point estimate | Lower 95% CI | Upper 95% CI |
| Women | | |  |  |  |  |  |  |  |  |  |  |  |  |  |  |  |
| Injuries | | |  |  |  |  |  |  |  |  |  |  |  |  |  |  |  |
|  | Unintentional injuries | |  |  |  |  |  |  |  |  |  |  |  |  |  |  |  |
|  |  | Transport injuries | 9.51% | 1.31% | 17.71% |  | 23.09% | 6.98% | 39.19% |  | 17.66% | 3.42% | 31.89% |  | 4.86% | 0.29% | 9.43% |
|  |  | Poisonings | 0.00% | 0.00% | 0.00% |  | 6.37% | 0.00% | 13.64% |  | 9.45% | 0.00% | 20.55% |  | 0.83% | 0.02% | 1.64% |
|  |  | Falls | 0.00% | 0.00% | 0.00% |  | 6.37% | 0.00% | 13.64% |  | 9.45% | 0.00% | 20.55% |  | 0.83% | 0.02% | 1.64% |
|  |  | Fires, heat and hot substances | 0.00% | 0.00% | 0.00% |  | 6.37% | 0.00% | 13.64% |  | 9.45% | 0.00% | 20.55% |  | 0.83% | 0.02% | 1.64% |
|  |  | Drownings | 0.00% | 0.00% | 0.00% |  | 6.37% | 0.00% | 13.64% |  | 9.45% | 0.00% | 20.55% |  | 0.83% | 0.02% | 1.64% |
|  |  | Other unintentional injuries | 0.00% | 0.00% | 0.00% |  | 6.37% | 0.00% | 13.64% |  | 9.45% | 0.00% | 20.55% |  | 0.83% | 0.02% | 1.64% |
|  | Intentional injuries | |  |  |  |  |  |  |  |  |  |  |  |  |  |  |  |
|  |  | Self-inflicted injuries | 0.00% | 0.00% | 0.00% |  | 6.37% | 0.00% | 13.64% |  | 9.45% | 0.00% | 20.55% |  | 0.83% | 0.02% | 1.64% |
|  |  | Violence | 12.42% | 7.05% | 17.79% |  | 35.60% | 20.20% | 50.99% |  | 20.63% | 11.71% | 29.55% |  | 9.29% | 5.27% | 13.30% |
|  |  | Other intentional injuries | 0.00% | 0.00% | 0.00% |  | 6.37% | 0.00% | 13.64% |  | 9.45% | 0.00% | 20.55% |  | 0.83% | 0.02% | 1.64% |
| Men | |  |  |  |  |  |  |  |  |  |  |  |  |  |  |  |  |
| Injuries | | |  |  |  |  |  |  |  |  |  |  |  |  |  |  |  |
|  | Unintentional injuries | |  |  |  |  |  |  |  |  |  |  |  |  |  |  |  |
|  |  | Transport injuries | 9.51% | 9.15% | 9.87% |  | 34.30% | 23.76% | 44.84% |  | 27.06% | 14.21% | 39.91% |  | 5.99% | 4.98% | 7.01% |
|  |  | Poisonings | 0.00% | 0.00% | 0.00% |  | 35.44% | 13.95% | 56.94% |  | 33.25% | 11.80% | 54.70% |  | 5.55% | 1.45% | 9.66% |
|  |  | Falls | 0.00% | 0.00% | 0.00% |  | 35.44% | 13.95% | 56.94% |  | 33.25% | 11.80% | 54.70% |  | 5.55% | 1.45% | 9.66% |
|  |  | Fires, heat and hot substances | 0.00% | 0.00% | 0.00% |  | 35.44% | 13.95% | 56.94% |  | 33.25% | 11.80% | 54.70% |  | 5.55% | 1.45% | 9.66% |
|  |  | Drownings | 0.00% | 0.00% | 0.00% |  | 35.44% | 13.95% | 56.94% |  | 33.25% | 11.80% | 54.70% |  | 5.55% | 1.45% | 9.66% |
|  |  | Other unintentional injuries | 0.00% | 0.00% | 0.00% |  | 35.44% | 13.95% | 56.94% |  | 33.25% | 11.80% | 54.70% |  | 5.55% | 1.45% | 9.66% |
|  | Intentional injuries | |  |  |  |  |  |  |  |  |  |  |  |  |  |  |  |
|  |  | Self-inflicted injuries | 0.00% | 0.00% | 0.00% |  | 35.44% | 13.95% | 56.94% |  | 33.25% | 11.80% | 54.70% |  | 5.55% | 1.45% | 9.66% |
|  |  | Violence | 12.42% | 7.05% | 17.79% |  | 35.60% | 20.20% | 50.99% |  | 20.63% | 11.71% | 29.55% |  | 9.29% | 5.27% | 13.30% |
|  |  | Other intentional injuries | 0.00% | 0.00% | 0.00% |  | 35.44% | 13.95% | 56.94% |  | 33.25% | 11.80% | 54.70% |  | 5.55% | 1.45% | 9.66% |

Table 36: Mortality Alcohol-Attributable Fractions for injuries (with harms to others included): North Africa Middle East

|  |  |  | 0 to 14 years of age | | |  | 15 to 34 years of age | | |  | 35 to 64 years of age | | |  | 65 years of age and older | | |
| --- | --- | --- | --- | --- | --- | --- | --- | --- | --- | --- | --- | --- | --- | --- | --- | --- | --- |
|  |  |  | Point estimate | Lower 95% CI | Upper 95% CI |  | Point estimate | Lower 95% CI | Upper 95% CI |  | Point estimate | Lower 95% CI | Upper 95% CI |  | Point estimate | Lower 95% CI | Upper 95% CI |
| Women | | |  |  |  |  |  |  |  |  |  |  |  |  |  |  |  |
| Injuries | | |  |  |  |  |  |  |  |  |  |  |  |  |  |  |  |
|  | Unintentional injuries | |  |  |  |  |  |  |  |  |  |  |  |  |  |  |  |
|  |  | Transport injuries | 1.34% | 0.00% | 4.51% |  | 3.15% | 0.00% | 11.52% |  | 2.02% | 0.00% | 6.72% |  | 0.61% | 0.00% | 2.10% |
|  |  | Poisonings | 0.00% | 0.00% | 0.00% |  | 0.49% | 0.00% | 3.27% |  | 0.29% | 0.00% | 4.26% |  | 0.04% | 0.00% | 1.27% |
|  |  | Falls | 0.00% | 0.00% | 0.00% |  | 0.49% | 0.00% | 3.27% |  | 0.29% | 0.00% | 4.26% |  | 0.04% | 0.00% | 1.27% |
|  |  | Fires, heat and hot substances | 0.00% | 0.00% | 0.00% |  | 0.49% | 0.00% | 3.27% |  | 0.29% | 0.00% | 4.26% |  | 0.04% | 0.00% | 1.27% |
|  |  | Drownings | 0.00% | 0.00% | 0.00% |  | 0.49% | 0.00% | 3.27% |  | 0.29% | 0.00% | 4.26% |  | 0.04% | 0.00% | 1.27% |
|  |  | Other unintentional injuries | 0.00% | 0.00% | 0.00% |  | 0.49% | 0.00% | 3.27% |  | 0.29% | 0.00% | 4.26% |  | 0.04% | 0.00% | 1.27% |
|  | Intentional injuries | |  |  |  |  |  |  |  |  |  |  |  |  |  |  |  |
|  |  | Self-inflicted injuries | 0.00% | 0.00% | 0.00% |  | 0.49% | 0.00% | 3.27% |  | 0.29% | 0.00% | 4.26% |  | 0.04% | 0.00% | 1.27% |
|  |  | Violence | 1.91% | 0.00% | 6.66% |  | 5.48% | 0.00% | 19.09% |  | 3.18% | 0.00% | 11.06% |  | 1.43% | 0.00% | 4.98% |
|  |  | Other intentional injuries | 0.00% | 0.00% | 0.00% |  | 0.49% | 0.00% | 3.27% |  | 0.29% | 0.00% | 4.26% |  | 0.04% | 0.00% | 1.27% |
| Men | |  |  |  |  |  |  |  |  |  |  |  |  |  |  |  |  |
| Injuries | | |  |  |  |  |  |  |  |  |  |  |  |  |  |  |  |
|  | Unintentional injuries | |  |  |  |  |  |  |  |  |  |  |  |  |  |  |  |
|  |  | Transport injuries | 1.34% | 1.28% | 1.39% |  | 6.58% | 2.36% | 24.95% |  | 2.72% | 1.80% | 4.45% |  | 0.68% | 0.60% | 0.76% |
|  |  | Poisonings | 0.00% | 0.00% | 0.00% |  | 7.91% | 0.16% | 15.66% |  | 3.26% | 0.00% | 6.99% |  | 0.08% | 0.01% | 0.15% |
|  |  | Falls | 0.00% | 0.00% | 0.00% |  | 7.91% | 0.16% | 15.66% |  | 3.26% | 0.00% | 6.99% |  | 0.08% | 0.01% | 0.15% |
|  |  | Fires, heat and hot substances | 0.00% | 0.00% | 0.00% |  | 7.91% | 0.16% | 15.66% |  | 3.26% | 0.00% | 6.99% |  | 0.08% | 0.01% | 0.15% |
|  |  | Drownings | 0.00% | 0.00% | 0.00% |  | 7.91% | 0.16% | 15.66% |  | 3.26% | 0.00% | 6.99% |  | 0.08% | 0.01% | 0.15% |
|  |  | Other unintentional injuries | 0.00% | 0.00% | 0.00% |  | 7.91% | 0.16% | 15.66% |  | 3.26% | 0.00% | 6.99% |  | 0.08% | 0.01% | 0.15% |
|  | Intentional injuries | |  |  |  |  |  |  |  |  |  |  |  |  |  |  |  |
|  |  | Self-inflicted injuries | 0.00% | 0.00% | 0.00% |  | 7.91% | 0.16% | 15.66% |  | 3.26% | 0.00% | 6.99% |  | 0.08% | 0.01% | 0.15% |
|  |  | Violence | 1.91% | 0.00% | 6.66% |  | 5.48% | 0.00% | 19.09% |  | 3.18% | 0.00% | 11.06% |  | 1.43% | 0.00% | 4.98% |
|  |  | Other intentional injuries | 0.00% | 0.00% | 0.00% |  | 7.91% | 0.16% | 15.66% |  | 3.26% | 0.00% | 6.99% |  | 0.08% | 0.01% | 0.15% |

Table 37: Mortality Alcohol-Attributable Fractions for injuries (with harms to others included): North America High Income

|  |  |  | 0 to 14 years of age | | |  | 15 to 34 years of age | | |  | 35 to 64 years of age | | |  | 65 years of age and older | | |
| --- | --- | --- | --- | --- | --- | --- | --- | --- | --- | --- | --- | --- | --- | --- | --- | --- | --- |
|  |  |  | Point estimate | Lower 95% CI | Upper 95% CI |  | Point estimate | Lower 95% CI | Upper 95% CI |  | Point estimate | Lower 95% CI | Upper 95% CI |  | Point estimate | Lower 95% CI | Upper 95% CI |
| Women | | |  |  |  |  |  |  |  |  |  |  |  |  |  |  |  |
| Injuries | | |  |  |  |  |  |  |  |  |  |  |  |  |  |  |  |
|  | Unintentional injuries | |  |  |  |  |  |  |  |  |  |  |  |  |  |  |  |
|  |  | Transport injuries | 1.34% | 0.00% | 4.51% |  | 3.15% | 0.00% | 11.52% |  | 2.02% | 0.00% | 6.72% |  | 0.61% | 0.00% | 2.10% |
|  |  | Poisonings | 0.00% | 0.00% | 0.00% |  | 0.49% | 0.00% | 3.27% |  | 0.29% | 0.00% | 4.26% |  | 0.04% | 0.00% | 1.27% |
|  |  | Falls | 0.00% | 0.00% | 0.00% |  | 0.49% | 0.00% | 3.27% |  | 0.29% | 0.00% | 4.26% |  | 0.04% | 0.00% | 1.27% |
|  |  | Fires, heat and hot substances | 0.00% | 0.00% | 0.00% |  | 0.49% | 0.00% | 3.27% |  | 0.29% | 0.00% | 4.26% |  | 0.04% | 0.00% | 1.27% |
|  |  | Drownings | 0.00% | 0.00% | 0.00% |  | 0.49% | 0.00% | 3.27% |  | 0.29% | 0.00% | 4.26% |  | 0.04% | 0.00% | 1.27% |
|  |  | Other unintentional injuries | 0.00% | 0.00% | 0.00% |  | 0.49% | 0.00% | 3.27% |  | 0.29% | 0.00% | 4.26% |  | 0.04% | 0.00% | 1.27% |
|  | Intentional injuries | |  |  |  |  |  |  |  |  |  |  |  |  |  |  |  |
|  |  | Self-inflicted injuries | 0.00% | 0.00% | 0.00% |  | 0.49% | 0.00% | 3.27% |  | 0.29% | 0.00% | 4.26% |  | 0.04% | 0.00% | 1.27% |
|  |  | Violence | 1.91% | 0.00% | 6.66% |  | 5.48% | 0.00% | 19.09% |  | 3.18% | 0.00% | 11.06% |  | 1.43% | 0.00% | 4.98% |
|  |  | Other intentional injuries | 0.00% | 0.00% | 0.00% |  | 0.49% | 0.00% | 3.27% |  | 0.29% | 0.00% | 4.26% |  | 0.04% | 0.00% | 1.27% |
| Men | |  |  |  |  |  |  |  |  |  |  |  |  |  |  |  |  |
| Injuries | | |  |  |  |  |  |  |  |  |  |  |  |  |  |  |  |
|  | Unintentional injuries | |  |  |  |  |  |  |  |  |  |  |  |  |  |  |  |
|  |  | Transport injuries | 1.34% | 1.28% | 1.39% |  | 6.58% | 2.36% | 24.95% |  | 2.72% | 1.80% | 4.45% |  | 0.68% | 0.60% | 0.76% |
|  |  | Poisonings | 0.00% | 0.00% | 0.00% |  | 7.91% | 0.16% | 15.66% |  | 3.26% | 0.00% | 6.99% |  | 0.08% | 0.01% | 0.15% |
|  |  | Falls | 0.00% | 0.00% | 0.00% |  | 7.91% | 0.16% | 15.66% |  | 3.26% | 0.00% | 6.99% |  | 0.08% | 0.01% | 0.15% |
|  |  | Fires, heat and hot substances | 0.00% | 0.00% | 0.00% |  | 7.91% | 0.16% | 15.66% |  | 3.26% | 0.00% | 6.99% |  | 0.08% | 0.01% | 0.15% |
|  |  | Drownings | 0.00% | 0.00% | 0.00% |  | 7.91% | 0.16% | 15.66% |  | 3.26% | 0.00% | 6.99% |  | 0.08% | 0.01% | 0.15% |
|  |  | Other unintentional injuries | 0.00% | 0.00% | 0.00% |  | 7.91% | 0.16% | 15.66% |  | 3.26% | 0.00% | 6.99% |  | 0.08% | 0.01% | 0.15% |
|  | Intentional injuries | |  |  |  |  |  |  |  |  |  |  |  |  |  |  |  |
|  |  | Self-inflicted injuries | 0.00% | 0.00% | 0.00% |  | 7.91% | 0.16% | 15.66% |  | 3.26% | 0.00% | 6.99% |  | 0.08% | 0.01% | 0.15% |
|  |  | Violence | 1.91% | 0.00% | 6.66% |  | 5.48% | 0.00% | 19.09% |  | 3.18% | 0.00% | 11.06% |  | 1.43% | 0.00% | 4.98% |
|  |  | Other intentional injuries | 0.00% | 0.00% | 0.00% |  | 7.91% | 0.16% | 15.66% |  | 3.26% | 0.00% | 6.99% |  | 0.08% | 0.01% | 0.15% |

Table 38: Mortality Alcohol-Attributable Fractions for injuries (with harms to others included): Oceania

|  |  |  | 0 to 14 years of age | | |  | 15 to 34 years of age | | |  | 35 to 64 years of age | | |  | 65 years of age and older | | |
| --- | --- | --- | --- | --- | --- | --- | --- | --- | --- | --- | --- | --- | --- | --- | --- | --- | --- |
|  |  |  | Point estimate | Lower 95% CI | Upper 95% CI |  | Point estimate | Lower 95% CI | Upper 95% CI |  | Point estimate | Lower 95% CI | Upper 95% CI |  | Point estimate | Lower 95% CI | Upper 95% CI |
| Women | | |  |  |  |  |  |  |  |  |  |  |  |  |  |  |  |
| Injuries | | |  |  |  |  |  |  |  |  |  |  |  |  |  |  |  |
|  | Unintentional injuries | |  |  |  |  |  |  |  |  |  |  |  |  |  |  |  |
|  |  | Transport injuries | 7.54% | 2.18% | 12.90% |  | 16.76% | 6.87% | 26.64% |  | 12.57% | 4.79% | 20.34% |  | 4.38% | 1.13% | 7.64% |
|  |  | Poisonings | 0.00% | 0.00% | 0.00% |  | 3.33% | 1.24% | 5.43% |  | 1.81% | 0.46% | 3.17% |  | 0.82% | 0.20% | 1.45% |
|  |  | Falls | 0.00% | 0.00% | 0.00% |  | 3.33% | 1.24% | 5.43% |  | 1.81% | 0.46% | 3.17% |  | 0.82% | 0.20% | 1.45% |
|  |  | Fires, heat and hot substances | 0.00% | 0.00% | 0.00% |  | 3.33% | 1.24% | 5.43% |  | 1.81% | 0.46% | 3.17% |  | 0.82% | 0.20% | 1.45% |
|  |  | Drownings | 0.00% | 0.00% | 0.00% |  | 3.33% | 1.24% | 5.43% |  | 1.81% | 0.46% | 3.17% |  | 0.82% | 0.20% | 1.45% |
|  |  | Other unintentional injuries | 0.00% | 0.00% | 0.00% |  | 3.33% | 1.24% | 5.43% |  | 1.81% | 0.46% | 3.17% |  | 0.82% | 0.20% | 1.45% |
|  | Intentional injuries | |  |  |  |  |  |  |  |  |  |  |  |  |  |  |  |
|  |  | Self-inflicted injuries | 0.00% | 0.00% | 0.00% |  | 3.33% | 1.24% | 5.43% |  | 1.81% | 0.46% | 3.17% |  | 0.82% | 0.20% | 1.45% |
|  |  | Violence | 4.13% | 2.77% | 5.48% |  | 11.83% | 7.95% | 15.70% |  | 6.85% | 4.61% | 9.10% |  | 3.08% | 2.07% | 4.10% |
|  |  | Other intentional injuries | 0.00% | 0.00% | 0.00% |  | 3.33% | 1.24% | 5.43% |  | 1.81% | 0.46% | 3.17% |  | 0.82% | 0.20% | 1.45% |
| Men | |  |  |  |  |  |  |  |  |  |  |  |  |  |  |  |  |
| Injuries | | |  |  |  |  |  |  |  |  |  |  |  |  |  |  |  |
|  | Unintentional injuries | |  |  |  |  |  |  |  |  |  |  |  |  |  |  |  |
|  |  | Transport injuries | 7.54% | 7.38% | 7.70% |  | 28.06% | 21.93% | 34.19% |  | 21.76% | 16.88% | 26.65% |  | 9.03% | 6.59% | 11.47% |
|  |  | Poisonings | 0.00% | 0.00% | 0.00% |  | 11.32% | 5.38% | 17.27% |  | 10.31% | 4.82% | 15.80% |  | 5.08% | 2.32% | 7.83% |
|  |  | Falls | 0.00% | 0.00% | 0.00% |  | 11.32% | 5.38% | 17.27% |  | 10.31% | 4.82% | 15.80% |  | 5.08% | 2.32% | 7.83% |
|  |  | Fires, heat and hot substances | 0.00% | 0.00% | 0.00% |  | 11.32% | 5.38% | 17.27% |  | 10.31% | 4.82% | 15.80% |  | 5.08% | 2.32% | 7.83% |
|  |  | Drownings | 0.00% | 0.00% | 0.00% |  | 11.32% | 5.38% | 17.27% |  | 10.31% | 4.82% | 15.80% |  | 5.08% | 2.32% | 7.83% |
|  |  | Other unintentional injuries | 0.00% | 0.00% | 0.00% |  | 11.32% | 5.38% | 17.27% |  | 10.31% | 4.82% | 15.80% |  | 5.08% | 2.32% | 7.83% |
|  | Intentional injuries | |  |  |  |  |  |  |  |  |  |  |  |  |  |  |  |
|  |  | Self-inflicted injuries | 0.00% | 0.00% | 0.00% |  | 11.32% | 5.38% | 17.27% |  | 10.31% | 4.82% | 15.80% |  | 5.08% | 2.32% | 7.83% |
|  |  | Violence | 4.13% | 2.77% | 5.48% |  | 11.83% | 7.95% | 15.70% |  | 6.85% | 4.61% | 9.10% |  | 3.08% | 2.07% | 4.10% |
|  |  | Other intentional injuries | 0.00% | 0.00% | 0.00% |  | 11.32% | 5.38% | 17.27% |  | 10.31% | 4.82% | 15.80% |  | 5.08% | 2.32% | 7.83% |

Table 39: Mortality Alcohol-Attributable Fractions for injuries (with harms to others included): Sub-Saharan Africa Central

|  |  |  | 0 to 14 years of age | | |  | 15 to 34 years of age | | |  | 35 to 64 years of age | | |  | 65 years of age and older | | |
| --- | --- | --- | --- | --- | --- | --- | --- | --- | --- | --- | --- | --- | --- | --- | --- | --- | --- |
|  |  |  | Point estimate | Lower 95% CI | Upper 95% CI |  | Point estimate | Lower 95% CI | Upper 95% CI |  | Point estimate | Lower 95% CI | Upper 95% CI |  | Point estimate | Lower 95% CI | Upper 95% CI |
| Women | | |  |  |  |  |  |  |  |  |  |  |  |  |  |  |  |
| Injuries | | |  |  |  |  |  |  |  |  |  |  |  |  |  |  |  |
|  | Unintentional injuries | |  |  |  |  |  |  |  |  |  |  |  |  |  |  |  |
|  |  | Transport injuries | 7.11% | 2.01% | 12.22% |  | 17.42% | 7.39% | 27.45% |  | 14.55% | 6.28% | 22.82% |  | 4.56% | 1.28% | 7.84% |
|  |  | Poisonings | 0.00% | 0.00% | 0.00% |  | 4.10% | 1.41% | 6.78% |  | 4.25% | 0.83% | 7.66% |  | 0.92% | 0.24% | 1.61% |
|  |  | Falls | 0.00% | 0.00% | 0.00% |  | 4.10% | 1.41% | 6.78% |  | 4.25% | 0.83% | 7.66% |  | 0.92% | 0.24% | 1.61% |
|  |  | Fires, heat and hot substances | 0.00% | 0.00% | 0.00% |  | 4.10% | 1.41% | 6.78% |  | 4.25% | 0.83% | 7.66% |  | 0.92% | 0.24% | 1.61% |
|  |  | Drownings | 0.00% | 0.00% | 0.00% |  | 4.10% | 1.41% | 6.78% |  | 4.25% | 0.83% | 7.66% |  | 0.92% | 0.24% | 1.61% |
|  |  | Other unintentional injuries | 0.00% | 0.00% | 0.00% |  | 4.10% | 1.41% | 6.78% |  | 4.25% | 0.83% | 7.66% |  | 0.92% | 0.24% | 1.61% |
|  | Intentional injuries | |  |  |  |  |  |  |  |  |  |  |  |  |  |  |  |
|  |  | Self-inflicted injuries | 0.00% | 0.00% | 0.00% |  | 4.10% | 1.41% | 6.78% |  | 4.25% | 0.83% | 7.66% |  | 0.92% | 0.24% | 1.61% |
|  |  | Violence | 4.44% | 2.96% | 5.91% |  | 12.71% | 8.50% | 16.93% |  | 7.37% | 4.92% | 9.81% |  | 3.32% | 2.22% | 4.42% |
|  |  | Other intentional injuries | 0.00% | 0.00% | 0.00% |  | 4.10% | 1.41% | 6.78% |  | 4.25% | 0.83% | 7.66% |  | 0.92% | 0.24% | 1.61% |
| Men | |  |  |  |  |  |  |  |  |  |  |  |  |  |  |  |  |
| Injuries | | |  |  |  |  |  |  |  |  |  |  |  |  |  |  |  |
|  | Unintentional injuries | |  |  |  |  |  |  |  |  |  |  |  |  |  |  |  |
|  |  | Transport injuries | 7.11% | 6.97% | 7.26% |  | 23.73% | 19.06% | 28.40% |  | 22.02% | 16.94% | 27.09% |  | 6.79% | 5.17% | 8.40% |
|  |  | Poisonings | 0.00% | 0.00% | 0.00% |  | 10.39% | 4.58% | 16.20% |  | 12.00% | 5.05% | 18.96% |  | 3.13% | 1.33% | 4.92% |
|  |  | Falls | 0.00% | 0.00% | 0.00% |  | 10.39% | 4.58% | 16.20% |  | 12.00% | 5.05% | 18.96% |  | 3.13% | 1.33% | 4.92% |
|  |  | Fires, heat and hot substances | 0.00% | 0.00% | 0.00% |  | 10.39% | 4.58% | 16.20% |  | 12.00% | 5.05% | 18.96% |  | 3.13% | 1.33% | 4.92% |
|  |  | Drownings | 0.00% | 0.00% | 0.00% |  | 10.39% | 4.58% | 16.20% |  | 12.00% | 5.05% | 18.96% |  | 3.13% | 1.33% | 4.92% |
|  |  | Other unintentional injuries | 0.00% | 0.00% | 0.00% |  | 10.39% | 4.58% | 16.20% |  | 12.00% | 5.05% | 18.96% |  | 3.13% | 1.33% | 4.92% |
|  | Intentional injuries | |  |  |  |  |  |  |  |  |  |  |  |  |  |  |  |
|  |  | Self-inflicted injuries | 0.00% | 0.00% | 0.00% |  | 10.39% | 4.58% | 16.20% |  | 12.00% | 5.05% | 18.96% |  | 3.13% | 1.33% | 4.92% |
|  |  | Violence | 4.44% | 2.96% | 5.91% |  | 12.71% | 8.50% | 16.93% |  | 7.37% | 4.92% | 9.81% |  | 3.32% | 2.22% | 4.42% |
|  |  | Other intentional injuries | 0.00% | 0.00% | 0.00% |  | 10.39% | 4.58% | 16.20% |  | 12.00% | 5.05% | 18.96% |  | 3.13% | 1.33% | 4.92% |

Table 40: Mortality Alcohol-Attributable Fractions for injuries (with harms to others included): Sub-Saharan Africa East

|  |  |  | 0 to 14 years of age | | |  | 15 to 34 years of age | | |  | 35 to 64 years of age | | |  | 65 years of age and older | | |
| --- | --- | --- | --- | --- | --- | --- | --- | --- | --- | --- | --- | --- | --- | --- | --- | --- | --- |
|  |  |  | Point estimate | Lower 95% CI | Upper 95% CI |  | Point estimate | Lower 95% CI | Upper 95% CI |  | Point estimate | Lower 95% CI | Upper 95% CI |  | Point estimate | Lower 95% CI | Upper 95% CI |
| Women | | |  |  |  |  |  |  |  |  |  |  |  |  |  |  |  |
| Injuries | | |  |  |  |  |  |  |  |  |  |  |  |  |  |  |  |
|  | Unintentional injuries | |  |  |  |  |  |  |  |  |  |  |  |  |  |  |  |
|  |  | Transport injuries | 6.46% | 0.00% | 13.64% |  | 13.91% | 0.71% | 27.52% |  | 14.30% | 0.00% | 29.44% |  | 4.32% | 0.59% | 8.69% |
|  |  | Poisonings | 0.00% | 0.00% | 0.00% |  | 2.00% | 0.00% | 5.33% |  | 6.40% | 0.00% | 16.01% |  | 1.83% | 0.00% | 4.62% |
|  |  | Falls | 0.00% | 0.00% | 0.00% |  | 2.00% | 0.00% | 5.33% |  | 6.40% | 0.00% | 16.01% |  | 1.83% | 0.00% | 4.62% |
|  |  | Fires, heat and hot substances | 0.00% | 0.00% | 0.00% |  | 2.00% | 0.00% | 5.33% |  | 6.40% | 0.00% | 16.01% |  | 1.83% | 0.00% | 4.62% |
|  |  | Drownings | 0.00% | 0.00% | 0.00% |  | 2.00% | 0.00% | 5.33% |  | 6.40% | 0.00% | 16.01% |  | 1.83% | 0.00% | 4.62% |
|  |  | Other unintentional injuries | 0.00% | 0.00% | 0.00% |  | 2.00% | 0.00% | 5.33% |  | 6.40% | 0.00% | 16.01% |  | 1.83% | 0.00% | 4.62% |
|  | Intentional injuries | |  |  |  |  |  |  |  |  |  |  |  |  |  |  |  |
|  |  | Self-inflicted injuries | 0.00% | 0.00% | 0.00% |  | 2.00% | 0.00% | 5.33% |  | 6.40% | 0.00% | 16.01% |  | 1.83% | 0.00% | 4.62% |
|  |  | Violence | 7.02% | 3.31% | 10.73% |  | 20.12% | 9.49% | 30.74% |  | 11.66% | 5.50% | 17.82% |  | 5.25% | 2.48% | 8.02% |
|  |  | Other intentional injuries | 0.00% | 0.00% | 0.00% |  | 2.00% | 0.00% | 5.33% |  | 6.40% | 0.00% | 16.01% |  | 1.83% | 0.00% | 4.62% |
| Men | |  |  |  |  |  |  |  |  |  |  |  |  |  |  |  |  |
| Injuries | | |  |  |  |  |  |  |  |  |  |  |  |  |  |  |  |
|  | Unintentional injuries | |  |  |  |  |  |  |  |  |  |  |  |  |  |  |  |
|  |  | Transport injuries | 6.46% | 6.18% | 6.73% |  | 17.39% | 13.34% | 21.45% |  | 26.40% | 7.08% | 46.60% |  | 7.62% | 4.71% | 10.52% |
|  |  | Poisonings | 0.00% | 0.00% | 0.00% |  | 11.43% | 2.93% | 19.92% |  | 33.49% | 9.93% | 57.05% |  | 12.20% | 2.96% | 21.43% |
|  |  | Falls | 0.00% | 0.00% | 0.00% |  | 11.43% | 2.93% | 19.92% |  | 33.49% | 9.93% | 57.05% |  | 12.20% | 2.96% | 21.43% |
|  |  | Fires, heat and hot substances | 0.00% | 0.00% | 0.00% |  | 11.43% | 2.93% | 19.92% |  | 33.49% | 9.93% | 57.05% |  | 12.20% | 2.96% | 21.43% |
|  |  | Drownings | 0.00% | 0.00% | 0.00% |  | 11.43% | 2.93% | 19.92% |  | 33.49% | 9.93% | 57.05% |  | 12.20% | 2.96% | 21.43% |
|  |  | Other unintentional injuries | 0.00% | 0.00% | 0.00% |  | 11.43% | 2.93% | 19.92% |  | 33.49% | 9.93% | 57.05% |  | 12.20% | 2.96% | 21.43% |
|  | Intentional injuries | |  |  |  |  |  |  |  |  |  |  |  |  |  |  |  |
|  |  | Self-inflicted injuries | 0.00% | 0.00% | 0.00% |  | 11.43% | 2.93% | 19.92% |  | 33.49% | 9.93% | 57.05% |  | 12.20% | 2.96% | 21.43% |
|  |  | Violence | 7.02% | 3.31% | 10.73% |  | 20.12% | 9.49% | 30.74% |  | 11.66% | 5.50% | 17.82% |  | 5.25% | 2.48% | 8.02% |
|  |  | Other intentional injuries | 0.00% | 0.00% | 0.00% |  | 11.43% | 2.93% | 19.92% |  | 33.49% | 9.93% | 57.05% |  | 12.20% | 2.96% | 21.43% |

Table 41: Mortality Alcohol-Attributable Fractions for injuries (with harms to others included): Sub-Saharan Africa Southern

|  |  |  | 0 to 14 years of age | | |  | 15 to 34 years of age | | |  | 35 to 64 years of age | | |  | 65 years of age and older | | |
| --- | --- | --- | --- | --- | --- | --- | --- | --- | --- | --- | --- | --- | --- | --- | --- | --- | --- |
|  |  |  | Point estimate | Lower 95% CI | Upper 95% CI |  | Point estimate | Lower 95% CI | Upper 95% CI |  | Point estimate | Lower 95% CI | Upper 95% CI |  | Point estimate | Lower 95% CI | Upper 95% CI |
| Women | | |  |  |  |  |  |  |  |  |  |  |  |  |  |  |  |
| Injuries | | |  |  |  |  |  |  |  |  |  |  |  |  |  |  |  |
|  | Unintentional injuries | |  |  |  |  |  |  |  |  |  |  |  |  |  |  |  |
|  |  | Transport injuries | 24.34% | 7.27% | 41.41% |  | 49.51% | 22.11% | 76.91% |  | 40.82% | 15.67% | 65.96% |  | 12.93% | 2.10% | 23.76% |
|  |  | Poisonings | 0.00% | 0.00% | 0.00% |  | 13.00% | 0.00% | 29.88% |  | 10.28% | 0.00% | 23.77% |  | 1.87% | 0.00% | 4.06% |
|  |  | Falls | 0.00% | 0.00% | 0.00% |  | 13.00% | 0.00% | 29.88% |  | 10.28% | 0.00% | 23.77% |  | 1.87% | 0.00% | 4.06% |
|  |  | Fires, heat and hot substances | 0.00% | 0.00% | 0.00% |  | 13.00% | 0.00% | 29.88% |  | 10.28% | 0.00% | 23.77% |  | 1.87% | 0.00% | 4.06% |
|  |  | Drownings | 0.00% | 0.00% | 0.00% |  | 13.00% | 0.00% | 29.88% |  | 10.28% | 0.00% | 23.77% |  | 1.87% | 0.00% | 4.06% |
|  |  | Other unintentional injuries | 0.00% | 0.00% | 0.00% |  | 13.00% | 0.00% | 29.88% |  | 10.28% | 0.00% | 23.77% |  | 1.87% | 0.00% | 4.06% |
|  | Intentional injuries | |  |  |  |  |  |  |  |  |  |  |  |  |  |  |  |
|  |  | Self-inflicted injuries | 0.00% | 0.00% | 0.00% |  | 13.00% | 0.00% | 29.88% |  | 10.28% | 0.00% | 23.77% |  | 1.87% | 0.00% | 4.06% |
|  |  | Violence | 19.41% | 10.59% | 28.22% |  | 55.62% | 30.35% | 80.89% |  | 32.23% | 17.59% | 46.88% |  | 14.51% | 7.92% | 21.10% |
|  |  | Other intentional injuries | 0.00% | 0.00% | 0.00% |  | 13.00% | 0.00% | 29.88% |  | 10.28% | 0.00% | 23.77% |  | 1.87% | 0.00% | 4.06% |
| Men | |  |  |  |  |  |  |  |  |  |  |  |  |  |  |  |  |
| Injuries | | |  |  |  |  |  |  |  |  |  |  |  |  |  |  |  |
|  | Unintentional injuries | |  |  |  |  |  |  |  |  |  |  |  |  |  |  |  |
|  |  | Transport injuries | 24.34% | 22.81% | 25.86% |  | 74.74% | 44.11% | 90.00% |  | 68.93% | 37.06% | 90.00% |  | 17.52% | 13.57% | 21.46% |
|  |  | Poisonings | 0.00% | 0.00% | 0.00% |  | 51.35% | 21.79% | 80.91% |  | 51.15% | 21.29% | 81.01% |  | 11.55% | 3.53% | 19.57% |
|  |  | Falls | 0.00% | 0.00% | 0.00% |  | 51.35% | 21.79% | 80.91% |  | 51.15% | 21.29% | 81.01% |  | 11.55% | 3.53% | 19.57% |
|  |  | Fires, heat and hot substances | 0.00% | 0.00% | 0.00% |  | 51.35% | 21.79% | 80.91% |  | 51.15% | 21.29% | 81.01% |  | 11.55% | 3.53% | 19.57% |
|  |  | Drownings | 0.00% | 0.00% | 0.00% |  | 51.35% | 21.79% | 80.91% |  | 51.15% | 21.29% | 81.01% |  | 11.55% | 3.53% | 19.57% |
|  |  | Other unintentional injuries | 0.00% | 0.00% | 0.00% |  | 51.35% | 21.79% | 80.91% |  | 51.15% | 21.29% | 81.01% |  | 11.55% | 3.53% | 19.57% |
|  | Intentional injuries | |  |  |  |  |  |  |  |  |  |  |  |  |  |  |  |
|  |  | Self-inflicted injuries | 0.00% | 0.00% | 0.00% |  | 51.35% | 21.79% | 80.91% |  | 51.15% | 21.29% | 81.01% |  | 11.55% | 3.53% | 19.57% |
|  |  | Violence | 19.41% | 10.59% | 28.22% |  | 55.62% | 30.35% | 80.89% |  | 32.23% | 17.59% | 46.88% |  | 14.51% | 7.92% | 21.10% |
|  |  | Other intentional injuries | 0.00% | 0.00% | 0.00% |  | 51.35% | 21.79% | 80.91% |  | 51.15% | 21.29% | 81.01% |  | 11.55% | 3.53% | 19.57% |

Table 42: Mortality Alcohol-Attributable Fractions for injuries (with harms to others included): Sub-Saharan Africa West

|  |  |  | 0 to 14 years of age | | |  | 15 to 34 years of age | | |  | 35 to 64 years of age | | |  | 65 years of age and older | | |
| --- | --- | --- | --- | --- | --- | --- | --- | --- | --- | --- | --- | --- | --- | --- | --- | --- | --- |
|  |  |  | Point estimate | Lower 95% CI | Upper 95% CI |  | Point estimate | Lower 95% CI | Upper 95% CI |  | Point estimate | Lower 95% CI | Upper 95% CI |  | Point estimate | Lower 95% CI | Upper 95% CI |
| Women | | |  |  |  |  |  |  |  |  |  |  |  |  |  |  |  |
| Injuries | | |  |  |  |  |  |  |  |  |  |  |  |  |  |  |  |
|  | Unintentional injuries | |  |  |  |  |  |  |  |  |  |  |  |  |  |  |  |
|  |  | Transport injuries | 6.51% | 0.00% | 13.32% |  | 14.42% | 1.21% | 27.63% |  | 14.42% | 0.51% | 28.35% |  | 4.30% | 0.88% | 8.18% |
|  |  | Poisonings | 0.00% | 0.00% | 0.00% |  | 4.41% | 0.09% | 8.72% |  | 13.71% | 1.39% | 26.04% |  | 3.54% | 0.85% | 6.23% |
|  |  | Falls | 0.00% | 0.00% | 0.00% |  | 4.41% | 0.09% | 8.72% |  | 13.71% | 1.39% | 26.04% |  | 3.54% | 0.85% | 6.23% |
|  |  | Fires, heat and hot substances | 0.00% | 0.00% | 0.00% |  | 4.41% | 0.09% | 8.72% |  | 13.71% | 1.39% | 26.04% |  | 3.54% | 0.85% | 6.23% |
|  |  | Drownings | 0.00% | 0.00% | 0.00% |  | 4.41% | 0.09% | 8.72% |  | 13.71% | 1.39% | 26.04% |  | 3.54% | 0.85% | 6.23% |
|  |  | Other unintentional injuries | 0.00% | 0.00% | 0.00% |  | 4.41% | 0.09% | 8.72% |  | 13.71% | 1.39% | 26.04% |  | 3.54% | 0.85% | 6.23% |
|  | Intentional injuries | |  |  |  |  |  |  |  |  |  |  |  |  |  |  |  |
|  |  | Self-inflicted injuries | 0.00% | 0.00% | 0.00% |  | 4.41% | 0.09% | 8.72% |  | 13.71% | 1.39% | 26.04% |  | 3.54% | 0.85% | 6.23% |
|  |  | Violence | 9.83% | 5.74% | 13.92% |  | 28.17% | 16.44% | 39.89% |  | 16.32% | 9.53% | 23.12% |  | 7.35% | 4.29% | 10.41% |
|  |  | Other intentional injuries | 0.00% | 0.00% | 0.00% |  | 4.41% | 0.09% | 8.72% |  | 13.71% | 1.39% | 26.04% |  | 3.54% | 0.85% | 6.23% |
| Men | |  |  |  |  |  |  |  |  |  |  |  |  |  |  |  |  |
| Injuries | | |  |  |  |  |  |  |  |  |  |  |  |  |  |  |  |
|  | Unintentional injuries | |  |  |  |  |  |  |  |  |  |  |  |  |  |  |  |
|  |  | Transport injuries | 6.51% | 6.26% | 6.76% |  | 18.13% | 13.36% | 22.89% |  | 24.58% | 8.87% | 40.30% |  | 6.94% | 5.38% | 8.50% |
|  |  | Poisonings | 0.00% | 0.00% | 0.00% |  | 17.33% | 6.01% | 28.64% |  | 36.97% | 14.12% | 59.82% |  | 9.39% | 3.41% | 15.37% |
|  |  | Falls | 0.00% | 0.00% | 0.00% |  | 17.33% | 6.01% | 28.64% |  | 36.97% | 14.12% | 59.82% |  | 9.39% | 3.41% | 15.37% |
|  |  | Fires, heat and hot substances | 0.00% | 0.00% | 0.00% |  | 17.33% | 6.01% | 28.64% |  | 36.97% | 14.12% | 59.82% |  | 9.39% | 3.41% | 15.37% |
|  |  | Drownings | 0.00% | 0.00% | 0.00% |  | 17.33% | 6.01% | 28.64% |  | 36.97% | 14.12% | 59.82% |  | 9.39% | 3.41% | 15.37% |
|  |  | Other unintentional injuries | 0.00% | 0.00% | 0.00% |  | 17.33% | 6.01% | 28.64% |  | 36.97% | 14.12% | 59.82% |  | 9.39% | 3.41% | 15.37% |
|  | Intentional injuries | |  |  |  |  |  |  |  |  |  |  |  |  |  |  |  |
|  |  | Self-inflicted injuries | 0.00% | 0.00% | 0.00% |  | 17.33% | 6.01% | 28.64% |  | 36.97% | 14.12% | 59.82% |  | 9.39% | 3.41% | 15.37% |
|  |  | Violence | 9.83% | 5.74% | 13.92% |  | 28.17% | 16.44% | 39.89% |  | 16.32% | 9.53% | 23.12% |  | 7.35% | 4.29% | 10.41% |
|  |  | Other intentional injuries | 0.00% | 0.00% | 0.00% |  | 17.33% | 6.01% | 28.64% |  | 36.97% | 14.12% | 59.82% |  | 9.39% | 3.41% | 15.37% |
