# Supplementary material for: Global burden of injuries attributable to alcohol consumption in 2004: a novel way of calculating the burden of injuries attributable to alcohol consumption
Source: Popul Health Metr. 2012 May 18;10:9. doi: 10.1186/1478-7954-10-9 (PMC3463441; doi:10.1186/1478-7954-10-9)
Supplement: Additional file 3 — Alcohol-Attributable Fractions for injuries. [file 1478-7954-10-9-S3.docx]

Appendix 3: Deaths from injuries attributable to alcohol consumption (without harms to others)

Table 1: Deaths from injuries attributable to alcohol consumption (without harms to others): Asia Pacific [high income]

|  |  |  | 0 to 14 years of age | | |  | 15 to 34 years of age | | |  | 35 to 64 years of age | | |  | 65 years of age and older | | |
| --- | --- | --- | --- | --- | --- | --- | --- | --- | --- | --- | --- | --- | --- | --- | --- | --- | --- |
|  |  |  | Point estimate | Lower 95% CI | Upper 95% CI |  | Point estimate | Lower 95% CI | Upper 95% CI |  | Point estimate | Lower 95% CI | Upper 95% CI |  | Point estimate | Lower 95% CI | Upper 95% CI |
| Women | |  |  |  |  |  |  |  |  |  |  |  |  |  |  |  |  |
| Injuries | |  | 0 | 0 | 0 |  | 152 | 69 | 235 |  | 592 | 240 | 945 |  | 361 | 162 | 560 |
|  | Unintentional injuries | | 0 | 0 | 0 |  | 37 | 19 | 55 |  | 221 | 96 | 346 |  | 275 | 125 | 425 |
|  |  | Transport injuries | 0 | 0 | 0 |  | 16 | 10 | 22 |  | 83 | 43 | 123 |  | 36 | 21 | 51 |
|  |  | Poisonings | 0 | 0 | 0 |  | 3 | 2 | 5 |  | 10 | 4 | 16 |  | 2 | 1 | 3 |
|  |  | Falls | 0 | 0 | 0 |  | 5 | 2 | 7 |  | 27 | 11 | 44 |  | 67 | 29 | 106 |
|  |  | Fires, heat and hot substances | 0 | 0 | 0 |  | 3 | 1 | 4 |  | 11 | 4 | 18 |  | 8 | 3 | 12 |
|  |  | Drownings | 0 | 0 | 0 |  | 4 | 2 | 7 |  | 29 | 11 | 47 |  | 40 | 17 | 62 |
|  |  | Other unintentional injuries | 0 | 0 | 0 |  | 6 | 3 | 10 |  | 61 | 24 | 98 |  | 122 | 53 | 191 |
|  | Intentional injuries | | 0 | 0 | 0 |  | 115 | 50 | 179 |  | 371 | 143 | 599 |  | 86 | 37 | 134 |
|  |  | Self-inflicted injuries | 0 | 0 | 0 |  | 115 | 50 | 179 |  | 371 | 143 | 599 |  | 86 | 37 | 134 |
|  |  | Violence | 0 | 0 | 0 |  | 0 | 0 | 0 |  | 0 | 0 | 0 |  | 0 | 0 | 0 |
|  |  | Other intentional injuries | 0 | 0 | 0 |  | 0 | 0 | 0 |  | 0 | 0 | 0 |  | 0 | 0 | 0 |
| Men | |  |  |  |  |  |  |  |  |  |  |  |  |  |  |  |  |
| Injuries | |  | 0 | 0 | 0 |  | 1583 | 702 | 2463 |  | 11599 | 5565 | 17633 |  | 2885 | 1190 | 4580 |
|  | Unintentional injuries | | 0 | 0 | 0 |  | 556 | 273 | 839 |  | 4301 | 2088 | 6513 |  | 1939 | 810 | 3068 |
|  |  | Transport injuries | 0 | 0 | 0 |  | 213 | 129 | 296 |  | 917 | 477 | 1357 |  | 167 | 99 | 236 |
|  |  | Poisonings | 0 | 0 | 0 |  | 28 | 12 | 44 |  | 162 | 77 | 247 |  | 19 | 8 | 31 |
|  |  | Falls | 0 | 0 | 0 |  | 80 | 33 | 126 |  | 941 | 448 | 1433 |  | 442 | 177 | 707 |
|  |  | Fires, heat and hot substances | 0 | 0 | 0 |  | 14 | 6 | 22 |  | 181 | 86 | 275 |  | 69 | 28 | 110 |
|  |  | Drownings | 0 | 0 | 0 |  | 82 | 34 | 130 |  | 489 | 233 | 745 |  | 254 | 102 | 405 |
|  |  | Other unintentional injuries | 0 | 0 | 0 |  | 139 | 58 | 220 |  | 1611 | 768 | 2455 |  | 988 | 397 | 1579 |
|  | Intentional injuries | | 0 | 0 | 0 |  | 1027 | 430 | 1625 |  | 7298 | 3476 | 11120 |  | 946 | 380 | 1512 |
|  |  | Self-inflicted injuries | 0 | 0 | 0 |  | 1025 | 429 | 1621 |  | 7291 | 3473 | 11109 |  | 945 | 379 | 1510 |
|  |  | Violence | 0 | 0 | 0 |  | 0 | 0 | 0 |  | 0 | 0 | 0 |  | 0 | 0 | 0 |
|  |  | Other intentional injuries | 0 | 0 | 0 |  | 2 | 1 | 3 |  | 7 | 4 | 11 |  | 1 | 0 | 2 |

Table 2: Deaths from injuries attributable to alcohol consumption (without harms to others): Asia Central

|  |  |  | 0 to 14 years of age | | |  | 15 to 34 years of age | | |  | 35 to 64 years of age | | |  | 65 years of age and older | | |
| --- | --- | --- | --- | --- | --- | --- | --- | --- | --- | --- | --- | --- | --- | --- | --- | --- | --- |
|  |  |  | Point estimate | Lower 95% CI | Upper 95% CI |  | Point estimate | Lower 95% CI | Upper 95% CI |  | Point estimate | Lower 95% CI | Upper 95% CI |  | Point estimate | Lower 95% CI | Upper 95% CI |
| Women | |  |  |  |  |  |  |  |  |  |  |  |  |  |  |  |  |
| Injuries | |  | 0 | 0 | 0 |  | 109 | 34 | 184 |  | 304 | 87 | 520 |  | 28 | 10 | 47 |
|  | Unintentional injuries | | 0 | 0 | 0 |  | 86 | 32 | 141 |  | 260 | 81 | 439 |  | 26 | 10 | 43 |
|  |  | Transport injuries | 0 | 0 | 0 |  | 40 | 26 | 54 |  | 81 | 56 | 107 |  | 16 | 10 | 21 |
|  |  | Poisonings | 0 | 0 | 0 |  | 9 | 1 | 17 |  | 44 | 6 | 81 |  | 2 | 0 | 5 |
|  |  | Falls | 0 | 0 | 0 |  | 2 | 0 | 5 |  | 10 | 1 | 19 |  | 1 | 0 | 1 |
|  |  | Fires, heat and hot substances | 0 | 0 | 0 |  | 8 | 1 | 16 |  | 17 | 2 | 32 |  | 1 | 0 | 3 |
|  |  | Drownings | 0 | 0 | 0 |  | 7 | 1 | 12 |  | 13 | 2 | 24 |  | 0 | 0 | 1 |
|  |  | Other unintentional injuries | 0 | 0 | 0 |  | 20 | 2 | 38 |  | 95 | 13 | 176 |  | 6 | 0 | 12 |
|  | Intentional injuries | | 0 | 0 | 0 |  | 23 | 3 | 43 |  | 44 | 6 | 81 |  | 2 | 0 | 4 |
|  |  | Self-inflicted injuries | 0 | 0 | 0 |  | 23 | 3 | 43 |  | 43 | 6 | 80 |  | 2 | 0 | 4 |
|  |  | Violence | 0 | 0 | 0 |  | 0 | 0 | 0 |  | 0 | 0 | 0 |  | 0 | 0 | 0 |
|  |  | Other intentional injuries | 0 | 0 | 0 |  | 0 | 0 | 0 |  | 0 | 0 | 1 |  | 0 | 0 | 0 |
| Men | |  |  |  |  |  |  |  |  |  |  |  |  |  |  |  |  |
| Injuries | |  | 0 | 0 | 0 |  | 2296 | 1064 | 3527 |  | 4156 | 2151 | 6161 |  | 372 | 179 | 564 |
|  | Unintentional injuries | | 0 | 0 | 0 |  | 1721 | 834 | 2608 |  | 3357 | 1781 | 4934 |  | 307 | 151 | 463 |
|  |  | Transport injuries | 0 | 0 | 0 |  | 562 | 370 | 755 |  | 1002 | 688 | 1316 |  | 87 | 56 | 119 |
|  |  | Poisonings | 0 | 0 | 0 |  | 200 | 80 | 320 |  | 517 | 240 | 794 |  | 40 | 17 | 62 |
|  |  | Falls | 0 | 0 | 0 |  | 78 | 31 | 125 |  | 153 | 71 | 234 |  | 18 | 8 | 28 |
|  |  | Fires, heat and hot substances | 0 | 0 | 0 |  | 61 | 25 | 98 |  | 132 | 61 | 203 |  | 17 | 7 | 26 |
|  |  | Drownings | 0 | 0 | 0 |  | 183 | 73 | 292 |  | 245 | 114 | 377 |  | 16 | 7 | 25 |
|  |  | Other unintentional injuries | 0 | 0 | 0 |  | 637 | 255 | 1018 |  | 1308 | 607 | 2009 |  | 130 | 56 | 204 |
|  | Intentional injuries | | 0 | 0 | 0 |  | 575 | 230 | 919 |  | 799 | 370 | 1227 |  | 64 | 28 | 101 |
|  |  | Self-inflicted injuries | 0 | 0 | 0 |  | 565 | 226 | 904 |  | 778 | 361 | 1195 |  | 64 | 28 | 100 |
|  |  | Violence | 0 | 0 | 0 |  | 0 | 0 | 0 |  | 0 | 0 | 0 |  | 0 | 0 | 0 |
|  |  | Other intentional injuries | 0 | 0 | 0 |  | 9 | 4 | 15 |  | 21 | 10 | 32 |  | 0 | 0 | 1 |

Table 3: Deaths from injuries attributable to alcohol consumption (without harms to others): Asia East

|  |  |  | 0 to 14 years of age | | |  | 15 to 34 years of age | | |  | 35 to 64 years of age | | |  | 65 years of age and older | | |
| --- | --- | --- | --- | --- | --- | --- | --- | --- | --- | --- | --- | --- | --- | --- | --- | --- | --- |
|  |  |  | Point estimate | Lower 95% CI | Upper 95% CI |  | Point estimate | Lower 95% CI | Upper 95% CI |  | Point estimate | Lower 95% CI | Upper 95% CI |  | Point estimate | Lower 95% CI | Upper 95% CI |
| Women | |  |  |  |  |  |  |  |  |  |  |  |  |  |  |  |  |
| Injuries | |  | 0 | 0 | 0 |  | 450 | 121 | 778 |  | 4043 | 375 | 8625 |  | 1012 | 48 | 2009 |
|  | Unintentional injuries | | 0 | 0 | 0 |  | 285 | 96 | 473 |  | 2245 | 375 | 4516 |  | 619 | 48 | 1208 |
|  |  | Transport injuries | 0 | 0 | 0 |  | 125 | 72 | 178 |  | 832 | 375 | 1289 |  | 99 | 48 | 150 |
|  |  | Poisonings | 0 | 0 | 0 |  | 39 | 6 | 72 |  | 268 | 0 | 613 |  | 42 | 0 | 85 |
|  |  | Falls | 0 | 0 | 0 |  | 33 | 5 | 62 |  | 349 | 0 | 797 |  | 254 | 0 | 516 |
|  |  | Fires, heat and hot substances | 0 | 0 | 0 |  | 3 | 0 | 5 |  | 26 | 0 | 60 |  | 29 | 0 | 59 |
|  |  | Drownings | 0 | 0 | 0 |  | 50 | 8 | 93 |  | 380 | 0 | 869 |  | 101 | 0 | 205 |
|  |  | Other unintentional injuries | 0 | 0 | 0 |  | 35 | 5 | 64 |  | 388 | 0 | 888 |  | 95 | 0 | 193 |
|  | Intentional injuries | | 0 | 0 | 0 |  | 165 | 25 | 305 |  | 1798 | 0 | 4109 |  | 393 | 0 | 801 |
|  |  | Self-inflicted injuries | 0 | 0 | 0 |  | 163 | 25 | 302 |  | 1787 | 0 | 4083 |  | 393 | 0 | 800 |
|  |  | Violence | 0 | 0 | 0 |  | 0 | 0 | 0 |  | 0 | 0 | 0 |  | 0 | 0 | 0 |
|  |  | Other intentional injuries | 0 | 0 | 0 |  | 2 | 0 | 3 |  | 11 | 0 | 25 |  | 0 | 0 | 1 |
| Men | |  |  |  |  |  |  |  |  |  |  |  |  |  |  |  |  |
| Injuries | |  | 0 | 0 | 0 |  | 7075 | 3503 | 10647 |  | 48309 | 18982 | 77636 |  | 11881 | 3978 | 19784 |
|  | Unintentional injuries | | 0 | 0 | 0 |  | 5967 | 2999 | 8936 |  | 36118 | 14358 | 57879 |  | 7110 | 2435 | 11786 |
|  |  | Transport injuries | 0 | 0 | 0 |  | 2328 | 1342 | 3314 |  | 9194 | 4146 | 14243 |  | 844 | 409 | 1279 |
|  |  | Poisonings | 0 | 0 | 0 |  | 405 | 184 | 625 |  | 4317 | 1638 | 6997 |  | 646 | 209 | 1083 |
|  |  | Falls | 0 | 0 | 0 |  | 835 | 380 | 1289 |  | 8495 | 3222 | 13768 |  | 2997 | 969 | 5024 |
|  |  | Fires, heat and hot substances | 0 | 0 | 0 |  | 21 | 10 | 33 |  | 478 | 181 | 775 |  | 332 | 107 | 557 |
|  |  | Drownings | 0 | 0 | 0 |  | 1061 | 483 | 1638 |  | 3944 | 1496 | 6392 |  | 885 | 286 | 1483 |
|  |  | Other unintentional injuries | 0 | 0 | 0 |  | 1318 | 600 | 2036 |  | 9689 | 3675 | 15703 |  | 1407 | 455 | 2359 |
|  | Intentional injuries | | 0 | 0 | 0 |  | 1108 | 504 | 1712 |  | 12190 | 4624 | 19757 |  | 4771 | 1543 | 7999 |
|  |  | Self-inflicted injuries | 0 | 0 | 0 |  | 1040 | 474 | 1607 |  | 11923 | 4522 | 19324 |  | 4762 | 1540 | 7984 |
|  |  | Violence | 0 | 0 | 0 |  | 0 | 0 | 0 |  | 0 | 0 | 0 |  | 0 | 0 | 0 |
|  |  | Other intentional injuries | 0 | 0 | 0 |  | 68 | 31 | 104 |  | 267 | 101 | 433 |  | 9 | 3 | 14 |

Table 4: Deaths from injuries attributable to alcohol consumption (without harms to others): Asia South

|  |  |  | 0 to 14 years of age | | |  | 15 to 34 years of age | | |  | 35 to 64 years of age | | |  | 65 years of age and older | | |
| --- | --- | --- | --- | --- | --- | --- | --- | --- | --- | --- | --- | --- | --- | --- | --- | --- | --- |
|  |  |  | Point estimate | Lower 95% CI | Upper 95% CI |  | Point estimate | Lower 95% CI | Upper 95% CI |  | Point estimate | Lower 95% CI | Upper 95% CI |  | Point estimate | Lower 95% CI | Upper 95% CI |
| Women | |  |  |  |  |  |  |  |  |  |  |  |  |  |  |  |  |
| Injuries | |  | 0 | 0 | 0 |  | 211 | 19 | 4452 |  | 1703 | 46 | 28365 |  | 50 | 0 | 2466 |
|  | Unintentional injuries | | 0 | 0 | 0 |  | 157 | 19 | 3066 |  | 1376 | 46 | 22353 |  | 48 | 0 | 2274 |
|  |  | Transport injuries | 0 | 0 | 0 |  | 40 | 19 | 61 |  | 181 | 46 | 316 |  | 26 | 0 | 52 |
|  |  | Poisonings | 0 | 0 | 0 |  | 7 | 0 | 181 |  | 210 | 0 | 3877 |  | 3 | 0 | 344 |
|  |  | Falls | 0 | 0 | 0 |  | 5 | 0 | 123 |  | 120 | 0 | 2208 |  | 6 | 0 | 634 |
|  |  | Fires, heat and hot substances | 0 | 0 | 0 |  | 63 | 0 | 1613 |  | 396 | 0 | 7294 |  | 3 | 0 | 336 |
|  |  | Drownings | 0 | 0 | 0 |  | 9 | 0 | 228 |  | 72 | 0 | 1335 |  | 1 | 0 | 136 |
|  |  | Other unintentional injuries | 0 | 0 | 0 |  | 33 | 0 | 859 |  | 397 | 0 | 7322 |  | 8 | 0 | 771 |
|  | Intentional injuries | | 0 | 0 | 0 |  | 54 | 0 | 1386 |  | 326 | 0 | 6011 |  | 2 | 0 | 193 |
|  |  | Self-inflicted injuries | 0 | 0 | 0 |  | 54 | 0 | 1378 |  | 321 | 0 | 5921 |  | 2 | 0 | 183 |
|  |  | Violence | 0 | 0 | 0 |  | 0 | 0 | 0 |  | 0 | 0 | 0 |  | 0 | 0 | 0 |
|  |  | Other intentional injuries | 0 | 0 | 0 |  | 0 | 0 | 8 |  | 5 | 0 | 91 |  | 0 | 0 | 9 |
| Men | |  |  |  |  |  |  |  |  |  |  |  |  |  |  |  |  |
| Injuries | |  | 0 | 0 | 0 |  | 13769 | 3022 | 24516 |  | 41628 | 7139 | 76117 |  | 7199 | 15 | 14385 |
|  | Unintentional injuries | | 0 | 0 | 0 |  | 9705 | 2419 | 16991 |  | 32834 | 5864 | 59805 |  | 6249 | 13 | 12488 |
|  |  | Transport injuries | 0 | 0 | 0 |  | 2936 | 1416 | 4456 |  | 10166 | 2578 | 17754 |  | 818 | 0 | 1639 |
|  |  | Poisonings | 0 | 0 | 0 |  | 637 | 94 | 1179 |  | 4351 | 631 | 8070 |  | 577 | 1 | 1152 |
|  |  | Falls | 0 | 0 | 0 |  | 678 | 101 | 1256 |  | 3277 | 475 | 6080 |  | 2315 | 6 | 4624 |
|  |  | Fires, heat and hot substances | 0 | 0 | 0 |  | 1275 | 189 | 2362 |  | 3323 | 482 | 6165 |  | 344 | 1 | 687 |
|  |  | Drownings | 0 | 0 | 0 |  | 1138 | 169 | 2107 |  | 2313 | 335 | 4292 |  | 400 | 1 | 799 |
|  |  | Other unintentional injuries | 0 | 0 | 0 |  | 3041 | 451 | 5630 |  | 9403 | 1363 | 17443 |  | 1796 | 4 | 3587 |
|  | Intentional injuries | | 0 | 0 | 0 |  | 4064 | 602 | 7525 |  | 8794 | 1275 | 16312 |  | 950 | 2 | 1897 |
|  |  | Self-inflicted injuries | 0 | 0 | 0 |  | 3964 | 588 | 7341 |  | 8522 | 1235 | 15809 |  | 880 | 2 | 1758 |
|  |  | Violence | 0 | 0 | 0 |  | 0 | 0 | 0 |  | 0 | 0 | 0 |  | 0 | 0 | 0 |
|  |  | Other intentional injuries | 0 | 0 | 0 |  | 100 | 15 | 185 |  | 271 | 39 | 503 |  | 70 | 0 | 139 |

Table 5: Deaths from injuries attributable to alcohol consumption (without harms to others): Asia Southeast

|  |  |  | 0 to 14 years of age | | |  | 15 to 34 years of age | | |  | 35 to 64 years of age | | |  | 65 years of age and older | | |
| --- | --- | --- | --- | --- | --- | --- | --- | --- | --- | --- | --- | --- | --- | --- | --- | --- | --- |
|  |  |  | Point estimate | Lower 95% CI | Upper 95% CI |  | Point estimate | Lower 95% CI | Upper 95% CI |  | Point estimate | Lower 95% CI | Upper 95% CI |  | Point estimate | Lower 95% CI | Upper 95% CI |
| Women | |  |  |  |  |  |  |  |  |  |  |  |  |  |  |  |  |
| Injuries | |  | 0 | 0 | 0 |  | 303 | 15 | 1139 |  | 869 | 0 | 3706 |  | 25 | 0 | 81 |
|  | Unintentional injuries | | 0 | 0 | 0 |  | 251 | 15 | 933 |  | 767 | 0 | 3248 |  | 24 | 0 | 76 |
|  |  | Transport injuries | 0 | 0 | 0 |  | 25 | 15 | 35 |  | 101 | 0 | 240 |  | 7 | 0 | 14 |
|  |  | Poisonings | 0 | 0 | 0 |  | 5 | 0 | 20 |  | 37 | 0 | 165 |  | 1 | 0 | 4 |
|  |  | Falls | 0 | 0 | 0 |  | 4 | 0 | 17 |  | 32 | 0 | 146 |  | 4 | 0 | 14 |
|  |  | Fires, heat and hot substances | 0 | 0 | 0 |  | 36 | 0 | 142 |  | 64 | 0 | 288 |  | 1 | 0 | 4 |
|  |  | Drownings | 0 | 0 | 0 |  | 10 | 0 | 41 |  | 30 | 0 | 137 |  | 1 | 0 | 3 |
|  |  | Other unintentional injuries | 0 | 0 | 0 |  | 171 | 0 | 678 |  | 503 | 0 | 2271 |  | 10 | 0 | 37 |
|  | Intentional injuries | | 0 | 0 | 0 |  | 52 | 0 | 206 |  | 101 | 0 | 458 |  | 1 | 0 | 5 |
|  |  | Self-inflicted injuries | 0 | 0 | 0 |  | 52 | 0 | 205 |  | 100 | 0 | 454 |  | 1 | 0 | 4 |
|  |  | Violence | 0 | 0 | 0 |  | 0 | 0 | 0 |  | 0 | 0 | 0 |  | 0 | 0 | 0 |
|  |  | Other intentional injuries | 0 | 0 | 0 |  | 0 | 0 | 1 |  | 1 | 0 | 4 |  | 0 | 0 | 0 |
| Men | |  |  |  |  |  |  |  |  |  |  |  |  |  |  |  |  |
| Injuries | |  | 0 | 0 | 0 |  | 4806 | 1859 | 7753 |  | 20443 | 4396 | 37900 |  | 2432 | 480 | 4384 |
|  | Unintentional injuries | | 0 | 0 | 0 |  | 3907 | 1588 | 6225 |  | 16854 | 3450 | 31668 |  | 2049 | 400 | 3698 |
|  |  | Transport injuries | 0 | 0 | 0 |  | 1421 | 840 | 2002 |  | 3760 | 0 | 8931 |  | 161 | 6 | 316 |
|  |  | Poisonings | 0 | 0 | 0 |  | 79 | 24 | 134 |  | 830 | 219 | 1442 |  | 102 | 21 | 182 |
|  |  | Falls | 0 | 0 | 0 |  | 148 | 44 | 251 |  | 1405 | 370 | 2439 |  | 523 | 109 | 937 |
|  |  | Fires, heat and hot substances | 0 | 0 | 0 |  | 122 | 37 | 208 |  | 522 | 138 | 906 |  | 62 | 13 | 111 |
|  |  | Drownings | 0 | 0 | 0 |  | 327 | 98 | 555 |  | 1110 | 293 | 1928 |  | 141 | 29 | 252 |
|  |  | Other unintentional injuries | 0 | 0 | 0 |  | 1810 | 545 | 3076 |  | 9227 | 2431 | 16022 |  | 1060 | 221 | 1900 |
|  | Intentional injuries | | 0 | 0 | 0 |  | 900 | 271 | 1528 |  | 3589 | 946 | 6232 |  | 383 | 80 | 686 |
|  |  | Self-inflicted injuries | 0 | 0 | 0 |  | 876 | 264 | 1489 |  | 3480 | 917 | 6043 |  | 373 | 78 | 668 |
|  |  | Violence | 0 | 0 | 0 |  | 0 | 0 | 0 |  | 0 | 0 | 0 |  | 0 | 0 | 0 |
|  |  | Other intentional injuries | 0 | 0 | 0 |  | 23 | 7 | 40 |  | 109 | 29 | 190 |  | 10 | 2 | 18 |

Table 6: Deaths from injuries attributable to alcohol consumption (without harms to others): Australiasia

|  |  |  | 0 to 14 years of age | | |  | 15 to 34 years of age | | |  | 35 to 64 years of age | | |  | 65 years of age and older | | |
| --- | --- | --- | --- | --- | --- | --- | --- | --- | --- | --- | --- | --- | --- | --- | --- | --- | --- |
|  |  |  | Point estimate | Lower 95% CI | Upper 95% CI |  | Point estimate | Lower 95% CI | Upper 95% CI |  | Point estimate | Lower 95% CI | Upper 95% CI |  | Point estimate | Lower 95% CI | Upper 95% CI |
| Women | |  |  |  |  |  |  |  |  |  |  |  |  |  |  |  |  |
| Injuries | |  | 0 | 0 | 0 |  | 28 | 14 | 42 |  | 45 | 22 | 69 |  | 43 | 20 | 65 |
|  | Unintentional injuries | | 0 | 0 | 0 |  | 16 | 8 | 23 |  | 24 | 12 | 37 |  | 40 | 19 | 62 |
|  |  | Transport injuries | 0 | 0 | 0 |  | 5 | 3 | 8 |  | 7 | 4 | 10 |  | 2 | 1 | 3 |
|  |  | Poisonings | 0 | 0 | 0 |  | 5 | 2 | 8 |  | 7 | 3 | 11 |  | 1 | 1 | 2 |
|  |  | Falls | 0 | 0 | 0 |  | 1 | 0 | 1 |  | 2 | 1 | 3 |  | 14 | 7 | 22 |
|  |  | Fires, heat and hot substances | 0 | 0 | 0 |  | 0 | 0 | 1 |  | 1 | 0 | 1 |  | 1 | 0 | 1 |
|  |  | Drownings | 0 | 0 | 0 |  | 1 | 0 | 1 |  | 1 | 1 | 2 |  | 0 | 0 | 1 |
|  |  | Other unintentional injuries | 0 | 0 | 0 |  | 4 | 2 | 5 |  | 7 | 3 | 10 |  | 22 | 10 | 34 |
|  | Intentional injuries | | 0 | 0 | 0 |  | 13 | 6 | 20 |  | 21 | 10 | 32 |  | 2 | 1 | 4 |
|  |  | Self-inflicted injuries | 0 | 0 | 0 |  | 13 | 6 | 20 |  | 21 | 10 | 32 |  | 2 | 1 | 4 |
|  |  | Violence | 0 | 0 | 0 |  | 0 | 0 | 0 |  | 0 | 0 | 0 |  | 0 | 0 | 0 |
|  |  | Other intentional injuries | 0 | 0 | 0 |  | 0 | 0 | 0 |  | 0 | 0 | 0 |  | 0 | 0 | 0 |
| Men | |  |  |  |  |  |  |  |  |  |  |  |  |  |  |  |  |
| Injuries | |  | 0 | 0 | 0 |  | 307 | 150 | 464 |  | 551 | 268 | 835 |  | 232 | 103 | 362 |
|  | Unintentional injuries | | 0 | 0 | 0 |  | 159 | 81 | 236 |  | 277 | 136 | 418 |  | 185 | 82 | 288 |
|  |  | Transport injuries | 0 | 0 | 0 |  | 47 | 29 | 66 |  | 43 | 24 | 63 |  | 7 | 3 | 10 |
|  |  | Poisonings | 0 | 0 | 0 |  | 40 | 19 | 62 |  | 67 | 32 | 102 |  | 4 | 2 | 6 |
|  |  | Falls | 0 | 0 | 0 |  | 12 | 5 | 18 |  | 31 | 15 | 48 |  | 62 | 27 | 97 |
|  |  | Fires, heat and hot substances | 0 | 0 | 0 |  | 2 | 1 | 2 |  | 8 | 4 | 12 |  | 4 | 2 | 7 |
|  |  | Drownings | 0 | 0 | 0 |  | 12 | 6 | 19 |  | 20 | 9 | 30 |  | 5 | 2 | 8 |
|  |  | Other unintentional injuries | 0 | 0 | 0 |  | 46 | 21 | 70 |  | 108 | 52 | 164 |  | 103 | 46 | 160 |
|  | Intentional injuries | | 0 | 0 | 0 |  | 148 | 69 | 228 |  | 275 | 132 | 417 |  | 47 | 21 | 74 |
|  |  | Self-inflicted injuries | 0 | 0 | 0 |  | 148 | 69 | 228 |  | 274 | 132 | 416 |  | 47 | 21 | 74 |
|  |  | Violence | 0 | 0 | 0 |  | 0 | 0 | 0 |  | 0 | 0 | 0 |  | 0 | 0 | 0 |
|  |  | Other intentional injuries | 0 | 0 | 0 |  | 0 | 0 | 0 |  | 0 | 0 | 1 |  | 0 | 0 | 0 |

Table 7: Deaths from injuries attributable to alcohol consumption (without harms to others): Caribbean

|  |  |  | 0 to 14 years of age | | |  | 15 to 34 years of age | | |  | 35 to 64 years of age | | |  | 65 years of age and older | | |
| --- | --- | --- | --- | --- | --- | --- | --- | --- | --- | --- | --- | --- | --- | --- | --- | --- | --- |
|  |  |  | Point estimate | Lower 95% CI | Upper 95% CI |  | Point estimate | Lower 95% CI | Upper 95% CI |  | Point estimate | Lower 95% CI | Upper 95% CI |  | Point estimate | Lower 95% CI | Upper 95% CI |
| Women | |  |  |  |  |  |  |  |  |  |  |  |  |  |  |  |  |
| Injuries | |  | 0 | 0 | 0 |  | 83 | 16 | 151 |  | 36 | 16 | 57 |  | 8 | 2 | 13 |
|  | Unintentional injuries | | 0 | 0 | 0 |  | 69 | 14 | 125 |  | 31 | 14 | 48 |  | 8 | 2 | 13 |
|  |  | Transport injuries | 0 | 0 | 0 |  | 11 | 7 | 15 |  | 14 | 8 | 19 |  | 2 | 1 | 3 |
|  |  | Poisonings | 0 | 0 | 0 |  | 1 | 0 | 2 |  | 0 | 0 | 1 |  | 0 | 0 | 0 |
|  |  | Falls | 0 | 0 | 0 |  | 0 | 0 | 1 |  | 1 | 0 | 2 |  | 3 | 1 | 5 |
|  |  | Fires, heat and hot substances | 0 | 0 | 0 |  | 1 | 0 | 2 |  | 1 | 0 | 1 |  | 0 | 0 | 0 |
|  |  | Drownings | 0 | 0 | 0 |  | 2 | 0 | 3 |  | 1 | 0 | 1 |  | 0 | 0 | 0 |
|  |  | Other unintentional injuries | 0 | 0 | 0 |  | 54 | 7 | 101 |  | 15 | 5 | 24 |  | 3 | 1 | 5 |
|  | Intentional injuries | | 0 | 0 | 0 |  | 14 | 2 | 26 |  | 6 | 2 | 9 |  | 0 | 0 | 1 |
|  |  | Self-inflicted injuries | 0 | 0 | 0 |  | 14 | 2 | 26 |  | 6 | 2 | 9 |  | 0 | 0 | 1 |
|  |  | Violence | 0 | 0 | 0 |  | 0 | 0 | 0 |  | 0 | 0 | 0 |  | 0 | 0 | 0 |
|  |  | Other intentional injuries | 0 | 0 | 0 |  | 0 | 0 | 0 |  | 0 | 0 | 0 |  | 0 | 0 | 0 |
| Men | |  |  |  |  |  |  |  |  |  |  |  |  |  |  |  |  |
| Injuries | |  | 0 | 0 | 0 |  | 497 | 237 | 758 |  | 824 | 373 | 1276 |  | 217 | 80 | 354 |
|  | Unintentional injuries | | 0 | 0 | 0 |  | 404 | 199 | 610 |  | 606 | 283 | 928 |  | 172 | 64 | 280 |
|  |  | Transport injuries | 0 | 0 | 0 |  | 168 | 103 | 233 |  | 188 | 112 | 265 |  | 20 | 10 | 30 |
|  |  | Poisonings | 0 | 0 | 0 |  | 19 | 8 | 31 |  | 34 | 14 | 54 |  | 2 | 1 | 4 |
|  |  | Falls | 0 | 0 | 0 |  | 12 | 5 | 19 |  | 49 | 20 | 77 |  | 64 | 23 | 105 |
|  |  | Fires, heat and hot substances | 0 | 0 | 0 |  | 2 | 1 | 3 |  | 7 | 3 | 11 |  | 2 | 1 | 4 |
|  |  | Drownings | 0 | 0 | 0 |  | 32 | 13 | 52 |  | 45 | 19 | 72 |  | 5 | 2 | 8 |
|  |  | Other unintentional injuries | 0 | 0 | 0 |  | 170 | 69 | 272 |  | 283 | 116 | 449 |  | 78 | 28 | 128 |
|  | Intentional injuries | | 0 | 0 | 0 |  | 93 | 38 | 148 |  | 218 | 90 | 347 |  | 45 | 16 | 74 |
|  |  | Self-inflicted injuries | 0 | 0 | 0 |  | 92 | 37 | 146 |  | 217 | 89 | 345 |  | 45 | 16 | 74 |
|  |  | Violence | 0 | 0 | 0 |  | 0 | 0 | 0 |  | 0 | 0 | 0 |  | 0 | 0 | 0 |
|  |  | Other intentional injuries | 0 | 0 | 0 |  | 1 | 0 | 2 |  | 1 | 1 | 2 |  | 0 | 0 | 0 |

Table 8: Deaths from injuries attributable to alcohol consumption (without harms to others): Europe Central

|  |  |  | 0 to 14 years of age | | |  | 15 to 34 years of age | | |  | 35 to 64 years of age | | |  | 65 years of age and older | | |
| --- | --- | --- | --- | --- | --- | --- | --- | --- | --- | --- | --- | --- | --- | --- | --- | --- | --- |
|  |  |  | Point estimate | Lower 95% CI | Upper 95% CI |  | Point estimate | Lower 95% CI | Upper 95% CI |  | Point estimate | Lower 95% CI | Upper 95% CI |  | Point estimate | Lower 95% CI | Upper 95% CI |
| Women | |  |  |  |  |  |  |  |  |  |  |  |  |  |  |  |  |
| Injuries | |  | 0 | 0 | 0 |  | 240 | 46 | 434 |  | 783 | 70 | 1496 |  | 152 | 33 | 271 |
|  | Unintentional injuries | | 0 | 0 | 0 |  | 159 | 45 | 274 |  | 505 | 60 | 951 |  | 138 | 30 | 247 |
|  |  | Transport injuries | 0 | 0 | 0 |  | 72 | 44 | 101 |  | 159 | 46 | 271 |  | 60 | 15 | 104 |
|  |  | Poisonings | 0 | 0 | 0 |  | 21 | 0 | 41 |  | 60 | 2 | 118 |  | 3 | 1 | 5 |
|  |  | Falls | 0 | 0 | 0 |  | 11 | 0 | 22 |  | 78 | 3 | 154 |  | 56 | 11 | 101 |
|  |  | Fires, heat and hot substances | 0 | 0 | 0 |  | 4 | 0 | 8 |  | 19 | 1 | 38 |  | 3 | 1 | 5 |
|  |  | Drownings | 0 | 0 | 0 |  | 10 | 0 | 21 |  | 40 | 2 | 78 |  | 2 | 0 | 4 |
|  |  | Other unintentional injuries | 0 | 0 | 0 |  | 41 | 1 | 81 |  | 149 | 6 | 292 |  | 15 | 3 | 27 |
|  | Intentional injuries | | 0 | 0 | 0 |  | 81 | 1 | 160 |  | 278 | 11 | 545 |  | 14 | 3 | 25 |
|  |  | Self-inflicted injuries | 0 | 0 | 0 |  | 80 | 1 | 159 |  | 277 | 11 | 544 |  | 14 | 3 | 25 |
|  |  | Violence | 0 | 0 | 0 |  | 0 | 0 | 0 |  | 0 | 0 | 0 |  | 0 | 0 | 0 |
|  |  | Other intentional injuries | 0 | 0 | 0 |  | 0 | 0 | 1 |  | 0 | 0 | 0 |  | 0 | 0 | 0 |
| Men | |  |  |  |  |  |  |  |  |  |  |  |  |  |  |  |  |
| Injuries | |  | 0 | 0 | 0 |  | 4051 | 2009 | 6094 |  | 16152 | 7616 | 22577 |  | 4586 | 1931 | 7241 |
|  | Unintentional injuries | | 0 | 0 | 0 |  | 2647 | 1364 | 3930 |  | 10252 | 4666 | 14623 |  | 3225 | 1339 | 5110 |
|  |  | Transport injuries | 0 | 0 | 0 |  | 975 | 596 | 1354 |  | 2224 | 651 | 3797 |  | 348 | 88 | 608 |
|  |  | Poisonings | 0 | 0 | 0 |  | 243 | 112 | 375 |  | 1290 | 645 | 1740 |  | 185 | 80 | 290 |
|  |  | Falls | 0 | 0 | 0 |  | 215 | 99 | 332 |  | 1860 | 930 | 2508 |  | 1446 | 629 | 2263 |
|  |  | Fires, heat and hot substances | 0 | 0 | 0 |  | 52 | 24 | 80 |  | 343 | 172 | 463 |  | 124 | 54 | 193 |
|  |  | Drownings | 0 | 0 | 0 |  | 307 | 141 | 473 |  | 874 | 437 | 1179 |  | 176 | 77 | 276 |
|  |  | Other unintentional injuries | 0 | 0 | 0 |  | 854 | 392 | 1316 |  | 3660 | 1830 | 4935 |  | 946 | 411 | 1481 |
|  | Intentional injuries | | 0 | 0 | 0 |  | 1405 | 645 | 2164 |  | 5899 | 2950 | 7954 |  | 1362 | 592 | 2131 |
|  |  | Self-inflicted injuries | 0 | 0 | 0 |  | 1403 | 644 | 2161 |  | 5897 | 2949 | 7951 |  | 1361 | 592 | 2131 |
|  |  | Violence | 0 | 0 | 0 |  | 0 | 0 | 0 |  | 0 | 0 | 0 |  | 0 | 0 | 0 |
|  |  | Other intentional injuries | 0 | 0 | 0 |  | 2 | 1 | 3 |  | 2 | 1 | 3 |  | 0 | 0 | 0 |

Table 9: Deaths from injuries attributable to alcohol consumption (without harms to others): Europe Eastern

|  |  |  | 0 to 14 years of age | | |  | 15 to 34 years of age | | |  | 35 to 64 years of age | | |  | 65 years of age and older | | |
| --- | --- | --- | --- | --- | --- | --- | --- | --- | --- | --- | --- | --- | --- | --- | --- | --- | --- |
|  |  |  | Point estimate | Lower 95% CI | Upper 95% CI |  | Point estimate | Lower 95% CI | Upper 95% CI |  | Point estimate | Lower 95% CI | Upper 95% CI |  | Point estimate | Lower 95% CI | Upper 95% CI |
| Women | |  |  |  |  |  |  |  |  |  |  |  |  |  |  |  |  |
| Injuries | |  | 0 | 0 | 0 |  | 4817 | 1772 | 7862 |  | 10707 | 2426 | 18989 |  | 1500 | 753 | 2147 |
|  | Unintentional injuries | | 0 | 0 | 0 |  | 4004 | 1681 | 6328 |  | 9471 | 2337 | 16605 |  | 1393 | 753 | 1917 |
|  |  | Transport injuries | 0 | 0 | 0 |  | 1385 | 1385 | 1385 |  | 1781 | 1781 | 1781 |  | 982 | 753 | 1036 |
|  |  | Poisonings | 0 | 0 | 0 |  | 957 | 108 | 1806 |  | 3104 | 224 | 5984 |  | 96 | 0 | 205 |
|  |  | Falls | 0 | 0 | 0 |  | 169 | 19 | 318 |  | 457 | 33 | 882 |  | 65 | 0 | 139 |
|  |  | Fires, heat and hot substances | 0 | 0 | 0 |  | 126 | 14 | 237 |  | 479 | 35 | 923 |  | 55 | 0 | 118 |
|  |  | Drownings | 0 | 0 | 0 |  | 223 | 25 | 421 |  | 355 | 26 | 683 |  | 22 | 0 | 47 |
|  |  | Other unintentional injuries | 0 | 0 | 0 |  | 1145 | 129 | 2160 |  | 3295 | 238 | 6352 |  | 174 | 0 | 372 |
|  | Intentional injuries | | 0 | 0 | 0 |  | 813 | 92 | 1534 |  | 1237 | 89 | 2384 |  | 107 | 0 | 230 |
|  |  | Self-inflicted injuries | 0 | 0 | 0 |  | 813 | 92 | 1534 |  | 1237 | 89 | 2384 |  | 107 | 0 | 230 |
|  |  | Violence | 0 | 0 | 0 |  | 0 | 0 | 0 |  | 0 | 0 | 0 |  | 0 | 0 | 0 |
|  |  | Other intentional injuries | 0 | 0 | 0 |  | 0 | 0 | 0 |  | 0 | 0 | 0 |  | 0 | 0 | 0 |
| Men | |  |  |  |  |  |  |  |  |  |  |  |  |  |  |  |  |
| Injuries | |  | 0 | 0 | 0 |  | 77846 | 50989 | 82686 |  | 143382 | 87351 | 168631 |  | 13028 | 6841 | 18743 |
|  | Unintentional injuries | | 0 | 0 | 0 |  | 60287 | 41192 | 63728 |  | 118946 | 73852 | 139267 |  | 10305 | 5579 | 14559 |
|  |  | Transport injuries | 0 | 0 | 0 |  | 17094 | 17094 | 17094 |  | 18196 | 18196 | 18196 |  | 2645 | 2029 | 2788 |
|  |  | Poisonings | 0 | 0 | 0 |  | 14948 | 8340 | 16138 |  | 36071 | 19926 | 43347 |  | 2071 | 960 | 3183 |
|  |  | Falls | 0 | 0 | 0 |  | 2735 | 1526 | 2953 |  | 7103 | 3924 | 8536 |  | 944 | 437 | 1450 |
|  |  | Fires, heat and hot substances | 0 | 0 | 0 |  | 1765 | 985 | 1905 |  | 5772 | 3189 | 6936 |  | 676 | 313 | 1039 |
|  |  | Drownings | 0 | 0 | 0 |  | 4815 | 2686 | 5198 |  | 7338 | 4053 | 8817 |  | 520 | 241 | 799 |
|  |  | Other unintentional injuries | 0 | 0 | 0 |  | 18931 | 10562 | 20439 |  | 44465 | 24563 | 53434 |  | 3449 | 1599 | 5300 |
|  | Intentional injuries | | 0 | 0 | 0 |  | 17559 | 9797 | 18958 |  | 24436 | 13499 | 29364 |  | 2723 | 1262 | 4184 |
|  |  | Self-inflicted injuries | 0 | 0 | 0 |  | 17559 | 9797 | 18958 |  | 24436 | 13499 | 29364 |  | 2723 | 1262 | 4184 |
|  |  | Violence | 0 | 0 | 0 |  | 0 | 0 | 0 |  | 0 | 0 | 0 |  | 0 | 0 | 0 |
|  |  | Other intentional injuries | 0 | 0 | 0 |  | 0 | 0 | 0 |  | 0 | 0 | 0 |  | 0 | 0 | 0 |

Table 10: Deaths from injuries attributable to alcohol consumption (without harms to others): Europe Western

|  |  |  | 0 to 14 years of age | | |  | 15 to 34 years of age | | |  | 35 to 64 years of age | | |  | 65 years of age and older | | |
| --- | --- | --- | --- | --- | --- | --- | --- | --- | --- | --- | --- | --- | --- | --- | --- | --- | --- |
|  |  |  | Point estimate | Lower 95% CI | Upper 95% CI |  | Point estimate | Lower 95% CI | Upper 95% CI |  | Point estimate | Lower 95% CI | Upper 95% CI |  | Point estimate | Lower 95% CI | Upper 95% CI |
| Women | |  |  |  |  |  |  |  |  |  |  |  |  |  |  |  |  |
| Injuries | |  | 0 | 0 | 0 |  | 329 | 140 | 517 |  | 1381 | 205 | 2557 |  | 1732 | 632 | 2832 |
|  | Unintentional injuries | | 0 | 0 | 0 |  | 194 | 91 | 296 |  | 681 | 124 | 1239 |  | 1570 | 573 | 2568 |
|  |  | Transport injuries | 0 | 0 | 0 |  | 115 | 63 | 167 |  | 162 | 64 | 261 |  | 57 | 22 | 91 |
|  |  | Poisonings | 0 | 0 | 0 |  | 25 | 9 | 41 |  | 98 | 11 | 184 |  | 27 | 10 | 44 |
|  |  | Falls | 0 | 0 | 0 |  | 10 | 4 | 17 |  | 147 | 17 | 276 |  | 804 | 293 | 1316 |
|  |  | Fires, heat and hot substances | 0 | 0 | 0 |  | 6 | 2 | 9 |  | 35 | 4 | 66 |  | 27 | 10 | 45 |
|  |  | Drownings | 0 | 0 | 0 |  | 7 | 3 | 12 |  | 36 | 4 | 68 |  | 17 | 6 | 27 |
|  |  | Other unintentional injuries | 0 | 0 | 0 |  | 31 | 11 | 50 |  | 204 | 24 | 384 |  | 639 | 233 | 1046 |
|  | Intentional injuries | | 0 | 0 | 0 |  | 135 | 49 | 221 |  | 700 | 81 | 1318 |  | 162 | 59 | 264 |
|  |  | Self-inflicted injuries | 0 | 0 | 0 |  | 135 | 49 | 221 |  | 700 | 81 | 1318 |  | 162 | 59 | 264 |
|  |  | Violence | 0 | 0 | 0 |  | 0 | 0 | 0 |  | 0 | 0 | 0 |  | 0 | 0 | 0 |
|  |  | Other intentional injuries | 0 | 0 | 0 |  | 0 | 0 | 0 |  | 0 | 0 | 0 |  | 0 | 0 | 0 |
| Men | |  |  |  |  |  |  |  |  |  |  |  |  |  |  |  |  |
| Injuries | |  | 0 | 0 | 0 |  | 5077 | 2398 | 7756 |  | 14425 | 6652 | 22198 |  | 7678 | 3211 | 12145 |
|  | Unintentional injuries | | 0 | 0 | 0 |  | 2846 | 1397 | 4295 |  | 7273 | 3299 | 11248 |  | 5719 | 2390 | 9048 |
|  |  | Transport injuries | 0 | 0 | 0 |  | 1254 | 682 | 1825 |  | 1445 | 566 | 2324 |  | 245 | 97 | 393 |
|  |  | Poisonings | 0 | 0 | 0 |  | 432 | 194 | 670 |  | 908 | 426 | 1391 |  | 105 | 44 | 167 |
|  |  | Falls | 0 | 0 | 0 |  | 253 | 114 | 393 |  | 1673 | 785 | 2562 |  | 2658 | 1114 | 4202 |
|  |  | Fires, heat and hot substances | 0 | 0 | 0 |  | 63 | 28 | 98 |  | 274 | 129 | 420 |  | 130 | 54 | 205 |
|  |  | Drownings | 0 | 0 | 0 |  | 201 | 90 | 312 |  | 524 | 246 | 803 |  | 180 | 75 | 285 |
|  |  | Other unintentional injuries | 0 | 0 | 0 |  | 643 | 289 | 998 |  | 2448 | 1148 | 3748 |  | 2401 | 1006 | 3796 |
|  | Intentional injuries | | 0 | 0 | 0 |  | 2231 | 1001 | 3460 |  | 7152 | 3354 | 10950 |  | 1959 | 821 | 3097 |
|  |  | Self-inflicted injuries | 0 | 0 | 0 |  | 2229 | 1000 | 3457 |  | 7151 | 3353 | 10949 |  | 1959 | 821 | 3097 |
|  |  | Violence | 0 | 0 | 0 |  | 0 | 0 | 0 |  | 0 | 0 | 0 |  | 0 | 0 | 0 |
|  |  | Other intentional injuries | 0 | 0 | 0 |  | 2 | 1 | 3 |  | 1 | 0 | 1 |  | 0 | 0 | 0 |

Table 11: Deaths from injuries attributable to alcohol consumption (without harms to others): Latin America Andean

|  |  |  | 0 to 14 years of age | | |  | 15 to 34 years of age | | |  | 35 to 64 years of age | | |  | 65 years of age and older | | |
| --- | --- | --- | --- | --- | --- | --- | --- | --- | --- | --- | --- | --- | --- | --- | --- | --- | --- |
|  |  |  | Point estimate | Lower 95% CI | Upper 95% CI |  | Point estimate | Lower 95% CI | Upper 95% CI |  | Point estimate | Lower 95% CI | Upper 95% CI |  | Point estimate | Lower 95% CI | Upper 95% CI |
| Women | |  |  |  |  |  |  |  |  |  |  |  |  |  |  |  |  |
| Injuries | |  | 0 | 0 | 0 |  | 76 | 11 | 176 |  | 57 | 12 | 108 |  | 19 | 1 | 44 |
|  | Unintentional injuries | | 0 | 0 | 0 |  | 58 | 11 | 129 |  | 54 | 12 | 101 |  | 19 | 1 | 43 |
|  |  | Transport injuries | 0 | 0 | 0 |  | 18 | 11 | 25 |  | 25 | 12 | 38 |  | 2 | 1 | 2 |
|  |  | Poisonings | 0 | 0 | 0 |  | 2 | 0 | 5 |  | 1 | 0 | 2 |  | 0 | 0 | 1 |
|  |  | Falls | 0 | 0 | 0 |  | 1 | 0 | 3 |  | 1 | 0 | 2 |  | 1 | 0 | 2 |
|  |  | Fires, heat and hot substances | 0 | 0 | 0 |  | 2 | 0 | 4 |  | 1 | 0 | 3 |  | 1 | 0 | 1 |
|  |  | Drownings | 0 | 0 | 0 |  | 4 | 0 | 11 |  | 2 | 0 | 5 |  | 1 | 0 | 2 |
|  |  | Other unintentional injuries | 0 | 0 | 0 |  | 31 | 0 | 82 |  | 24 | 0 | 52 |  | 15 | 0 | 35 |
|  | Intentional injuries | | 0 | 0 | 0 |  | 18 | 0 | 47 |  | 3 | 0 | 7 |  | 0 | 0 | 1 |
|  |  | Self-inflicted injuries | 0 | 0 | 0 |  | 18 | 0 | 47 |  | 3 | 0 | 7 |  | 0 | 0 | 1 |
|  |  | Violence | 0 | 0 | 0 |  | 0 | 0 | 0 |  | 0 | 0 | 0 |  | 0 | 0 | 0 |
|  |  | Other intentional injuries | 0 | 0 | 0 |  | 0 | 0 | 0 |  | 0 | 0 | 0 |  | 0 | 0 | 0 |
| Men | |  |  |  |  |  |  |  |  |  |  |  |  |  |  |  |  |
| Injuries | |  | 0 | 0 | 0 |  | 786 | 226 | 1347 |  | 1339 | 385 | 2294 |  | 38 | 10 | 66 |
|  | Unintentional injuries | | 0 | 0 | 0 |  | 466 | 134 | 798 |  | 921 | 265 | 1578 |  | 32 | 8 | 55 |
|  |  | Transport injuries | 0 | 0 | 0 |  | 306 | 88 | 524 |  | 643 | 185 | 1102 |  | 20 | 5 | 34 |
|  |  | Poisonings | 0 | 0 | 0 |  | 15 | 4 | 25 |  | 49 | 14 | 83 |  | 1 | 0 | 2 |
|  |  | Falls | 0 | 0 | 0 |  | 20 | 6 | 35 |  | 60 | 17 | 103 |  | 4 | 1 | 6 |
|  |  | Fires, heat and hot substances | 0 | 0 | 0 |  | 11 | 3 | 19 |  | 25 | 7 | 43 |  | 2 | 0 | 3 |
|  |  | Drownings | 0 | 0 | 0 |  | 114 | 33 | 195 |  | 144 | 41 | 247 |  | 5 | 1 | 10 |
|  |  | Other unintentional injuries | 0 | 0 | 0 |  | 0 | 0 | 0 |  | 0 | 0 | 0 |  | 0 | 0 | 0 |
|  | Intentional injuries | | 0 | 0 | 0 |  | 321 | 92 | 549 |  | 418 | 120 | 716 |  | 6 | 2 | 11 |
|  |  | Self-inflicted injuries | 0 | 0 | 0 |  | 0 | 0 | 0 |  | 0 | 0 | 0 |  | 0 | 0 | 0 |
|  |  | Violence | 0 | 0 | 0 |  | 321 | 92 | 549 |  | 418 | 120 | 716 |  | 6 | 2 | 11 |
|  |  | Other intentional injuries | 0 | 0 | 0 |  | 0 | 0 | 0 |  | 0 | 0 | 0 |  | 0 | 0 | 0 |

Table 12: Deaths from injuries attributable to alcohol consumption (without harms to others): Latin America Central

|  |  |  | 0 to 14 years of age | | |  | 15 to 34 years of age | | |  | 35 to 64 years of age | | |  | 65 years of age and older | | |
| --- | --- | --- | --- | --- | --- | --- | --- | --- | --- | --- | --- | --- | --- | --- | --- | --- | --- |
|  |  |  | Point estimate | Lower 95% CI | Upper 95% CI |  | Point estimate | Lower 95% CI | Upper 95% CI |  | Point estimate | Lower 95% CI | Upper 95% CI |  | Point estimate | Lower 95% CI | Upper 95% CI |
| Women | |  |  |  |  |  |  |  |  |  |  |  |  |  |  |  |  |
| Injuries | |  | 0 | 0 | 0 |  | 400 | 164 | 691 |  | 366 | 197 | 557 |  | 139 | 65 | 212 |
|  | Unintentional injuries | | 0 | 0 | 0 |  | 325 | 164 | 512 |  | 347 | 197 | 513 |  | 138 | 65 | 210 |
|  |  | Transport injuries | 0 | 0 | 0 |  | 257 | 164 | 349 |  | 299 | 197 | 400 |  | 107 | 63 | 151 |
|  |  | Poisonings | 0 | 0 | 0 |  | 8 | 0 | 20 |  | 4 | 0 | 8 |  | 1 | 0 | 3 |
|  |  | Falls | 0 | 0 | 0 |  | 5 | 0 | 12 |  | 9 | 0 | 20 |  | 11 | 1 | 21 |
|  |  | Fires, heat and hot substances | 0 | 0 | 0 |  | 3 | 0 | 8 |  | 3 | 0 | 7 |  | 1 | 0 | 2 |
|  |  | Drownings | 0 | 0 | 0 |  | 15 | 0 | 35 |  | 4 | 0 | 10 |  | 1 | 0 | 1 |
|  |  | Other unintentional injuries | 0 | 0 | 0 |  | 37 | 0 | 88 |  | 29 | 0 | 68 |  | 16 | 1 | 32 |
|  | Intentional injuries | | 0 | 0 | 0 |  | 75 | 0 | 179 |  | 19 | 0 | 44 |  | 1 | 0 | 2 |
|  |  | Self-inflicted injuries | 0 | 0 | 0 |  | 74 | 0 | 175 |  | 19 | 0 | 44 |  | 1 | 0 | 2 |
|  |  | Violence | 0 | 0 | 0 |  | 0 | 0 | 0 |  | 0 | 0 | 0 |  | 0 | 0 | 0 |
|  |  | Other intentional injuries | 0 | 0 | 0 |  | 2 | 0 | 4 |  | 0 | 0 | 0 |  | 0 | 0 | 0 |
| Men | |  |  |  |  |  |  |  |  |  |  |  |  |  |  |  |  |
| Injuries | |  | 0 | 0 | 0 |  | 7603 | 4101 | 11105 |  | 8856 | 4966 | 12746 |  | 1989 | 919 | 3058 |
|  | Unintentional injuries | | 0 | 0 | 0 |  | 6254 | 3552 | 8956 |  | 7749 | 4494 | 11004 |  | 1826 | 861 | 2792 |
|  |  | Transport injuries | 0 | 0 | 0 |  | 4351 | 2778 | 5925 |  | 5078 | 3354 | 6803 |  | 891 | 525 | 1258 |
|  |  | Poisonings | 0 | 0 | 0 |  | 108 | 44 | 172 |  | 147 | 63 | 231 |  | 40 | 14 | 66 |
|  |  | Falls | 0 | 0 | 0 |  | 223 | 91 | 356 |  | 605 | 258 | 952 |  | 316 | 113 | 519 |
|  |  | Fires, heat and hot substances | 0 | 0 | 0 |  | 49 | 20 | 78 |  | 90 | 38 | 141 |  | 39 | 14 | 65 |
|  |  | Drownings | 0 | 0 | 0 |  | 548 | 223 | 873 |  | 420 | 179 | 662 |  | 75 | 27 | 123 |
|  |  | Other unintentional injuries | 0 | 0 | 0 |  | 975 | 397 | 1554 |  | 1409 | 601 | 2216 |  | 464 | 167 | 762 |
|  | Intentional injuries | | 0 | 0 | 0 |  | 1349 | 549 | 2149 |  | 1107 | 472 | 1741 |  | 162 | 58 | 266 |
|  |  | Self-inflicted injuries | 0 | 0 | 0 |  | 1168 | 475 | 1860 |  | 1064 | 454 | 1674 |  | 162 | 58 | 265 |
|  |  | Violence | 0 | 0 | 0 |  | 0 | 0 | 0 |  | 0 | 0 | 0 |  | 0 | 0 | 0 |
|  |  | Other intentional injuries | 0 | 0 | 0 |  | 181 | 74 | 289 |  | 43 | 18 | 68 |  | 1 | 0 | 1 |

Table 13: Deaths from injuries attributable to alcohol consumption (without harms to others): Latin America Southern

|  |  |  | 0 to 14 years of age | | |  | 15 to 34 years of age | | |  | 35 to 64 years of age | | |  | 65 years of age and older | | |
| --- | --- | --- | --- | --- | --- | --- | --- | --- | --- | --- | --- | --- | --- | --- | --- | --- | --- |
|  |  |  | Point estimate | Lower 95% CI | Upper 95% CI |  | Point estimate | Lower 95% CI | Upper 95% CI |  | Point estimate | Lower 95% CI | Upper 95% CI |  | Point estimate | Lower 95% CI | Upper 95% CI |
| Women | |  |  |  |  |  |  |  |  |  |  |  |  |  |  |  |  |
| Injuries | |  | 0 | 0 | 0 |  | 53 | 20 | 87 |  | 102 | 7 | 207 |  | 70 | 18 | 121 |
|  | Unintentional injuries | | 0 | 0 | 0 |  | 35 | 15 | 55 |  | 66 | 7 | 132 |  | 65 | 17 | 113 |
|  |  | Transport injuries | 0 | 0 | 0 |  | 17 | 10 | 24 |  | 14 | 7 | 21 |  | 3 | 2 | 5 |
|  |  | Poisonings | 0 | 0 | 0 |  | 1 | 0 | 2 |  | 2 | 0 | 4 |  | 1 | 0 | 2 |
|  |  | Falls | 0 | 0 | 0 |  | 0 | 0 | 1 |  | 2 | 0 | 4 |  | 11 | 3 | 19 |
|  |  | Fires, heat and hot substances | 0 | 0 | 0 |  | 2 | 0 | 3 |  | 5 | 0 | 11 |  | 3 | 1 | 5 |
|  |  | Drownings | 0 | 0 | 0 |  | 1 | 0 | 2 |  | 3 | 0 | 7 |  | 1 | 0 | 1 |
|  |  | Other unintentional injuries | 0 | 0 | 0 |  | 13 | 3 | 23 |  | 40 | 0 | 84 |  | 46 | 11 | 81 |
|  | Intentional injuries | | 0 | 0 | 0 |  | 18 | 5 | 32 |  | 35 | 0 | 75 |  | 5 | 1 | 8 |
|  |  | Self-inflicted injuries | 0 | 0 | 0 |  | 18 | 5 | 32 |  | 35 | 0 | 75 |  | 5 | 1 | 8 |
|  |  | Violence | 0 | 0 | 0 |  | 0 | 0 | 0 |  | 0 | 0 | 0 |  | 0 | 0 | 0 |
|  |  | Other intentional injuries | 0 | 0 | 0 |  | 0 | 0 | 0 |  | 0 | 0 | 0 |  | 0 | 0 | 0 |
| Men | |  |  |  |  |  |  |  |  |  |  |  |  |  |  |  |  |
| Injuries | |  | 0 | 0 | 0 |  | 1150 | 505 | 1796 |  | 1314 | 540 | 2088 |  | 501 | 183 | 818 |
|  | Unintentional injuries | | 0 | 0 | 0 |  | 688 | 317 | 1060 |  | 869 | 364 | 1374 |  | 379 | 140 | 619 |
|  |  | Transport injuries | 0 | 0 | 0 |  | 184 | 111 | 257 |  | 177 | 90 | 265 |  | 29 | 14 | 43 |
|  |  | Poisonings | 0 | 0 | 0 |  | 12 | 5 | 19 |  | 16 | 6 | 25 |  | 6 | 2 | 11 |
|  |  | Falls | 0 | 0 | 0 |  | 26 | 11 | 41 |  | 57 | 22 | 91 |  | 47 | 17 | 77 |
|  |  | Fires, heat and hot substances | 0 | 0 | 0 |  | 26 | 10 | 41 |  | 40 | 16 | 64 |  | 26 | 9 | 43 |
|  |  | Drownings | 0 | 0 | 0 |  | 86 | 35 | 136 |  | 78 | 31 | 126 |  | 16 | 6 | 27 |
|  |  | Other unintentional injuries | 0 | 0 | 0 |  | 356 | 145 | 566 |  | 501 | 198 | 803 |  | 255 | 91 | 418 |
|  | Intentional injuries | | 0 | 0 | 0 |  | 462 | 189 | 736 |  | 445 | 176 | 714 |  | 122 | 44 | 200 |
|  |  | Self-inflicted injuries | 0 | 0 | 0 |  | 462 | 188 | 735 |  | 445 | 176 | 714 |  | 122 | 44 | 200 |
|  |  | Violence | 0 | 0 | 0 |  | 0 | 0 | 0 |  | 0 | 0 | 0 |  | 0 | 0 | 0 |
|  |  | Other intentional injuries | 0 | 0 | 0 |  | 1 | 0 | 1 |  | 0 | 0 | 0 |  | 0 | 0 | 0 |

Table 14: Deaths from injuries attributable to alcohol consumption (without harms to others): Latin America Tropical

|  |  |  | 0 to 14 years of age | | |  | 15 to 34 years of age | | |  | 35 to 64 years of age | | |  | 65 years of age and older | | |
| --- | --- | --- | --- | --- | --- | --- | --- | --- | --- | --- | --- | --- | --- | --- | --- | --- | --- |
|  |  |  | Point estimate | Lower 95% CI | Upper 95% CI |  | Point estimate | Lower 95% CI | Upper 95% CI |  | Point estimate | Lower 95% CI | Upper 95% CI |  | Point estimate | Lower 95% CI | Upper 95% CI |
| Women | |  |  |  |  |  |  |  |  |  |  |  |  |  |  |  |  |
| Injuries | |  | 0 | 0 | 0 |  | 303 | 87 | 539 |  | 391 | 30 | 796 |  | 47 | 4 | 91 |
|  | Unintentional injuries | | 0 | 0 | 0 |  | 244 | 87 | 411 |  | 298 | 30 | 593 |  | 46 | 4 | 87 |
|  |  | Transport injuries | 0 | 0 | 0 |  | 172 | 87 | 258 |  | 140 | 30 | 250 |  | 7 | 3 | 11 |
|  |  | Poisonings | 0 | 0 | 0 |  | 2 | 0 | 4 |  | 2 | 0 | 5 |  | 0 | 0 | 0 |
|  |  | Falls | 0 | 0 | 0 |  | 6 | 0 | 14 |  | 33 | 0 | 72 |  | 22 | 0 | 44 |
|  |  | Fires, heat and hot substances | 0 | 0 | 0 |  | 9 | 0 | 20 |  | 18 | 0 | 40 |  | 1 | 0 | 3 |
|  |  | Drownings | 0 | 0 | 0 |  | 19 | 0 | 40 |  | 23 | 0 | 50 |  | 1 | 0 | 1 |
|  |  | Other unintentional injuries | 0 | 0 | 0 |  | 35 | 0 | 75 |  | 81 | 0 | 177 |  | 14 | 0 | 27 |
|  | Intentional injuries | | 0 | 0 | 0 |  | 60 | 0 | 128 |  | 94 | 0 | 203 |  | 2 | 0 | 4 |
|  |  | Self-inflicted injuries | 0 | 0 | 0 |  | 60 | 0 | 128 |  | 94 | 0 | 203 |  | 2 | 0 | 4 |
|  |  | Violence | 0 | 0 | 0 |  | 0 | 0 | 0 |  | 0 | 0 | 0 |  | 0 | 0 | 0 |
|  |  | Other intentional injuries | 0 | 0 | 0 |  | 0 | 0 | 0 |  | 0 | 0 | 0 |  | 0 | 0 | 0 |
| Men | |  |  |  |  |  |  |  |  |  |  |  |  |  |  |  |  |
| Injuries | |  | 0 | 0 | 0 |  | 6895 | 3024 | 10766 |  | 6594 | 2030 | 11159 |  | 356 | 104 | 609 |
|  | Unintentional injuries | | 0 | 0 | 0 |  | 5568 | 2502 | 8634 |  | 5344 | 1586 | 9103 |  | 304 | 90 | 517 |
|  |  | Transport injuries | 0 | 0 | 0 |  | 2843 | 1430 | 4256 |  | 2215 | 475 | 3955 |  | 54 | 25 | 82 |
|  |  | Poisonings | 0 | 0 | 0 |  | 26 | 10 | 42 |  | 30 | 11 | 49 |  | 1 | 0 | 2 |
|  |  | Falls | 0 | 0 | 0 |  | 351 | 138 | 564 |  | 878 | 312 | 1444 |  | 119 | 31 | 207 |
|  |  | Fires, heat and hot substances | 0 | 0 | 0 |  | 91 | 36 | 146 |  | 123 | 44 | 202 |  | 9 | 2 | 16 |
|  |  | Drownings | 0 | 0 | 0 |  | 1018 | 401 | 1636 |  | 713 | 253 | 1172 |  | 18 | 5 | 31 |
|  |  | Other unintentional injuries | 0 | 0 | 0 |  | 1238 | 487 | 1989 |  | 1386 | 492 | 2280 |  | 103 | 27 | 179 |
|  | Intentional injuries | | 0 | 0 | 0 |  | 1327 | 522 | 2132 |  | 1250 | 444 | 2056 |  | 53 | 14 | 92 |
|  |  | Self-inflicted injuries | 0 | 0 | 0 |  | 1135 | 447 | 1824 |  | 1232 | 437 | 2027 |  | 53 | 14 | 92 |
|  |  | Violence | 0 | 0 | 0 |  | 0 | 0 | 0 |  | 0 | 0 | 0 |  | 0 | 0 | 0 |
|  |  | Other intentional injuries | 0 | 0 | 0 |  | 192 | 76 | 309 |  | 18 | 6 | 30 |  | 0 | 0 | 0 |

Table 15: Deaths from injuries attributable to alcohol consumption (without harms to others): North Africa Middle East

|  |  |  | 0 to 14 years of age | | |  | 15 to 34 years of age | | |  | 35 to 64 years of age | | |  | 65 years of age and older | | |
| --- | --- | --- | --- | --- | --- | --- | --- | --- | --- | --- | --- | --- | --- | --- | --- | --- | --- |
|  |  |  | Point estimate | Lower 95% CI | Upper 95% CI |  | Point estimate | Lower 95% CI | Upper 95% CI |  | Point estimate | Lower 95% CI | Upper 95% CI |  | Point estimate | Lower 95% CI | Upper 95% CI |
| Women | |  |  |  |  |  |  |  |  |  |  |  |  |  |  |  |  |
| Injuries | |  | 0 | 0 | 0 |  | 95 | 0 | 593 |  | 28 | 0 | 326 |  | 3 | 0 | 76 |
|  | Unintentional injuries | | 0 | 0 | 0 |  | 74 | 0 | 453 |  | 24 | 0 | 261 |  | 3 | 0 | 70 |
|  |  | Transport injuries | 0 | 0 | 0 |  | 34 | 0 | 189 |  | 8 | 0 | 24 |  | 0 | 0 | 1 |
|  |  | Poisonings | 0 | 0 | 0 |  | 5 | 0 | 31 |  | 2 | 0 | 23 |  | 0 | 0 | 5 |
|  |  | Falls | 0 | 0 | 0 |  | 2 | 0 | 15 |  | 3 | 0 | 44 |  | 1 | 0 | 25 |
|  |  | Fires, heat and hot substances | 0 | 0 | 0 |  | 10 | 0 | 68 |  | 3 | 0 | 41 |  | 0 | 0 | 8 |
|  |  | Drownings | 0 | 0 | 0 |  | 4 | 0 | 26 |  | 1 | 0 | 14 |  | 0 | 0 | 2 |
|  |  | Other unintentional injuries | 0 | 0 | 0 |  | 19 | 0 | 124 |  | 8 | 0 | 115 |  | 1 | 0 | 29 |
|  | Intentional injuries | | 0 | 0 | 0 |  | 21 | 0 | 141 |  | 4 | 0 | 65 |  | 0 | 0 | 6 |
|  |  | Self-inflicted injuries | 0 | 0 | 0 |  | 21 | 0 | 137 |  | 4 | 0 | 63 |  | 0 | 0 | 5 |
|  |  | Violence | 0 | 0 | 0 |  | 0 | 0 | 0 |  | 0 | 0 | 0 |  | 0 | 0 | 0 |
|  |  | Other intentional injuries | 0 | 0 | 0 |  | 1 | 0 | 3 |  | 0 | 0 | 1 |  | 0 | 0 | 1 |
| Men | |  |  |  |  |  |  |  |  |  |  |  |  |  |  |  |  |
| Injuries | |  | 0 | 0 | 0 |  | 4296 | 56 | 13830 |  | 1016 | 0 | 2387 |  | 15 | 3 | 27 |
|  | Unintentional injuries | | 0 | 0 | 0 |  | 3705 | 44 | 12661 |  | 879 | 0 | 2093 |  | 15 | 3 | 26 |
|  |  | Transport injuries | 0 | 0 | 0 |  | 1512 | 0 | 8318 |  | 240 | 0 | 722 |  | 8 | 2 | 15 |
|  |  | Poisonings | 0 | 0 | 0 |  | 223 | 4 | 441 |  | 96 | 0 | 206 |  | 0 | 0 | 1 |
|  |  | Falls | 0 | 0 | 0 |  | 214 | 4 | 423 |  | 128 | 0 | 275 |  | 2 | 0 | 4 |
|  |  | Fires, heat and hot substances | 0 | 0 | 0 |  | 150 | 3 | 297 |  | 32 | 0 | 68 |  | 0 | 0 | 1 |
|  |  | Drownings | 0 | 0 | 0 |  | 512 | 10 | 1013 |  | 62 | 0 | 134 |  | 0 | 0 | 1 |
|  |  | Other unintentional injuries | 0 | 0 | 0 |  | 1095 | 22 | 2168 |  | 321 | 0 | 689 |  | 3 | 1 | 6 |
|  | Intentional injuries | | 0 | 0 | 0 |  | 590 | 12 | 1169 |  | 137 | 0 | 294 |  | 1 | 0 | 1 |
|  |  | Self-inflicted injuries | 0 | 0 | 0 |  | 518 | 10 | 1025 |  | 118 | 0 | 252 |  | 1 | 0 | 1 |
|  |  | Violence | 0 | 0 | 0 |  | 0 | 0 | 0 |  | 0 | 0 | 0 |  | 0 | 0 | 0 |
|  |  | Other intentional injuries | 0 | 0 | 0 |  | 73 | 1 | 144 |  | 20 | 0 | 42 |  | 0 | 0 | 0 |

Table 16: Deaths from injuries attributable to alcohol consumption (without harms to others): North America [high Income]

|  |  |  | 0 to 14 years of age | | |  | 15 to 34 years of age | | |  | 35 to 64 years of age | | |  | 65 years of age and older | | |
| --- | --- | --- | --- | --- | --- | --- | --- | --- | --- | --- | --- | --- | --- | --- | --- | --- | --- |
|  |  |  | Point estimate | Lower 95% CI | Upper 95% CI |  | Point estimate | Lower 95% CI | Upper 95% CI |  | Point estimate | Lower 95% CI | Upper 95% CI |  | Point estimate | Lower 95% CI | Upper 95% CI |
| Women | |  |  |  |  |  |  |  |  |  |  |  |  |  |  |  |  |
| Injuries | |  | 0 | 0 | 0 |  | 701 | 254 | 1148 |  | 877 | 315 | 1439 |  | 328 | 122 | 535 |
|  | Unintentional injuries | | 0 | 0 | 0 |  | 531 | 205 | 857 |  | 603 | 221 | 984 |  | 313 | 116 | 510 |
|  |  | Transport injuries | 0 | 0 | 0 |  | 308 | 140 | 475 |  | 135 | 62 | 209 |  | 29 | 14 | 44 |
|  |  | Poisonings | 0 | 0 | 0 |  | 158 | 46 | 270 |  | 282 | 96 | 467 |  | 9 | 3 | 15 |
|  |  | Falls | 0 | 0 | 0 |  | 10 | 3 | 18 |  | 53 | 18 | 87 |  | 141 | 50 | 231 |
|  |  | Fires, heat and hot substances | 0 | 0 | 0 |  | 14 | 4 | 25 |  | 26 | 9 | 43 |  | 9 | 3 | 15 |
|  |  | Drownings | 0 | 0 | 0 |  | 10 | 3 | 17 |  | 14 | 5 | 23 |  | 2 | 1 | 4 |
|  |  | Other unintentional injuries | 0 | 0 | 0 |  | 31 | 9 | 53 |  | 94 | 32 | 155 |  | 123 | 44 | 202 |
|  | Intentional injuries | | 0 | 0 | 0 |  | 170 | 49 | 291 |  | 274 | 93 | 454 |  | 15 | 6 | 25 |
|  |  | Self-inflicted injuries | 0 | 0 | 0 |  | 170 | 49 | 291 |  | 274 | 93 | 454 |  | 15 | 6 | 25 |
|  |  | Violence | 0 | 0 | 0 |  | 0 | 0 | 0 |  | 0 | 0 | 0 |  | 0 | 0 | 0 |
|  |  | Other intentional injuries | 0 | 0 | 0 |  | 0 | 0 | 0 |  | 0 | 0 | 1 |  | 0 | 0 | 0 |
| Men | |  |  |  |  |  |  |  |  |  |  |  |  |  |  |  |  |
| Injuries | |  | 0 | 0 | 0 |  | 9218 | 4242 | 14195 |  | 8606 | 3719 | 13493 |  | 2332 | 887 | 3777 |
|  | Unintentional injuries | | 0 | 0 | 0 |  | 5745 | 2638 | 8852 |  | 5144 | 2233 | 8056 |  | 1798 | 687 | 2910 |
|  |  | Transport injuries | 0 | 0 | 0 |  | 2243 | 1021 | 3466 |  | 885 | 404 | 1366 |  | 113 | 56 | 170 |
|  |  | Poisonings | 0 | 0 | 0 |  | 1932 | 892 | 2972 |  | 2066 | 887 | 3245 |  | 58 | 22 | 94 |
|  |  | Falls | 0 | 0 | 0 |  | 238 | 110 | 366 |  | 622 | 267 | 977 |  | 815 | 305 | 1324 |
|  |  | Fires, heat and hot substances | 0 | 0 | 0 |  | 120 | 55 | 184 |  | 195 | 84 | 307 |  | 64 | 24 | 103 |
|  |  | Drownings | 0 | 0 | 0 |  | 364 | 168 | 559 |  | 237 | 102 | 373 |  | 36 | 14 | 59 |
|  |  | Other unintentional injuries | 0 | 0 | 0 |  | 848 | 391 | 1304 |  | 1139 | 489 | 1789 |  | 713 | 267 | 1159 |
|  | Intentional injuries | | 0 | 0 | 0 |  | 3473 | 1604 | 5343 |  | 3462 | 1486 | 5438 |  | 534 | 200 | 867 |
|  |  | Self-inflicted injuries | 0 | 0 | 0 |  | 3404 | 1572 | 5236 |  | 3421 | 1469 | 5373 |  | 532 | 199 | 865 |
|  |  | Violence | 0 | 0 | 0 |  | 0 | 0 | 0 |  | 0 | 0 | 0 |  | 0 | 0 | 0 |
|  |  | Other intentional injuries | 0 | 0 | 0 |  | 70 | 32 | 107 |  | 41 | 18 | 65 |  | 1 | 0 | 2 |

Table 17: Deaths from injuries attributable to alcohol consumption (without harms to others): Oceania

|  |  |  | 0 to 14 years of age | | |  | 15 to 34 years of age | | |  | 35 to 64 years of age | | |  | 65 years of age and older | | |
| --- | --- | --- | --- | --- | --- | --- | --- | --- | --- | --- | --- | --- | --- | --- | --- | --- | --- |
|  |  |  | Point estimate | Lower 95% CI | Upper 95% CI |  | Point estimate | Lower 95% CI | Upper 95% CI |  | Point estimate | Lower 95% CI | Upper 95% CI |  | Point estimate | Lower 95% CI | Upper 95% CI |
| Women | |  |  |  |  |  |  |  |  |  |  |  |  |  |  |  |  |
| Injuries | |  | 0 | 0 | 0 |  | 23 | 9 | 37 |  | 10 | 3 | 17 |  | 2 | 0 | 3 |
|  | Unintentional injuries | | 0 | 0 | 0 |  | 17 | 7 | 27 |  | 8 | 3 | 13 |  | 1 | 0 | 2 |
|  |  | Transport injuries | 0 | 0 | 0 |  | 2 | 1 | 3 |  | 2 | 1 | 3 |  | 0 | 0 | 0 |
|  |  | Poisonings | 0 | 0 | 0 |  | 1 | 0 | 2 |  | 1 | 0 | 2 |  | 0 | 0 | 0 |
|  |  | Falls | 0 | 0 | 0 |  | 1 | 0 | 1 |  | 1 | 0 | 1 |  | 0 | 0 | 1 |
|  |  | Fires, heat and hot substances | 0 | 0 | 0 |  | 7 | 3 | 12 |  | 2 | 1 | 4 |  | 0 | 0 | 0 |
|  |  | Drownings | 0 | 0 | 0 |  | 2 | 1 | 3 |  | 1 | 0 | 1 |  | 0 | 0 | 0 |
|  |  | Other unintentional injuries | 0 | 0 | 0 |  | 5 | 2 | 8 |  | 2 | 0 | 3 |  | 0 | 0 | 1 |
|  | Intentional injuries | | 0 | 0 | 0 |  | 6 | 2 | 10 |  | 2 | 0 | 3 |  | 0 | 0 | 0 |
|  |  | Self-inflicted injuries | 0 | 0 | 0 |  | 6 | 2 | 9 |  | 2 | 0 | 3 |  | 0 | 0 | 0 |
|  |  | Violence | 0 | 0 | 0 |  | 0 | 0 | 0 |  | 0 | 0 | 0 |  | 0 | 0 | 0 |
|  |  | Other intentional injuries | 0 | 0 | 0 |  | 0 | 0 | 0 |  | 0 | 0 | 0 |  | 0 | 0 | 0 |
| Men | |  |  |  |  |  |  |  |  |  |  |  |  |  |  |  |  |
| Injuries | |  | 0 | 0 | 0 |  | 148 | 79 | 217 |  | 107 | 56 | 158 |  | 12 | 6 | 18 |
|  | Unintentional injuries | | 0 | 0 | 0 |  | 122 | 67 | 178 |  | 91 | 49 | 134 |  | 10 | 5 | 15 |
|  |  | Transport injuries | 0 | 0 | 0 |  | 55 | 35 | 74 |  | 39 | 24 | 53 |  | 3 | 2 | 5 |
|  |  | Poisonings | 0 | 0 | 0 |  | 7 | 3 | 10 |  | 9 | 4 | 14 |  | 1 | 0 | 1 |
|  |  | Falls | 0 | 0 | 0 |  | 5 | 2 | 8 |  | 7 | 3 | 10 |  | 3 | 1 | 4 |
|  |  | Fires, heat and hot substances | 0 | 0 | 0 |  | 7 | 3 | 11 |  | 6 | 3 | 9 |  | 0 | 0 | 1 |
|  |  | Drownings | 0 | 0 | 0 |  | 14 | 7 | 22 |  | 8 | 4 | 12 |  | 1 | 0 | 1 |
|  |  | Other unintentional injuries | 0 | 0 | 0 |  | 35 | 16 | 53 |  | 23 | 11 | 36 |  | 2 | 1 | 3 |
|  | Intentional injuries | | 0 | 0 | 0 |  | 26 | 12 | 39 |  | 16 | 7 | 24 |  | 2 | 1 | 2 |
|  |  | Self-inflicted injuries | 0 | 0 | 0 |  | 25 | 12 | 38 |  | 15 | 7 | 23 |  | 2 | 1 | 2 |
|  |  | Violence | 0 | 0 | 0 |  | 0 | 0 | 0 |  | 0 | 0 | 0 |  | 0 | 0 | 0 |
|  |  | Other intentional injuries | 0 | 0 | 0 |  | 1 | 0 | 2 |  | 1 | 0 | 1 |  | 0 | 0 | 0 |

Table 18: Deaths from injuries attributable to alcohol consumption (without harms to others): Sub-Saharan Africa Central

|  |  |  | 0 to 14 years of age | | |  | 15 to 34 years of age | | |  | 35 to 64 years of age | | |  | 65 years of age and older | | |
| --- | --- | --- | --- | --- | --- | --- | --- | --- | --- | --- | --- | --- | --- | --- | --- | --- | --- |
|  |  |  | Point estimate | Lower 95% CI | Upper 95% CI |  | Point estimate | Lower 95% CI | Upper 95% CI |  | Point estimate | Lower 95% CI | Upper 95% CI |  | Point estimate | Lower 95% CI | Upper 95% CI |
| Women | |  |  |  |  |  |  |  |  |  |  |  |  |  |  |  |  |
| Injuries | |  | 0 | 0 | 0 |  | 191 | 85 | 297 |  | 190 | 70 | 310 |  | 23 | 9 | 37 |
|  | Unintentional injuries | | 0 | 0 | 0 |  | 156 | 73 | 240 |  | 162 | 65 | 258 |  | 19 | 8 | 31 |
|  |  | Transport injuries | 0 | 0 | 0 |  | 67 | 42 | 92 |  | 76 | 48 | 104 |  | 8 | 5 | 12 |
|  |  | Poisonings | 0 | 0 | 0 |  | 24 | 8 | 40 |  | 24 | 5 | 43 |  | 3 | 1 | 6 |
|  |  | Falls | 0 | 0 | 0 |  | 3 | 1 | 4 |  | 5 | 1 | 9 |  | 3 | 1 | 4 |
|  |  | Fires, heat and hot substances | 0 | 0 | 0 |  | 9 | 3 | 15 |  | 11 | 2 | 19 |  | 2 | 0 | 3 |
|  |  | Drownings | 0 | 0 | 0 |  | 27 | 9 | 45 |  | 16 | 3 | 29 |  | 1 | 0 | 1 |
|  |  | Other unintentional injuries | 0 | 0 | 0 |  | 26 | 9 | 43 |  | 30 | 6 | 54 |  | 3 | 1 | 5 |
|  | Intentional injuries | | 0 | 0 | 0 |  | 35 | 12 | 58 |  | 29 | 6 | 52 |  | 3 | 1 | 6 |
|  |  | Self-inflicted injuries | 0 | 0 | 0 |  | 35 | 12 | 58 |  | 29 | 6 | 52 |  | 3 | 1 | 6 |
|  |  | Violence | 0 | 0 | 0 |  | 0 | 0 | 0 |  | 0 | 0 | 0 |  | 0 | 0 | 0 |
|  |  | Other intentional injuries | 0 | 0 | 0 |  | 0 | 0 | 0 |  | 0 | 0 | 0 |  | 0 | 0 | 0 |
| Men | |  |  |  |  |  |  |  |  |  |  |  |  |  |  |  |  |
| Injuries | |  | 0 | 0 | 0 |  | 2440 | 1234 | 3646 |  | 2367 | 1201 | 3532 |  | 97 | 47 | 147 |
|  | Unintentional injuries | | 0 | 0 | 0 |  | 2166 | 1114 | 3218 |  | 2092 | 1086 | 3098 |  | 82 | 41 | 124 |
|  |  | Transport injuries | 0 | 0 | 0 |  | 855 | 536 | 1174 |  | 976 | 617 | 1336 |  | 39 | 22 | 55 |
|  |  | Poisonings | 0 | 0 | 0 |  | 160 | 71 | 250 |  | 185 | 78 | 293 |  | 2 | 1 | 4 |
|  |  | Falls | 0 | 0 | 0 |  | 38 | 17 | 60 |  | 73 | 31 | 115 |  | 9 | 4 | 14 |
|  |  | Fires, heat and hot substances | 0 | 0 | 0 |  | 26 | 12 | 41 |  | 41 | 17 | 65 |  | 4 | 2 | 6 |
|  |  | Drownings | 0 | 0 | 0 |  | 303 | 133 | 473 |  | 405 | 170 | 640 |  | 3 | 1 | 5 |
|  |  | Other unintentional injuries | 0 | 0 | 0 |  | 783 | 345 | 1220 |  | 411 | 173 | 649 |  | 26 | 11 | 40 |
|  | Intentional injuries | | 0 | 0 | 0 |  | 274 | 121 | 428 |  | 275 | 116 | 434 |  | 15 | 6 | 23 |
|  |  | Self-inflicted injuries | 0 | 0 | 0 |  | 274 | 121 | 428 |  | 275 | 116 | 434 |  | 15 | 6 | 23 |
|  |  | Violence | 0 | 0 | 0 |  | 0 | 0 | 0 |  | 0 | 0 | 0 |  | 0 | 0 | 0 |
|  |  | Other intentional injuries | 0 | 0 | 0 |  | 0 | 0 | 0 |  | 0 | 0 | 0 |  | 0 | 0 | 0 |

Table 19: Deaths from injuries attributable to alcohol consumption (without harms to others): Sub-Saharan Africa East

|  |  |  | 0 to 14 years of age | | |  | 15 to 34 years of age | | |  | 35 to 64 years of age | | |  | 65 years of age and older | | |
| --- | --- | --- | --- | --- | --- | --- | --- | --- | --- | --- | --- | --- | --- | --- | --- | --- | --- |
|  |  |  | Point estimate | Lower 95% CI | Upper 95% CI |  | Point estimate | Lower 95% CI | Upper 95% CI |  | Point estimate | Lower 95% CI | Upper 95% CI |  | Point estimate | Lower 95% CI | Upper 95% CI |
| Women | |  |  |  |  |  |  |  |  |  |  |  |  |  |  |  |  |
| Injuries | |  | 0 | 0 | 0 |  | 310 | 39 | 726 |  | 921 | 0 | 2161 |  | 139 | 14 | 319 |
|  | Unintentional injuries | | 0 | 0 | 0 |  | 245 | 39 | 554 |  | 760 | 0 | 1756 |  | 115 | 14 | 258 |
|  |  | Transport injuries | 0 | 0 | 0 |  | 93 | 39 | 147 |  | 317 | 0 | 649 |  | 33 | 14 | 53 |
|  |  | Poisonings | 0 | 0 | 0 |  | 31 | 0 | 82 |  | 97 | 0 | 243 |  | 19 | 0 | 47 |
|  |  | Falls | 0 | 0 | 0 |  | 7 | 0 | 19 |  | 31 | 0 | 77 |  | 19 | 0 | 47 |
|  |  | Fires, heat and hot substances | 0 | 0 | 0 |  | 18 | 0 | 49 |  | 56 | 0 | 140 |  | 12 | 0 | 31 |
|  |  | Drownings | 0 | 0 | 0 |  | 30 | 0 | 81 |  | 63 | 0 | 157 |  | 4 | 0 | 10 |
|  |  | Other unintentional injuries | 0 | 0 | 0 |  | 65 | 0 | 174 |  | 195 | 0 | 489 |  | 28 | 0 | 70 |
|  | Intentional injuries | | 0 | 0 | 0 |  | 64 | 0 | 172 |  | 162 | 0 | 404 |  | 24 | 0 | 61 |
|  |  | Self-inflicted injuries | 0 | 0 | 0 |  | 64 | 0 | 171 |  | 161 | 0 | 404 |  | 24 | 0 | 61 |
|  |  | Violence | 0 | 0 | 0 |  | 0 | 0 | 0 |  | 0 | 0 | 0 |  | 0 | 0 | 0 |
|  |  | Other intentional injuries | 0 | 0 | 0 |  | 0 | 0 | 1 |  | 0 | 0 | 1 |  | 0 | 0 | 0 |
| Men | |  |  |  |  |  |  |  |  |  |  |  |  |  |  |  |  |
| Injuries | |  | 0 | 0 | 0 |  | 4759 | 1387 | 8131 |  | 12266 | 2635 | 22051 |  | 1036 | 285 | 1786 |
|  | Unintentional injuries | | 0 | 0 | 0 |  | 4061 | 1209 | 6914 |  | 10268 | 2043 | 18647 |  | 813 | 231 | 1395 |
|  |  | Transport injuries | 0 | 0 | 0 |  | 1035 | 434 | 1636 |  | 3375 | 0 | 6905 |  | 197 | 81 | 314 |
|  |  | Poisonings | 0 | 0 | 0 |  | 352 | 90 | 614 |  | 1095 | 324 | 1865 |  | 24 | 6 | 43 |
|  |  | Falls | 0 | 0 | 0 |  | 126 | 32 | 220 |  | 582 | 173 | 992 |  | 131 | 32 | 230 |
|  |  | Fires, heat and hot substances | 0 | 0 | 0 |  | 89 | 23 | 155 |  | 308 | 91 | 525 |  | 56 | 14 | 98 |
|  |  | Drownings | 0 | 0 | 0 |  | 651 | 167 | 1136 |  | 1792 | 531 | 3053 |  | 33 | 8 | 58 |
|  |  | Other unintentional injuries | 0 | 0 | 0 |  | 1808 | 463 | 3154 |  | 3115 | 923 | 5307 |  | 371 | 90 | 653 |
|  | Intentional injuries | | 0 | 0 | 0 |  | 698 | 179 | 1217 |  | 1998 | 592 | 3404 |  | 223 | 54 | 391 |
|  |  | Self-inflicted injuries | 0 | 0 | 0 |  | 688 | 176 | 1200 |  | 1981 | 587 | 3374 |  | 220 | 53 | 387 |
|  |  | Violence | 0 | 0 | 0 |  | 0 | 0 | 0 |  | 0 | 0 | 0 |  | 0 | 0 | 0 |
|  |  | Other intentional injuries | 0 | 0 | 0 |  | 10 | 3 | 18 |  | 17 | 5 | 30 |  | 3 | 1 | 5 |

Table 20: Deaths from injuries attributable to alcohol consumption (without harms to others): Sub-Saharan Africa Southern

|  |  |  | 0 to 14 years of age | | |  | 15 to 34 years of age | | |  | 35 to 64 years of age | | |  | 65 years of age and older | | |
| --- | --- | --- | --- | --- | --- | --- | --- | --- | --- | --- | --- | --- | --- | --- | --- | --- | --- |
|  |  |  | Point estimate | Lower 95% CI | Upper 95% CI |  | Point estimate | Lower 95% CI | Upper 95% CI |  | Point estimate | Lower 95% CI | Upper 95% CI |  | Point estimate | Lower 95% CI | Upper 95% CI |
| Women | |  |  |  |  |  |  |  |  |  |  |  |  |  |  |  |  |
| Injuries | |  | 0 | 0 | 0 |  | 535 | 116 | 1042 |  | 592 | 131 | 1143 |  | 47 | 6 | 93 |
|  | Unintentional injuries | | 0 | 0 | 0 |  | 426 | 116 | 791 |  | 499 | 131 | 925 |  | 41 | 6 | 80 |
|  |  | Transport injuries | 0 | 0 | 0 |  | 242 | 116 | 369 |  | 308 | 131 | 486 |  | 14 | 6 | 21 |
|  |  | Poisonings | 0 | 0 | 0 |  | 38 | 0 | 87 |  | 27 | 0 | 63 |  | 3 | 0 | 7 |
|  |  | Falls | 0 | 0 | 0 |  | 6 | 0 | 14 |  | 14 | 0 | 31 |  | 6 | 0 | 13 |
|  |  | Fires, heat and hot substances | 0 | 0 | 0 |  | 73 | 0 | 168 |  | 76 | 0 | 175 |  | 8 | 0 | 17 |
|  |  | Drownings | 0 | 0 | 0 |  | 28 | 0 | 65 |  | 15 | 0 | 35 |  | 0 | 0 | 1 |
|  |  | Other unintentional injuries | 0 | 0 | 0 |  | 38 | 0 | 88 |  | 58 | 0 | 135 |  | 10 | 0 | 21 |
|  | Intentional injuries | | 0 | 0 | 0 |  | 109 | 0 | 252 |  | 94 | 0 | 217 |  | 6 | 0 | 13 |
|  |  | Self-inflicted injuries | 0 | 0 | 0 |  | 108 | 0 | 248 |  | 93 | 0 | 214 |  | 6 | 0 | 13 |
|  |  | Violence | 0 | 0 | 0 |  | 0 | 0 | 0 |  | 0 | 0 | 0 |  | 0 | 0 | 0 |
|  |  | Other intentional injuries | 0 | 0 | 0 |  | 1 | 0 | 3 |  | 1 | 0 | 3 |  | 0 | 0 | 0 |
| Men | |  |  |  |  |  |  |  |  |  |  |  |  |  |  |  |  |
| Injuries | |  | 0 | 0 | 0 |  | 7439 | 3353 | 11525 |  | 6942 | 2920 | 10964 |  | 263 | 93 | 433 |
|  | Unintentional injuries | | 0 | 0 | 0 |  | 5924 | 2710 | 9137 |  | 5609 | 2365 | 8854 |  | 199 | 74 | 324 |
|  |  | Transport injuries | 0 | 0 | 0 |  | 3651 | 1746 | 5556 |  | 3582 | 1522 | 5643 |  | 76 | 36 | 117 |
|  |  | Poisonings | 0 | 0 | 0 |  | 240 | 102 | 378 |  | 199 | 83 | 315 |  | 3 | 1 | 5 |
|  |  | Falls | 0 | 0 | 0 |  | 129 | 55 | 204 |  | 223 | 93 | 353 |  | 30 | 9 | 50 |
|  |  | Fires, heat and hot substances | 0 | 0 | 0 |  | 412 | 175 | 649 |  | 483 | 201 | 766 |  | 22 | 7 | 37 |
|  |  | Drownings | 0 | 0 | 0 |  | 578 | 245 | 911 |  | 412 | 171 | 652 |  | 4 | 1 | 7 |
|  |  | Other unintentional injuries | 0 | 0 | 0 |  | 914 | 388 | 1440 |  | 710 | 295 | 1125 |  | 64 | 19 | 108 |
|  | Intentional injuries | | 0 | 0 | 0 |  | 1515 | 643 | 2387 |  | 1333 | 555 | 2111 |  | 64 | 20 | 109 |
|  |  | Self-inflicted injuries | 0 | 0 | 0 |  | 1483 | 629 | 2337 |  | 1293 | 538 | 2047 |  | 63 | 19 | 107 |
|  |  | Violence | 0 | 0 | 0 |  | 0 | 0 | 0 |  | 0 | 0 | 0 |  | 0 | 0 | 0 |
|  |  | Other intentional injuries | 0 | 0 | 0 |  | 32 | 13 | 50 |  | 40 | 17 | 63 |  | 1 | 0 | 2 |

Table 21: Deaths from injuries attributable to alcohol consumption (without harms to others): Sub-Saharan Africa West

|  |  |  | 0 to 14 years of age | | |  | 15 to 34 years of age | | |  | 35 to 64 years of age | | |  | 65 years of age and older | | |
| --- | --- | --- | --- | --- | --- | --- | --- | --- | --- | --- | --- | --- | --- | --- | --- | --- | --- |
|  |  |  | Point estimate | Lower 95% CI | Upper 95% CI |  | Point estimate | Lower 95% CI | Upper 95% CI |  | Point estimate | Lower 95% CI | Upper 95% CI |  | Point estimate | Lower 95% CI | Upper 95% CI |
| Women | |  |  |  |  |  |  |  |  |  |  |  |  |  |  |  |  |
| Injuries | |  | 0 | 0 | 0 |  | 588 | 47 | 1129 |  | 1505 | 150 | 2861 |  | 221 | 64 | 379 |
|  | Unintentional injuries | | 0 | 0 | 0 |  | 459 | 45 | 874 |  | 1259 | 125 | 2394 |  | 191 | 56 | 325 |
|  |  | Transport injuries | 0 | 0 | 0 |  | 103 | 37 | 168 |  | 265 | 24 | 507 |  | 27 | 17 | 37 |
|  |  | Poisonings | 0 | 0 | 0 |  | 88 | 2 | 174 |  | 244 | 25 | 463 |  | 44 | 10 | 77 |
|  |  | Falls | 0 | 0 | 0 |  | 10 | 0 | 20 |  | 61 | 6 | 116 |  | 42 | 10 | 73 |
|  |  | Fires, heat and hot substances | 0 | 0 | 0 |  | 38 | 1 | 75 |  | 127 | 13 | 242 |  | 29 | 7 | 50 |
|  |  | Drownings | 0 | 0 | 0 |  | 102 | 2 | 201 |  | 188 | 19 | 357 |  | 8 | 2 | 14 |
|  |  | Other unintentional injuries | 0 | 0 | 0 |  | 119 | 3 | 235 |  | 373 | 38 | 709 |  | 42 | 10 | 75 |
|  | Intentional injuries | | 0 | 0 | 0 |  | 129 | 3 | 255 |  | 246 | 25 | 468 |  | 30 | 7 | 53 |
|  |  | Self-inflicted injuries | 0 | 0 | 0 |  | 129 | 3 | 255 |  | 246 | 25 | 468 |  | 30 | 7 | 53 |
|  |  | Violence | 0 | 0 | 0 |  | 0 | 0 | 0 |  | 0 | 0 | 0 |  | 0 | 0 | 0 |
|  |  | Other intentional injuries | 0 | 0 | 0 |  | 0 | 0 | 0 |  | 0 | 0 | 0 |  | 0 | 0 | 0 |
| Men | |  |  |  |  |  |  |  |  |  |  |  |  |  |  |  |  |
| Injuries | |  | 0 | 0 | 0 |  | 5833 | 2037 | 9629 |  | 12634 | 3878 | 21389 |  | 783 | 330 | 1235 |
|  | Unintentional injuries | | 0 | 0 | 0 |  | 5036 | 1761 | 8312 |  | 11000 | 3254 | 18746 |  | 653 | 283 | 1022 |
|  |  | Transport injuries | 0 | 0 | 0 |  | 1046 | 378 | 1714 |  | 3240 | 290 | 6190 |  | 170 | 108 | 232 |
|  |  | Poisonings | 0 | 0 | 0 |  | 486 | 168 | 803 |  | 1167 | 446 | 1888 |  | 19 | 7 | 31 |
|  |  | Falls | 0 | 0 | 0 |  | 141 | 49 | 232 |  | 667 | 255 | 1079 |  | 120 | 44 | 197 |
|  |  | Fires, heat and hot substances | 0 | 0 | 0 |  | 98 | 34 | 162 |  | 372 | 142 | 602 |  | 52 | 19 | 85 |
|  |  | Drownings | 0 | 0 | 0 |  | 1012 | 351 | 1674 |  | 2753 | 1052 | 4455 |  | 26 | 9 | 42 |
|  |  | Other unintentional injuries | 0 | 0 | 0 |  | 2253 | 781 | 3726 |  | 2801 | 1070 | 4532 |  | 265 | 96 | 434 |
|  | Intentional injuries | | 0 | 0 | 0 |  | 797 | 276 | 1317 |  | 1634 | 624 | 2644 |  | 130 | 47 | 213 |
|  |  | Self-inflicted injuries | 0 | 0 | 0 |  | 797 | 276 | 1317 |  | 1634 | 624 | 2644 |  | 130 | 47 | 213 |
|  |  | Violence | 0 | 0 | 0 |  | 0 | 0 | 0 |  | 0 | 0 | 0 |  | 0 | 0 | 0 |
|  |  | Other intentional injuries | 0 | 0 | 0 |  | 0 | 0 | 0 |  | 0 | 0 | 0 |  | 0 | 0 | 0 |
