# Supplementary material for: Global burden of injuries attributable to alcohol consumption in 2004: a novel way of calculating the burden of injuries attributable to alcohol consumption
Source: Popul Health Metr. 2012 May 18;10:9. doi: 10.1186/1478-7954-10-9 (PMC3463441; doi:10.1186/1478-7954-10-9)
Supplement: Additional file 4 — Deaths from injuries attributable to alcohol consumption. [file 1478-7954-10-9-S4.docx]

Appendix 4: Deaths from injuries attributable to alcohol consumption (with harms to others)

Table 1: Deaths from injuries attributable to alcohol consumption (with harms to others): Asia Pacific [high income]

|  |  |  | 0 to 14 years of age | | |  | 15 to 34 years of age | | |  | 35 to 64 years of age | | |  | 65 years of age and older | | |
| --- | --- | --- | --- | --- | --- | --- | --- | --- | --- | --- | --- | --- | --- | --- | --- | --- | --- |
|  |  |  | Point estimate | Lower 95% CI | Upper 95% CI |  | Point estimate | Lower 95% CI | Upper 95% CI |  | Point estimate | Lower 95% CI | Upper 95% CI |  | Point estimate | Lower 95% CI | Upper 95% CI |
| Women | |  |  |  |  |  |  |  |  |  |  |  |  |  |  |  |  |
| Injuries | |  | 23 | 9 | 36 |  | 289 | 125 | 453 |  | 776 | 307 | 1245 |  | 441 | 176 | 706 |
|  | Unintentional injuries | | 12 | 3 | 21 |  | 121 | 43 | 199 |  | 345 | 128 | 563 |  | 343 | 131 | 555 |
|  |  | Transport injuries | 12 | 3 | 21 |  | 99 | 33 | 166 |  | 207 | 75 | 340 |  | 104 | 28 | 181 |
|  |  | Poisonings | 0 | 0 | 0 |  | 3 | 2 | 5 |  | 10 | 4 | 16 |  | 2 | 1 | 3 |
|  |  | Falls | 0 | 0 | 0 |  | 5 | 2 | 7 |  | 27 | 11 | 44 |  | 67 | 29 | 106 |
|  |  | Fires, heat and hot substances | 0 | 0 | 0 |  | 3 | 1 | 4 |  | 11 | 4 | 18 |  | 8 | 3 | 12 |
|  |  | Drownings | 0 | 0 | 0 |  | 4 | 2 | 7 |  | 29 | 11 | 47 |  | 40 | 17 | 62 |
|  |  | Other unintentional injuries | 0 | 0 | 0 |  | 6 | 3 | 10 |  | 61 | 24 | 98 |  | 122 | 53 | 191 |
|  | Intentional injuries | | 11 | 7 | 16 |  | 168 | 82 | 254 |  | 431 | 179 | 682 |  | 98 | 44 | 152 |
|  |  | Self-inflicted injuries | 0 | 0 | 0 |  | 115 | 50 | 179 |  | 371 | 143 | 599 |  | 86 | 37 | 134 |
|  |  | Violence | 11 | 7 | 16 |  | 53 | 32 | 75 |  | 59 | 36 | 83 |  | 12 | 7 | 17 |
|  |  | Other intentional injuries | 0 | 0 | 0 |  | 0 | 0 | 0 |  | 0 | 0 | 0 |  | 0 | 0 | 0 |
| Men |  |  |  |  |  |  |  |  |  |  |  |  |  |  |  |  |  |
| Injuries | |  | 24 | 22 | 27 |  | 1964 | 1053 | 2875 |  | 12059 | 5981 | 18137 |  | 2987 | 1287 | 4687 |
|  | Unintentional injuries | | 18 | 18 | 18 |  | 882 | 590 | 1173 |  | 4670 | 2451 | 6890 |  | 2031 | 901 | 3161 |
|  |  | Transport injuries | 18 | 18 | 18 |  | 539 | 447 | 631 |  | 1286 | 839 | 1734 |  | 259 | 190 | 329 |
|  |  | Poisonings | 0 | 0 | 0 |  | 28 | 12 | 44 |  | 162 | 77 | 247 |  | 19 | 8 | 31 |
|  |  | Falls | 0 | 0 | 0 |  | 80 | 33 | 126 |  | 941 | 448 | 1433 |  | 442 | 177 | 707 |
|  |  | Fires, heat and hot substances | 0 | 0 | 0 |  | 14 | 6 | 22 |  | 181 | 86 | 275 |  | 69 | 28 | 110 |
|  |  | Drownings | 0 | 0 | 0 |  | 82 | 34 | 130 |  | 489 | 233 | 745 |  | 254 | 102 | 405 |
|  |  | Other unintentional injuries | 0 | 0 | 0 |  | 139 | 58 | 220 |  | 1611 | 768 | 2455 |  | 988 | 397 | 1579 |
|  | Intentional injuries | | 7 | 4 | 9 |  | 1082 | 463 | 1702 |  | 7388 | 3530 | 11247 |  | 956 | 386 | 1526 |
|  |  | Self-inflicted injuries | 0 | 0 | 0 |  | 1025 | 429 | 1621 |  | 7291 | 3473 | 11109 |  | 945 | 379 | 1510 |
|  |  | Violence | 7 | 4 | 9 |  | 55 | 33 | 77 |  | 90 | 54 | 127 |  | 10 | 6 | 14 |
|  |  | Other intentional injuries | 0 | 0 | 0 |  | 2 | 1 | 3 |  | 7 | 4 | 11 |  | 1 | 0 | 2 |

Table 2: Deaths from injuries attributable to alcohol consumption (with harms to others): Asia Central

|  |  |  | 0 to 14 years of age | | |  | 15 to 34 years of age | | |  | 35 to 64 years of age | | |  | 65 years of age and older | | |
| --- | --- | --- | --- | --- | --- | --- | --- | --- | --- | --- | --- | --- | --- | --- | --- | --- | --- |
|  |  |  | Point estimate | Lower 95% CI | Upper 95% CI |  | Point estimate | Lower 95% CI | Upper 95% CI |  | Point estimate | Lower 95% CI | Upper 95% CI |  | Point estimate | Lower 95% CI | Upper 95% CI |
| Women | |  |  |  |  |  |  |  |  |  |  |  |  |  |  |  |  |
| Injuries | |  | 51 | 19 | 83 |  | 361 | 164 | 558 |  | 538 | 199 | 878 |  | 63 | 23 | 104 |
|  | Unintentional injuries | | 45 | 16 | 75 |  | 242 | 101 | 384 |  | 416 | 143 | 689 |  | 47 | 14 | 80 |
|  |  | Transport injuries | 45 | 16 | 75 |  | 196 | 95 | 296 |  | 237 | 117 | 357 |  | 36 | 14 | 58 |
|  |  | Poisonings | 0 | 0 | 0 |  | 9 | 1 | 17 |  | 44 | 6 | 81 |  | 2 | 0 | 5 |
|  |  | Falls | 0 | 0 | 0 |  | 2 | 0 | 5 |  | 10 | 1 | 19 |  | 1 | 0 | 1 |
|  |  | Fires, heat and hot substances | 0 | 0 | 0 |  | 8 | 1 | 16 |  | 17 | 2 | 32 |  | 1 | 0 | 3 |
|  |  | Drownings | 0 | 0 | 0 |  | 7 | 1 | 12 |  | 13 | 2 | 24 |  | 0 | 0 | 1 |
|  |  | Other unintentional injuries | 0 | 0 | 0 |  | 20 | 2 | 38 |  | 95 | 13 | 176 |  | 6 | 0 | 12 |
|  | Intentional injuries | | 6 | 4 | 8 |  | 119 | 63 | 174 |  | 123 | 56 | 189 |  | 17 | 9 | 24 |
|  |  | Self-inflicted injuries | 0 | 0 | 0 |  | 23 | 3 | 43 |  | 43 | 6 | 80 |  | 2 | 0 | 4 |
|  |  | Violence | 6 | 4 | 8 |  | 96 | 61 | 131 |  | 79 | 50 | 108 |  | 15 | 9 | 20 |
|  |  | Other intentional injuries | 0 | 0 | 0 |  | 0 | 0 | 0 |  | 0 | 0 | 1 |  | 0 | 0 | 0 |
| Men |  |  |  |  |  |  |  |  |  |  |  |  |  |  |  |  |  |
| Injuries | |  | 143 | 132 | 154 |  | 3213 | 1835 | 4592 |  | 4862 | 2751 | 6972 |  | 418 | 220 | 617 |
|  | Unintentional injuries | | 123 | 119 | 127 |  | 2290 | 1384 | 3196 |  | 3809 | 2220 | 5398 |  | 338 | 182 | 494 |
|  |  | Transport injuries | 123 | 119 | 127 |  | 1131 | 919 | 1342 |  | 1454 | 1128 | 1780 |  | 118 | 86 | 150 |
|  |  | Poisonings | 0 | 0 | 0 |  | 200 | 80 | 320 |  | 517 | 240 | 794 |  | 40 | 17 | 62 |
|  |  | Falls | 0 | 0 | 0 |  | 78 | 31 | 125 |  | 153 | 71 | 234 |  | 18 | 8 | 28 |
|  |  | Fires, heat and hot substances | 0 | 0 | 0 |  | 61 | 25 | 98 |  | 132 | 61 | 203 |  | 17 | 7 | 26 |
|  |  | Drownings | 0 | 0 | 0 |  | 183 | 73 | 292 |  | 245 | 114 | 377 |  | 16 | 7 | 25 |
|  |  | Other unintentional injuries | 0 | 0 | 0 |  | 637 | 255 | 1018 |  | 1308 | 607 | 2009 |  | 130 | 56 | 204 |
|  | Intentional injuries | | 20 | 12 | 27 |  | 924 | 451 | 1396 |  | 1052 | 531 | 1574 |  | 80 | 38 | 123 |
|  |  | Self-inflicted injuries | 0 | 0 | 0 |  | 565 | 226 | 904 |  | 778 | 361 | 1195 |  | 64 | 28 | 100 |
|  |  | Violence | 20 | 12 | 27 |  | 349 | 221 | 477 |  | 254 | 161 | 347 |  | 16 | 10 | 22 |
|  |  | Other intentional injuries | 0 | 0 | 0 |  | 9 | 4 | 15 |  | 21 | 10 | 32 |  | 0 | 0 | 1 |

Table 3: Deaths from injuries attributable to alcohol consumption (with harms to others): Asia East

|  |  |  | 0 to 14 years of age | | |  | 15 to 34 years of age | | |  | 35 to 64 years of age | | |  | 65 years of age and older | | |
| --- | --- | --- | --- | --- | --- | --- | --- | --- | --- | --- | --- | --- | --- | --- | --- | --- | --- |
|  |  |  | Point estimate | Lower 95% CI | Upper 95% CI |  | Point estimate | Lower 95% CI | Upper 95% CI |  | Point estimate | Lower 95% CI | Upper 95% CI |  | Point estimate | Lower 95% CI | Upper 95% CI |
| Women | |  |  |  |  |  |  |  |  |  |  |  |  |  |  |  |  |
| Injuries | |  | 246 | 67 | 425 |  | 2397 | 662 | 4132 |  | 6418 | 953 | 12797 |  | 1250 | 79 | 2454 |
|  | Unintentional injuries | | 159 | 26 | 293 |  | 1797 | 430 | 3163 |  | 4239 | 772 | 8108 |  | 814 | 59 | 1588 |
|  |  | Transport injuries | 159 | 26 | 293 |  | 1637 | 406 | 2867 |  | 2827 | 772 | 4882 |  | 294 | 59 | 530 |
|  |  | Poisonings | 0 | 0 | 0 |  | 39 | 6 | 72 |  | 268 | 0 | 613 |  | 42 | 0 | 85 |
|  |  | Falls | 0 | 0 | 0 |  | 33 | 5 | 62 |  | 349 | 0 | 797 |  | 254 | 0 | 516 |
|  |  | Fires, heat and hot substances | 0 | 0 | 0 |  | 3 | 0 | 5 |  | 26 | 0 | 60 |  | 29 | 0 | 59 |
|  |  | Drownings | 0 | 0 | 0 |  | 50 | 8 | 93 |  | 380 | 0 | 869 |  | 101 | 0 | 205 |
|  |  | Other unintentional injuries | 0 | 0 | 0 |  | 35 | 5 | 64 |  | 388 | 0 | 888 |  | 95 | 0 | 193 |
|  | Intentional injuries | | 86 | 41 | 132 |  | 601 | 232 | 969 |  | 2179 | 181 | 4688 |  | 436 | 20 | 866 |
|  |  | Self-inflicted injuries | 0 | 0 | 0 |  | 163 | 25 | 302 |  | 1787 | 0 | 4083 |  | 393 | 0 | 800 |
|  |  | Violence | 86 | 41 | 132 |  | 436 | 207 | 665 |  | 380 | 181 | 579 |  | 43 | 20 | 65 |
|  |  | Other intentional injuries | 0 | 0 | 0 |  | 2 | 0 | 3 |  | 11 | 0 | 25 |  | 0 | 0 | 1 |
| Men |  |  |  |  |  |  |  |  |  |  |  |  |  |  |  |  |  |
| Injuries | |  | 325 | 292 | 359 |  | 14559 | 9807 | 19311 |  | 53134 | 23313 | 82954 |  | 12286 | 4335 | 20237 |
|  | Unintentional injuries | | 269 | 265 | 272 |  | 11424 | 8338 | 14509 |  | 40120 | 18298 | 61942 |  | 7429 | 2752 | 12107 |
|  |  | Transport injuries | 269 | 265 | 272 |  | 7785 | 6682 | 8887 |  | 13196 | 8086 | 18307 |  | 1163 | 725 | 1600 |
|  |  | Poisonings | 0 | 0 | 0 |  | 405 | 184 | 625 |  | 4317 | 1638 | 6997 |  | 646 | 209 | 1083 |
|  |  | Falls | 0 | 0 | 0 |  | 835 | 380 | 1289 |  | 8495 | 3222 | 13768 |  | 2997 | 969 | 5024 |
|  |  | Fires, heat and hot substances | 0 | 0 | 0 |  | 21 | 10 | 33 |  | 478 | 181 | 775 |  | 332 | 107 | 557 |
|  |  | Drownings | 0 | 0 | 0 |  | 1061 | 483 | 1638 |  | 3944 | 1496 | 6392 |  | 885 | 286 | 1483 |
|  |  | Other unintentional injuries | 0 | 0 | 0 |  | 1318 | 600 | 2036 |  | 9689 | 3675 | 15703 |  | 1407 | 455 | 2359 |
|  | Intentional injuries | | 57 | 27 | 86 |  | 3135 | 1468 | 4802 |  | 13014 | 5015 | 21012 |  | 4857 | 1584 | 8130 |
|  |  | Self-inflicted injuries | 0 | 0 | 0 |  | 1040 | 474 | 1607 |  | 11923 | 4522 | 19324 |  | 4762 | 1540 | 7984 |
|  |  | Violence | 57 | 27 | 86 |  | 2027 | 964 | 3090 |  | 823 | 392 | 1255 |  | 86 | 41 | 131 |
|  |  | Other intentional injuries | 0 | 0 | 0 |  | 68 | 31 | 104 |  | 267 | 101 | 433 |  | 9 | 3 | 14 |

Table 4: Deaths from injuries attributable to alcohol consumption (with harms to others): Asia South

|  |  |  | 0 to 14 years of age | | |  | 15 to 34 years of age | | |  | 35 to 64 years of age | | |  | 65 years of age and older | | |
| --- | --- | --- | --- | --- | --- | --- | --- | --- | --- | --- | --- | --- | --- | --- | --- | --- | --- |
|  |  |  | Point estimate | Lower 95% CI | Upper 95% CI |  | Point estimate | Lower 95% CI | Upper 95% CI |  | Point estimate | Lower 95% CI | Upper 95% CI |  | Point estimate | Lower 95% CI | Upper 95% CI |
| Women | |  |  |  |  |  |  |  |  |  |  |  |  |  |  |  |  |
| Injuries | |  | 710 | 16 | 1663 |  | 2130 | 130 | 10136 |  | 3871 | 144 | 34414 |  | 362 | 0 | 3417 |
|  | Unintentional injuries | | 615 | 16 | 1215 |  | 1364 | 130 | 5369 |  | 2886 | 144 | 25275 |  | 248 | 0 | 2689 |
|  |  | Transport injuries | 615 | 16 | 1215 |  | 1247 | 130 | 2364 |  | 1691 | 144 | 3238 |  | 225 | 0 | 467 |
|  |  | Poisonings | 0 | 0 | 0 |  | 7 | 0 | 181 |  | 210 | 0 | 3877 |  | 3 | 0 | 344 |
|  |  | Falls | 0 | 0 | 0 |  | 5 | 0 | 123 |  | 120 | 0 | 2208 |  | 6 | 0 | 634 |
|  |  | Fires, heat and hot substances | 0 | 0 | 0 |  | 63 | 0 | 1613 |  | 396 | 0 | 7294 |  | 3 | 0 | 336 |
|  |  | Drownings | 0 | 0 | 0 |  | 9 | 0 | 228 |  | 72 | 0 | 1335 |  | 1 | 0 | 136 |
|  |  | Other unintentional injuries | 0 | 0 | 0 |  | 33 | 0 | 859 |  | 397 | 0 | 7322 |  | 8 | 0 | 771 |
|  | Intentional injuries | | 95 | 0 | 449 |  | 767 | 0 | 4767 |  | 985 | 0 | 9139 |  | 115 | 0 | 728 |
|  |  | Self-inflicted injuries | 0 | 0 | 0 |  | 54 | 0 | 1378 |  | 321 | 0 | 5921 |  | 2 | 0 | 183 |
|  |  | Violence | 95 | 0 | 449 |  | 713 | 0 | 3382 |  | 659 | 0 | 3128 |  | 113 | 0 | 536 |
|  |  | Other intentional injuries | 0 | 0 | 0 |  | 0 | 0 | 8 |  | 5 | 0 | 91 |  | 0 | 0 | 9 |
| Men |  |  |  |  |  |  |  |  |  |  |  |  |  |  |  |  |  |
| Injuries | |  | 939 | 798 | 1416 |  | 21391 | 8278 | 40458 |  | 47686 | 11856 | 86844 |  | 7798 | 401 | 15773 |
|  | Unintentional injuries | | 817 | 798 | 836 |  | 15159 | 7676 | 22642 |  | 37680 | 10582 | 64779 |  | 6639 | 398 | 12882 |
|  |  | Transport injuries | 817 | 798 | 836 |  | 8390 | 6672 | 10107 |  | 15012 | 7296 | 22728 |  | 1208 | 385 | 2033 |
|  |  | Poisonings | 0 | 0 | 0 |  | 637 | 94 | 1179 |  | 4351 | 631 | 8070 |  | 577 | 1 | 1152 |
|  |  | Falls | 0 | 0 | 0 |  | 678 | 101 | 1256 |  | 3277 | 475 | 6080 |  | 2315 | 6 | 4624 |
|  |  | Fires, heat and hot substances | 0 | 0 | 0 |  | 1275 | 189 | 2362 |  | 3323 | 482 | 6165 |  | 344 | 1 | 687 |
|  |  | Drownings | 0 | 0 | 0 |  | 1138 | 169 | 2107 |  | 2313 | 335 | 4292 |  | 400 | 1 | 799 |
|  |  | Other unintentional injuries | 0 | 0 | 0 |  | 3041 | 451 | 5630 |  | 9403 | 1363 | 17443 |  | 1796 | 4 | 3587 |
|  | Intentional injuries | | 122 | 0 | 581 |  | 6232 | 602 | 17817 |  | 10006 | 1275 | 22065 |  | 1159 | 2 | 2891 |
|  |  | Self-inflicted injuries | 0 | 0 | 0 |  | 3964 | 588 | 7341 |  | 8522 | 1235 | 15809 |  | 880 | 2 | 1758 |
|  |  | Violence | 122 | 0 | 581 |  | 2169 | 0 | 10291 |  | 1212 | 0 | 5752 |  | 209 | 0 | 994 |
|  |  | Other intentional injuries | 0 | 0 | 0 |  | 100 | 15 | 185 |  | 271 | 39 | 503 |  | 70 | 0 | 139 |

Table 5: Deaths from injuries attributable to alcohol consumption (with harms to others): Asia Southeast

|  |  |  | 0 to 14 years of age | | |  | 15 to 34 years of age | | |  | 35 to 64 years of age | | |  | 65 years of age and older | | |
| --- | --- | --- | --- | --- | --- | --- | --- | --- | --- | --- | --- | --- | --- | --- | --- | --- | --- |
|  |  |  | Point estimate | Lower 95% CI | Upper 95% CI |  | Point estimate | Lower 95% CI | Upper 95% CI |  | Point estimate | Lower 95% CI | Upper 95% CI |  | Point estimate | Lower 95% CI | Upper 95% CI |
| Women | |  |  |  |  |  |  |  |  |  |  |  |  |  |  |  |  |
| Injuries | |  | 171 | 13 | 355 |  | 1191 | 155 | 2844 |  | 1763 | 132 | 5448 |  | 153 | 18 | 343 |
|  | Unintentional injuries | | 136 | 0 | 298 |  | 761 | 15 | 2022 |  | 1307 | 0 | 4411 |  | 105 | 0 | 262 |
|  |  | Transport injuries | 136 | 0 | 298 |  | 535 | 15 | 1124 |  | 641 | 0 | 1402 |  | 88 | 0 | 200 |
|  |  | Poisonings | 0 | 0 | 0 |  | 5 | 0 | 20 |  | 37 | 0 | 165 |  | 1 | 0 | 4 |
|  |  | Falls | 0 | 0 | 0 |  | 4 | 0 | 17 |  | 32 | 0 | 146 |  | 4 | 0 | 14 |
|  |  | Fires, heat and hot substances | 0 | 0 | 0 |  | 36 | 0 | 142 |  | 64 | 0 | 288 |  | 1 | 0 | 4 |
|  |  | Drownings | 0 | 0 | 0 |  | 10 | 0 | 41 |  | 30 | 0 | 137 |  | 1 | 0 | 3 |
|  |  | Other unintentional injuries | 0 | 0 | 0 |  | 171 | 0 | 678 |  | 503 | 0 | 2271 |  | 10 | 0 | 37 |
|  | Intentional injuries | | 35 | 13 | 58 |  | 430 | 140 | 822 |  | 457 | 132 | 1037 |  | 48 | 17 | 81 |
|  |  | Self-inflicted injuries | 0 | 0 | 0 |  | 52 | 0 | 205 |  | 100 | 0 | 454 |  | 1 | 0 | 4 |
|  |  | Violence | 35 | 13 | 58 |  | 378 | 140 | 616 |  | 355 | 132 | 579 |  | 47 | 17 | 76 |
|  |  | Other intentional injuries | 0 | 0 | 0 |  | 0 | 0 | 1 |  | 1 | 0 | 4 |  | 0 | 0 | 0 |
| Men |  |  |  |  |  |  |  |  |  |  |  |  |  |  |  |  |  |
| Injuries | |  | 345 | 291 | 400 |  | 10269 | 5366 | 15173 |  | 23936 | 6666 | 42618 |  | 2707 | 680 | 4734 |
|  | Unintentional injuries | | 271 | 264 | 278 |  | 6446 | 4012 | 8880 |  | 18490 | 5032 | 33359 |  | 2209 | 557 | 3860 |
|  |  | Transport injuries | 271 | 264 | 278 |  | 3960 | 3264 | 4657 |  | 5396 | 1582 | 10621 |  | 321 | 164 | 478 |
|  |  | Poisonings | 0 | 0 | 0 |  | 79 | 24 | 134 |  | 830 | 219 | 1442 |  | 102 | 21 | 182 |
|  |  | Falls | 0 | 0 | 0 |  | 148 | 44 | 251 |  | 1405 | 370 | 2439 |  | 523 | 109 | 937 |
|  |  | Fires, heat and hot substances | 0 | 0 | 0 |  | 122 | 37 | 208 |  | 522 | 138 | 906 |  | 62 | 13 | 111 |
|  |  | Drownings | 0 | 0 | 0 |  | 327 | 98 | 555 |  | 1110 | 293 | 1928 |  | 141 | 29 | 252 |
|  |  | Other unintentional injuries | 0 | 0 | 0 |  | 1810 | 545 | 3076 |  | 9227 | 2431 | 16022 |  | 1060 | 221 | 1900 |
|  | Intentional injuries | | 74 | 28 | 121 |  | 3823 | 1353 | 6292 |  | 5446 | 1633 | 9259 |  | 498 | 123 | 874 |
|  |  | Self-inflicted injuries | 0 | 0 | 0 |  | 876 | 264 | 1489 |  | 3480 | 917 | 6043 |  | 373 | 78 | 668 |
|  |  | Violence | 74 | 28 | 121 |  | 2923 | 1082 | 4764 |  | 1857 | 688 | 3027 |  | 115 | 43 | 188 |
|  |  | Other intentional injuries | 0 | 0 | 0 |  | 23 | 7 | 40 |  | 109 | 29 | 190 |  | 10 | 2 | 18 |

Table 6: Deaths from injuries attributable to alcohol consumption (with harms to others): Australiasia

|  |  |  | 0 to 14 years of age | | |  | 15 to 34 years of age | | |  | 35 to 64 years of age | | |  | 65 years of age and older | | |
| --- | --- | --- | --- | --- | --- | --- | --- | --- | --- | --- | --- | --- | --- | --- | --- | --- | --- |
|  |  |  | Point estimate | Lower 95% CI | Upper 95% CI |  | Point estimate | Lower 95% CI | Upper 95% CI |  | Point estimate | Lower 95% CI | Upper 95% CI |  | Point estimate | Lower 95% CI | Upper 95% CI |
| Women | |  |  |  |  |  |  |  |  |  |  |  |  |  |  |  |  |
| Injuries | |  | 3 | 2 | 4 |  | 52 | 32 | 72 |  | 60 | 33 | 88 |  | 46 | 22 | 70 |
|  | Unintentional injuries | | 2 | 1 | 2 |  | 30 | 20 | 40 |  | 35 | 20 | 50 |  | 43 | 20 | 66 |
|  |  | Transport injuries | 2 | 1 | 2 |  | 20 | 15 | 25 |  | 17 | 12 | 23 |  | 5 | 2 | 7 |
|  |  | Poisonings | 0 | 0 | 0 |  | 5 | 2 | 8 |  | 7 | 3 | 11 |  | 1 | 1 | 2 |
|  |  | Falls | 0 | 0 | 0 |  | 1 | 0 | 1 |  | 2 | 1 | 3 |  | 14 | 7 | 22 |
|  |  | Fires, heat and hot substances | 0 | 0 | 0 |  | 0 | 0 | 1 |  | 1 | 0 | 1 |  | 1 | 0 | 1 |
|  |  | Drownings | 0 | 0 | 0 |  | 1 | 0 | 1 |  | 1 | 1 | 2 |  | 0 | 0 | 1 |
|  |  | Other unintentional injuries | 0 | 0 | 0 |  | 4 | 2 | 5 |  | 7 | 3 | 10 |  | 22 | 10 | 34 |
|  | Intentional injuries | | 1 | 1 | 2 |  | 22 | 13 | 32 |  | 25 | 13 | 38 |  | 3 | 2 | 5 |
|  |  | Self-inflicted injuries | 0 | 0 | 0 |  | 13 | 6 | 20 |  | 21 | 10 | 32 |  | 2 | 1 | 4 |
|  |  | Violence | 1 | 1 | 2 |  | 9 | 6 | 12 |  | 4 | 3 | 6 |  | 1 | 1 | 1 |
|  |  | Other intentional injuries | 0 | 0 | 0 |  | 0 | 0 | 0 |  | 0 | 0 | 0 |  | 0 | 0 | 0 |
| Men |  |  |  |  |  |  |  |  |  |  |  |  |  |  |  |  |  |
| Injuries | |  | 4 | 4 | 5 |  | 381 | 216 | 546 |  | 592 | 305 | 880 |  | 236 | 107 | 366 |
|  | Unintentional injuries | | 3 | 3 | 3 |  | 207 | 130 | 285 |  | 304 | 163 | 445 |  | 188 | 85 | 291 |
|  |  | Transport injuries | 3 | 3 | 3 |  | 96 | 78 | 115 |  | 70 | 50 | 89 |  | 10 | 7 | 14 |
|  |  | Poisonings | 0 | 0 | 0 |  | 40 | 19 | 62 |  | 67 | 32 | 102 |  | 4 | 2 | 6 |
|  |  | Falls | 0 | 0 | 0 |  | 12 | 5 | 18 |  | 31 | 15 | 48 |  | 62 | 27 | 97 |
|  |  | Fires, heat and hot substances | 0 | 0 | 0 |  | 2 | 1 | 2 |  | 8 | 4 | 12 |  | 4 | 2 | 7 |
|  |  | Drownings | 0 | 0 | 0 |  | 12 | 6 | 19 |  | 20 | 9 | 30 |  | 5 | 2 | 8 |
|  |  | Other unintentional injuries | 0 | 0 | 0 |  | 46 | 21 | 70 |  | 108 | 52 | 164 |  | 103 | 46 | 160 |
|  | Intentional injuries | | 1 | 1 | 1 |  | 174 | 87 | 261 |  | 288 | 142 | 435 |  | 48 | 21 | 75 |
|  |  | Self-inflicted injuries | 0 | 0 | 0 |  | 148 | 69 | 228 |  | 274 | 132 | 416 |  | 47 | 21 | 74 |
|  |  | Violence | 1 | 1 | 1 |  | 25 | 17 | 33 |  | 14 | 10 | 18 |  | 1 | 0 | 1 |
|  |  | Other intentional injuries | 0 | 0 | 0 |  | 0 | 0 | 0 |  | 0 | 0 | 1 |  | 0 | 0 | 0 |

Table 7: Deaths from injuries attributable to alcohol consumption (with harms to others): Caribbean

|  |  |  | 0 to 14 years of age | | |  | 15 to 34 years of age | | |  | 35 to 64 years of age | | |  | 65 years of age and older | | |
| --- | --- | --- | --- | --- | --- | --- | --- | --- | --- | --- | --- | --- | --- | --- | --- | --- | --- |
|  |  |  | Point estimate | Lower 95% CI | Upper 95% CI |  | Point estimate | Lower 95% CI | Upper 95% CI |  | Point estimate | Lower 95% CI | Upper 95% CI |  | Point estimate | Lower 95% CI | Upper 95% CI |
| Women | |  |  |  |  |  |  |  |  |  |  |  |  |  |  |  |  |
| Injuries | |  | 12 | 4 | 19 |  | 178 | 64 | 293 |  | 96 | 42 | 150 |  | 16 | 5 | 28 |
|  | Unintentional injuries | | 8 | 2 | 14 |  | 108 | 26 | 190 |  | 64 | 23 | 105 |  | 13 | 3 | 23 |
|  |  | Transport injuries | 8 | 2 | 14 |  | 50 | 19 | 81 |  | 47 | 18 | 76 |  | 8 | 2 | 13 |
|  |  | Poisonings | 0 | 0 | 0 |  | 1 | 0 | 2 |  | 0 | 0 | 1 |  | 0 | 0 | 0 |
|  |  | Falls | 0 | 0 | 0 |  | 0 | 0 | 1 |  | 1 | 0 | 2 |  | 3 | 1 | 5 |
|  |  | Fires, heat and hot substances | 0 | 0 | 0 |  | 1 | 0 | 2 |  | 1 | 0 | 1 |  | 0 | 0 | 0 |
|  |  | Drownings | 0 | 0 | 0 |  | 2 | 0 | 3 |  | 1 | 0 | 1 |  | 0 | 0 | 0 |
|  |  | Other unintentional injuries | 0 | 0 | 0 |  | 54 | 7 | 101 |  | 15 | 5 | 24 |  | 3 | 1 | 5 |
|  | Intentional injuries | | 4 | 2 | 5 |  | 70 | 37 | 102 |  | 32 | 18 | 45 |  | 3 | 2 | 4 |
|  |  | Self-inflicted injuries | 0 | 0 | 0 |  | 14 | 2 | 26 |  | 6 | 2 | 9 |  | 0 | 0 | 1 |
|  |  | Violence | 4 | 2 | 5 |  | 56 | 36 | 76 |  | 26 | 17 | 36 |  | 3 | 2 | 4 |
|  |  | Other intentional injuries | 0 | 0 | 0 |  | 0 | 0 | 0 |  | 0 | 0 | 0 |  | 0 | 0 | 0 |
| Men |  |  |  |  |  |  |  |  |  |  |  |  |  |  |  |  |  |
| Injuries | |  | 19 | 17 | 20 |  | 1027 | 631 | 1423 |  | 1114 | 602 | 1626 |  | 247 | 105 | 390 |
|  | Unintentional injuries | | 14 | 14 | 14 |  | 570 | 361 | 778 |  | 733 | 409 | 1058 |  | 188 | 80 | 296 |
|  |  | Transport injuries | 14 | 14 | 14 |  | 334 | 265 | 402 |  | 315 | 237 | 394 |  | 36 | 26 | 45 |
|  |  | Poisonings | 0 | 0 | 0 |  | 19 | 8 | 31 |  | 34 | 14 | 54 |  | 2 | 1 | 4 |
|  |  | Falls | 0 | 0 | 0 |  | 12 | 5 | 19 |  | 49 | 20 | 77 |  | 64 | 23 | 105 |
|  |  | Fires, heat and hot substances | 0 | 0 | 0 |  | 2 | 1 | 3 |  | 7 | 3 | 11 |  | 2 | 1 | 4 |
|  |  | Drownings | 0 | 0 | 0 |  | 32 | 13 | 52 |  | 45 | 19 | 72 |  | 5 | 2 | 8 |
|  |  | Other unintentional injuries | 0 | 0 | 0 |  | 170 | 69 | 272 |  | 283 | 116 | 449 |  | 78 | 28 | 128 |
|  | Intentional injuries | | 5 | 3 | 6 |  | 457 | 269 | 645 |  | 381 | 193 | 569 |  | 60 | 25 | 94 |
|  |  | Self-inflicted injuries | 0 | 0 | 0 |  | 92 | 37 | 146 |  | 217 | 89 | 345 |  | 45 | 16 | 74 |
|  |  | Violence | 5 | 3 | 6 |  | 364 | 232 | 497 |  | 162 | 103 | 221 |  | 15 | 9 | 20 |
|  |  | Other intentional injuries | 0 | 0 | 0 |  | 1 | 0 | 2 |  | 1 | 1 | 2 |  | 0 | 0 | 0 |

Table 8: Deaths from injuries attributable to alcohol consumption (with harms to others): Europe Central

|  |  |  | 0 to 14 years of age | | |  | 15 to 34 years of age | | |  | 35 to 64 years of age | | |  | 65 years of age and older | | |
| --- | --- | --- | --- | --- | --- | --- | --- | --- | --- | --- | --- | --- | --- | --- | --- | --- | --- |
|  |  |  | Point estimate | Lower 95% CI | Upper 95% CI |  | Point estimate | Lower 95% CI | Upper 95% CI |  | Point estimate | Lower 95% CI | Upper 95% CI |  | Point estimate | Lower 95% CI | Upper 95% CI |
| Women | |  |  |  |  |  |  |  |  |  |  |  |  |  |  |  |  |
| Injuries | |  | 55 | 14 | 97 |  | 637 | 205 | 1070 |  | 1165 | 208 | 2123 |  | 261 | 52 | 470 |
|  | Unintentional injuries | | 46 | 9 | 84 |  | 436 | 132 | 740 |  | 764 | 124 | 1405 |  | 219 | 33 | 406 |
|  |  | Transport injuries | 46 | 9 | 84 |  | 349 | 130 | 567 |  | 418 | 111 | 724 |  | 141 | 18 | 263 |
|  |  | Poisonings | 0 | 0 | 0 |  | 21 | 0 | 41 |  | 60 | 2 | 118 |  | 3 | 1 | 5 |
|  |  | Falls | 0 | 0 | 0 |  | 11 | 0 | 22 |  | 78 | 3 | 154 |  | 56 | 11 | 101 |
|  |  | Fires, heat and hot substances | 0 | 0 | 0 |  | 4 | 0 | 8 |  | 19 | 1 | 38 |  | 3 | 1 | 5 |
|  |  | Drownings | 0 | 0 | 0 |  | 10 | 0 | 21 |  | 40 | 2 | 78 |  | 2 | 0 | 4 |
|  |  | Other unintentional injuries | 0 | 0 | 0 |  | 41 | 1 | 81 |  | 149 | 6 | 292 |  | 15 | 3 | 27 |
|  | Intentional injuries | | 9 | 5 | 12 |  | 201 | 73 | 330 |  | 401 | 84 | 718 |  | 42 | 19 | 64 |
|  |  | Self-inflicted injuries | 0 | 0 | 0 |  | 80 | 1 | 159 |  | 277 | 11 | 544 |  | 14 | 3 | 25 |
|  |  | Violence | 9 | 5 | 12 |  | 121 | 72 | 170 |  | 123 | 73 | 173 |  | 28 | 17 | 39 |
|  |  | Other intentional injuries | 0 | 0 | 0 |  | 0 | 0 | 1 |  | 0 | 0 | 0 |  | 0 | 0 | 0 |
| Men |  |  |  |  |  |  |  |  |  |  |  |  |  |  |  |  |  |
| Injuries | |  | 81 | 71 | 90 |  | 5372 | 3120 | 7624 |  | 17272 | 8546 | 23888 |  | 4752 | 2077 | 7428 |
|  | Unintentional injuries | | 68 | 64 | 72 |  | 3585 | 2247 | 4924 |  | 11013 | 5382 | 15428 |  | 3351 | 1462 | 5241 |
|  |  | Transport injuries | 68 | 64 | 72 |  | 1914 | 1479 | 2348 |  | 2985 | 1367 | 4603 |  | 474 | 211 | 738 |
|  |  | Poisonings | 0 | 0 | 0 |  | 243 | 112 | 375 |  | 1290 | 645 | 1740 |  | 185 | 80 | 290 |
|  |  | Falls | 0 | 0 | 0 |  | 215 | 99 | 332 |  | 1860 | 930 | 2508 |  | 1446 | 629 | 2263 |
|  |  | Fires, heat and hot substances | 0 | 0 | 0 |  | 52 | 24 | 80 |  | 343 | 172 | 463 |  | 124 | 54 | 193 |
|  |  | Drownings | 0 | 0 | 0 |  | 307 | 141 | 473 |  | 874 | 437 | 1179 |  | 176 | 77 | 276 |
|  |  | Other unintentional injuries | 0 | 0 | 0 |  | 854 | 392 | 1316 |  | 3660 | 1830 | 4935 |  | 946 | 411 | 1481 |
|  | Intentional injuries | | 13 | 8 | 18 |  | 1786 | 873 | 2700 |  | 6259 | 3165 | 8459 |  | 1401 | 616 | 2187 |
|  |  | Self-inflicted injuries | 0 | 0 | 0 |  | 1403 | 644 | 2161 |  | 5897 | 2949 | 7951 |  | 1361 | 592 | 2131 |
|  |  | Violence | 13 | 8 | 18 |  | 382 | 227 | 536 |  | 360 | 214 | 505 |  | 40 | 24 | 56 |
|  |  | Other intentional injuries | 0 | 0 | 0 |  | 2 | 1 | 3 |  | 2 | 1 | 3 |  | 0 | 0 | 0 |

Table 9: Deaths from injuries attributable to alcohol consumption (with harms to others): Europe Eastern

|  |  |  | 0 to 14 years of age | | |  | 15 to 34 years of age | | |  | 35 to 64 years of age | | |  | 65 years of age and older | | |
| --- | --- | --- | --- | --- | --- | --- | --- | --- | --- | --- | --- | --- | --- | --- | --- | --- | --- |
|  |  |  | Point estimate | Lower 95% CI | Upper 95% CI |  | Point estimate | Lower 95% CI | Upper 95% CI |  | Point estimate | Lower 95% CI | Upper 95% CI |  | Point estimate | Lower 95% CI | Upper 95% CI |
| Women | |  |  |  |  |  |  |  |  |  |  |  |  |  |  |  |  |
| Injuries | |  | 376 | 223 | 529 |  | 10004 | 5329 | 13722 |  | 16323 | 6108 | 26538 |  | 2604 | 1324 | 3784 |
|  | Unintentional injuries | | 289 | 166 | 412 |  | 6219 | 3291 | 9147 |  | 11803 | 3867 | 19738 |  | 1936 | 957 | 2800 |
|  |  | Transport injuries | 289 | 166 | 412 |  | 3600 | 2996 | 4205 |  | 4113 | 3312 | 4913 |  | 1525 | 957 | 1919 |
|  |  | Poisonings | 0 | 0 | 0 |  | 957 | 108 | 1806 |  | 3104 | 224 | 5984 |  | 96 | 0 | 205 |
|  |  | Falls | 0 | 0 | 0 |  | 169 | 19 | 318 |  | 457 | 33 | 882 |  | 65 | 0 | 139 |
|  |  | Fires, heat and hot substances | 0 | 0 | 0 |  | 126 | 14 | 237 |  | 479 | 35 | 923 |  | 55 | 0 | 118 |
|  |  | Drownings | 0 | 0 | 0 |  | 223 | 25 | 421 |  | 355 | 26 | 683 |  | 22 | 0 | 47 |
|  |  | Other unintentional injuries | 0 | 0 | 0 |  | 1145 | 129 | 2160 |  | 3295 | 238 | 6352 |  | 174 | 0 | 372 |
|  | Intentional injuries | | 87 | 57 | 117 |  | 3785 | 2038 | 4574 |  | 4521 | 2241 | 6801 |  | 668 | 367 | 984 |
|  |  | Self-inflicted injuries | 0 | 0 | 0 |  | 813 | 92 | 1534 |  | 1237 | 89 | 2384 |  | 107 | 0 | 230 |
|  |  | Violence | 87 | 57 | 117 |  | 2972 | 1947 | 3040 |  | 3284 | 2151 | 4417 |  | 560 | 367 | 753 |
|  |  | Other intentional injuries | 0 | 0 | 0 |  | 0 | 0 | 0 |  | 0 | 0 | 0 |  | 0 | 0 | 0 |
| Men |  |  |  |  |  |  |  |  |  |  |  |  |  |  |  |  |  |
| Injuries | |  | 558 | 507 | 609 |  | 89623 | 58704 | 94734 |  | 154775 | 94814 | 183954 |  | 13577 | 7232 | 19354 |
|  | Unintentional injuries | | 462 | 444 | 480 |  | 60287 | 41192 | 63728 |  | 118946 | 73852 | 139267 |  | 10399 | 5673 | 14559 |
|  |  | Transport injuries | 462 | 444 | 480 |  | 17094 | 17094 | 17094 |  | 18196 | 18196 | 18196 |  | 2739 | 2122 | 2788 |
|  |  | Poisonings | 0 | 0 | 0 |  | 14948 | 8340 | 16138 |  | 36071 | 19926 | 43347 |  | 2071 | 960 | 3183 |
|  |  | Falls | 0 | 0 | 0 |  | 2735 | 1526 | 2953 |  | 7103 | 3924 | 8536 |  | 944 | 437 | 1450 |
|  |  | Fires, heat and hot substances | 0 | 0 | 0 |  | 1765 | 985 | 1905 |  | 5772 | 3189 | 6936 |  | 676 | 313 | 1039 |
|  |  | Drownings | 0 | 0 | 0 |  | 4815 | 2686 | 5198 |  | 7338 | 4053 | 8817 |  | 520 | 241 | 799 |
|  |  | Other unintentional injuries | 0 | 0 | 0 |  | 18931 | 10562 | 20439 |  | 44465 | 24563 | 53434 |  | 3449 | 1599 | 5300 |
|  | Intentional injuries | | 96 | 63 | 129 |  | 29337 | 17511 | 31006 |  | 35828 | 20962 | 44687 |  | 3177 | 1559 | 4795 |
|  |  | Self-inflicted injuries | 0 | 0 | 0 |  | 17559 | 9797 | 18958 |  | 24436 | 13499 | 29364 |  | 2723 | 1262 | 4184 |
|  |  | Violence | 96 | 63 | 129 |  | 11777 | 7715 | 12048 |  | 11393 | 7463 | 15323 |  | 454 | 298 | 611 |
|  |  | Other intentional injuries | 0 | 0 | 0 |  | 0 | 0 | 0 |  | 0 | 0 | 0 |  | 0 | 0 | 0 |

Table 10: Deaths from injuries attributable to alcohol consumption (with harms to others): Europe Western

|  |  |  | 0 to 14 years of age | | |  | 15 to 34 years of age | | |  | 35 to 64 years of age | | |  | 65 years of age and older | | |
| --- | --- | --- | --- | --- | --- | --- | --- | --- | --- | --- | --- | --- | --- | --- | --- | --- | --- |
|  |  |  | Point estimate | Lower 95% CI | Upper 95% CI |  | Point estimate | Lower 95% CI | Upper 95% CI |  | Point estimate | Lower 95% CI | Upper 95% CI |  | Point estimate | Lower 95% CI | Upper 95% CI |
| Women | |  |  |  |  |  |  |  |  |  |  |  |  |  |  |  |  |
| Injuries | |  | 42 | 14 | 70 |  | 830 | 336 | 1323 |  | 1769 | 350 | 3189 |  | 1844 | 656 | 3033 |
|  | Unintentional injuries | | 28 | 5 | 50 |  | 510 | 168 | 851 |  | 924 | 175 | 1674 |  | 1653 | 578 | 2729 |
|  |  | Transport injuries | 28 | 5 | 50 |  | 431 | 140 | 722 |  | 405 | 115 | 696 |  | 139 | 27 | 252 |
|  |  | Poisonings | 0 | 0 | 0 |  | 25 | 9 | 41 |  | 98 | 11 | 184 |  | 27 | 10 | 44 |
|  |  | Falls | 0 | 0 | 0 |  | 10 | 4 | 17 |  | 147 | 17 | 276 |  | 804 | 293 | 1316 |
|  |  | Fires, heat and hot substances | 0 | 0 | 0 |  | 6 | 2 | 9 |  | 35 | 4 | 66 |  | 27 | 10 | 45 |
|  |  | Drownings | 0 | 0 | 0 |  | 7 | 3 | 12 |  | 36 | 4 | 68 |  | 17 | 6 | 27 |
|  |  | Other unintentional injuries | 0 | 0 | 0 |  | 31 | 11 | 50 |  | 204 | 24 | 384 |  | 639 | 233 | 1046 |
|  | Intentional injuries | | 14 | 9 | 20 |  | 320 | 168 | 472 |  | 845 | 174 | 1515 |  | 191 | 78 | 304 |
|  |  | Self-inflicted injuries | 0 | 0 | 0 |  | 135 | 49 | 221 |  | 700 | 81 | 1318 |  | 162 | 59 | 264 |
|  |  | Violence | 14 | 9 | 20 |  | 185 | 119 | 251 |  | 145 | 93 | 197 |  | 29 | 19 | 40 |
|  |  | Other intentional injuries | 0 | 0 | 0 |  | 0 | 0 | 0 |  | 0 | 0 | 0 |  | 0 | 0 | 0 |
| Men |  |  |  |  |  |  |  |  |  |  |  |  |  |  |  |  |  |
| Injuries | |  | 59 | 51 | 66 |  | 6823 | 3939 | 9707 |  | 15541 | 7630 | 23451 |  | 7847 | 3368 | 12327 |
|  | Unintentional injuries | | 41 | 40 | 42 |  | 4135 | 2645 | 5626 |  | 8061 | 4066 | 12056 |  | 5858 | 2528 | 9189 |
|  |  | Transport injuries | 41 | 40 | 42 |  | 2543 | 1930 | 3156 |  | 2233 | 1333 | 3132 |  | 384 | 235 | 534 |
|  |  | Poisonings | 0 | 0 | 0 |  | 432 | 194 | 670 |  | 908 | 426 | 1391 |  | 105 | 44 | 167 |
|  |  | Falls | 0 | 0 | 0 |  | 253 | 114 | 393 |  | 1673 | 785 | 2562 |  | 2658 | 1114 | 4202 |
|  |  | Fires, heat and hot substances | 0 | 0 | 0 |  | 63 | 28 | 98 |  | 274 | 129 | 420 |  | 130 | 54 | 205 |
|  |  | Drownings | 0 | 0 | 0 |  | 201 | 90 | 312 |  | 524 | 246 | 803 |  | 180 | 75 | 285 |
|  |  | Other unintentional injuries | 0 | 0 | 0 |  | 643 | 289 | 998 |  | 2448 | 1148 | 3748 |  | 2401 | 1006 | 3796 |
|  | Intentional injuries | | 18 | 12 | 25 |  | 2688 | 1295 | 4081 |  | 7480 | 3564 | 11395 |  | 1989 | 840 | 3138 |
|  |  | Self-inflicted injuries | 0 | 0 | 0 |  | 2229 | 1000 | 3457 |  | 7151 | 3353 | 10949 |  | 1959 | 821 | 3097 |
|  |  | Violence | 18 | 12 | 25 |  | 457 | 294 | 620 |  | 328 | 211 | 445 |  | 30 | 19 | 40 |
|  |  | Other intentional injuries | 0 | 0 | 0 |  | 2 | 1 | 3 |  | 1 | 0 | 1 |  | 0 | 0 | 0 |

Table 11: Deaths from injuries attributable to alcohol consumption (with harms to others): Latin America Andean

|  |  |  | 0 to 14 years of age | | |  | 15 to 34 years of age | | |  | 35 to 64 years of age | | |  | 65 years of age and older | | |
| --- | --- | --- | --- | --- | --- | --- | --- | --- | --- | --- | --- | --- | --- | --- | --- | --- | --- |
|  |  |  | Point estimate | Lower 95% CI | Upper 95% CI |  | Point estimate | Lower 95% CI | Upper 95% CI |  | Point estimate | Lower 95% CI | Upper 95% CI |  | Point estimate | Lower 95% CI | Upper 95% CI |
| Women | |  |  |  |  |  |  |  |  |  |  |  |  |  |  |  |  |
| Injuries | |  | 24 | 7 | 42 |  | 184 | 51 | 352 |  | 129 | 36 | 228 |  | 31 | 3 | 65 |
|  | Unintentional injuries | | 18 | 4 | 32 |  | 123 | 30 | 241 |  | 104 | 25 | 189 |  | 28 | 2 | 60 |
|  |  | Transport injuries | 18 | 4 | 32 |  | 83 | 30 | 136 |  | 75 | 25 | 126 |  | 10 | 2 | 19 |
|  |  | Poisonings | 0 | 0 | 0 |  | 2 | 0 | 5 |  | 1 | 0 | 2 |  | 0 | 0 | 1 |
|  |  | Falls | 0 | 0 | 0 |  | 1 | 0 | 3 |  | 1 | 0 | 2 |  | 1 | 0 | 2 |
|  |  | Fires, heat and hot substances | 0 | 0 | 0 |  | 2 | 0 | 4 |  | 1 | 0 | 3 |  | 1 | 0 | 1 |
|  |  | Drownings | 0 | 0 | 0 |  | 4 | 0 | 11 |  | 2 | 0 | 5 |  | 1 | 0 | 2 |
|  |  | Other unintentional injuries | 0 | 0 | 0 |  | 31 | 0 | 82 |  | 24 | 0 | 52 |  | 15 | 0 | 35 |
|  | Intentional injuries | | 6 | 3 | 9 |  | 61 | 22 | 112 |  | 25 | 11 | 39 |  | 4 | 2 | 5 |
|  |  | Self-inflicted injuries | 0 | 0 | 0 |  | 18 | 0 | 47 |  | 3 | 0 | 7 |  | 0 | 0 | 1 |
|  |  | Violence | 6 | 3 | 9 |  | 44 | 22 | 65 |  | 22 | 11 | 32 |  | 3 | 2 | 5 |
|  |  | Other intentional injuries | 0 | 0 | 0 |  | 0 | 0 | 0 |  | 0 | 0 | 0 |  | 0 | 0 | 0 |
| Men |  |  |  |  |  |  |  |  |  |  |  |  |  |  |  |  |  |
| Injuries | |  | 45 | 40 | 50 |  | 1535 | 736 | 2335 |  | 2106 | 833 | 3380 |  | 106 | 48 | 164 |
|  | Unintentional injuries | | 37 | 36 | 38 |  | 1022 | 499 | 1546 |  | 1803 | 706 | 2901 |  | 91 | 41 | 140 |
|  |  | Transport injuries | 37 | 36 | 38 |  | 409 | 322 | 495 |  | 515 | 336 | 694 |  | 32 | 26 | 38 |
|  |  | Poisonings | 0 | 0 | 0 |  | 15 | 4 | 25 |  | 49 | 14 | 83 |  | 1 | 0 | 2 |
|  |  | Falls | 0 | 0 | 0 |  | 20 | 6 | 35 |  | 60 | 17 | 103 |  | 4 | 1 | 6 |
|  |  | Fires, heat and hot substances | 0 | 0 | 0 |  | 11 | 3 | 19 |  | 25 | 7 | 43 |  | 2 | 0 | 3 |
|  |  | Drownings | 0 | 0 | 0 |  | 114 | 33 | 195 |  | 144 | 41 | 247 |  | 5 | 1 | 10 |
|  |  | Other unintentional injuries | 0 | 0 | 0 |  | 454 | 130 | 778 |  | 1011 | 291 | 1731 |  | 47 | 12 | 82 |
|  | Intentional injuries | | 8 | 4 | 12 |  | 513 | 237 | 789 |  | 303 | 127 | 479 |  | 16 | 7 | 24 |
|  |  | Self-inflicted injuries | 0 | 0 | 0 |  | 93 | 27 | 160 |  | 116 | 33 | 199 |  | 3 | 1 | 5 |
|  |  | Violence | 8 | 4 | 12 |  | 419 | 210 | 629 |  | 187 | 94 | 280 |  | 13 | 6 | 19 |
|  |  | Other intentional injuries | 0 | 0 | 0 |  | 0 | 0 | 0 |  | 0 | 0 | 0 |  | 0 | 0 | 0 |

Table 12: Deaths from injuries attributable to alcohol consumption (with harms to others): Latin America Central

|  |  |  | 0 to 14 years of age | | |  | 15 to 34 years of age | | |  | 35 to 64 years of age | | |  | 65 years of age and older | | |
| --- | --- | --- | --- | --- | --- | --- | --- | --- | --- | --- | --- | --- | --- | --- | --- | --- | --- |
|  |  |  | Point estimate | Lower 95% CI | Upper 95% CI |  | Point estimate | Lower 95% CI | Upper 95% CI |  | Point estimate | Lower 95% CI | Upper 95% CI |  | Point estimate | Lower 95% CI | Upper 95% CI |
| Women | |  |  |  |  |  |  |  |  |  |  |  |  |  |  |  |  |
| Injuries | |  | 350 | 141 | 558 |  | 2061 | 1059 | 3116 |  | 1281 | 641 | 1944 |  | 314 | 111 | 516 |
|  | Unintentional injuries | | 296 | 109 | 484 |  | 1200 | 588 | 1838 |  | 971 | 466 | 1492 |  | 290 | 98 | 483 |
|  |  | Transport injuries | 296 | 109 | 484 |  | 1132 | 588 | 1676 |  | 923 | 466 | 1380 |  | 260 | 96 | 423 |
|  |  | Poisonings | 0 | 0 | 0 |  | 8 | 0 | 20 |  | 4 | 0 | 8 |  | 1 | 0 | 3 |
|  |  | Falls | 0 | 0 | 0 |  | 5 | 0 | 12 |  | 9 | 0 | 20 |  | 11 | 1 | 21 |
|  |  | Fires, heat and hot substances | 0 | 0 | 0 |  | 3 | 0 | 8 |  | 3 | 0 | 7 |  | 1 | 0 | 2 |
|  |  | Drownings | 0 | 0 | 0 |  | 15 | 0 | 35 |  | 4 | 0 | 10 |  | 1 | 0 | 1 |
|  |  | Other unintentional injuries | 0 | 0 | 0 |  | 37 | 0 | 88 |  | 29 | 0 | 68 |  | 16 | 1 | 32 |
|  | Intentional injuries | | 53 | 32 | 75 |  | 860 | 471 | 1278 |  | 310 | 174 | 452 |  | 23 | 13 | 33 |
|  |  | Self-inflicted injuries | 0 | 0 | 0 |  | 74 | 0 | 175 |  | 19 | 0 | 44 |  | 1 | 0 | 2 |
|  |  | Violence | 53 | 32 | 75 |  | 785 | 471 | 1099 |  | 291 | 174 | 407 |  | 22 | 13 | 31 |
|  |  | Other intentional injuries | 0 | 0 | 0 |  | 2 | 0 | 4 |  | 0 | 0 | 0 |  | 0 | 0 | 0 |
| Men |  |  |  |  |  |  |  |  |  |  |  |  |  |  |  |  |  |
| Injuries | |  | 599 | 543 | 655 |  | 20953 | 13209 | 28697 |  | 14085 | 8830 | 19341 |  | 2415 | 1285 | 3544 |
|  | Unintentional injuries | | 510 | 489 | 530 |  | 9276 | 6466 | 12086 |  | 9720 | 6403 | 13037 |  | 2119 | 1147 | 3092 |
|  |  | Transport injuries | 510 | 489 | 530 |  | 7373 | 5692 | 9054 |  | 7050 | 5263 | 8836 |  | 1184 | 811 | 1557 |
|  |  | Poisonings | 0 | 0 | 0 |  | 108 | 44 | 172 |  | 147 | 63 | 231 |  | 40 | 14 | 66 |
|  |  | Falls | 0 | 0 | 0 |  | 223 | 91 | 356 |  | 605 | 258 | 952 |  | 316 | 113 | 519 |
|  |  | Fires, heat and hot substances | 0 | 0 | 0 |  | 49 | 20 | 78 |  | 90 | 38 | 141 |  | 39 | 14 | 65 |
|  |  | Drownings | 0 | 0 | 0 |  | 548 | 223 | 873 |  | 420 | 179 | 662 |  | 75 | 27 | 123 |
|  |  | Other unintentional injuries | 0 | 0 | 0 |  | 975 | 397 | 1554 |  | 1409 | 601 | 2216 |  | 464 | 167 | 762 |
|  | Intentional injuries | | 89 | 53 | 124 |  | 11677 | 6743 | 16611 |  | 4365 | 2426 | 6304 |  | 295 | 138 | 452 |
|  |  | Self-inflicted injuries | 0 | 0 | 0 |  | 1168 | 475 | 1860 |  | 1064 | 454 | 1674 |  | 162 | 58 | 265 |
|  |  | Violence | 89 | 53 | 124 |  | 10328 | 6194 | 14462 |  | 3258 | 1954 | 4562 |  | 133 | 80 | 186 |
|  |  | Other intentional injuries | 0 | 0 | 0 |  | 181 | 74 | 289 |  | 43 | 18 | 68 |  | 1 | 0 | 1 |

Table 13: Deaths from injuries attributable to alcohol consumption (with harms to others): Latin America Southern

|  |  |  | 0 to 14 years of age | | |  | 15 to 34 years of age | | |  | 35 to 64 years of age | | |  | 65 years of age and older | | |
| --- | --- | --- | --- | --- | --- | --- | --- | --- | --- | --- | --- | --- | --- | --- | --- | --- | --- |
|  |  |  | Point estimate | Lower 95% CI | Upper 95% CI |  | Point estimate | Lower 95% CI | Upper 95% CI |  | Point estimate | Lower 95% CI | Upper 95% CI |  | Point estimate | Lower 95% CI | Upper 95% CI |
| Women | |  |  |  |  |  |  |  |  |  |  |  |  |  |  |  |  |
| Injuries | |  | 13 | 5 | 20 |  | 133 | 56 | 209 |  | 160 | 32 | 298 |  | 80 | 22 | 138 |
|  | Unintentional injuries | | 8 | 2 | 14 |  | 75 | 27 | 123 |  | 99 | 16 | 189 |  | 70 | 18 | 123 |
|  |  | Transport injuries | 8 | 2 | 14 |  | 57 | 22 | 92 |  | 47 | 16 | 78 |  | 8 | 2 | 15 |
|  |  | Poisonings | 0 | 0 | 0 |  | 1 | 0 | 2 |  | 2 | 0 | 4 |  | 1 | 0 | 2 |
|  |  | Falls | 0 | 0 | 0 |  | 0 | 0 | 1 |  | 2 | 0 | 4 |  | 11 | 3 | 19 |
|  |  | Fires, heat and hot substances | 0 | 0 | 0 |  | 2 | 0 | 3 |  | 5 | 0 | 11 |  | 3 | 1 | 5 |
|  |  | Drownings | 0 | 0 | 0 |  | 1 | 0 | 2 |  | 3 | 0 | 7 |  | 1 | 0 | 1 |
|  |  | Other unintentional injuries | 0 | 0 | 0 |  | 13 | 3 | 23 |  | 40 | 0 | 84 |  | 46 | 11 | 81 |
|  | Intentional injuries | | 5 | 3 | 6 |  | 58 | 30 | 86 |  | 61 | 16 | 110 |  | 10 | 5 | 15 |
|  |  | Self-inflicted injuries | 0 | 0 | 0 |  | 18 | 5 | 32 |  | 35 | 0 | 75 |  | 5 | 1 | 8 |
|  |  | Violence | 5 | 3 | 6 |  | 40 | 25 | 54 |  | 25 | 16 | 35 |  | 5 | 3 | 7 |
|  |  | Other intentional injuries | 0 | 0 | 0 |  | 0 | 0 | 0 |  | 0 | 0 | 0 |  | 0 | 0 | 0 |
| Men |  |  |  |  |  |  |  |  |  |  |  |  |  |  |  |  |  |
| Injuries | |  | 18 | 16 | 20 |  | 1704 | 906 | 2501 |  | 1627 | 790 | 2465 |  | 534 | 210 | 858 |
|  | Unintentional injuries | | 13 | 13 | 14 |  | 838 | 463 | 1213 |  | 1018 | 510 | 1526 |  | 395 | 155 | 635 |
|  |  | Transport injuries | 13 | 13 | 14 |  | 334 | 257 | 410 |  | 326 | 236 | 417 |  | 45 | 30 | 59 |
|  |  | Poisonings | 0 | 0 | 0 |  | 12 | 5 | 19 |  | 16 | 6 | 25 |  | 6 | 2 | 11 |
|  |  | Falls | 0 | 0 | 0 |  | 26 | 11 | 41 |  | 57 | 22 | 91 |  | 47 | 17 | 77 |
|  |  | Fires, heat and hot substances | 0 | 0 | 0 |  | 26 | 10 | 41 |  | 40 | 16 | 64 |  | 26 | 9 | 43 |
|  |  | Drownings | 0 | 0 | 0 |  | 86 | 35 | 136 |  | 78 | 31 | 126 |  | 16 | 6 | 27 |
|  |  | Other unintentional injuries | 0 | 0 | 0 |  | 356 | 145 | 566 |  | 501 | 198 | 803 |  | 255 | 91 | 418 |
|  | Intentional injuries | | 5 | 3 | 7 |  | 866 | 444 | 1289 |  | 609 | 280 | 939 |  | 139 | 55 | 223 |
|  |  | Self-inflicted injuries | 0 | 0 | 0 |  | 462 | 188 | 735 |  | 445 | 176 | 714 |  | 122 | 44 | 200 |
|  |  | Violence | 5 | 3 | 7 |  | 404 | 255 | 553 |  | 164 | 104 | 225 |  | 17 | 11 | 24 |
|  |  | Other intentional injuries | 0 | 0 | 0 |  | 1 | 0 | 1 |  | 0 | 0 | 0 |  | 0 | 0 | 0 |

Table 14: Deaths from injuries attributable to alcohol consumption (with harms to others): Latin America Tropical

|  |  |  | 0 to 14 years of age | | |  | 15 to 34 years of age | | |  | 35 to 64 years of age | | |  | 65 years of age and older | | |
| --- | --- | --- | --- | --- | --- | --- | --- | --- | --- | --- | --- | --- | --- | --- | --- | --- | --- |
|  |  |  | Point estimate | Lower 95% CI | Upper 95% CI |  | Point estimate | Lower 95% CI | Upper 95% CI |  | Point estimate | Lower 95% CI | Upper 95% CI |  | Point estimate | Lower 95% CI | Upper 95% CI |
| Women | |  |  |  |  |  |  |  |  |  |  |  |  |  |  |  |  |
| Injuries | |  | 130 | 36 | 224 |  | 1697 | 718 | 2695 |  | 1100 | 293 | 1951 |  | 131 | 18 | 244 |
|  | Unintentional injuries | | 88 | 12 | 164 |  | 715 | 195 | 1246 |  | 662 | 98 | 1254 |  | 106 | 5 | 207 |
|  |  | Transport injuries | 88 | 12 | 164 |  | 644 | 195 | 1093 |  | 504 | 98 | 910 |  | 68 | 4 | 131 |
|  |  | Poisonings | 0 | 0 | 0 |  | 2 | 0 | 4 |  | 2 | 0 | 5 |  | 0 | 0 | 0 |
|  |  | Falls | 0 | 0 | 0 |  | 6 | 0 | 14 |  | 33 | 0 | 72 |  | 22 | 0 | 44 |
|  |  | Fires, heat and hot substances | 0 | 0 | 0 |  | 9 | 0 | 20 |  | 18 | 0 | 40 |  | 1 | 0 | 3 |
|  |  | Drownings | 0 | 0 | 0 |  | 19 | 0 | 40 |  | 23 | 0 | 50 |  | 1 | 0 | 1 |
|  |  | Other unintentional injuries | 0 | 0 | 0 |  | 35 | 0 | 75 |  | 81 | 0 | 177 |  | 14 | 0 | 27 |
|  | Intentional injuries | | 41 | 23 | 59 |  | 982 | 523 | 1449 |  | 438 | 196 | 697 |  | 25 | 13 | 37 |
|  |  | Self-inflicted injuries | 0 | 0 | 0 |  | 60 | 0 | 128 |  | 94 | 0 | 203 |  | 2 | 0 | 4 |
|  |  | Violence | 41 | 23 | 59 |  | 922 | 523 | 1321 |  | 345 | 196 | 494 |  | 23 | 13 | 33 |
|  |  | Other intentional injuries | 0 | 0 | 0 |  | 0 | 0 | 0 |  | 0 | 0 | 0 |  | 0 | 0 | 0 |
| Men |  |  |  |  |  |  |  |  |  |  |  |  |  |  |  |  |  |
| Injuries | |  | 236 | 195 | 278 |  | 21368 | 12037 | 30700 |  | 11592 | 5487 | 17698 |  | 615 | 305 | 925 |
|  | Unintentional injuries | | 154 | 148 | 159 |  | 7637 | 4475 | 10799 |  | 6932 | 3108 | 10756 |  | 437 | 220 | 654 |
|  |  | Transport injuries | 154 | 148 | 159 |  | 4912 | 3402 | 6421 |  | 3803 | 1997 | 5608 |  | 187 | 155 | 218 |
|  |  | Poisonings | 0 | 0 | 0 |  | 26 | 10 | 42 |  | 30 | 11 | 49 |  | 1 | 0 | 2 |
|  |  | Falls | 0 | 0 | 0 |  | 351 | 138 | 564 |  | 878 | 312 | 1444 |  | 119 | 31 | 207 |
|  |  | Fires, heat and hot substances | 0 | 0 | 0 |  | 91 | 36 | 146 |  | 123 | 44 | 202 |  | 9 | 2 | 16 |
|  |  | Drownings | 0 | 0 | 0 |  | 1018 | 401 | 1636 |  | 713 | 253 | 1172 |  | 18 | 5 | 31 |
|  |  | Other unintentional injuries | 0 | 0 | 0 |  | 1238 | 487 | 1989 |  | 1386 | 492 | 2280 |  | 103 | 27 | 179 |
|  | Intentional injuries | | 83 | 47 | 119 |  | 13731 | 7562 | 19901 |  | 4660 | 2379 | 6942 |  | 178 | 85 | 272 |
|  |  | Self-inflicted injuries | 0 | 0 | 0 |  | 1135 | 447 | 1824 |  | 1232 | 437 | 2027 |  | 53 | 14 | 92 |
|  |  | Violence | 83 | 47 | 119 |  | 12404 | 7040 | 17768 |  | 3410 | 1936 | 4885 |  | 126 | 71 | 180 |
|  |  | Other intentional injuries | 0 | 0 | 0 |  | 192 | 76 | 309 |  | 18 | 6 | 30 |  | 0 | 0 | 0 |

Table 15: Deaths from injuries attributable to alcohol consumption (with harms to others): North Africa Middle East

|  |  |  | 0 to 14 years of age | | |  | 15 to 34 years of age | | |  | 35 to 64 years of age | | |  | 65 years of age and older | | |
| --- | --- | --- | --- | --- | --- | --- | --- | --- | --- | --- | --- | --- | --- | --- | --- | --- | --- |
|  |  |  | Point estimate | Lower 95% CI | Upper 95% CI |  | Point estimate | Lower 95% CI | Upper 95% CI |  | Point estimate | Lower 95% CI | Upper 95% CI |  | Point estimate | Lower 95% CI | Upper 95% CI |
| Women | |  |  |  |  |  |  |  |  |  |  |  |  |  |  |  |  |
| Injuries | |  | 108 | 0 | 366 |  | 336 | 0 | 1402 |  | 200 | 0 | 903 |  | 28 | 0 | 165 |
|  | Unintentional injuries | | 103 | 0 | 346 |  | 247 | 0 | 1024 |  | 169 | 0 | 746 |  | 25 | 0 | 149 |
|  |  | Transport injuries | 103 | 0 | 346 |  | 208 | 0 | 761 |  | 153 | 0 | 509 |  | 23 | 0 | 79 |
|  |  | Poisonings | 0 | 0 | 0 |  | 5 | 0 | 31 |  | 2 | 0 | 23 |  | 0 | 0 | 5 |
|  |  | Falls | 0 | 0 | 0 |  | 2 | 0 | 15 |  | 3 | 0 | 44 |  | 1 | 0 | 25 |
|  |  | Fires, heat and hot substances | 0 | 0 | 0 |  | 10 | 0 | 68 |  | 3 | 0 | 41 |  | 0 | 0 | 8 |
|  |  | Drownings | 0 | 0 | 0 |  | 4 | 0 | 26 |  | 1 | 0 | 14 |  | 0 | 0 | 2 |
|  |  | Other unintentional injuries | 0 | 0 | 0 |  | 19 | 0 | 124 |  | 8 | 0 | 115 |  | 1 | 0 | 29 |
|  | Intentional injuries | | 6 | 0 | 20 |  | 89 | 0 | 378 |  | 31 | 0 | 157 |  | 3 | 0 | 16 |
|  |  | Self-inflicted injuries | 0 | 0 | 0 |  | 21 | 0 | 137 |  | 4 | 0 | 63 |  | 0 | 0 | 5 |
|  |  | Violence | 6 | 0 | 20 |  | 68 | 0 | 237 |  | 26 | 0 | 92 |  | 3 | 0 | 11 |
|  |  | Other intentional injuries | 0 | 0 | 0 |  | 1 | 0 | 3 |  | 0 | 0 | 1 |  | 0 | 0 | 1 |
| Men |  |  |  |  |  |  |  |  |  |  |  |  |  |  |  |  |  |
| Injuries | |  | 188 | 172 | 218 |  | 5568 | 939 | 15971 |  | 1689 | 534 | 3355 |  | 82 | 61 | 115 |
|  | Unintentional injuries | | 179 | 172 | 187 |  | 4654 | 927 | 13676 |  | 1445 | 534 | 2691 |  | 73 | 61 | 86 |
|  |  | Transport injuries | 179 | 172 | 187 |  | 2460 | 883 | 9333 |  | 806 | 534 | 1320 |  | 67 | 60 | 75 |
|  |  | Poisonings | 0 | 0 | 0 |  | 223 | 4 | 441 |  | 96 | 0 | 206 |  | 0 | 0 | 1 |
|  |  | Falls | 0 | 0 | 0 |  | 214 | 4 | 423 |  | 128 | 0 | 275 |  | 2 | 0 | 4 |
|  |  | Fires, heat and hot substances | 0 | 0 | 0 |  | 150 | 3 | 297 |  | 32 | 0 | 68 |  | 0 | 0 | 1 |
|  |  | Drownings | 0 | 0 | 0 |  | 512 | 10 | 1013 |  | 62 | 0 | 134 |  | 0 | 0 | 1 |
|  |  | Other unintentional injuries | 0 | 0 | 0 |  | 1095 | 22 | 2168 |  | 321 | 0 | 689 |  | 3 | 1 | 6 |
|  | Intentional injuries | | 9 | 0 | 31 |  | 914 | 12 | 2295 |  | 244 | 0 | 664 |  | 9 | 0 | 29 |
|  |  | Self-inflicted injuries | 0 | 0 | 0 |  | 518 | 10 | 1025 |  | 118 | 0 | 252 |  | 1 | 0 | 1 |
|  |  | Violence | 9 | 0 | 31 |  | 323 | 0 | 1126 |  | 106 | 0 | 370 |  | 8 | 0 | 27 |
|  |  | Other intentional injuries | 0 | 0 | 0 |  | 73 | 1 | 144 |  | 20 | 0 | 42 |  | 0 | 0 | 0 |

Table 16: Deaths from injuries attributable to alcohol consumption (with harms to others): North America [high Income]

|  |  |  | 0 to 14 years of age | | |  | 15 to 34 years of age | | |  | 35 to 64 years of age | | |  | 65 years of age and older | | |
| --- | --- | --- | --- | --- | --- | --- | --- | --- | --- | --- | --- | --- | --- | --- | --- | --- | --- |
|  |  |  | Point estimate | Lower 95% CI | Upper 95% CI |  | Point estimate | Lower 95% CI | Upper 95% CI |  | Point estimate | Lower 95% CI | Upper 95% CI |  | Point estimate | Lower 95% CI | Upper 95% CI |
| Women | |  |  |  |  |  |  |  |  |  |  |  |  |  |  |  |  |
| Injuries | |  | 107 | 41 | 172 |  | 1700 | 673 | 2727 |  | 1594 | 586 | 2603 |  | 446 | 146 | 747 |
|  | Unintentional injuries | | 57 | 9 | 105 |  | 1077 | 333 | 1821 |  | 1037 | 310 | 1764 |  | 400 | 120 | 680 |
|  |  | Transport injuries | 57 | 9 | 105 |  | 854 | 268 | 1439 |  | 570 | 151 | 989 |  | 116 | 19 | 214 |
|  |  | Poisonings | 0 | 0 | 0 |  | 158 | 46 | 270 |  | 282 | 96 | 467 |  | 9 | 3 | 15 |
|  |  | Falls | 0 | 0 | 0 |  | 10 | 3 | 18 |  | 53 | 18 | 87 |  | 141 | 50 | 231 |
|  |  | Fires, heat and hot substances | 0 | 0 | 0 |  | 14 | 4 | 25 |  | 26 | 9 | 43 |  | 9 | 3 | 15 |
|  |  | Drownings | 0 | 0 | 0 |  | 10 | 3 | 17 |  | 14 | 5 | 23 |  | 2 | 1 | 4 |
|  |  | Other unintentional injuries | 0 | 0 | 0 |  | 31 | 9 | 53 |  | 94 | 32 | 155 |  | 123 | 44 | 202 |
|  | Intentional injuries | | 49 | 32 | 67 |  | 623 | 340 | 906 |  | 557 | 275 | 839 |  | 46 | 25 | 67 |
|  |  | Self-inflicted injuries | 0 | 0 | 0 |  | 170 | 49 | 291 |  | 274 | 93 | 454 |  | 15 | 6 | 25 |
|  |  | Violence | 49 | 32 | 67 |  | 453 | 291 | 615 |  | 283 | 182 | 384 |  | 31 | 20 | 42 |
|  |  | Other intentional injuries | 0 | 0 | 0 |  | 0 | 0 | 0 |  | 0 | 0 | 1 |  | 0 | 0 | 0 |
| Men |  |  |  |  |  |  |  |  |  |  |  |  |  |  |  |  |  |
| Injuries | |  | 137 | 113 | 161 |  | 12823 | 6965 | 18681 |  | 10390 | 5181 | 15599 |  | 2493 | 1032 | 3955 |
|  | Unintentional injuries | | 74 | 73 | 76 |  | 6987 | 3843 | 10130 |  | 6096 | 3161 | 9032 |  | 1916 | 804 | 3029 |
|  |  | Transport injuries | 74 | 73 | 76 |  | 3485 | 2227 | 4744 |  | 1837 | 1332 | 2342 |  | 231 | 173 | 290 |
|  |  | Poisonings | 0 | 0 | 0 |  | 1932 | 892 | 2972 |  | 2066 | 887 | 3245 |  | 58 | 22 | 94 |
|  |  | Falls | 0 | 0 | 0 |  | 238 | 110 | 366 |  | 622 | 267 | 977 |  | 815 | 305 | 1324 |
|  |  | Fires, heat and hot substances | 0 | 0 | 0 |  | 120 | 55 | 184 |  | 195 | 84 | 307 |  | 64 | 24 | 103 |
|  |  | Drownings | 0 | 0 | 0 |  | 364 | 168 | 559 |  | 237 | 102 | 373 |  | 36 | 14 | 59 |
|  |  | Other unintentional injuries | 0 | 0 | 0 |  | 848 | 391 | 1304 |  | 1139 | 489 | 1789 |  | 713 | 267 | 1159 |
|  | Intentional injuries | | 63 | 40 | 85 |  | 5836 | 3121 | 8552 |  | 4294 | 2021 | 6567 |  | 577 | 228 | 926 |
|  |  | Self-inflicted injuries | 0 | 0 | 0 |  | 3404 | 1572 | 5236 |  | 3421 | 1469 | 5373 |  | 532 | 199 | 865 |
|  |  | Violence | 63 | 40 | 85 |  | 2363 | 1517 | 3209 |  | 832 | 534 | 1129 |  | 44 | 28 | 59 |
|  |  | Other intentional injuries | 0 | 0 | 0 |  | 70 | 32 | 107 |  | 41 | 18 | 65 |  | 1 | 0 | 2 |

Table 17: Deaths from injuries attributable to alcohol consumption (with harms to others): Oceania

|  |  |  | 0 to 14 years of age | | |  | 15 to 34 years of age | | |  | 35 to 64 years of age | | |  | 65 years of age and older | | |
| --- | --- | --- | --- | --- | --- | --- | --- | --- | --- | --- | --- | --- | --- | --- | --- | --- | --- |
|  |  |  | Point estimate | Lower 95% CI | Upper 95% CI |  | Point estimate | Lower 95% CI | Upper 95% CI |  | Point estimate | Lower 95% CI | Upper 95% CI |  | Point estimate | Lower 95% CI | Upper 95% CI |
| Women | |  |  |  |  |  |  |  |  |  |  |  |  |  |  |  |  |
| Injuries | |  | 7 | 2 | 11 |  | 38 | 16 | 60 |  | 25 | 10 | 41 |  | 3 | 1 | 5 |
|  | Unintentional injuries | | 6 | 2 | 10 |  | 27 | 10 | 43 |  | 19 | 6 | 31 |  | 2 | 1 | 4 |
|  |  | Transport injuries | 6 | 2 | 10 |  | 11 | 5 | 18 |  | 13 | 5 | 21 |  | 1 | 0 | 2 |
|  |  | Poisonings | 0 | 0 | 0 |  | 1 | 0 | 2 |  | 1 | 0 | 2 |  | 0 | 0 | 0 |
|  |  | Falls | 0 | 0 | 0 |  | 1 | 0 | 1 |  | 1 | 0 | 1 |  | 0 | 0 | 1 |
|  |  | Fires, heat and hot substances | 0 | 0 | 0 |  | 7 | 3 | 12 |  | 2 | 1 | 4 |  | 0 | 0 | 0 |
|  |  | Drownings | 0 | 0 | 0 |  | 2 | 1 | 3 |  | 1 | 0 | 1 |  | 0 | 0 | 0 |
|  |  | Other unintentional injuries | 0 | 0 | 0 |  | 5 | 2 | 8 |  | 2 | 0 | 3 |  | 0 | 0 | 1 |
|  | Intentional injuries | | 1 | 1 | 1 |  | 12 | 6 | 17 |  | 6 | 3 | 9 |  | 1 | 0 | 1 |
|  |  | Self-inflicted injuries | 0 | 0 | 0 |  | 6 | 2 | 9 |  | 2 | 0 | 3 |  | 0 | 0 | 0 |
|  |  | Violence | 1 | 1 | 1 |  | 6 | 4 | 8 |  | 4 | 3 | 6 |  | 0 | 0 | 0 |
|  |  | Other intentional injuries | 0 | 0 | 0 |  | 0 | 0 | 0 |  | 0 | 0 | 0 |  | 0 | 0 | 0 |
| Men |  |  |  |  |  |  |  |  |  |  |  |  |  |  |  |  |  |
| Injuries | |  | 11 | 10 | 11 |  | 241 | 154 | 328 |  | 163 | 103 | 224 |  | 15 | 8 | 21 |
|  | Unintentional injuries | | 9 | 9 | 9 |  | 164 | 107 | 220 |  | 120 | 77 | 164 |  | 12 | 7 | 17 |
|  |  | Transport injuries | 9 | 9 | 9 |  | 96 | 75 | 117 |  | 68 | 53 | 83 |  | 5 | 4 | 7 |
|  |  | Poisonings | 0 | 0 | 0 |  | 7 | 3 | 10 |  | 9 | 4 | 14 |  | 1 | 0 | 1 |
|  |  | Falls | 0 | 0 | 0 |  | 5 | 2 | 8 |  | 7 | 3 | 10 |  | 3 | 1 | 4 |
|  |  | Fires, heat and hot substances | 0 | 0 | 0 |  | 7 | 3 | 11 |  | 6 | 3 | 9 |  | 0 | 0 | 1 |
|  |  | Drownings | 0 | 0 | 0 |  | 14 | 7 | 22 |  | 8 | 4 | 12 |  | 1 | 0 | 1 |
|  |  | Other unintentional injuries | 0 | 0 | 0 |  | 35 | 16 | 53 |  | 23 | 11 | 36 |  | 2 | 1 | 3 |
|  | Intentional injuries | | 1 | 1 | 2 |  | 77 | 47 | 107 |  | 43 | 26 | 60 |  | 3 | 1 | 4 |
|  |  | Self-inflicted injuries | 0 | 0 | 0 |  | 25 | 12 | 38 |  | 15 | 7 | 23 |  | 2 | 1 | 2 |
|  |  | Violence | 1 | 1 | 2 |  | 51 | 34 | 68 |  | 27 | 18 | 36 |  | 1 | 1 | 1 |
|  |  | Other intentional injuries | 0 | 0 | 0 |  | 1 | 0 | 2 |  | 1 | 0 | 1 |  | 0 | 0 | 0 |

Table 18: Deaths from injuries attributable to alcohol consumption (with harms to others): Sub-Saharan Africa Central

|  |  |  | 0 to 14 years of age | | |  | 15 to 34 years of age | | |  | 35 to 64 years of age | | |  | 65 years of age and older | | |
| --- | --- | --- | --- | --- | --- | --- | --- | --- | --- | --- | --- | --- | --- | --- | --- | --- | --- |
|  |  |  | Point estimate | Lower 95% CI | Upper 95% CI |  | Point estimate | Lower 95% CI | Upper 95% CI |  | Point estimate | Lower 95% CI | Upper 95% CI |  | Point estimate | Lower 95% CI | Upper 95% CI |
| Women | |  |  |  |  |  |  |  |  |  |  |  |  |  |  |  |  |
| Injuries | |  | 336 | 110 | 563 |  | 609 | 302 | 917 |  | 415 | 170 | 659 |  | 49 | 16 | 81 |
|  | Unintentional injuries | | 299 | 85 | 513 |  | 358 | 145 | 571 |  | 309 | 113 | 504 |  | 39 | 11 | 66 |
|  |  | Transport injuries | 299 | 85 | 513 |  | 269 | 114 | 424 |  | 223 | 96 | 350 |  | 27 | 8 | 47 |
|  |  | Poisonings | 0 | 0 | 0 |  | 24 | 8 | 40 |  | 24 | 5 | 43 |  | 3 | 1 | 6 |
|  |  | Falls | 0 | 0 | 0 |  | 3 | 1 | 4 |  | 5 | 1 | 9 |  | 3 | 1 | 4 |
|  |  | Fires, heat and hot substances | 0 | 0 | 0 |  | 9 | 3 | 15 |  | 11 | 2 | 19 |  | 2 | 0 | 3 |
|  |  | Drownings | 0 | 0 | 0 |  | 27 | 9 | 45 |  | 16 | 3 | 29 |  | 1 | 0 | 1 |
|  |  | Other unintentional injuries | 0 | 0 | 0 |  | 26 | 9 | 43 |  | 30 | 6 | 54 |  | 3 | 1 | 5 |
|  | Intentional injuries | | 37 | 25 | 50 |  | 251 | 157 | 346 |  | 106 | 57 | 155 |  | 10 | 5 | 15 |
|  |  | Self-inflicted injuries | 0 | 0 | 0 |  | 35 | 12 | 58 |  | 29 | 6 | 52 |  | 3 | 1 | 6 |
|  |  | Violence | 37 | 25 | 50 |  | 217 | 145 | 288 |  | 77 | 52 | 103 |  | 7 | 4 | 9 |
|  |  | Other intentional injuries | 0 | 0 | 0 |  | 0 | 0 | 0 |  | 0 | 0 | 0 |  | 0 | 0 | 0 |
| Men |  |  |  |  |  |  |  |  |  |  |  |  |  |  |  |  |  |
| Injuries | |  | 541 | 513 | 568 |  | 5194 | 3345 | 7043 |  | 3612 | 2233 | 4991 |  | 150 | 93 | 207 |
|  | Unintentional injuries | | 487 | 477 | 497 |  | 3050 | 1974 | 4126 |  | 2736 | 1716 | 3756 |  | 115 | 73 | 157 |
|  |  | Transport injuries | 487 | 477 | 497 |  | 1740 | 1397 | 2082 |  | 1621 | 1247 | 1994 |  | 71 | 54 | 88 |
|  |  | Poisonings | 0 | 0 | 0 |  | 160 | 71 | 250 |  | 185 | 78 | 293 |  | 2 | 1 | 4 |
|  |  | Falls | 0 | 0 | 0 |  | 38 | 17 | 60 |  | 73 | 31 | 115 |  | 9 | 4 | 14 |
|  |  | Fires, heat and hot substances | 0 | 0 | 0 |  | 26 | 12 | 41 |  | 41 | 17 | 65 |  | 4 | 2 | 6 |
|  |  | Drownings | 0 | 0 | 0 |  | 303 | 133 | 473 |  | 405 | 170 | 640 |  | 3 | 1 | 5 |
|  |  | Other unintentional injuries | 0 | 0 | 0 |  | 783 | 345 | 1220 |  | 411 | 173 | 649 |  | 26 | 11 | 40 |
|  | Intentional injuries | | 53 | 36 | 71 |  | 2144 | 1370 | 2918 |  | 876 | 517 | 1235 |  | 35 | 20 | 51 |
|  |  | Self-inflicted injuries | 0 | 0 | 0 |  | 274 | 121 | 428 |  | 275 | 116 | 434 |  | 15 | 6 | 23 |
|  |  | Violence | 53 | 36 | 71 |  | 1870 | 1250 | 2490 |  | 601 | 402 | 800 |  | 21 | 14 | 27 |
|  |  | Other intentional injuries | 0 | 0 | 0 |  | 0 | 0 | 0 |  | 0 | 0 | 0 |  | 0 | 0 | 0 |

Table 19: Deaths from injuries attributable to alcohol consumption (with harms to others): Sub-Saharan Africa East

|  |  |  | 0 to 14 years of age | | |  | 15 to 34 years of age | | |  | 35 to 64 years of age | | |  | 65 years of age and older | | |
| --- | --- | --- | --- | --- | --- | --- | --- | --- | --- | --- | --- | --- | --- | --- | --- | --- | --- |
|  |  |  | Point estimate | Lower 95% CI | Upper 95% CI |  | Point estimate | Lower 95% CI | Upper 95% CI |  | Point estimate | Lower 95% CI | Upper 95% CI |  | Point estimate | Lower 95% CI | Upper 95% CI |
| Women | |  |  |  |  |  |  |  |  |  |  |  |  |  |  |  |  |
| Injuries | |  | 979 | 87 | 1961 |  | 2042 | 537 | 3714 |  | 1820 | 195 | 3797 |  | 243 | 31 | 525 |
|  | Unintentional injuries | | 794 | 0 | 1677 |  | 922 | 39 | 1929 |  | 1246 | 0 | 2763 |  | 182 | 14 | 407 |
|  |  | Transport injuries | 794 | 0 | 1677 |  | 770 | 39 | 1523 |  | 804 | 0 | 1656 |  | 101 | 14 | 202 |
|  |  | Poisonings | 0 | 0 | 0 |  | 31 | 0 | 82 |  | 97 | 0 | 243 |  | 19 | 0 | 47 |
|  |  | Falls | 0 | 0 | 0 |  | 7 | 0 | 19 |  | 31 | 0 | 77 |  | 19 | 0 | 47 |
|  |  | Fires, heat and hot substances | 0 | 0 | 0 |  | 18 | 0 | 49 |  | 56 | 0 | 140 |  | 12 | 0 | 31 |
|  |  | Drownings | 0 | 0 | 0 |  | 30 | 0 | 81 |  | 63 | 0 | 157 |  | 4 | 0 | 10 |
|  |  | Other unintentional injuries | 0 | 0 | 0 |  | 65 | 0 | 174 |  | 195 | 0 | 489 |  | 28 | 0 | 70 |
|  | Intentional injuries | | 185 | 87 | 283 |  | 1120 | 498 | 1785 |  | 574 | 195 | 1035 |  | 61 | 18 | 118 |
|  |  | Self-inflicted injuries | 0 | 0 | 0 |  | 64 | 0 | 171 |  | 161 | 0 | 404 |  | 24 | 0 | 61 |
|  |  | Violence | 185 | 87 | 283 |  | 1055 | 498 | 1613 |  | 413 | 195 | 630 |  | 37 | 18 | 57 |
|  |  | Other intentional injuries | 0 | 0 | 0 |  | 0 | 0 | 1 |  | 0 | 0 | 1 |  | 0 | 0 | 0 |
| Men |  |  |  |  |  |  |  |  |  |  |  |  |  |  |  |  |  |
| Injuries | |  | 1380 | 1210 | 1550 |  | 13054 | 6294 | 19815 |  | 15727 | 4904 | 26705 |  | 1265 | 451 | 2079 |
|  | Unintentional injuries | | 1151 | 1102 | 1200 |  | 6205 | 3212 | 9197 |  | 11590 | 3303 | 20032 |  | 927 | 342 | 1511 |
|  |  | Transport injuries | 1151 | 1102 | 1200 |  | 3178 | 2437 | 3919 |  | 4697 | 1260 | 8290 |  | 311 | 192 | 430 |
|  |  | Poisonings | 0 | 0 | 0 |  | 352 | 90 | 614 |  | 1095 | 324 | 1865 |  | 24 | 6 | 43 |
|  |  | Falls | 0 | 0 | 0 |  | 126 | 32 | 220 |  | 582 | 173 | 992 |  | 131 | 32 | 230 |
|  |  | Fires, heat and hot substances | 0 | 0 | 0 |  | 89 | 23 | 155 |  | 308 | 91 | 525 |  | 56 | 14 | 98 |
|  |  | Drownings | 0 | 0 | 0 |  | 651 | 167 | 1136 |  | 1792 | 531 | 3053 |  | 33 | 8 | 58 |
|  |  | Other unintentional injuries | 0 | 0 | 0 |  | 1808 | 463 | 3154 |  | 3115 | 923 | 5307 |  | 371 | 90 | 653 |
|  | Intentional injuries | | 229 | 108 | 349 |  | 6849 | 3081 | 10617 |  | 4137 | 1602 | 6673 |  | 338 | 109 | 568 |
|  |  | Self-inflicted injuries | 0 | 0 | 0 |  | 688 | 176 | 1200 |  | 1981 | 587 | 3374 |  | 220 | 53 | 387 |
|  |  | Violence | 229 | 108 | 349 |  | 6151 | 2902 | 9400 |  | 2139 | 1009 | 3269 |  | 115 | 54 | 176 |
|  |  | Other intentional injuries | 0 | 0 | 0 |  | 10 | 3 | 18 |  | 17 | 5 | 30 |  | 3 | 1 | 5 |

Table 20: Deaths from injuries attributable to alcohol consumption (with harms to others): Sub-Saharan Africa Southern

|  |  |  | 0 to 14 years of age | | |  | 15 to 34 years of age | | |  | 35 to 64 years of age | | |  | 65 years of age and older | | |
| --- | --- | --- | --- | --- | --- | --- | --- | --- | --- | --- | --- | --- | --- | --- | --- | --- | --- |
|  |  |  | Point estimate | Lower 95% CI | Upper 95% CI |  | Point estimate | Lower 95% CI | Upper 95% CI |  | Point estimate | Lower 95% CI | Upper 95% CI |  | Point estimate | Lower 95% CI | Upper 95% CI |
| Women | |  |  |  |  |  |  |  |  |  |  |  |  |  |  |  |  |
| Injuries | |  | 464 | 161 | 767 |  | 2682 | 1205 | 4247 |  | 2180 | 858 | 3590 |  | 243 | 67 | 425 |
|  | Unintentional injuries | | 372 | 111 | 633 |  | 1180 | 445 | 1969 |  | 1282 | 419 | 2204 |  | 151 | 20 | 286 |
|  |  | Transport injuries | 372 | 111 | 633 |  | 996 | 445 | 1548 |  | 1092 | 419 | 1765 |  | 124 | 20 | 227 |
|  |  | Poisonings | 0 | 0 | 0 |  | 38 | 0 | 87 |  | 27 | 0 | 63 |  | 3 | 0 | 7 |
|  |  | Falls | 0 | 0 | 0 |  | 6 | 0 | 14 |  | 14 | 0 | 31 |  | 6 | 0 | 13 |
|  |  | Fires, heat and hot substances | 0 | 0 | 0 |  | 73 | 0 | 168 |  | 76 | 0 | 175 |  | 8 | 0 | 17 |
|  |  | Drownings | 0 | 0 | 0 |  | 28 | 0 | 65 |  | 15 | 0 | 35 |  | 0 | 0 | 1 |
|  |  | Other unintentional injuries | 0 | 0 | 0 |  | 38 | 0 | 88 |  | 58 | 0 | 135 |  | 10 | 0 | 21 |
|  | Intentional injuries | | 92 | 50 | 134 |  | 1502 | 760 | 2277 |  | 897 | 438 | 1386 |  | 93 | 47 | 139 |
|  |  | Self-inflicted injuries | 0 | 0 | 0 |  | 108 | 0 | 248 |  | 93 | 0 | 214 |  | 6 | 0 | 13 |
|  |  | Violence | 92 | 50 | 134 |  | 1393 | 760 | 2026 |  | 803 | 438 | 1168 |  | 86 | 47 | 126 |
|  |  | Other intentional injuries | 0 | 0 | 0 |  | 1 | 0 | 3 |  | 1 | 0 | 3 |  | 0 | 0 | 0 |
| Men |  |  |  |  |  |  |  |  |  |  |  |  |  |  |  |  |  |
| Injuries | |  | 783 | 683 | 883 |  | 19341 | 10312 | 27368 |  | 11453 | 5778 | 16406 |  | 531 | 292 | 771 |
|  | Unintentional injuries | | 652 | 612 | 693 |  | 7146 | 3840 | 9449 |  | 6638 | 3322 | 9230 |  | 325 | 194 | 455 |
|  |  | Transport injuries | 652 | 612 | 693 |  | 4873 | 2876 | 5868 |  | 4611 | 2479 | 6020 |  | 203 | 157 | 248 |
|  |  | Poisonings | 0 | 0 | 0 |  | 240 | 102 | 378 |  | 199 | 83 | 315 |  | 3 | 1 | 5 |
|  |  | Falls | 0 | 0 | 0 |  | 129 | 55 | 204 |  | 223 | 93 | 353 |  | 30 | 9 | 50 |
|  |  | Fires, heat and hot substances | 0 | 0 | 0 |  | 412 | 175 | 649 |  | 483 | 201 | 766 |  | 22 | 7 | 37 |
|  |  | Drownings | 0 | 0 | 0 |  | 578 | 245 | 911 |  | 412 | 171 | 652 |  | 4 | 1 | 7 |
|  |  | Other unintentional injuries | 0 | 0 | 0 |  | 914 | 388 | 1440 |  | 710 | 295 | 1125 |  | 64 | 19 | 108 |
|  | Intentional injuries | | 131 | 71 | 190 |  | 12195 | 6471 | 17919 |  | 4815 | 2455 | 7176 |  | 207 | 97 | 316 |
|  |  | Self-inflicted injuries | 0 | 0 | 0 |  | 1483 | 629 | 2337 |  | 1293 | 538 | 2047 |  | 63 | 19 | 107 |
|  |  | Violence | 131 | 71 | 190 |  | 10680 | 5829 | 15532 |  | 3483 | 1901 | 5065 |  | 142 | 78 | 207 |
|  |  | Other intentional injuries | 0 | 0 | 0 |  | 32 | 13 | 50 |  | 40 | 17 | 63 |  | 1 | 0 | 2 |

Table 21: Deaths from injuries attributable to alcohol consumption (with harms to others): Sub-Saharan Africa West

|  |  |  | 0 to 14 years of age | | |  | 15 to 34 years of age | | |  | 35 to 64 years of age | | |  | 65 years of age and older | | |
| --- | --- | --- | --- | --- | --- | --- | --- | --- | --- | --- | --- | --- | --- | --- | --- | --- | --- |
|  |  |  | Point estimate | Lower 95% CI | Upper 95% CI |  | Point estimate | Lower 95% CI | Upper 95% CI |  | Point estimate | Lower 95% CI | Upper 95% CI |  | Point estimate | Lower 95% CI | Upper 95% CI |
| Women | |  |  |  |  |  |  |  |  |  |  |  |  |  |  |  |  |
| Injuries | |  | 965 | 129 | 1836 |  | 2841 | 1042 | 4640 |  | 2393 | 430 | 4356 |  | 310 | 82 | 547 |
|  | Unintentional injuries | | 745 | 0 | 1524 |  | 1044 | 65 | 2023 |  | 1665 | 125 | 3207 |  | 248 | 56 | 448 |
|  |  | Transport injuries | 745 | 0 | 1524 |  | 687 | 58 | 1317 |  | 672 | 24 | 1321 |  | 84 | 17 | 160 |
|  |  | Poisonings | 0 | 0 | 0 |  | 88 | 2 | 174 |  | 244 | 25 | 463 |  | 44 | 10 | 77 |
|  |  | Falls | 0 | 0 | 0 |  | 10 | 0 | 20 |  | 61 | 6 | 116 |  | 42 | 10 | 73 |
|  |  | Fires, heat and hot substances | 0 | 0 | 0 |  | 38 | 1 | 75 |  | 127 | 13 | 242 |  | 29 | 7 | 50 |
|  |  | Drownings | 0 | 0 | 0 |  | 102 | 2 | 201 |  | 188 | 19 | 357 |  | 8 | 2 | 14 |
|  |  | Other unintentional injuries | 0 | 0 | 0 |  | 119 | 3 | 235 |  | 373 | 38 | 709 |  | 42 | 10 | 75 |
|  | Intentional injuries | | 220 | 129 | 312 |  | 1797 | 976 | 2617 |  | 727 | 306 | 1149 |  | 62 | 26 | 99 |
|  |  | Self-inflicted injuries | 0 | 0 | 0 |  | 129 | 3 | 255 |  | 246 | 25 | 468 |  | 30 | 7 | 53 |
|  |  | Violence | 220 | 129 | 312 |  | 1668 | 974 | 2362 |  | 481 | 281 | 681 |  | 32 | 19 | 46 |
|  |  | Other intentional injuries | 0 | 0 | 0 |  | 0 | 0 | 0 |  | 0 | 0 | 0 |  | 0 | 0 | 0 |
| Men |  |  |  |  |  |  |  |  |  |  |  |  |  |  |  |  |  |
| Injuries | |  | 1377 | 1228 | 1526 |  | 14968 | 8058 | 21879 |  | 16628 | 6760 | 26496 |  | 1014 | 512 | 1517 |
|  | Unintentional injuries | | 1122 | 1079 | 1165 |  | 6958 | 3571 | 10345 |  | 12473 | 4664 | 20282 |  | 770 | 398 | 1142 |
|  |  | Transport injuries | 1122 | 1079 | 1165 |  | 2968 | 2188 | 3748 |  | 4713 | 1701 | 7726 |  | 288 | 223 | 352 |
|  |  | Poisonings | 0 | 0 | 0 |  | 486 | 168 | 803 |  | 1167 | 446 | 1888 |  | 19 | 7 | 31 |
|  |  | Falls | 0 | 0 | 0 |  | 141 | 49 | 232 |  | 667 | 255 | 1079 |  | 120 | 44 | 197 |
|  |  | Fires, heat and hot substances | 0 | 0 | 0 |  | 98 | 34 | 162 |  | 372 | 142 | 602 |  | 52 | 19 | 85 |
|  |  | Drownings | 0 | 0 | 0 |  | 1012 | 351 | 1674 |  | 2753 | 1052 | 4455 |  | 26 | 9 | 42 |
|  |  | Other unintentional injuries | 0 | 0 | 0 |  | 2253 | 781 | 3726 |  | 2801 | 1070 | 4532 |  | 265 | 96 | 434 |
|  | Intentional injuries | | 255 | 149 | 361 |  | 8010 | 4487 | 11533 |  | 4154 | 2095 | 6213 |  | 244 | 114 | 375 |
|  |  | Self-inflicted injuries | 0 | 0 | 0 |  | 797 | 276 | 1317 |  | 1634 | 624 | 2644 |  | 130 | 47 | 213 |
|  |  | Violence | 255 | 149 | 361 |  | 7213 | 4211 | 10216 |  | 2520 | 1471 | 3570 |  | 114 | 67 | 162 |
|  |  | Other intentional injuries | 0 | 0 | 0 |  | 0 | 0 | 0 |  | 0 | 0 | 0 |  | 0 | 0 | 0 |
