# Supplementary material for: Global burden of injuries attributable to alcohol consumption in 2004: a novel way of calculating the burden of injuries attributable to alcohol consumption
Source: Popul Health Metr. 2012 May 18;10:9. doi: 10.1186/1478-7954-10-9 (PMC3463441; doi:10.1186/1478-7954-10-9)
Supplement: Additional file 5 — Deaths from injuries attributable to alcohol consumption. [file 1478-7954-10-9-S5.docx]

Appendix 5. Population standardized alcohol-attributable years of life lived with disability per 100,000 people by GBD region for men and women

Figure 1. Population standardized alcohol-attributable years of life lived with disability per 100,000 people by GBD region for men

Figure 2. Population standardized alcohol-attributable years of life lived with disability per 100,000 people by GBD region for women
