# Supplementary material for: Global burden of injuries attributable to alcohol consumption in 2004: a novel way of calculating the burden of injuries attributable to alcohol consumption
Source: Popul Health Metr. 2012 May 18;10:9. doi: 10.1186/1478-7954-10-9 (PMC3463441; doi:10.1186/1478-7954-10-9)
Supplement: Additional file 6 — Population-standardized alcohol-attributable years of life lived with disability per 100,000 people by GBD region for men and women. [file 1478-7954-10-9-S6.docx]

Appendix 6. Alcohol-attributable injury caused by harms to others by global burden of disease region

| Table 1 Alcohol-attributable deaths caused by injuries from others by Global Burden of Disease region for 2004 | | | | | | | | | | | | | | | | | | | |
| --- | --- | --- | --- | --- | --- | --- | --- | --- | --- | --- | --- | --- | --- | --- | --- | --- | --- | --- | --- |
|  | Men | | | | | |  | | Women | | | | | |  | | Total | | |
|  | Point estimate | | Lower 95% confidence interval | | Upper 95% confidence interval | |  | | Point estimate | | Lower 95% confidence interval | | Upper 95% confidence interval | |  | | Point estimate | Lower 95% confidence interval | Upper 95% confidence interval |
| Asia, Pacific [High Income] | 970 | | 890 | | 1,050 | |  | | 420 | | 150 | | 700 | |  | | 1,390 | 1,030 | 1,750 |
| Asia, Central | 1,810 | | 1,540 | | 2,080 | |  | | 570 | | 270 | | 870 | |  | | 2,390 | 1,820 | 2,950 |
| Asia, East | 13,040 | | 11,290 | | 14,790 | |  | | 4,810 | | 1,220 | | 8,400 | |  | | 17,850 | 12,500 | 23,190 |
| Asia, South | 15,220 | | 11,160 | | 29,470 | |  | | 5,110 | | 220 | | 14,350 | |  | | 20,330 | 11,380 | 43,820 |
| Asia, Southeast | 9,580 | | 6,270 | | 12,890 | |  | | 2,080 | | 300 | | 4,060 | |  | | 11,660 | 6,570 | 16,950 |
| Australasia | 120 | | 110 | | 140 | |  | | 50 | | 30 | | 60 | |  | | 170 | 140 | 190 |
| Caribbean | 870 | | 660 | | 1,070 | |  | | 170 | | 80 | | 270 | |  | | 1,040 | 740 | 1,340 |
| Europe, Central | 2,690 | | 2,260 | | 3,120 | |  | | 940 | | 330 | | 1,560 | |  | | 3,630 | 2,590 | 4,680 |
| Europe, Eastern | 24,280 | | 16,080 | | 28,590 | |  | | 12,280 | | 8,030 | | 15,580 | |  | | 36,560 | 24,110 | 44,170 |
| Europe, Western | 3,090 | | 2,730 | | 3,450 | |  | | 1,040 | | 380 | | 1,710 | |  | | 4,130 | 3,110 | 5,160 |
| Latin America, Andean | 1,630 | | 1,040 | | 2,220 | |  | | 220 | | 70 | | 360 | |  | | 1,850 | 1,110 | 2,580 |
| Latin America, Central | 19,600 | | 13,880 | | 25,330 | |  | | 3,100 | | 1,530 | | 4,670 | |  | | 22,700 | 15,410 | 30,000 |
| Latin America, Southern | 920 | | 690 | | 1,140 | |  | | 160 | | 70 | | 250 | |  | | 1,080 | 760 | 1,390 |
| Latin America, Tropical | 19,970 | | 12,870 | | 27,070 | |  | | 2,320 | | 940 | | 3,690 | |  | | 22,280 | 13,810 | 30,750 |
| Northern Africa / Middle East | 2,200 | | 1,650 | | 3,410 | |  | | 550 | | 0 | | 1,840 | |  | | 2,750 | 1,650 | 5,250 |
| North America [High Income] | 5,690 | | 4,440 | | 6,930 | |  | | 1,940 | | 750 | | 3,130 | |  | | 7,630 | 5,200 | 10,060 |
| Oceania | 160 | | 130 | | 190 | |  | | 40 | | 20 | | 60 | |  | | 200 | 150 | 250 |
| Sub-Saharan Africa, Central | 4,590 | | 3,700 | | 5,490 | |  | | 1,000 | | 430 | | 1,580 | |  | | 5,600 | 4,130 | 7,060 |
| Sub-Saharan Africa, East | 13,370 | | 8,550 | | 18,180 | |  | | 3,710 | | 800 | | 6,790 | |  | | 17,080 | 9,350 | 24,970 |
| Sub-Saharan Africa, Southern | 17,460 | | 10,700 | | 22,510 | |  | | 4,390 | | 2,040 | | 6,750 | |  | | 21,860 | 12,740 | 29,260 |
| Sub-Saharan Africa, Western | 14,740 | | 10,310 | | 19,160 | |  | | 4,190 | | 1,420 | | 7,010 | |  | | 18,930 | 11,730 | 26,170 |
| World | 172,000 | | 120,900 | | 228,300 | |  | | 49,100 | | 19,100 | | 83,700 | |  | | 221,100 | 140,000 | 312,000 |
| Table 2 Alcohol-attributable PYLL caused by injuries from others by Global Burden of Disease region for 2004 | | | | | | | | | | | | | | | | | | | |
|  | | Men | | | | | |  | | Women | | | | | |  | Total | | |
|  | | Point estimate | | Lower 95% confidence interval | | Upper 95% confidence interval | |  | | Point estimate | | Lower 95% confidence interval | | Upper 95% confidence interval | |  | Point estimate | Lower 95% confidence interval | Upper 95% confidence interval |
| Asia, Pacific [High Income] | | 21,230 | | 19,400 | | 23,050 | |  | | 8,680 | | 3,340 | | 14,030 | |  | 29,910 | 22,740 | 37,080 |
| Asia, Central | | 47,680 | | 40,630 | | 54,720 | |  | | 14,460 | | 6,970 | | 21,950 | |  | 62,140 | 47,600 | 76,670 |
| Asia, East | | 346,400 | | 297,120 | | 395,690 | |  | | 116,760 | | 30,560 | | 202,960 | |  | 463,160 | 327,680 | 598,640 |
| Asia, South | | 392,890 | | 286,620 | | 765,380 | |  | | 127,940 | | 5,880 | | 355,810 | |  | 520,830 | 292,500 | 1,121,190 |
| Asia, Southeast | | 255,430 | | 167,520 | | 343,330 | |  | | 51,900 | | 7,450 | | 101,190 | |  | 307,320 | 174,970 | 444,520 |
| Australasia | | 3,350 | | 3,000 | | 3,700 | |  | | 1,200 | | 890 | | 1,520 | |  | 4,560 | 3,890 | 5,220 |
| Caribbean | | 23,380 | | 17,740 | | 29,030 | |  | | 4,710 | | 2,230 | | 7,190 | |  | 28,090 | 19,970 | 36,220 |
| Europe, Central | | 65,620 | | 55,200 | | 76,050 | |  | | 22,280 | | 8,160 | | 36,390 | |  | 87,900 | 63,360 | 112,440 |
| Europe, Eastern | | 600,920 | | 399,020 | | 689,010 | |  | | 287,230 | | 191,590 | | 353,640 | |  | 888,150 | 590,610 | 1,042,650 |
| Europe, Western | | 79,980 | | 70,570 | | 89,390 | |  | | 25,410 | | 9,600 | | 41,210 | |  | 105,380 | 80,170 | 130,600 |
| Latin America, Andean | | 38,970 | | 25,970 | | 51,980 | |  | | 5,850 | | 2,040 | | 9,650 | |  | 44,820 | 28,010 | 61,620 |
| Latin America, Central | | 560,380 | | 392,440 | | 728,320 | |  | | 85,740 | | 43,310 | | 128,160 | |  | 646,120 | 435,750 | 856,480 |
| Latin America, Southern | | 24,590 | | 18,310 | | 30,870 | |  | | 4,190 | | 1,850 | | 6,540 | |  | 28,780 | 20,160 | 37,400 |
| Latin America, Tropical | | 581,860 | | 370,020 | | 793,690 | |  | | 64,670 | | 27,310 | | 102,040 | |  | 646,530 | 397,330 | 895,730 |
| Northern Africa / Middle East | | 61,110 | | 45,120 | | 96,320 | |  | | 15,150 | | 0 | | 50,880 | |  | 76,260 | 45,120 | 147,200 |
| North America [High Income] | | 157,070 | | 121,110 | | 193,040 | |  | | 50,520 | | 20,330 | | 80,710 | |  | 207,590 | 141,440 | 273,750 |
| Oceania | | 4,470 | | 3,680 | | 5,250 | |  | | 1,020 | | 440 | | 1,600 | |  | 5,490 | 4,130 | 6,850 |
| Sub-Saharan Africa, Central | | 132,990 | | 106,950 | | 159,040 | |  | | 30,480 | | 13,110 | | 47,850 | |  | 163,480 | 120,060 | 206,900 |
| Sub-Saharan Africa, East | | 385,720 | | 246,490 | | 524,940 | |  | | 110,390 | | 23,490 | | 202,030 | |  | 496,110 | 269,990 | 726,970 |
| Sub-Saharan Africa, Southern | | 507,530 | | 309,170 | | 659,900 | |  | | 117,970 | | 55,390 | | 180,550 | |  | 625,510 | 364,570 | 840,450 |
| Sub-Saharan Africa, Western | | 423,370 | | 295,150 | | 551,580 | |  | | 127,800 | | 43,540 | | 213,380 | |  | 551,160 | 338,690 | 764,970 |
| World | | 4,714,900 | | 3,291,200 | | 6,264,300 | |  | | 1,274,400 | | 497,500 | | 2,159,300 | |  | 5,989,300 | 3,788,700 | 8,423,600 |

| Table 3 Alcohol-attributable YLD caused by injuries from others by Global Burden of Disease region for 2004 | | | | | | | | | | | |
| --- | --- | --- | --- | --- | --- | --- | --- | --- | --- | --- | --- |
|  | Men | | |  | Women | | |  | Total | | |
|  | Point estimate | Lower 95% confidence interval | Upper 95% confidence interval |  | Point estimate | Lower 95% confidence interval | Upper 95% confidence interval |  | Point estimate | Lower 95% confidence interval | Upper 95% confidence interval |
| Asia, Pacific [High Income] | 4,160 | 3,000 | 5,330 |  | 1,350 | 1,160 | 1,530 |  | 5,510 | 4,160 | 6,860 |
| Asia, Central | 5,530 | 3,860 | 7,200 |  | 2,580 | 2,200 | 2,960 |  | 8,110 | 6,060 | 10,150 |
| Asia, East | 28,930 | 20,960 | 36,900 |  | 14,410 | 12,130 | 16,680 |  | 43,340 | 33,090 | 53,580 |
| Asia, South | 47,010 | 28,060 | 108,620 |  | 24,380 | 19,040 | 43,880 |  | 71,380 | 47,100 | 152,490 |
| Asia, Southeast | 25,240 | 15,660 | 34,810 |  | 16,330 | 14,790 | 17,880 |  | 41,570 | 30,450 | 52,690 |
| Australasia | 220 | 200 | 240 |  | 100 | 100 | 110 |  | 330 | 300 | 350 |
| Caribbean | 6,620 | 4,470 | 8,770 |  | 1,670 | 1,340 | 1,990 |  | 8,290 | 5,820 | 10,770 |
| Europe, Central | 9,720 | 5,850 | 13,580 |  | 4,010 | 3,190 | 4,820 |  | 13,720 | 9,040 | 18,410 |
| Europe, Eastern | 105,510 | 68,610 | 142,350 |  | 59,080 | 47,940 | 70,230 |  | 164,590 | 116,550 | 212,580 |
| Europe, Western | 7,850 | 5,990 | 9,710 |  | 3,400 | 3,020 | 3,780 |  | 11,250 | 9,000 | 13,490 |
| Latin America, Andean | 4,190 | 2,710 | 5,680 |  | 1,350 | 1,180 | 1,530 |  | 5,540 | 3,880 | 7,200 |
| Latin America, Central | 125,900 | 75,770 | 176,020 |  | 20,190 | 16,180 | 24,210 |  | 146,090 | 91,950 | 200,230 |
| Latin America, Southern | 10,470 | 6,930 | 14,020 |  | 1,940 | 1,450 | 2,430 |  | 12,410 | 8,380 | 16,450 |
| Latin America, Tropical | 123,100 | 71,630 | 174,570 |  | 15,430 | 11,000 | 19,850 |  | 138,530 | 82,630 | 194,430 |
| Northern Africa / Middle East | 13,890 | 4,550 | 32,780 |  | 3,980 | 3,080 | 6,040 |  | 17,860 | 7,630 | 38,830 |
| North America [High Income] | 17,180 | 11,940 | 22,420 |  | 6,770 | 5,670 | 7,870 |  | 23,950 | 17,600 | 30,300 |
| Oceania | 300 | 240 | 360 |  | 280 | 270 | 290 |  | 570 | 500 | 650 |
| Sub-Saharan Africa, Central | 15,060 | 11,230 | 18,880 |  | 5,140 | 4,530 | 5,750 |  | 20,200 | 15,760 | 24,630 |
| Sub-Saharan Africa, East | 52,080 | 28,040 | 76,120 |  | 17,870 | 12,610 | 23,130 |  | 69,950 | 40,650 | 99,250 |
| Sub-Saharan Africa, Southern | 35,470 | 16,300 | 46,060 |  | 12,680 | 9,950 | 15,400 |  | 48,140 | 26,250 | 61,460 |
| Sub-Saharan Africa, Western | 57,140 | 35,640 | 78,650 |  | 19,490 | 14,870 | 24,110 |  | 76,630 | 50,500 | 102,760 |
| World | 695,600 | 421,600 | 1,013,100 |  | 232,400 | 185,700 | 294,500 |  | 928,000 | 607,300 | 1,307,600 |

| Table 4 Alcohol-attributable DALYs caused by injuries from others by Global Burden of Disease region for 2004 | | | | | | | | | | | |
| --- | --- | --- | --- | --- | --- | --- | --- | --- | --- | --- | --- |
|  | Men | | |  | Women | | |  | Total | | |
|  | Point estimate | Lower 95% confidence interval | Upper 95% confidence interval |  | Point estimate | Lower 95% confidence interval | Upper 95% confidence interval |  | Point estimate | Lower 95% confidence interval | Upper 95% confidence interval |
| Asia, Pacific [High Income] | 25,390 | 22,400 | 28,380 |  | 10,030 | 4,500 | 15,560 |  | 35,420 | 26,900 | 43,940 |
| Asia, Central | 53,200 | 44,490 | 61,910 |  | 17,040 | 9,170 | 24,910 |  | 70,250 | 53,660 | 86,830 |
| Asia, East | 375,330 | 318,080 | 432,580 |  | 131,170 | 42,700 | 219,640 |  | 506,500 | 360,780 | 652,220 |
| Asia, South | 439,900 | 314,690 | 873,990 |  | 152,310 | 24,920 | 399,690 |  | 592,210 | 339,600 | 1,273,680 |
| Asia, Southeast | 280,660 | 183,180 | 378,140 |  | 68,230 | 22,230 | 119,070 |  | 348,890 | 205,410 | 497,210 |
| Australasia | 3,570 | 3,200 | 3,950 |  | 1,310 | 980 | 1,630 |  | 4,880 | 4,180 | 5,580 |
| Caribbean | 30,010 | 22,210 | 37,800 |  | 6,380 | 3,580 | 9,180 |  | 36,390 | 25,790 | 46,980 |
| Europe, Central | 75,340 | 61,050 | 89,630 |  | 26,280 | 11,360 | 41,210 |  | 101,620 | 72,400 | 130,840 |
| Europe, Eastern | 706,430 | 467,630 | 831,360 |  | 346,320 | 239,520 | 423,870 |  | 1,052,740 | 707,150 | 1,255,230 |
| Europe, Western | 87,830 | 76,550 | 99,100 |  | 28,800 | 12,620 | 44,990 |  | 116,630 | 89,170 | 144,080 |
| Latin America, Andean | 43,170 | 28,680 | 57,650 |  | 7,200 | 3,220 | 11,170 |  | 50,360 | 31,900 | 68,830 |
| Latin America, Central | 686,280 | 468,210 | 904,340 |  | 105,930 | 59,490 | 152,370 |  | 792,210 | 527,700 | 1,056,720 |
| Latin America, Southern | 35,060 | 25,240 | 44,890 |  | 6,130 | 3,300 | 8,970 |  | 41,190 | 28,530 | 53,860 |
| Latin America, Tropical | 704,960 | 441,650 | 968,270 |  | 80,100 | 38,310 | 121,890 |  | 785,060 | 479,960 | 1,090,160 |
| Northern Africa / Middle East | 75,000 | 49,670 | 129,100 |  | 19,120 | 3,080 | 56,920 |  | 94,120 | 52,750 | 186,020 |
| North America [High Income] | 174,250 | 133,050 | 215,460 |  | 57,290 | 26,000 | 88,580 |  | 231,540 | 159,050 | 304,040 |
| Oceania | 4,760 | 3,920 | 5,610 |  | 1,300 | 710 | 1,890 |  | 6,060 | 4,630 | 7,490 |
| Sub-Saharan Africa, Central | 148,050 | 118,180 | 177,920 |  | 35,620 | 17,640 | 53,610 |  | 183,670 | 135,810 | 231,530 |
| Sub-Saharan Africa, East | 437,800 | 274,530 | 601,060 |  | 128,260 | 36,100 | 225,160 |  | 566,060 | 310,630 | 826,230 |
| Sub-Saharan Africa, Southern | 543,000 | 325,470 | 705,960 |  | 130,650 | 65,340 | 195,950 |  | 673,650 | 390,820 | 901,910 |
| Sub-Saharan Africa, Western | 480,510 | 330,780 | 630,230 |  | 147,290 | 58,410 | 237,500 |  | 627,800 | 389,190 | 867,730 |
| World | 5,410,500 | 3,712,900 | 7,277,300 |  | 1,506,800 | 683,200 | 2,453,800 |  | 6,917,300 | 4,396,000 | 9,731,100 |
